# Supplementary material for: Heterogeneous Evolution of Sex Chromosomes in the Torrent Frog Genus Amolops
Source: Int J Mol Sci. 2022 Sep 22;23(19):11146. doi: 10.3390/ijms231911146 (PMC9570394; doi:10.3390/ijms231911146)
Supplement: Supplementary file 1 [file ijms-23-11146-s001.zip › ijms-1904191-manuscript-supplementary-revised/manuscript-supplementary/Figure S1.pdf]

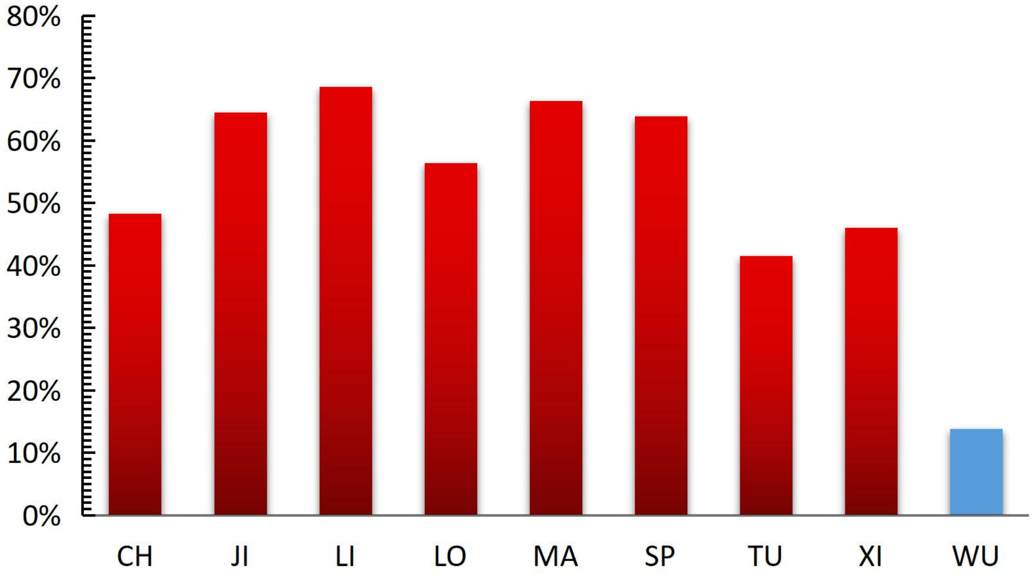

Figure S1. Rate of aligned sex-linked markers (y axes) for 9 species of *Amolops* aligned to the sex chromosome 5 of *A.mantzorum* (MA for *A. mantzorum*, SP for *A.* sp., JI for *A. jinjiangensis*, XI for *A. xinduqiao*, CH for *A. chunganensis,* LI for *A. lifanensis*, TU for *A. tuberodepressus*, LO for *A. loloensis*, WU for *A. wuyiensis*).
