# Supplementary material for: Heterogeneous Evolution of Sex Chromosomes in the Torrent Frog Genus Amolops
Source: Int J Mol Sci. 2022 Sep 22;23(19):11146. doi: 10.3390/ijms231911146 (PMC9570394; doi:10.3390/ijms231911146)
Supplement: Supplementary file 1 [file ijms-23-11146-s001.zip › ijms-1904191-manuscript-supplementary-revised/manuscript-supplementary/File S1.pdf]

File S1

Quality summary of *Amolops* by GBS or RAD sequencing. 182 individuals of 9 *Amolops* species of *A. chunganensis*, *A. jinjiangensis*, *A. lifanensis*, *A. loloensis*, *A. mantzorum*, *A.* sp., *A. tuberodepressus*, *A. wuyiensis*, *A. xinduqiao* were sequenced by GBS. At the same time, *A. jinjiangensis* and *A.*sp. were sequenced by RAD. The results after quality control of each species data were shown below. The results in Table S6 were cited from Luo et al. 2020 [34].

Table S1. Quality summary of *A. chunganensis* (12♂，8♀) by GBS sequencing

Serial	Sample	Raw Base(bp)	Clean Base(bp)	Effective Rate(%)	Error Rate(%)	Q20(%)	Q30(%)	GC Content(%)
1	xm5435M	2,306,490,624	2,306,469,888	100.00	0.04	96.66	91.13	43.55
2	xm5436M	2,275,203,168	2,275,186,176	100.00	0.04	96.41	90.62	44.33
3	xm5437M	2,240,751,456	2,240,728,992	100.00	0.04	95.97	89.37	44.52
4	xm5439M	2,684,068,128	2,684,040,768	100.00	0.04	97.27	92.56	44.64
5	xm5440M	2,238,629,184	2,238,607,584	100.00	0.04	96.46	90.73	43.67
6	xm5441M	2,400,928,416	2,400,906,816	100.00	0.04	96.86	91.67	44.05
7	xm5442M	2,509,143,840	2,509,117,632	100.00	0.04	96.62	91.10	43.84
8	xm5443M	1,815,708,960	1,815,708,960	100.00	0.04	96.28	90.16	43.23
9	xm5444M	2,399,499,936	2,399,480,064	100.00	0.04	96.01	89.62	43.64
10	xm140823M	2,199,256,992	2,199,239,136	100.00	0.04	96.44	90.59	44.25
11	xm140824M	2,682,247,680	2,682,221,760	100.00	0.04	96.21	90.09	43.95
12	xm140825M	2,093,039,424	2,093,019,264	100.00	0.04	96.35	90.58	42.74
13	xm5449F	2,299,457,664	2,299,436,640	100.00	0.04	95.90	89.35	44.64
14	xm5453F	1,729,766,016	1,729,747,296	100.00	0.04	95.94	89.37	44.34
15	xm5526F	2,290,128,768	2,290,108,608	100.00	0.04	96.33	90.44	44.49
16	xm5527F	2,325,840,480	2,325,821,184	100.00	0.04	96.81	91.70	44.50
17	xm5528F	1,682,022,816	1,682,007,840	100.00	0.04	96.50	90.85	44.12
18	xm5530F	2,161,843,776	2,161,824,192	100.00	0.04	96.40	90.68	44.22
19	xm5532F	2,139,924,096	2,139,906,816	100.00	0.04	97.00	92.15	44.23
20	xm140821F	2,702,542,176	2,702,520,576	100.00	0.04	96.35	90.45	43.28

Table S2. Quality summary of *A. jinjiangensis* (10♂，11♀) by GBS sequencing

Serial	Sample	Raw Base(bp)	Clean Base(bp)	Effective Rate(%)	Error Rate(%)	Q20(%)	Q30(%)	GC Content(%)
1	xm6119M	877,166,496	877,131,072	100.00	0.03	96.45	90.87	45.11
2	xm6124M	1,035,772,128	1,035,730,656	100.00	0.03	96.7	91.66	44.89
3	xm6153M	1,152,000,000	1,151,999,712	100.00	0.03	95.55	88.89	43.87
4	xm6155M	1,399,402,368	1,399,361,760	100.00	0.03	96.15	90.03	44.35
5	xm6159M	1,161,335,520	1,161,327,168	100.00	0.03	96.06	89.8	45.01
6	xm6160M	1,276,088,256	1,276,082,784	100.00	0.03	96.06	89.77	44.76
7	xm6161M	1,141,169,184	1,141,159,680	100.00	0.03	96.44	90.9	45.02
8	xm6170M	1,473,136,128	1,473,092,928	100.00	0.03	96.94	91.83	45.21
9	xm6172M	1,653,977,952	1,653,977,952	100.00	0.03	96.17	89.88	44.96
10	xm6176M	1,529,795,808	1,529,707,968	99.99	0.03	96.36	90.04	42.76
11	xm6107F	1,118,068,704	1,118,056,320	100.00	0.03	96.33	90.66	43.82
12	xm6111F	1,207,668,384	1,207,658,592	100.00	0.03	96.22	90.3	44.67
13	xm6118F	1,082,835,360	1,082,826,720	100.00	0.03	96.36	90.82	43.92
14	xm6120F	896,832,288	896,827,680	100.00	0.03	96.04	89.65	45.22
15	xm6121F	1,297,513,152	1,294,132,320	99.74	0.03	95.39	88.28	44.28
16	xm6129F	1,046,285,568	1,046,276,640	100.00	0.03	96.73	91.66	44.92
17	xm6145F	1,165,754,304	1,165,741,344	100.00	0.03	97.15	92.49	44.84
18	xm6151F	1,215,546,048	1,215,508,320	100.00	0.03	96.43	90.63	44.04
19	xm6154F	1,315,939,968	1,315,865,376	100.00	0.03	95.31	87.50	42.98
20	xm6157F	1,411,365,888	1,411,365,888	99.99	0.03	97.09	91.77	43.19
21	xm6158F	1,874,589,696	1,874,539,872	100.00	0.03	95.22	87.40	45.20

Table S3. Quality summary of *A. jinjiangensis* (3♂，4♀) by RAD sequencing

Serial	Sample	Raw Base(bp)	Clean Base(bp)	Effective Rate(%)	Error Rate(%)	Q20(%)	Q30(%)	GC Content(%)
1	xm6119M	9,953,211,300	9,577,712,400	96.23	0.03	96.48	91.92	43.37
2	xm6153M	8116745400	7749117600	95.55	0.03	96.655	92.38	42.94
3	xm6155M	16960781100	16332300600	96.345	0.03	96.69	92.495	43
4	xm6107F	9,698,685,600	9,640,968,000	99.40	0.03	96.55	91.88	42.27
5	xm6111F	10,568,827,800	10,222,118,400	96.72	0.03	96.47	91.88	43.16
6	xm6120F	9,549,554,700	9,272,001,300	97.09	0.03	96.70	92.40	42.84
7	xm6151F	9,073,832,100	8,784,897,300	96.82	0.03	96.51	91.99	42.92

Table S4. Quality summary of *A. lifanensis* (12♂，8♀) by GBS sequencing

Serial	Sample	Raw Base(bp)	Clean Base(bp)	Effective Rate(%)	Error Rate(%)	Q20(%)	Q30(%)	GC Content(%)
1	xm6846M	1,800,781,056	1,800,765,216	100.00	0.04	96.66	91.33	44.28
2	xm6847M	2,751,852,096	2,751,828,192	100.00	0.04	95.61	88.67	43.55
3	xm6848M	2,264,932,512	2,264,916,384	100.00	0.04	96.02	89.69	44.41
4	xm6850M	1,319,464,800	1,319,450,976	100.00	0.04	95.53	88.51	44.31
5	xm6851M	1,460,547,072	1,460,533,248	100.00	0.04	96.82	91.75	43.89
6	xm6852M	1,401,891,264	1,401,873,984	100.00	0.04	97.06	92.34	44.18
7	xm6853M	1,174,681,440	1,174,671,360	100.00	0.04	96.38	90.52	44.27
8	xm6854M	2,019,220,992	2,019,203,424	100.00	0.04	96.43	90.75	43.97
9	xm6855M	1,514,399,616	1,514,386,944	100.00	0.04	95.96	89.50	44.36
10	xm6856M	1,469,077,344	1,469,066,112	100.00	0.05	95.35	88.15	43.78
11	xm6857M	1,720,708,416	1,720,692,576	100.00	0.04	96.30	90.38	43.36
12	xm6864M	1,575,340,704	1,575,326,592	100.00	0.04	95.93	89.39	44.27
13	xm6428F	1,007,706,528	1,007,693,568	100.00	0.04	95.94	89.66	42.39
14	xm6431F	1,674,021,312	1,674,004,320	100.00	0.04	96.24	90.13	43.55
15	xm6436F	1,408,682,880	1,408,666,464	100.00	0.04	95.86	89.39	43.51
16	xm6437F	961,500,960	961,490,304	100.00	0.04	96.18	90.19	42.41
17	xm6849F	1,497,127,392	1,497,111,840	100.00	0.04	96.31	90.44	44.20
18	xm6867F	1,463,322,816	1,463,307,264	100.00	0.05	94.62	86.27	43.92
19	xm6878F	1,701,377,856	1,701,362,592	100.00	0.04	96.41	90.67	43.61
20	xm6892F	1,788,377,760	1,788,361,920	100.00	0.04	96.53	90.90	43.96

Table S5. Quality summary of *A. loloensis* (9♂，11♀) by GBS sequencing

Serial	Sample	Raw Base(bp)	Clean Base(bp)	Effective Rate(%)	Error Rate(%)	Q20(%)	Q30(%)	GC Content(%)
1	xm5561M	1,574,412,480	1,570,237,632	99.73	0.03	94.7	86.62	44.52
2	xm5562M	1,152,000,000	1,151,969,472	100	0.04	95.45	88.64	44.88
3	xm5573M	1,337,913,216	1,334,431,872	99.74	0.03	94.66	86.44	45
4	xm5575M	1,152,000,000	1,151,976,672	100	0.03	95.86	89.48	43.88
5	xm5579M	1,490,229,792	1,486,305,792	99.74	0.04	94.04	85.24	45.3
6	xm5581M	1,152,000,000	1,151,972,352	100	0.03	96.47	90.74	44.35
7	xm5585M	1,152,000,000	1,151,978,400	100	0.03	95.75	89.31	43.91
8	xm5586M	1,286,776,224	1,283,204,448	99.72	0.03	96	89.62	45.14
9	xm5592M	1,380,684,384	1,376,929,728	99.73	0.03	96.25	90.22	45
10	xm5560F	1,517,094,144	1,512,796,320	99.72	0.03	95.5	88.47	45.42
11	xm5572F	1,276,113,312	1,272,819,456	99.74	0.03	94.73	86.6	45.2
12	xm5574F	1,152,000,000	1,152,000,000	100	0.03	95.95	89.64	43.83
13	xm5576F	1,628,385,984	1,624,127,040	99.74	0.03	95.37	88.13	45.09
14	xm5577F	1,446,447,456	1,442,570,688	99.73	0.03	94.65	86.47	45.36
15	xm5578F	1,152,000,000	1,151,97,4080	100	0.03	95.64	89.03	44.34
16	xm5580F	1,152,000,000	1,151,966,304	100	0.04	95.06	87.72	44.77
17	xm5584F	1,511,993,376	1,508,133,024	99.74	0.03	95.38	88.13	45.02
18	xm5587F	1,350,221,184	1,346,569,344	99.73	0.03	95.5	88.42	45.12
19	xm5591F	1,466,617,248	1,462,538,592	99.72	0.03	95.71	88.94	45.06
20	xm5593F	1,460,370,528	1,456,305,120	99.72	0.03	95.42	88.28	45

Table S6. Quality summary of *A. mantzorum* (10♂，10♀) by GBS sequencing (Cite from: Luo et al. 2020) [34]

Serial	Sample	Raw Base(bp)	Clean Base(bp)	Effective Rate(%)	Error Rate(%)	Q20(%)	Q30(%)	GC Content(%)
1	xm5419M	1,115,742,816	944,460,576	84.65	0.04	94.39	86.31	44.22
2	xm5505M	1,235,666,304	1,129,111,200	91.38	0.04	94.51	86.02	43.11
3	xm5506M	1,362,431,232	1,142,590,464	83.86	0.04	94.1	85.74	44.33
4	xm5509M	1,405,836,288	1,280,830,176	91.11	0.04	94.32	85.68	43.16
5	xm5510M	1,183,251,744	969,465,024	81.93	0.04	93.27	84.06	44.49
6	xm5550M	918,683,424	839,546,784	91.39	0.04	94.84	86.75	44.25
7	xm5551M	991,366,560	795,010,464	80.19	0.04	92.48	82.41	44.66
8	xm5553M	1,378,397,952	1,088,229,888	78.95	0.04	91.9	81.28	44.23
9	xm5767M	894,136,896	668,430,720	74.76	0.06	90.31	78.15	44.33
10	xm5768M	1,329,713,568	1,088,465,184	81.86	0.04	93.2	83.93	44.33
11	xm5418F	1,048,606,848	945,563,328	90.17	0.04	94	85.06	42.76
12	xm5501F	774,791,712	692,184,384	89.34	0.04	93.61	84.09	44.47
13	xm5502F	1,004,658,048	908,922,240	90.47	0.04	94.1	85.19	44.22
14	xm5503F	1,241,951,040	1,007,745,120	81.14	0.04	93.15	83.69	44.7
15	xm5766F	1,343,048,544	1,203,297,696	89.59	0.04	93.61	84.19	44.41
16	xm5793F	967,158,720	863,706,240	89.3	0.04	93.35	83.61	44.2
17	xm5799F	448,333,056	401,740,416	89.61	0.04	93.67	84.38	42.42
18	xm5841F	994,315,680	903,437,280	90.86	0.04	94.2	85.38	43.86
19	xm5842F	1,237,210,560	1,123,719,840	90.83	0.04	94.23	85.44	44.01
20	xm5843F	862,236,288	772,805,664	89.63	0.04	93.48	83.92	43.45

Table S7. Quality summary of *A. mantzorum* (10♂，10♀) by GBS sequencing

Serial	Sample	Raw Base(bp)	Clean Base(bp)	Effective Rate(%)	Error Rate(%)	Q20(%)	Q30(%)	GC Content(%)
1	xm5419M	1,950,262,272	1,893,243,168	97.08	0.04	94.17	85.75	44.21
2	xm5505M	1,485,036,000	1,429,529,472	96.26	0.04	94.55	86.59	44.40
3	xm5506M	2,053,156,896	2,008,155,744	97.81	0.04	94.38	85.72	44.30
4	xm5509M	2,095,875,072	2,007,452,160	95.78	0.03	96.13	90.03	44.46
5	xm5510M	2,034,360,576	1,930,174,560	94.88	0.03	96.39	90.62	44.48
6	xm5550M	1,925,852,544	1,863,956,160	96.79	0.03	95.42	88.13	44.42
7	xm5551M	1,885,178,880	1,823,272,416	96.72	0.03	95.64	88.76	44.25
8	xm5553M	2,161,293,120	2,108,211,552	97.54	0.03	94.77	86.56	44.55
9	xm5767M	2,732,696,352	2,674,291,104	97.86	0.04	94.14	85.24	44.22
10	xm5768M	2,497,263,264	2,441,994,624	97.79	0.03	95.56	88.50	44.03
11	xm5418F	1,535,963,040	1,493,069,760	97.21	0.04	94.38	86.01	43.88
12	xm5501F	1,436,913,792	1,395,181,440	97.10	0.04	93.93	85.02	44.19
13	xm5502F	1,188,155,232	1,145,865,888	96.44	0.04	93.98	85.13	44.22
14	xm5503F	1,456,224,480	1,403,925,696	96.41	0.04	94.60	86.66	44.22
15	xm5766F	1,402,211,520	1,346,807,520	96.05	0.03	95.24	88.16	44.23
16	xm5793F	1,749,920,832	1,701,066,240	97.21	0.04	94.87	87.24	44.14
17	xm5799F	1,676,654,208	1,636,010,208	97.58	0.04	94.85	87.22	44.04
18	xm5841F	2,085,861,024	2,015,131,680	96.61	0.03	95.47	88.66	44.10
19	xm5842F	1,720,643,040	1,666,523,808	96.85	0.03	95.02	87.65	44.26
20	xm5843F	2,191,070,016	2,152,321,344	98.23	0.04	93.77	84.75	43.98

Table S8. Summary of GBS sequencing quality for *A.* sp. (8♂，9♀)

Serial	Sample	Raw Base(bp)	Clean Base(bp)	Effective Rate(%)	Error Rate(%)	Q20(%)	Q30(%)	GC Content(%)
1	xm5850M	1552468320	1548288864	99.73	0.03	95.23	87.88	45.23
2	xm5859M	1535858496	1531671552	99.73	0.03	96.22	90.13	44.86
3	xm5861M	1152000000	1151979552	100	0.03	95.63	89.06	44.21
4	xm5878M	1152000000	1151980992	100	0.03	95.64	89.06	44.43
5	xm6844M	1346936832	1343374272	99.74	0.04	94.34	85.77	45.12
6	xm6860M	1152000000	1151975232	100	0.04	95.2	88.06	44.49
7	xm6861M	1152000000	1151999712	100	0.04	95.23	88.11	44.62
8	xm6862M	1473472224	1469464992	99.73	0.03	94.41	86.03	45.39
9	xm5849F	1152000000	1151965728	100	0.03	95.73	89.26	44.75
10	xm5857F	1326930912	1323632736	99.75	0.04	92.53	82.12	45.33
11	xm5858F	1515288096	1511348832	99.74	0.04	93.88	84.94	45.05
12	xm5860F	1152000000	1151978112	100	0.03	96.06	90.03	44.45
13	xm5869F	1462565664	1458739008	99.74	0.03	95.6	88.66	45
14	xm6842F	1152000000	1151999424	100	0.03	96.19	90.25	44.09
15	xm6843F	1152000000	1151999712	100	0.03	95.93	89.67	44.23
16	xm6858F	1152000000	1152000000	100	0.04	95.46	88.62	43.34
17	xm6859F	1152000000	1152000000	100	0.04	95.3	88.22	43.94

Table S9. Summary of RAD-seq quality for *A.* sp. (5♂，4♀)

Serial	Sample	Raw Base(bp)	Clean Base(bp)	Effective Rate(%)	Error Rate(%)	Q20(%)	Q30(%)	GC Content(%)
1	xm5850M	11789933400	11492266500	97.575	0.03	96.07	91.28	42.56
2	xm5859M	12,578,748,900	12,390,646,200	98.50	0.03	96.88	92.59	42.20
3	xm5861M	11,180,502,000	10,989,065,700	98.29	0.03	96.99	92.86	42.14
4	xm6844M	8,825,242,500	8,674,115,700	98.29	0.03	96.90	92.66	42.23
5	xm6860M	10,371,290,400	10,303,875,600	99.35	0.03	96.50	91.78	42.26
6	xm5858F	9570614700	9343718100	97.8	0.03	95.86	90.435	42.68
7	xm5860F	8596305900	8375899800	97.545	0.03	95.745	90.295	42.715
8	xm6858F	13220097900	12969348600	98.175	0.03	95.725	90.215	42.8
9	xm6859F	8512762200	8276329200	97.36	0.03	96.07	91.215	42.46

Table S10. Quality summary of *A. tuberodpressus* (12♂，12♀) by GBS sequencing

Serial	Sample	Raw Base(bp)	Clean Base(bp)	Effective Rate(%)	Error Rate(%)	Q20(%)	Q30(%)	GC Content(%)
1	PJ112M	1,803,514,752	1,803,492,000	100.00	0.04	96.36	90.52	43.68
2	PJ113M	2,128,239,072	2,128,216,032	100.00	0.04	96.24	90.29	43.52
3	PJ117M	2,325,046,176	2,325,018,240	100.00	0.04	95.83	89.24	44.12
4	PJ146M	1,815,201,792	1,815,181,344	100.00	0.04	97.05	92.05	43.88
5	PJ147M	1,689,610,176	1,689,588,000	100.00	0.04	96.56	90.85	43.90
6	PJ149M	1,188,915,840	1,188,899,136	100.00	0.04	96.47	90.73	43.34
7	PJ150M	1,122,724,224	1,122,708,672	100.00	0.04	95.88	89.34	41.95
8	PJ151M	1,454,503,392	1,454,484,960	100.00	0.04	95.62	88.80	42.34
9	PJ152M	2,022,338,016	2,022,314,112	100.00	0.04	95.62	88.75	43.63
10	PJ153M	1,960,403,040	1,960,380,576	100.00	0.04	95.61	88.66	43.22
11	PJ154M	1,927,712,736	1,927,686,816	100.00	0.04	96.11	89.95	43.61
12	PJ155M	2,900,799,072	2,900,764,800	100.00	0.04	96.74	91.50	43.93
13	PJ114F	2,173,393,728	2,173,368,960	100.00	0.04	96.82	91.72	43.23
14	PJ115F	2,435,723,712	2,435,698,368	100.00	0.04	96.48	90.91	44.01
15	PJ116F	1,747,152,000	1,747,130,400	100.00	0.04	95.36	88.11	43.05
16	PJ119F	1,363,373,280	1,363,355,712	100.00	0.04	96.08	89.89	43.64
17	PJ120F	2,553,984,864	2,553,949,728	100.00	0.05	95.35	88.06	43.91
18	PJ121F	2,731,163,904	2,731,133,088	100.00	0.04	96.88	91.78	44.03
19	PJ143F	1,834,582,464	1,834,555,968	100.00	0.04	96.39	90.59	43.19
20	PJ144F	2,217,320,640	2,217,290,688	100.00	0.04	96.27	90.24	44.01
21	PJ145F	2,167,579,008	2,167,553,376	100.00	0.04	95.62	88.68	43.76
22	PJ148F	1,703,333,952	1,703,314,656	100.00	0.04	96.68	91.28	43.96
23	PJ161F	2,333,198,592	2,333,173,536	100.00	0.04	97.09	92.25	44.08
24	PJ7406F	2,035,731,744	2,035,704,096	100.00	0.04	96.24	90.16	44.20

Table S11. Quality summary of *A. wuyiensis* (8♂，12♀) by GBS sequencing

Serial	Sample	Raw Base(bp)	Clean Base(bp)	Effective Rate(%)	Error Rate(%)	Q20(%)	Q30(%)	GC Content(%)
1	YSQ08M	1,441,715,040	1,441,640,448	99.99	0.04	96.15	90.19	43.66
2	YSQ09M	1,771,709,472	1,771,613,568	99.99	0.04	95.95	89.53	43.01
3	YSQ36M	2,356,529,472	2,356,494,048	100.00	0.04	95.78	89.11	44.17
4	YSQ40M	1,859,647,104	1,859,630,112	100.00	0.05	94.18	85.43	43.70
5	YSQ41M	1,817,822,880	1,817,804,160	100.00	0.04	96.16	90.12	43.06
6	YSQ44M	1,057,004,928	1,056,945,888	99.99	0.04	95.86	89.54	43.36
7	YSQ56M	1,733,434,560	1,733,346,432	99.99	0.04	96.12	90.12	43.02
8	YSQ59M	971,926,848	971,879,328	100.00	0.05	95.31	88.18	43.64
9	YSQ18F	1,503,768,384	1,503,673,056	99.99	0.03	96.66	91.36	43.85
10	YSQ19F	1,571,986,944	1,571,917,248	100.00	0.03	95.94	89.68	43.45
11	YSQ20F	1,582,827,552	1,582,764,768	100.00	0.03	95.19	87.89	43.67
12	YSQ35F	1,567,900,512	1,567,881,216	100.00	0.04	96.14	90.11	43.25
13	YSQ37F	2,583,048,384	2,583,018,432	100.00	0.05	95.25	87.87	43.70
14	YSQ38F	2,735,546,976	2,735,519,040	100.00	0.04	96.27	90.24	43.59
15	YSQ39F	1,696,559,616	1,696,538,880	100.00	0.04	95.66	88.85	44.08
16	YSQ42F	2,159,686,080	2,159,663,616	100.00	0.04	96.37	90.53	43.88
17	YSQ43F	1,920,002,400	1,919,964,672	100.00	0.03	96.03	89.54	43.02
18	YSQ45F	1,042,810,848	1,042,755,552	99.99	0.04	95.58	88.94	43.41
19	YSQ46F	1,343,911,680	1,343,846,016	100.00	0.04	95.95	89.77	43.08
20	YSQ57F	1,599,984,000	1,599,909,696	100.00	0.04	95.94	89.71	43.78

Table S12. Quality summary of *A. xinduqiao* (10♂，10♀) by GBS sequencing

Serial	Sample	Raw Base(bp)	Clean Base(bp)	Effective Rate(%)	Error Rate(%)	Q20(%)	Q30(%)	GC Content(%)
1	xm6962M	3,037,070,880	2,999,713,248	98.04	0.03	95.75	89.06	44.62
2	xm6963M	2,871,851,328	2,808,228,672	96.57	0.03	96.86	92.00	44.68
3	xm6965M	3,403,996,128	3,360,809,376	98.06	0.03	96.46	91.03	44.76
4	xm6966M	3,146,573,664	3,095,291,520	97.54	0.03	96.64	91.58	45.05
5	xm6969M	3,248,957,952	3,198,638,592	97.78	0.03	96.56	91.23	44.84
6	xm6970M	2,451,777,984	2,417,828,544	97.80	0.03	96.27	90.42	44.15
7	xm6972M	2,899,848,384	2,847,703,968	97.39	0.03	96.09	89.89	44.24
8	xm6973M	3,447,077,760	3,399,854,976	97.91	0.03	95.86	89.35	44.73
9	xm6974M	3,140,776,512	3,080,302,848	97.00	0.03	95.88	89.38	45.01
10	xm6988M	3,223,574,784	3,172,661,856	97.38	0.03	96.40	90.83	45.00
11	xm6961F	3,150,419,040	3,114,325,440	98.14	0.03	94.72	86.51	44.12
12	xm6964F	2,423,266,272	2,405,527,776	98.72	0.04	92.65	82.10	44.21
13	xm6967F	3,046,049,568	2,983,961,376	97.00	0.03	95.62	88.73	44.34
14	xm6971F	3,512,104,128	3,432,028,032	96.87	0.03	95.80	89.09	44.48
15	xm6975F	3,258,797,184	3,211,950,240	97.79	0.03	96.45	90.91	44.13
16	xm6976F	2,823,986,592	2,780,961,408	97.52	0.03	96.36	90.78	44.69
17	xm6978F	3,006,267,552	2,962,451,808	97.68	0.03	96.06	90.06	44.70
18	xm6979F	2,947,009,824	2,901,694,464	97.40	0.03	96.62	91.30	44.41
19	xm6980F	2,999,314,080	2,954,251,296	97.28	0.03	96.50	91.03	44.61
20	xm6986F	3,277,192,320	3,211,748,064	96.91	0.03	96.31	90.60	44.84
