# Supplementary material for: Heterogeneous Evolution of Sex Chromosomes in the Torrent Frog Genus Amolops
Source: Int J Mol Sci. 2022 Sep 22;23(19):11146. doi: 10.3390/ijms231911146 (PMC9570394; doi:10.3390/ijms231911146)
Supplement: Supplementary file 1 [file ijms-23-11146-s001.zip › ijms-1904191-manuscript-supplementary-revised/manuscript-supplementary/File S10.pdf]

File S10

Sample size and location information. Bold font samples were used for GBS sequencing. The underlined samples were used for sex-linked markers sequences verification. The samples with # were used to optimize the parameter test. Add * individuals for RAD-seq sequencing at the same time. The samples with $ were used for sequencing GBS simplified genome again (Increased sequencing depth). Male individuals are represented by the symbol "M" and female individuals by the symbol "F". The voucher specimens were deposited in the Herpetological Museum of Chengdu Institute of Biology, Chinese Academy of Sciences.

1. *Amolops chunganensis* (n=20, 12M, 8F). Jianzigou Village, Gansu Province, China (N32°41'16.404", E105°15'34.02").

xm5435M、xm5436M、xm5437M、#xm5439M、xm5440M、#xm5441M、#xm5442M、xm5443M、xm5444M、xm140823M、#xm140824M、xm140825M、#xm5449F、xm5453F、#xm5526F、#xm5527F、xm5528F、xm5530F、xm5532F、#xm140821F、xm5445M、xm5446M、xm5447M、xm5448M、xm5450M、xm5451M、xm5452M、xm5454M、xm5455M、xm5456M、xm5332F、xm5333F、xm5334F、xm5511F、xm5512F、xm5515F

2. *Amolops jinjiangensis* (n=21, 10M, 11F). Gulong Village, Yunnan Province, China (N28°12′43.86″, E99°14′43.38″).

***xm6119M、xm6124M、*xm6153M、*#xm6155M、xm6159M、xm6160M、xm6161M、#xm6170M、#xm6172M、#xm6176M、*xm6107F、*xm6111F、xm6118F、*xm6120F、#xm6121F、xm6129F、xm6145F、*xm6151F、#xm6154F、#xm6157F、#xm6158F、**xm6103M、xm6106M、xm6115M、xm6123M、xm6124M、xm6125M、xm6130M、xm6146M、xm6156M、xm6168M、xm6108F、xm6112F、xm6152F、xm6162F、xm6163F、xm6169F、xm6171F、xm6173F、xm6174F、xm6177F

3. *Amolops lifanensis* (n=20, 12M, 8F). Kuikuizhai Village, Sichuan Province, China (N31°37′18.66″, E103°9′47.35″).

#xm6846M、#xm6847M、#xm6848M、xm6850M、xm6851M、xm6852M、xm6853M、#xm6854M、xm6855M、xm6856M、xm6857M、xm6864M、xm6428F、#xm6431F、xm6436F、xm6437F、#xm6849F、xm6867F、#xm6878F、#xm6892F、xm6429M、xm6432M、xm6433M、xm6434M、xm6435M、xm6865M、xm6868M、xm6869M、xm6870M、xm6871M

4. *Amolops loloensis* (n=20, 9M, 11F). Jiefang Village, Sichuan Province, China (N27°51'42.12", E102°34'24.24″).

#xm5561M、xm5562M、#xm5573M、xm5575M、#xm5579M、xm5581M、xm5585M、xm5586M、#xm5592M、#xm5560F、xm5572F、xm5574F、#xm5576F、xm5577F、xm5578F、xm5580F、xm5584F、xm5587F、#xm5591F、#xm5593F、xm5559M、xm5589M、xm5590M、PJ32M、PJ36M、PJ38M、PJ41M、PJ42M、PJ45M、PJ46M、xm5563F、xm5564F、xm5570F、xm5571F、xm5582F、xm5583F、xm5588F、PJ30F、PJ31F、PJ33F

5. *Amolops mantzorum* (n=20, 10M, 10F). Yanjingping Village, baoxing, Sichuan, China (N30°32'4.8", E102°54'50.6").

$xm5419M、$xm5505M、$xm5506M、$#xm5509M、$#xm5510M、$xm5550M、$xm5551M、$#xm5553M、$xm5767M、$#xm5768M、$xm5418F、$xm5501F、$xm5502F、$xm5503F、$#xm5766F、$xm5793F、$xm5799F、$#xm5841F、$#xm5842F、$#xm5843F、xm4333M、xm4405M、xm4450M、xm5226M、xm5259M、xm5261M、xm5305M、xm5833M、xm5835M、xm5856M、xm3155F、xm3157F、xm4336F、xm4352F、xm4406F、xm4449F、xm4490F、xm5223F、xm5806F、xm5807F

6. *Amolops s*p. (n=17, 8M, 9F). Baodingshan Village, Sichuan Province, China (N31°47′59.49″, E103°58′31.92″). *Amolops* sp., that was being regarded as the northern lineage of the *A. mantzorum*, is a putative species of torrent frogs delimited by Lu et al. (2014) [45], mainly distributed in the Minshan Mountains of the Southwest China. The phylogenetic inference by Lu et al. (2014) [45] with a mitochondrial gene (*cytb*) revealed the central lineage (including those specimens from the type locality) and northern lineage in the nominal species *A. mantzorum,* suggesting two cryptic species.

***#xm5850M、*#xm5859M、*xm5861M、xm5878M、*#xm6844M、*xm6860M、xm6861M、#xm6862M、xm5849F、#xm5857F、*#xm5858F、*xm5860F、#xm5869F、#xm6842F、xm6843F、*xm6858F、*xm6859F、**xm3026M、xm5500M、xm5537M、xm5852M、xm5864M、xm5866M、xm5870M、xm5872M、xm5876M、xm6863M、xm5499F、xm5536F、xm5540F、xm6845F、xm5851F、xm5862F、xm5863F、xm5865F、xm5867F、xm5871F

7. *Amolops tuberodepressus* (n=24, 12M, 12F). Tongjiashan Village, Yunnan Province, China (N24°6'37.8792", E101°26'16.1592").

PJ112M、#PJ113M、#PJ117M、PJ146M、PJ147M、PJ149M、PJ150M、PJ151M、#PJ152M、#PJ153M、PJ154M、PJ155M、PJ114F、#PJ115F、PJ116F、PJ119F、#PJ120F、#PJ121F、PJ143F、PJ144F、PJ145F、PJ148F、#PJ161F、PJ7406F、PJ110M、PJ111M、PJ118M、PJ162M、PJ163M、PJ164M、xm7407M、xm7409M、PJ109F、xm7408F

8. *Amolops wuyiensis* (n=20, 8M, 12F). Huangkeng Village, Fujian Province, China (N27°45′09.77″, E117°38′24.62″).

YSQ08M、#**YSQ09M**、#**YSQ36M**、#**YSQ40M**、#**YSQ41M**、**YSQ44M**、**YSQ56M**、**YSQ59M**、**YSQ18F**、**YSQ19F**、**YSQ20F**、**YSQ35F**、#**YSQ37F**、#**YSQ38F**、**YSQ39F**、#**YSQ42F**、#**YSQ43F**、**YSQ45F**、**YSQ46F**、**YSQ57F**、YSQ47F、YSQ48F、YSQ49F、YSQ50F、YSQ51F、YSQ52F、YSQ53F、YSQ54F、YSQ55F、YSQ58F

9. *Amolops xinduqiao* (n=20, 10M,10F). Xiabaisan village, Sichuan, China (N30°30'05.91", E101°30′05.07″).

xm6962M、**xm6963M**、#**xm6965M**、**xm6966M**、#**xm6969M**、**xm6970M**、**xm6972M**、#**xm6973M**、**xm6974M**、#**xm6988M**、#**xm6961F**、**xm6964F**、**xm6967F**、#**xm6971F**、#**xm6975F**、**xm6976F**、**xm6978F**、**xm6979F**、**xm6980F**、#**xm6986F**、xm6987M、xm6990M、xm6992M、xm6993M、xm6994M、xm6996M、xm6997M、xm6998M、xm7000M、xm7001M、xm6989F、xm6991F、xm6995F、xm6999F、xm7008F、xm7070F、xm7072F
