# Supplementary material for: Heterogeneous Evolution of Sex Chromosomes in the Torrent Frog Genus Amolops
Source: Int J Mol Sci. 2022 Sep 22;23(19):11146. doi: 10.3390/ijms231911146 (PMC9570394; doi:10.3390/ijms231911146)

File S2

The results from *Process_radtags* of *Amolops* (*A. chunganensis*, *A. jinjiangensis*, *A. lifanensis*, *A. loloensis*, *A. mantzorum*, *A.* sp., *A. tuberodepressus*, *A. wuyiensis*, *A. xinduqiao*). Abscissa represents the number of reads, and ordinate is the sample name of the individual. Male individuals are represented by the symbol “M” and female individuals by the symbol “F”.

A. chunganensis (GBS data)

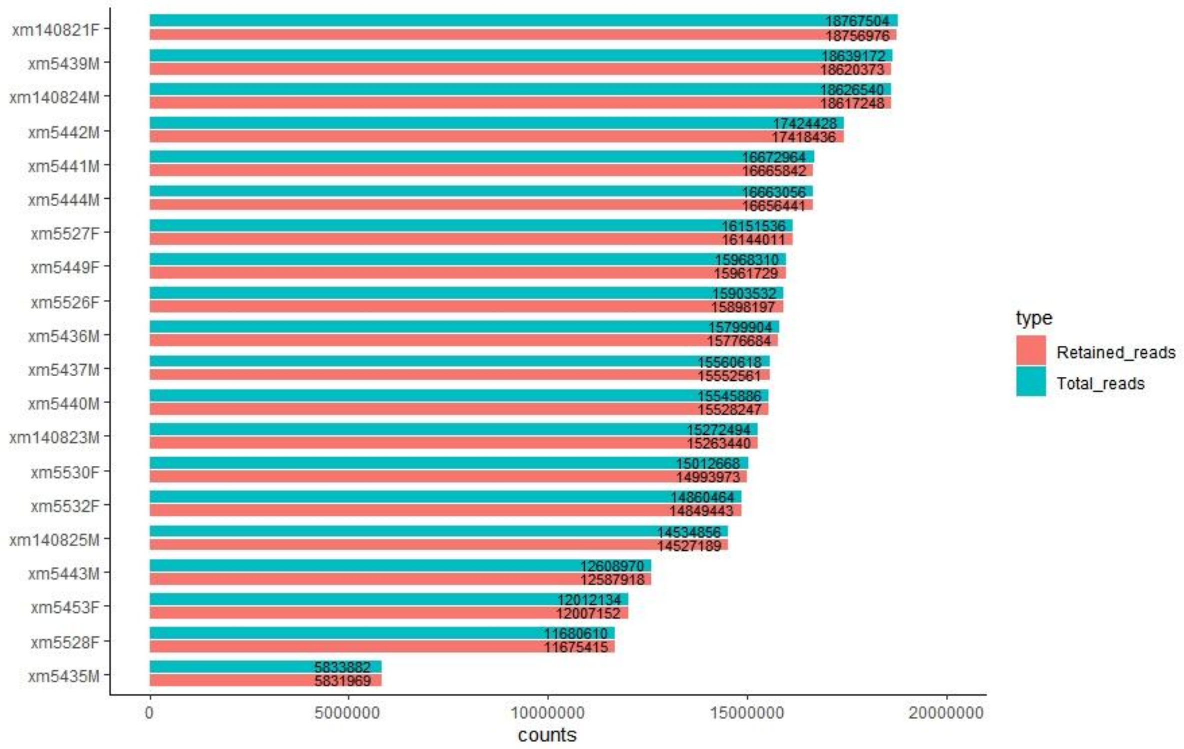

A. jinjiangensis (GBS data)

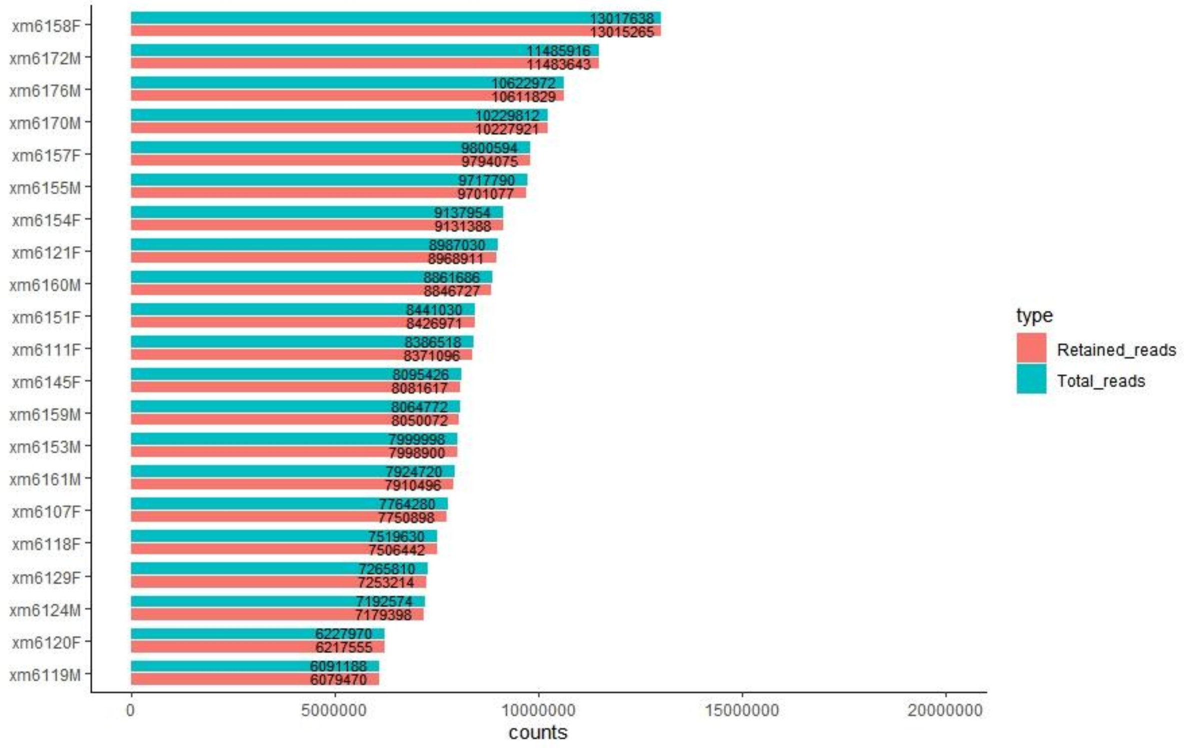

A. jinjiangensis (RAD-seq data)

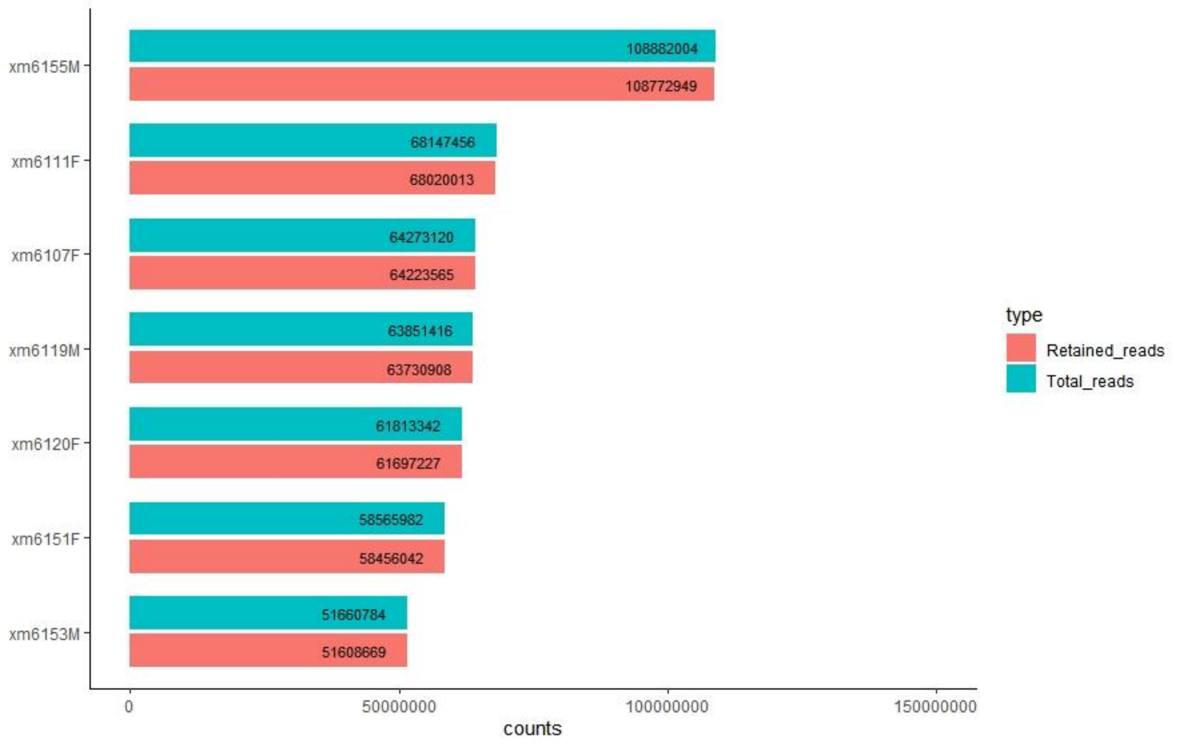

A. lifanensis (GBS data)

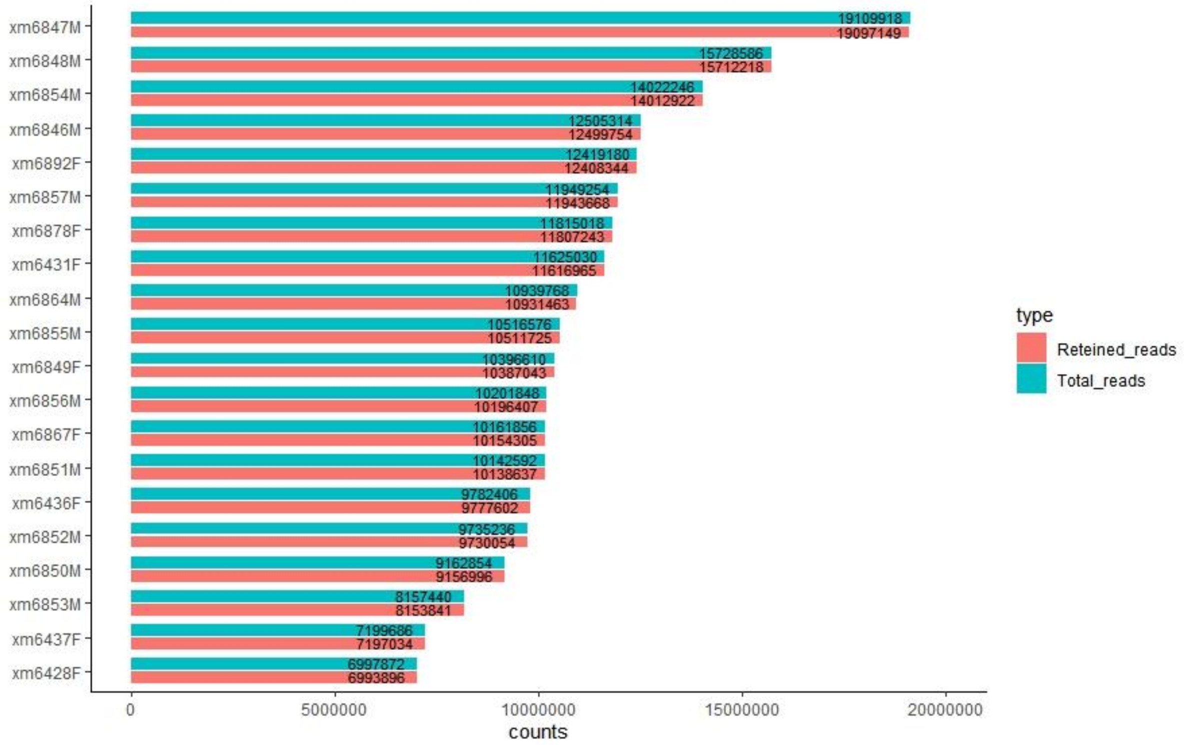

A. loloensis (GBS data)

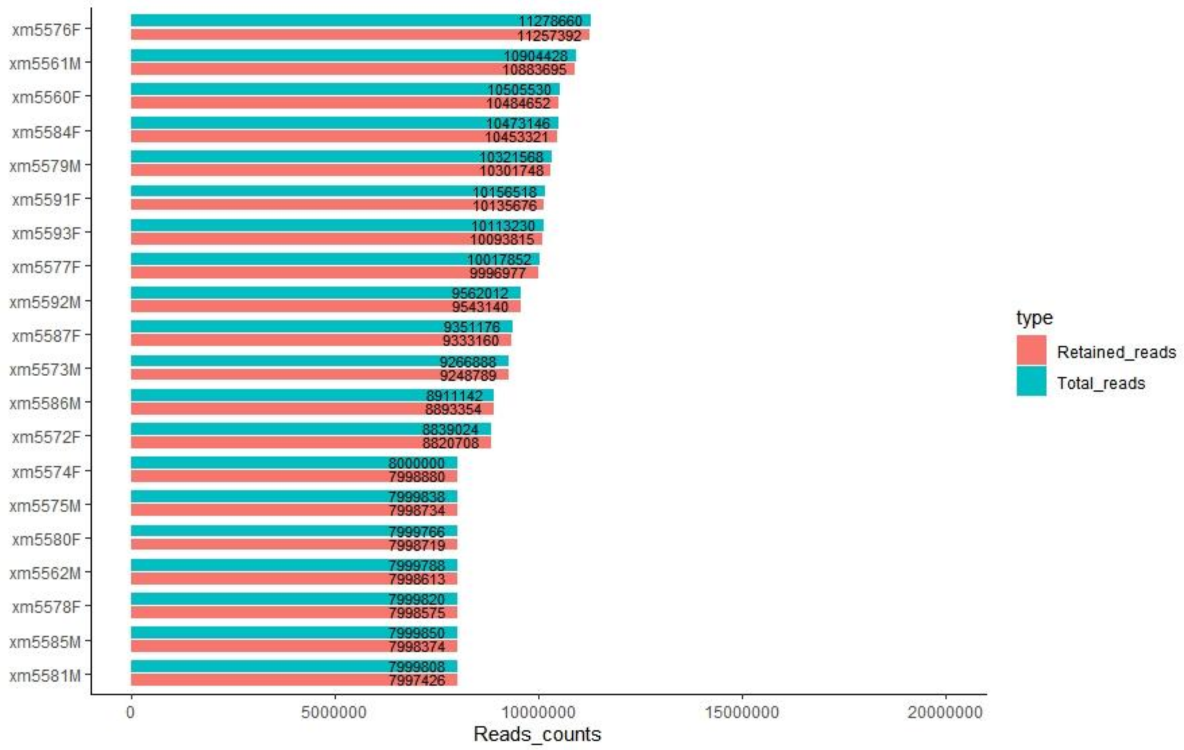

A. mantzorum (GBS data)

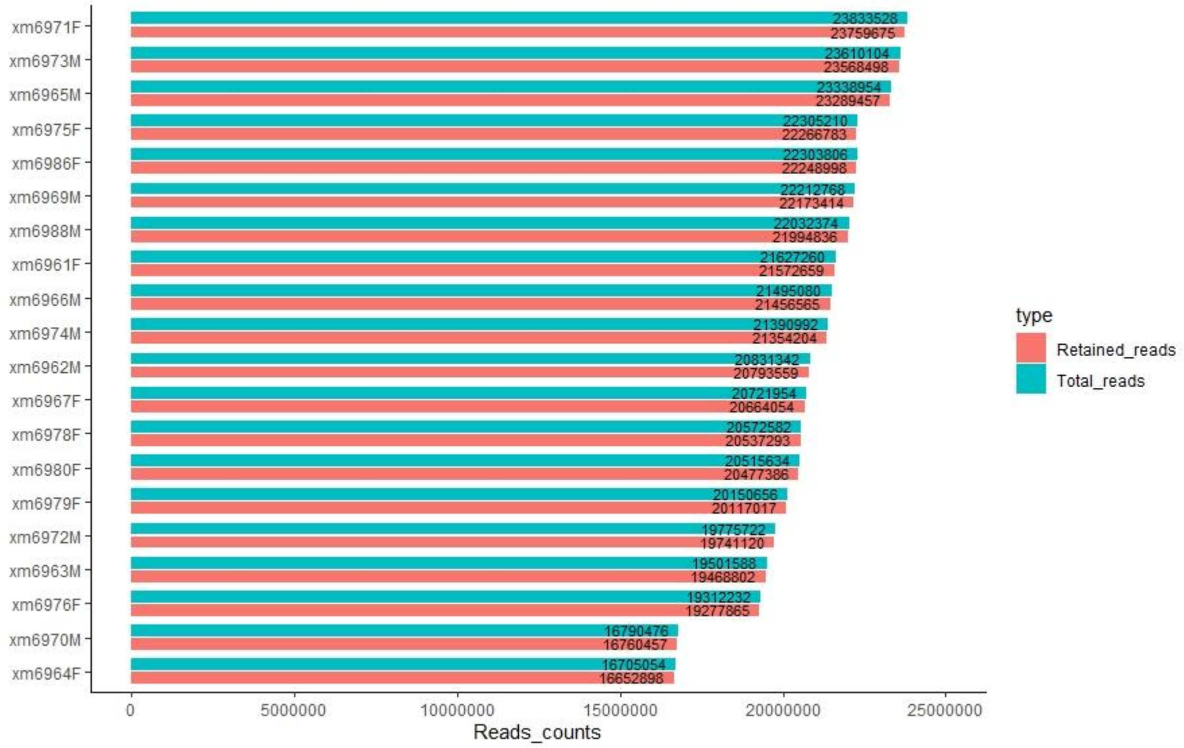

A. sp. (GBS data)

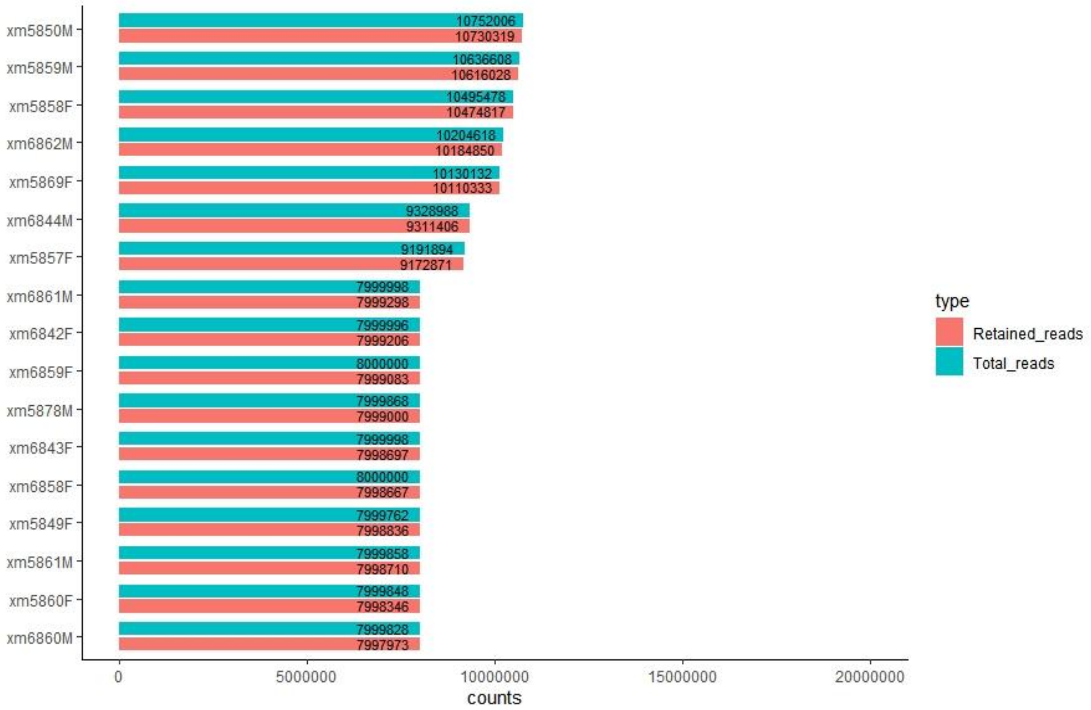

A. sp. (RAD-seq data)

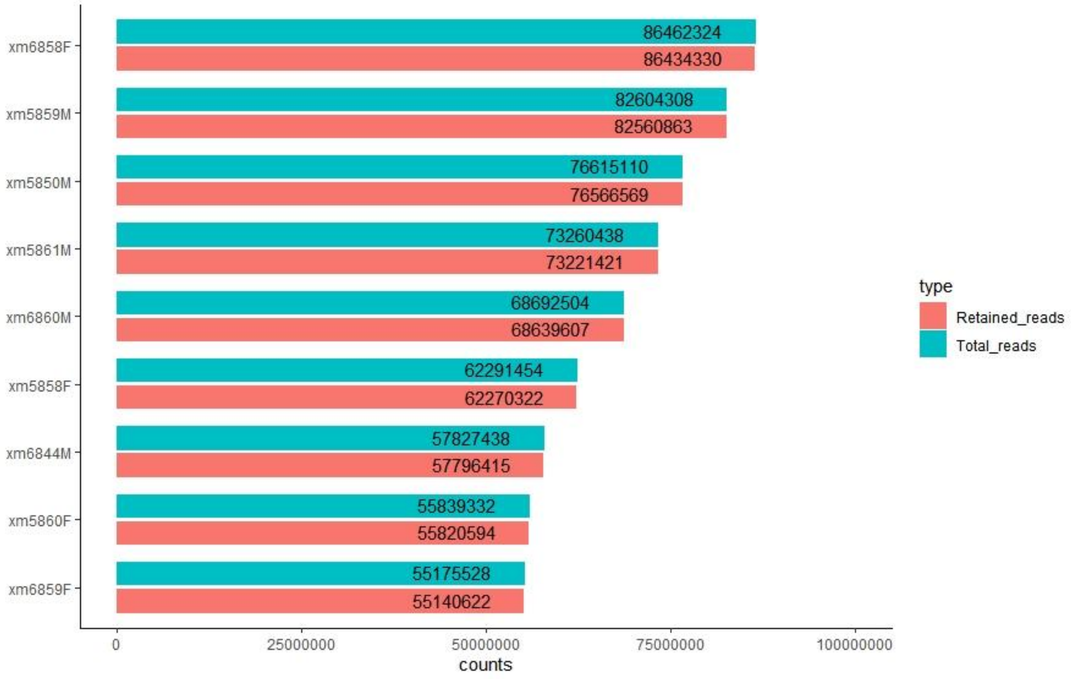

A. tuberodepressus (GBS data)

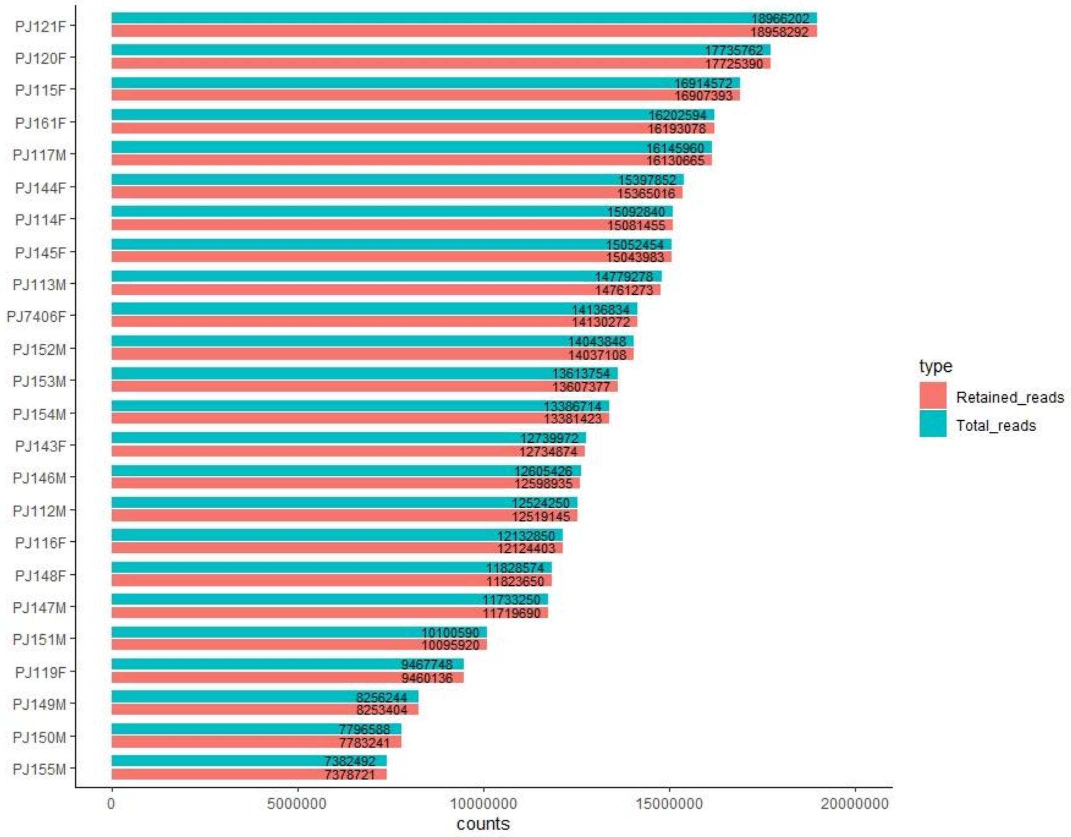

A. wuyiensis (GBS data)

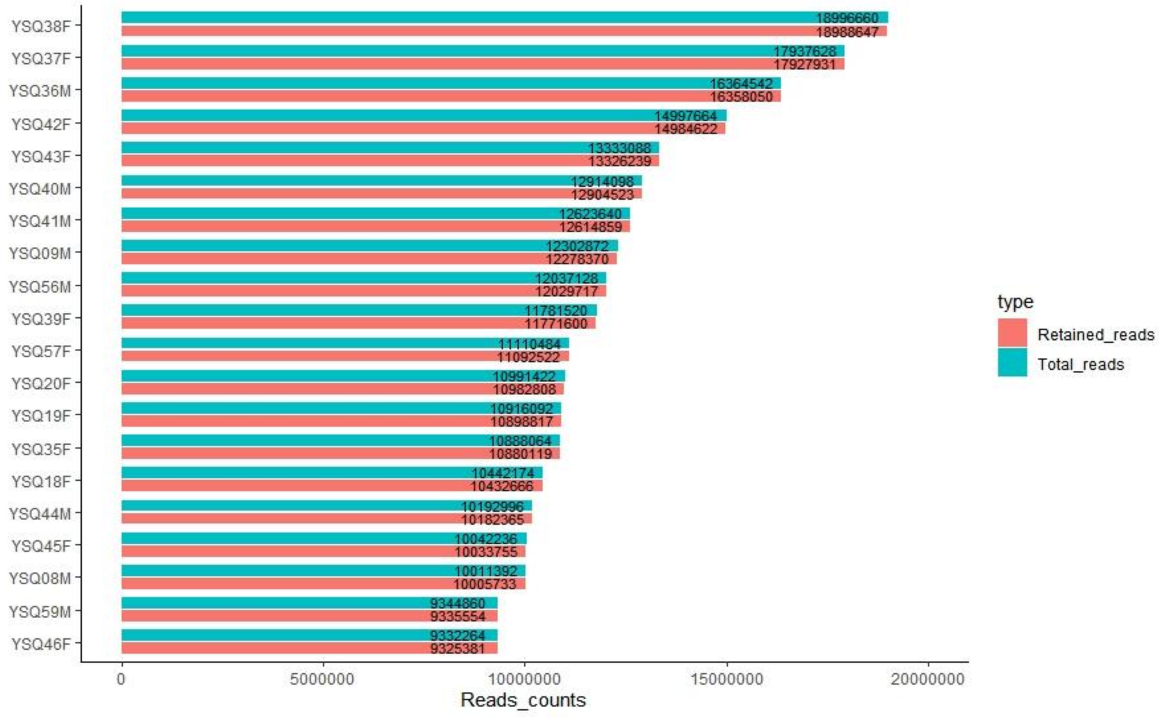

***A. xinduqiao* (GBS data)**

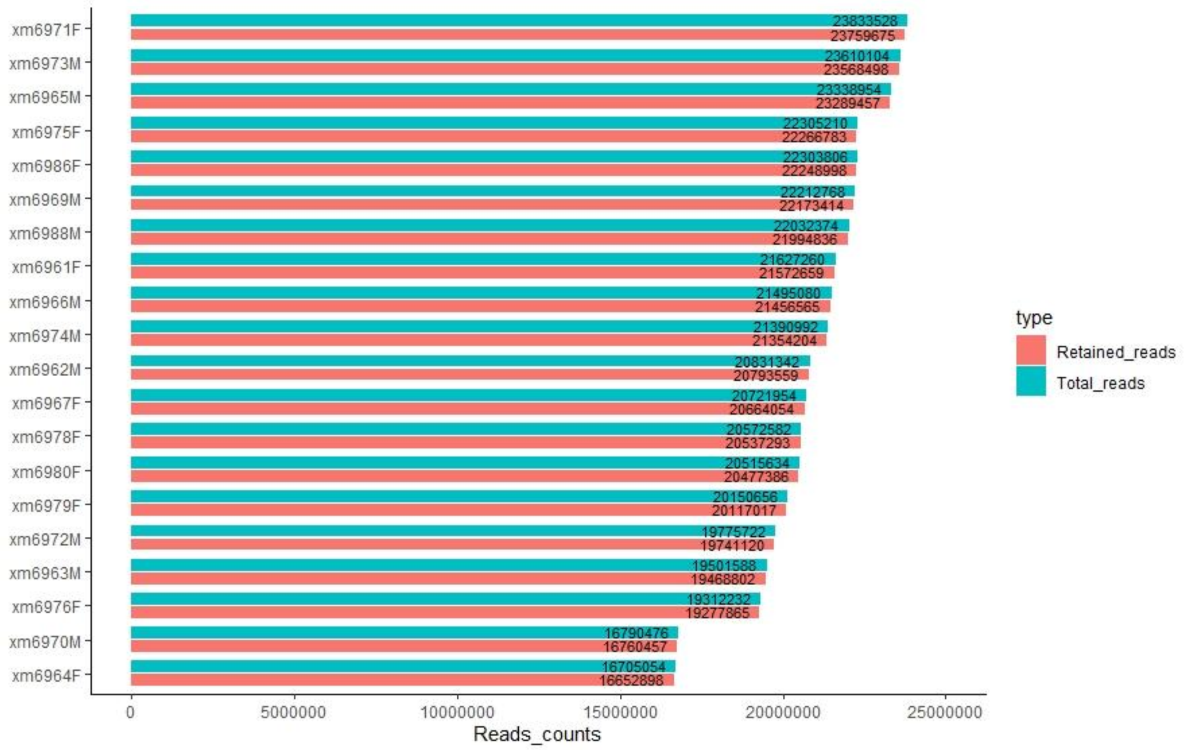

Supplement: Supplementary file 1 [file ijms-23-11146-s001.zip › ijms-1904191-manuscript-supplementary-revised/manuscript-supplementary/File S2.pdf]
