# Supplementary material for: Heterogeneous Evolution of Sex Chromosomes in the Torrent Frog Genus Amolops
Source: Int J Mol Sci. 2022 Sep 22;23(19):11146. doi: 10.3390/ijms231911146 (PMC9570394; doi:10.3390/ijms231911146)

File S3

Parameter optimization of Stacks *de novo* assembly analysis of *Amolops*. In both maps, we tried to increase M=n from 1 to 9, ensuring M=3, and to monitor the number of polymorphic loci found in 80% (r80 locus) or more of the sample, until we find the most stable set of values for M, n, and m (M controls the number of mismatches allowed between the two alleles of a heterozygote sample; n controls the number of mismatches allowed between any two alleles of the population; m controls the number of identical reads required to initiate a new putative allele). **a.** Number of loci shared by ≥80% of samples. **b.** Number and distribution of snps per locus.

A. Chunganensis **(Optimal parameter： M=n=3, m=3.)**

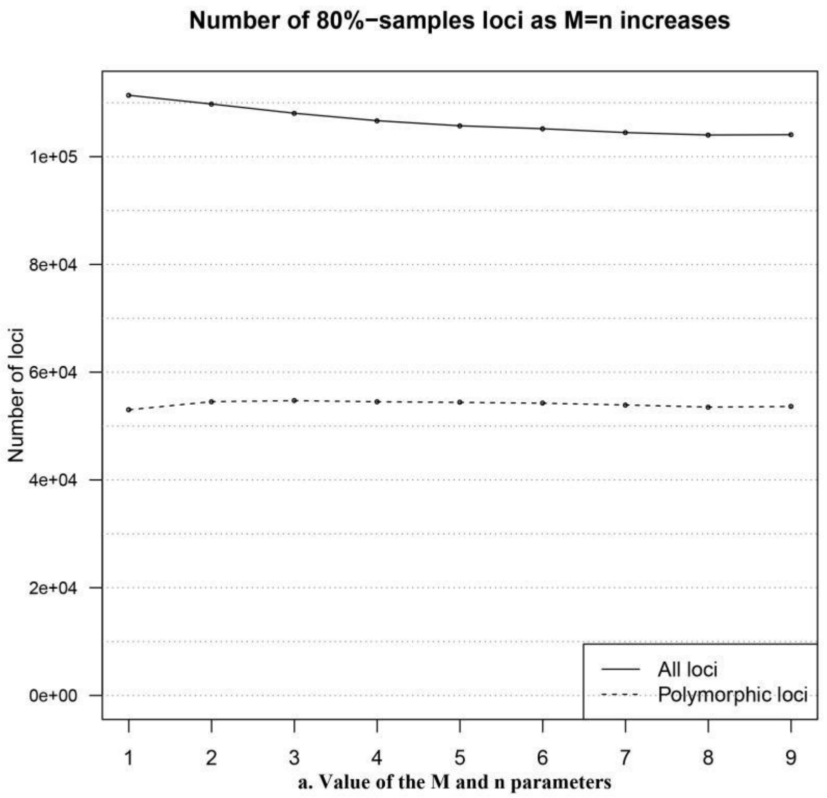

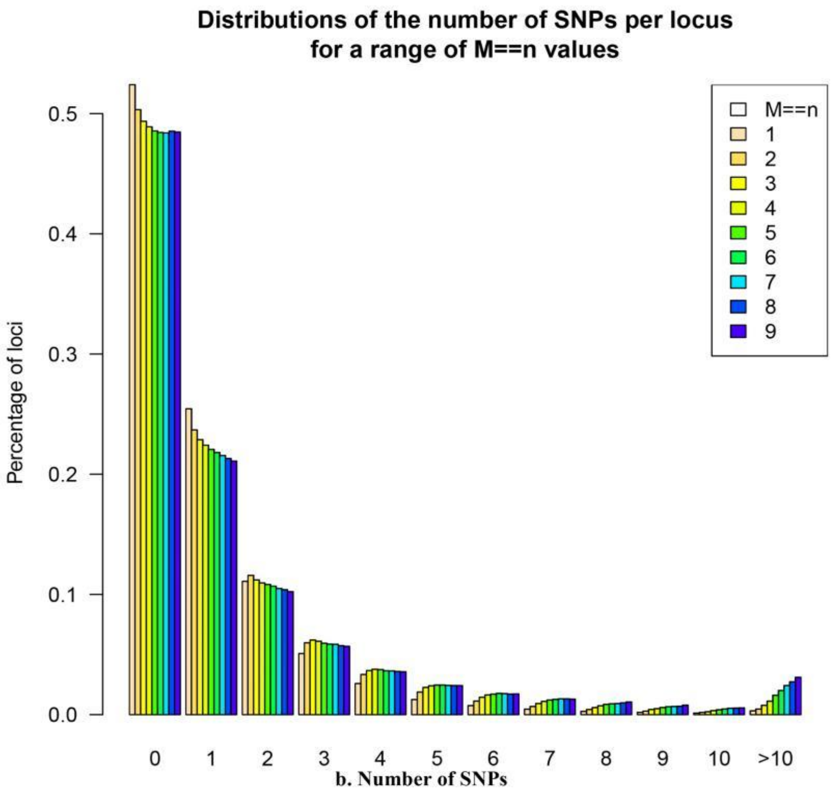

A. jinjiangensis **(Optimal parameter： M=n=2, m=3.)**

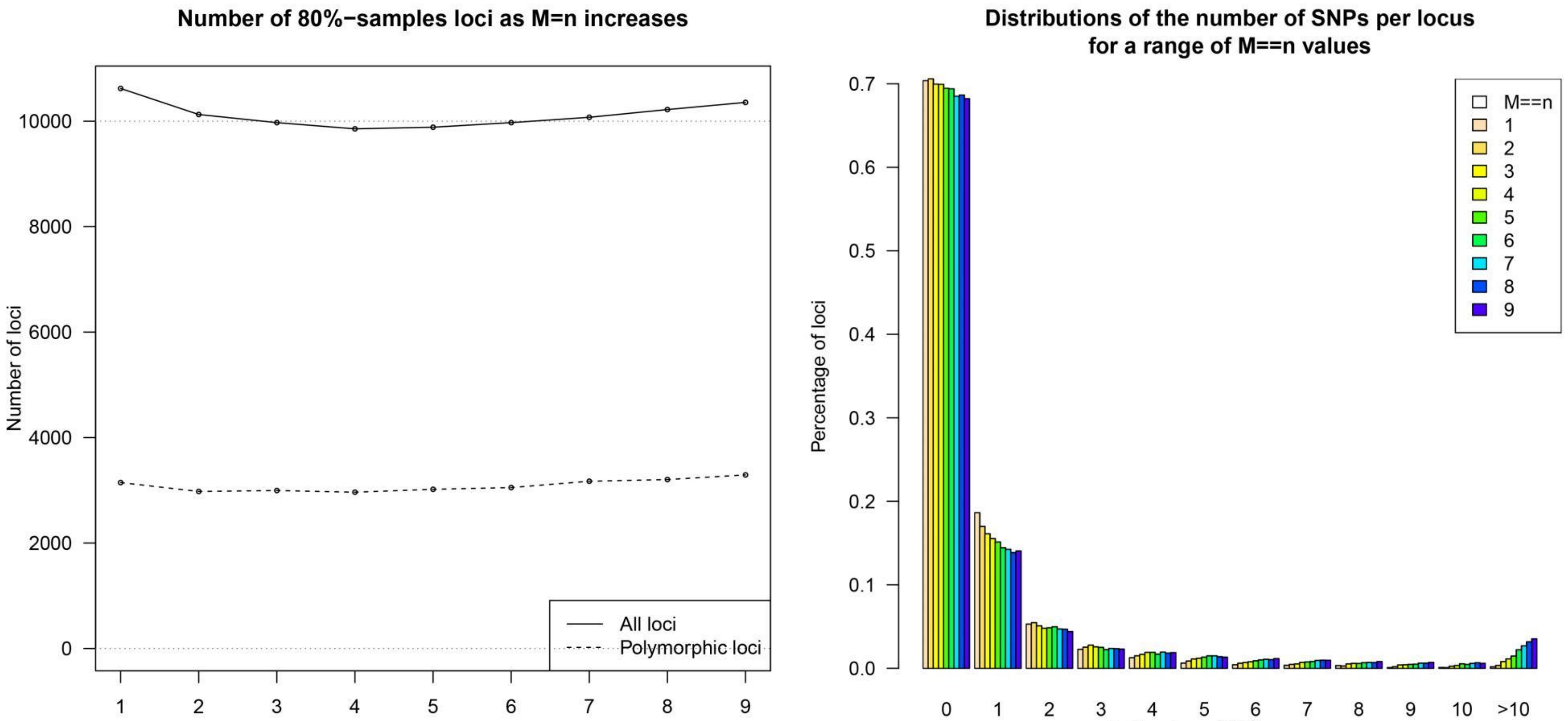

a. Value of the M and n parameters

b. Number of SNPs

***A. lifanensis* (Optimal parameter：M=n=2, m=3.)**

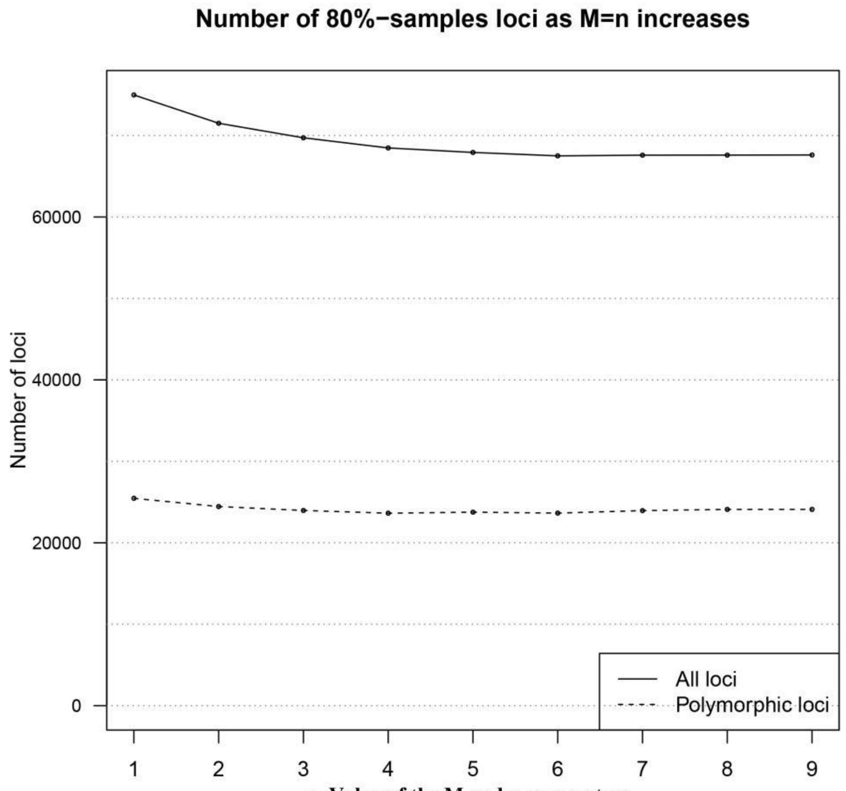

a. Value of the M and n parameters

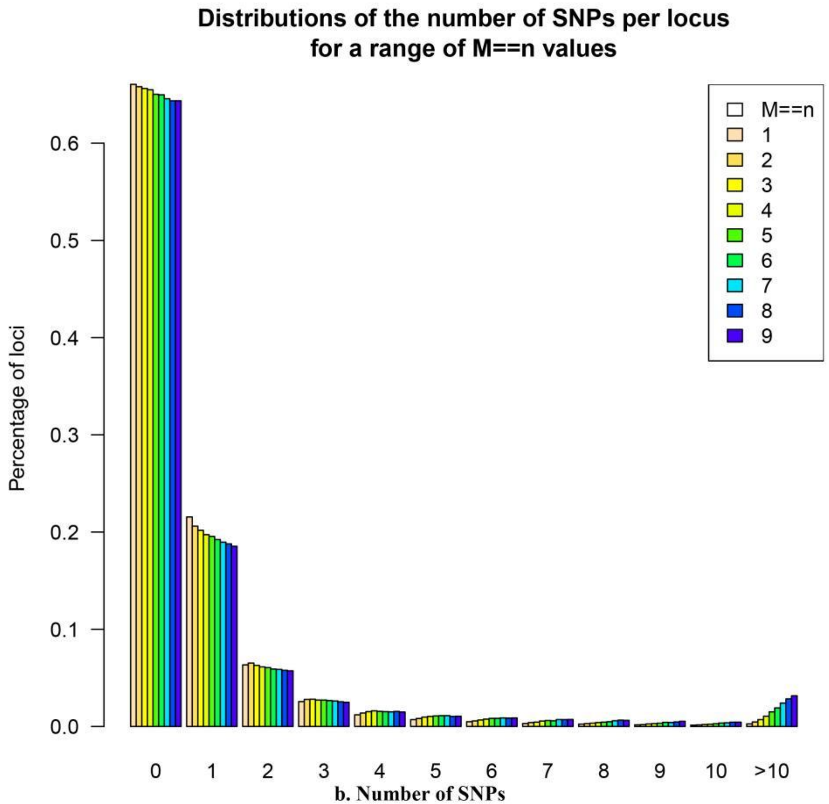

b. Number of SNPs

A. loloensis **(Optimal parameter：M=n=3, m=3.)**

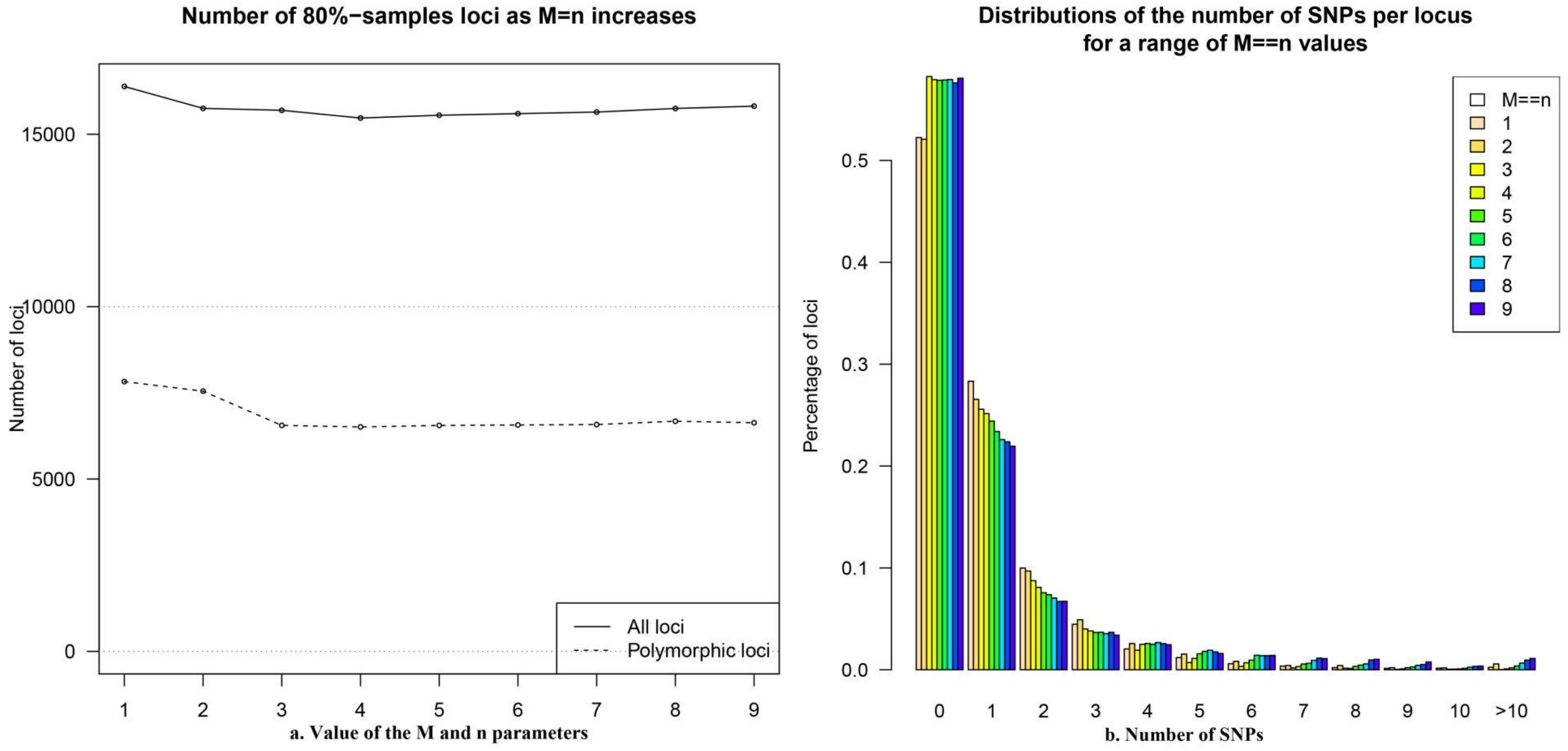

A. mantzorum **(Optimal parameter：M=n=3, m=3.)**

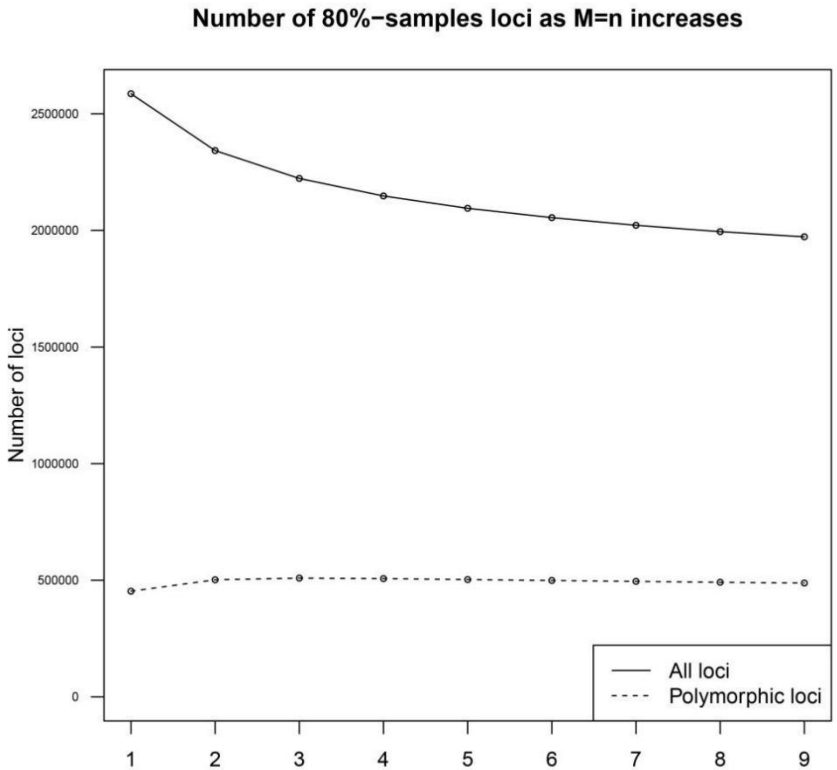

a. Value of the M and n parameters

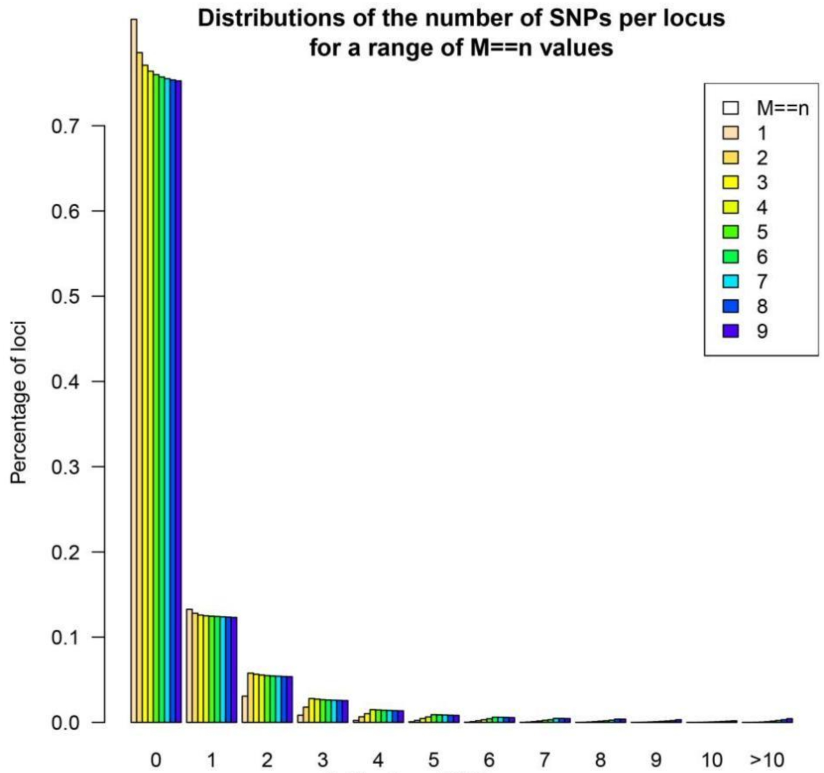

b. Number of SNPs

***A.* sp. (Optimal parameter：M=n=2, m=3.)**

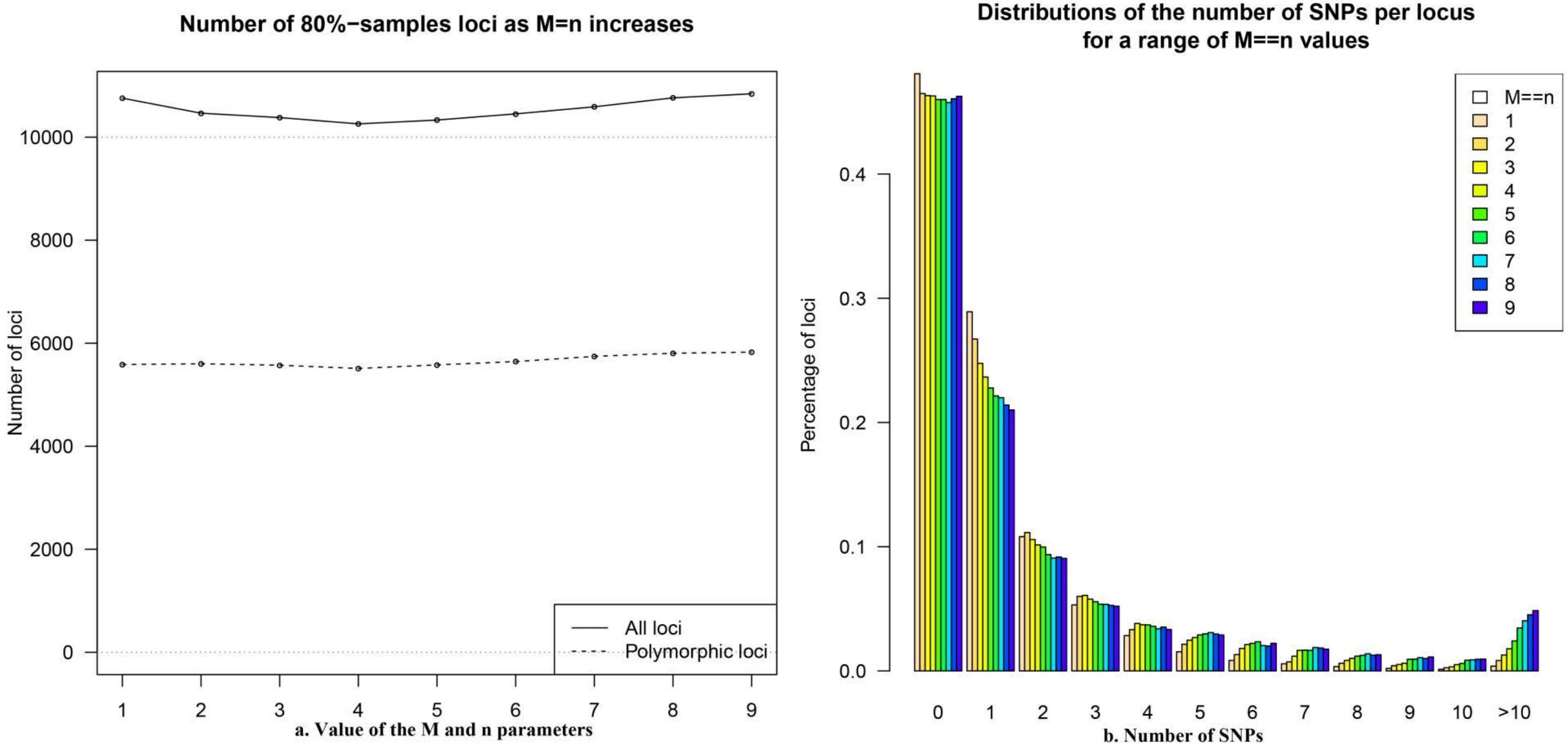

***A. tuberodepressus* (Optimal parameter：M=n=2, m=3.)**

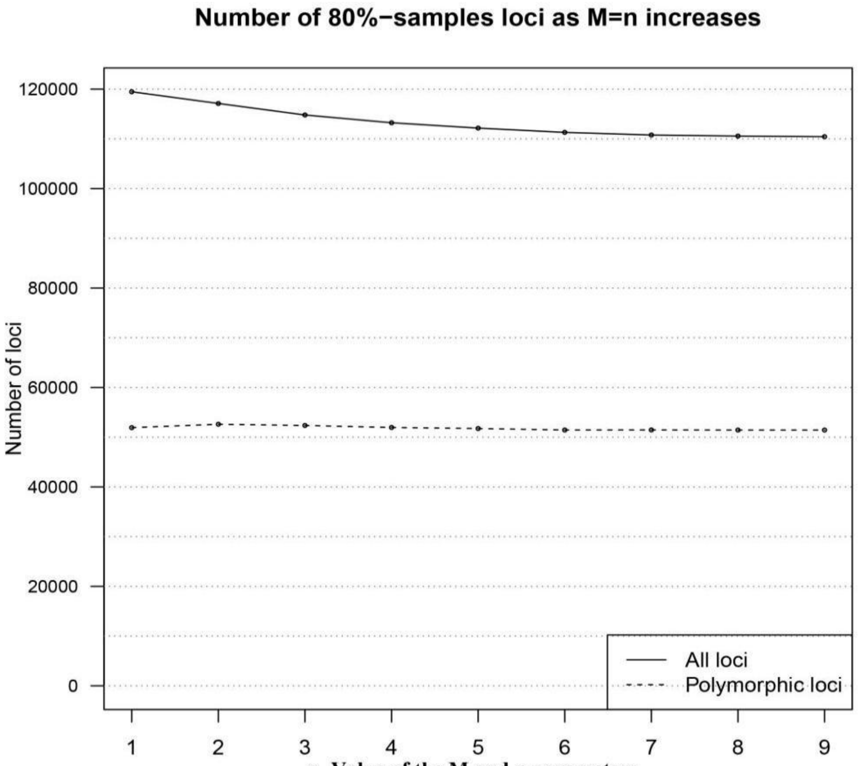

a. Value of the M and n parameters

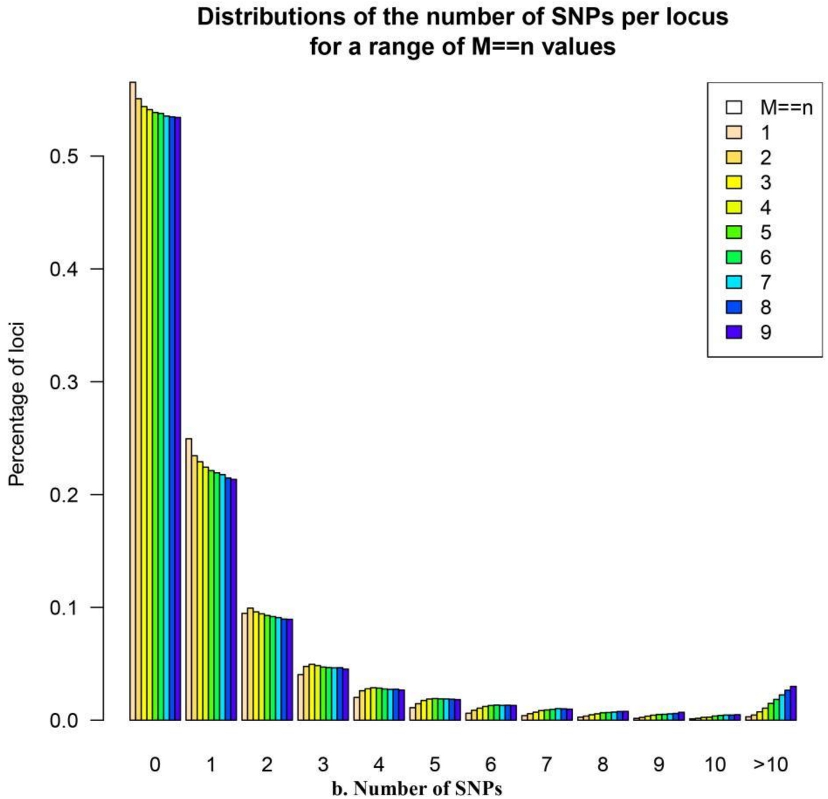

b. Number of SNPs

A. wuyiensis **(Optimal parameter： M=n=5, m=3.)**

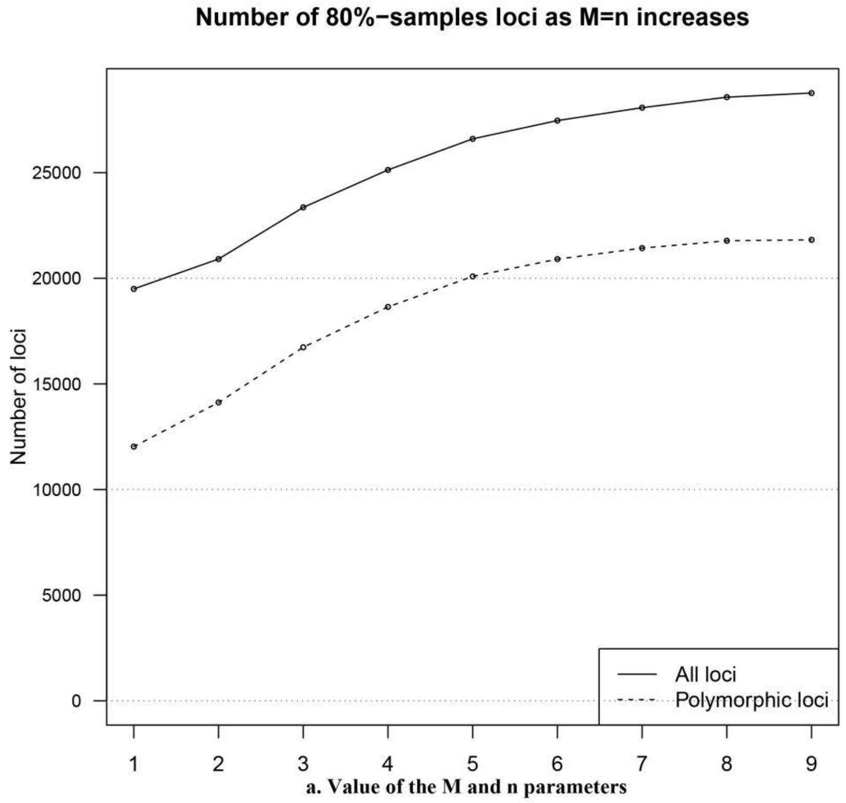

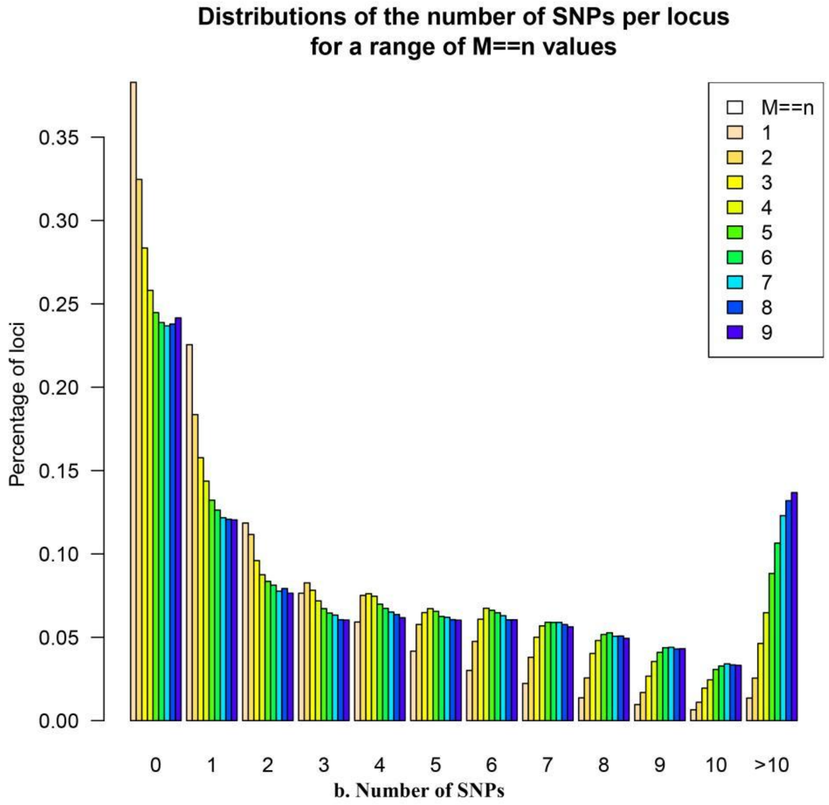

A. xinduqiao **(Optimal parameter： M=n=3, m=3.)**

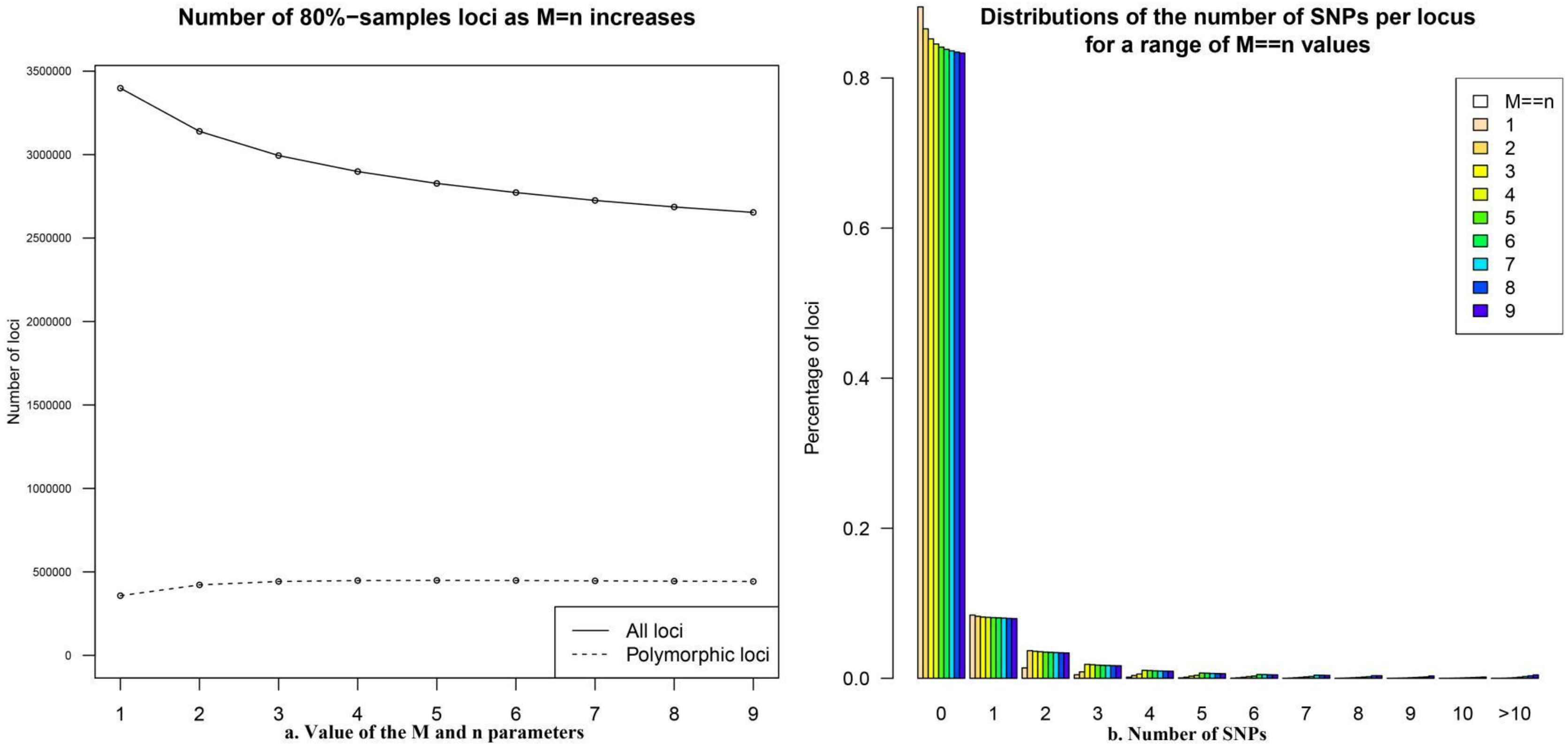

Supplement: Supplementary file 1 [file ijms-23-11146-s001.zip › ijms-1904191-manuscript-supplementary-revised/manuscript-supplementary/File S3.pdf]
