# Supplementary material for: Heterogeneous Evolution of Sex Chromosomes in the Torrent Frog Genus Amolops
Source: Int J Mol Sci. 2022 Sep 22;23(19):11146. doi: 10.3390/ijms231911146 (PMC9570394; doi:10.3390/ijms231911146)

File S4

PCR validation partial results of sex linkage sites for *Amolops* (*A. Chunganensis*, *A. jinjiangensis*, *A. lifanensis*, *A. loloensis*, *A. mantzorum*, *A.* sp., *A. tuberodepressus*, *A. wuyiensis*, *A. xinduqiao*). The variable loci of males were shown in blue boxes, and the variable loci of females were shown in black boxes. Female individuals were indicated by ♀ and male individuals used ♂. The polymorphic sites of locus show heterozygosity and homozygosity in all male individuals and female individuals, respectively.

A. Chunganensis **(Clocus_1058318)**

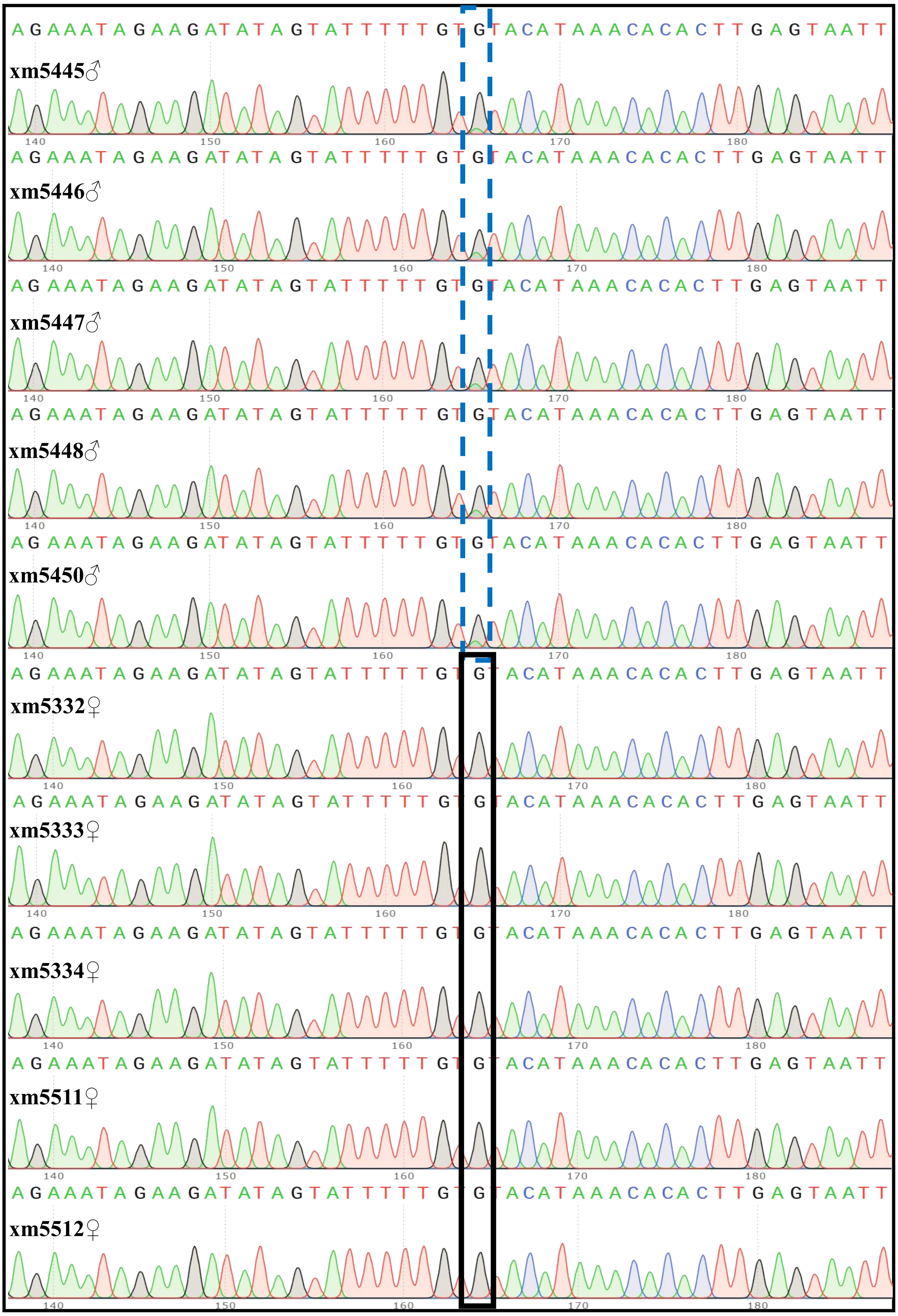

A. Chunganensis (Clocus_2909840)

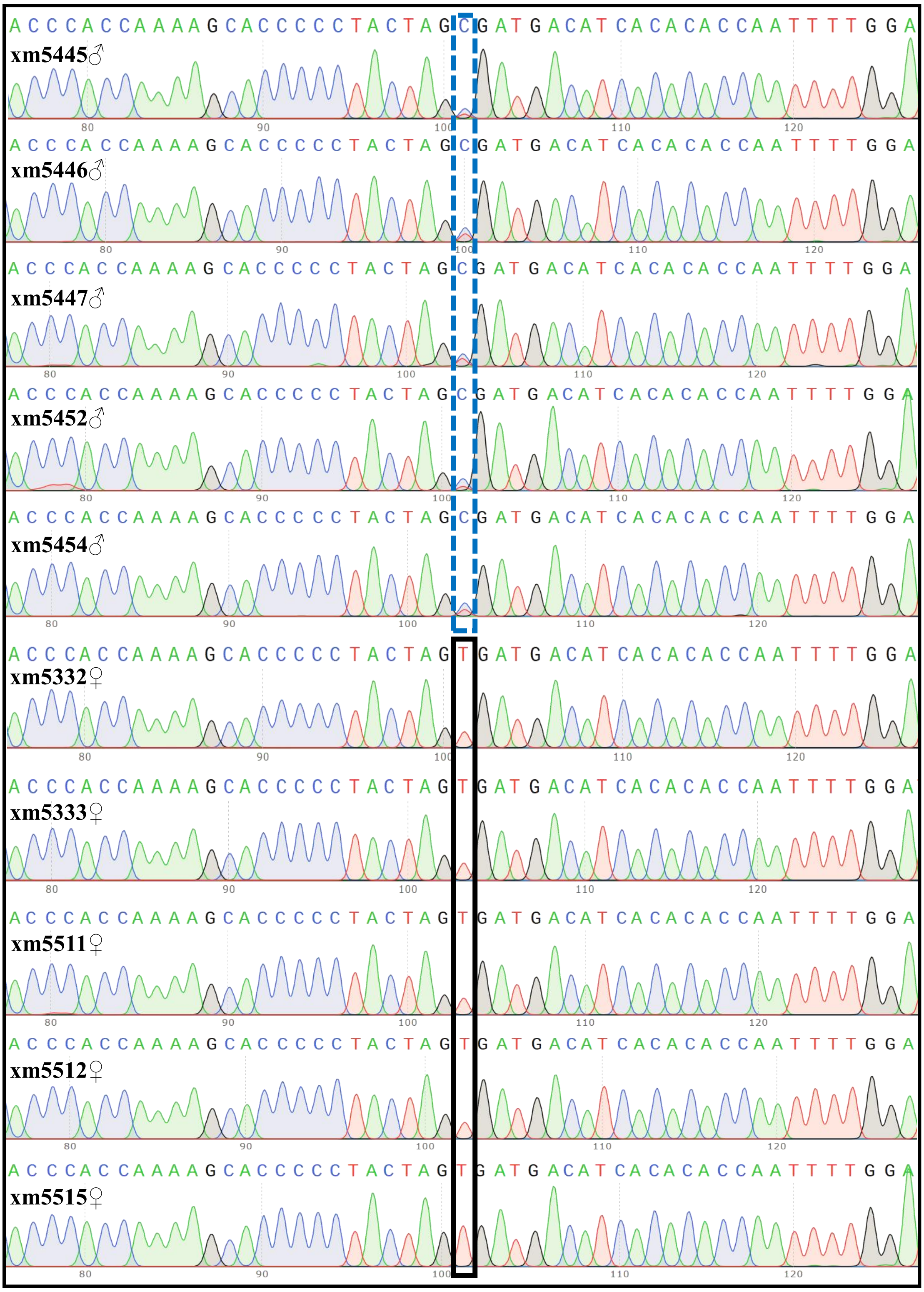

A. jinjiangensis (Clocus_483348)

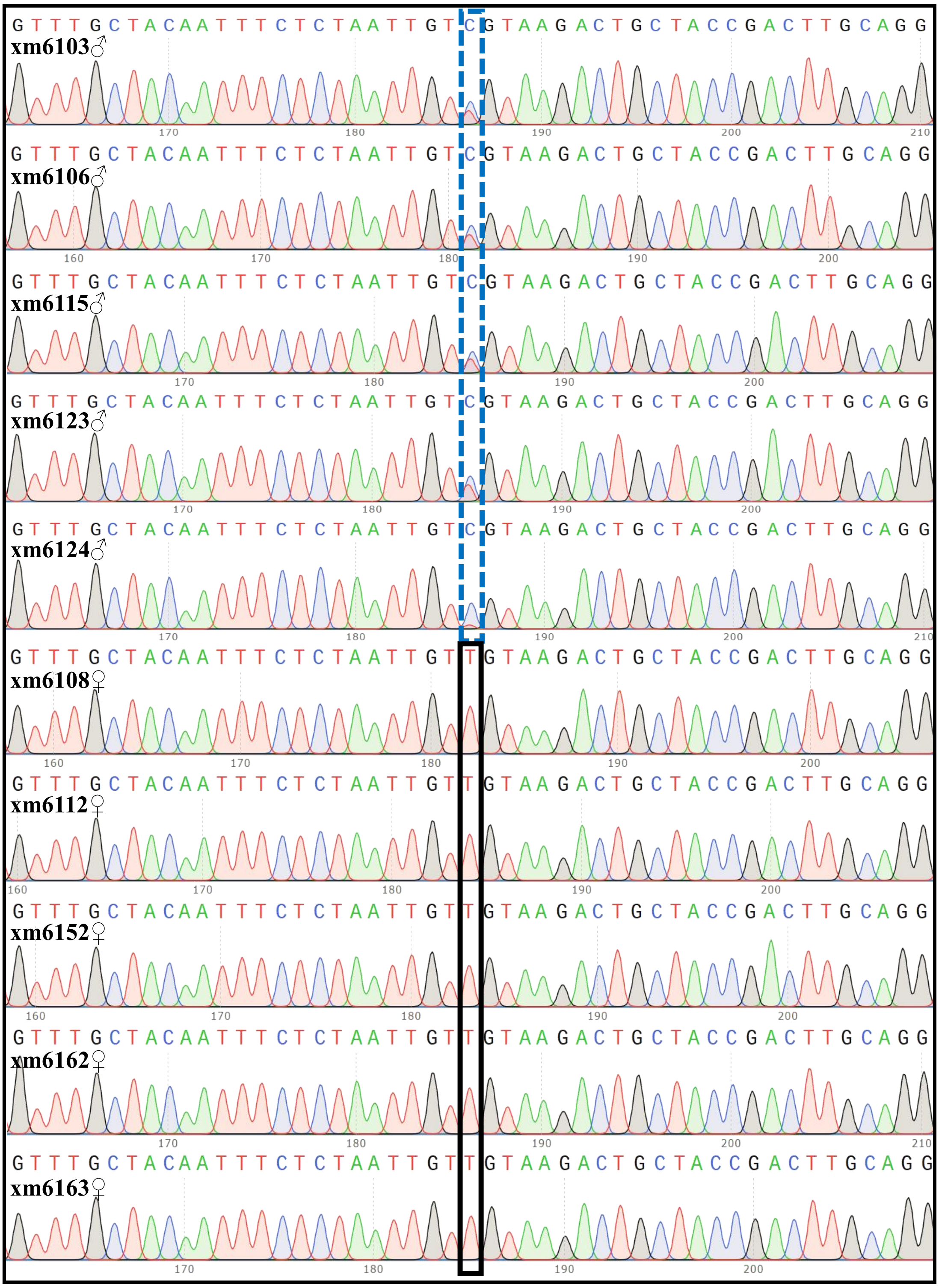

A. jinjiangensis (Clocus_1184383)

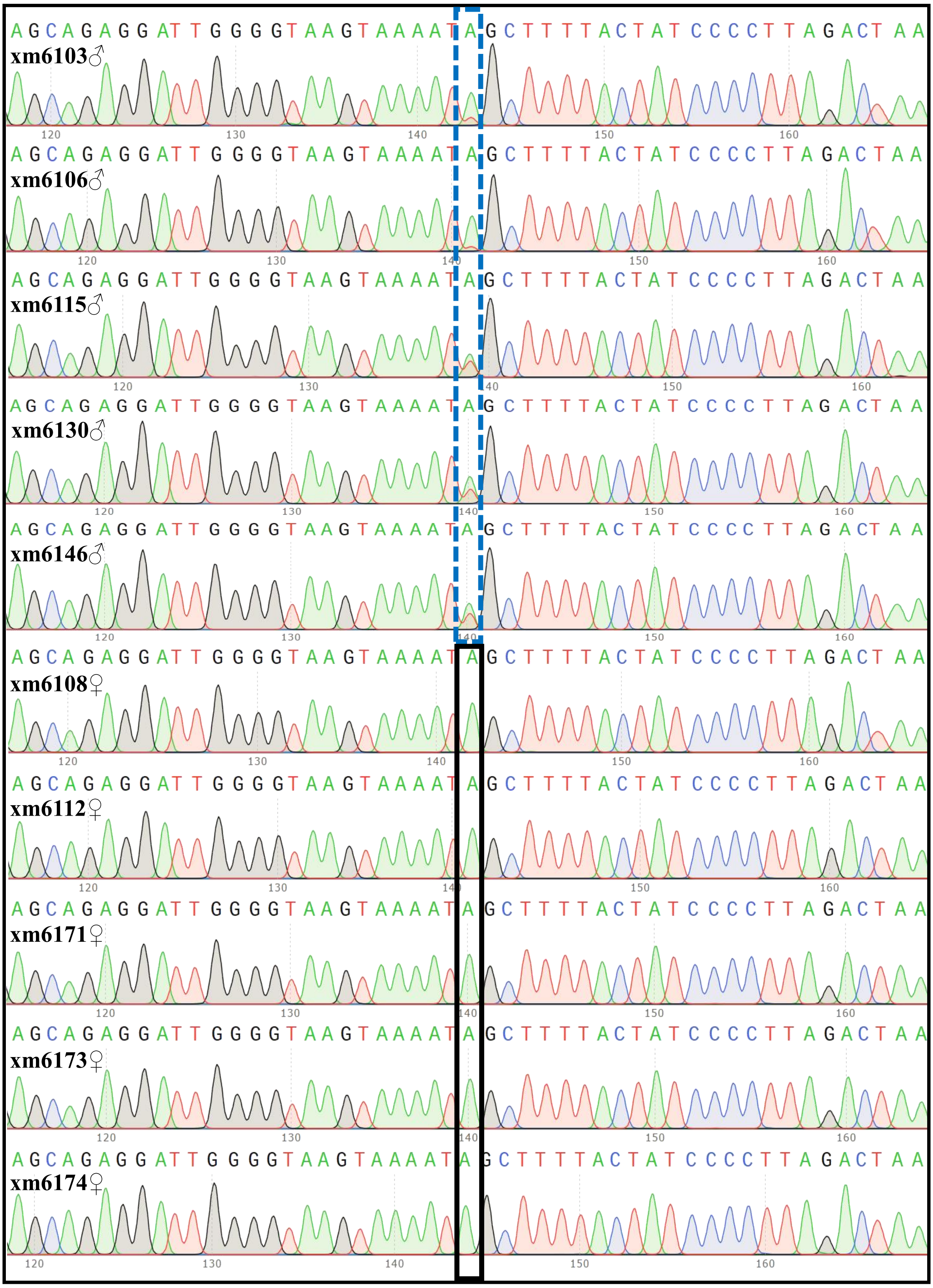

***A. lifanensis* (Clocus_174627)**

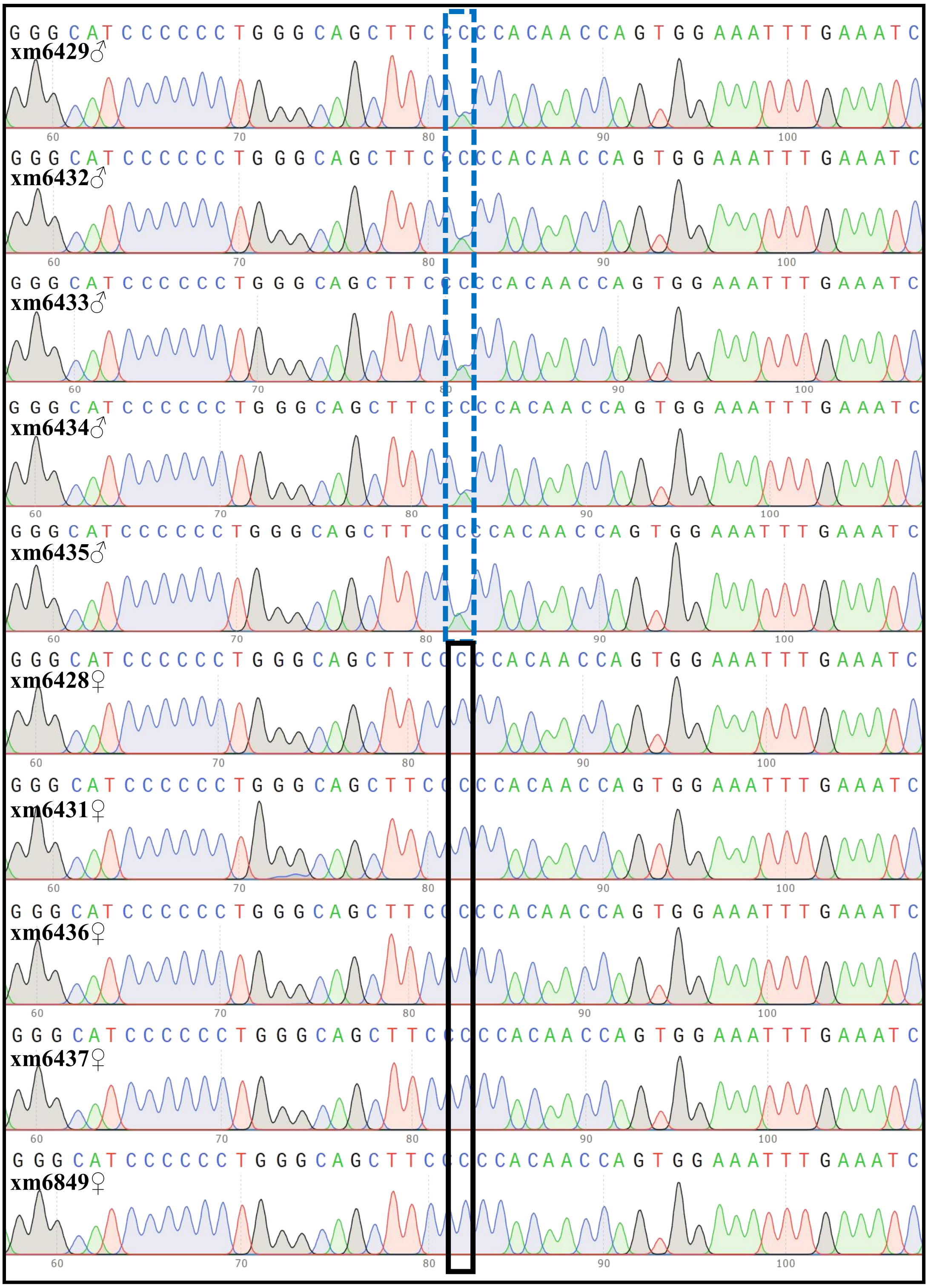

***A. lifanensis* (Clocus_375069)**

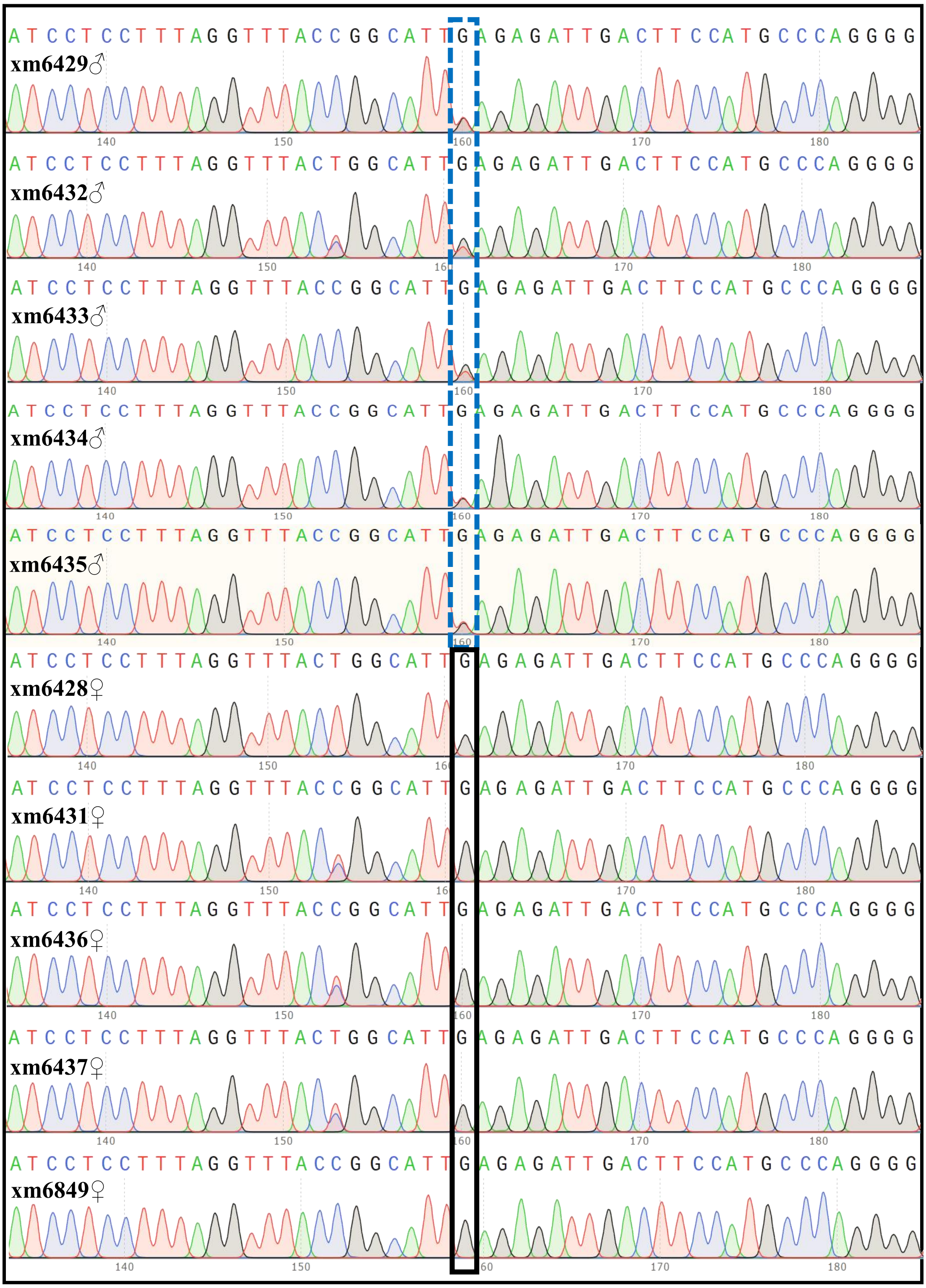

***A. lifanensis* (Clocus_430733)**

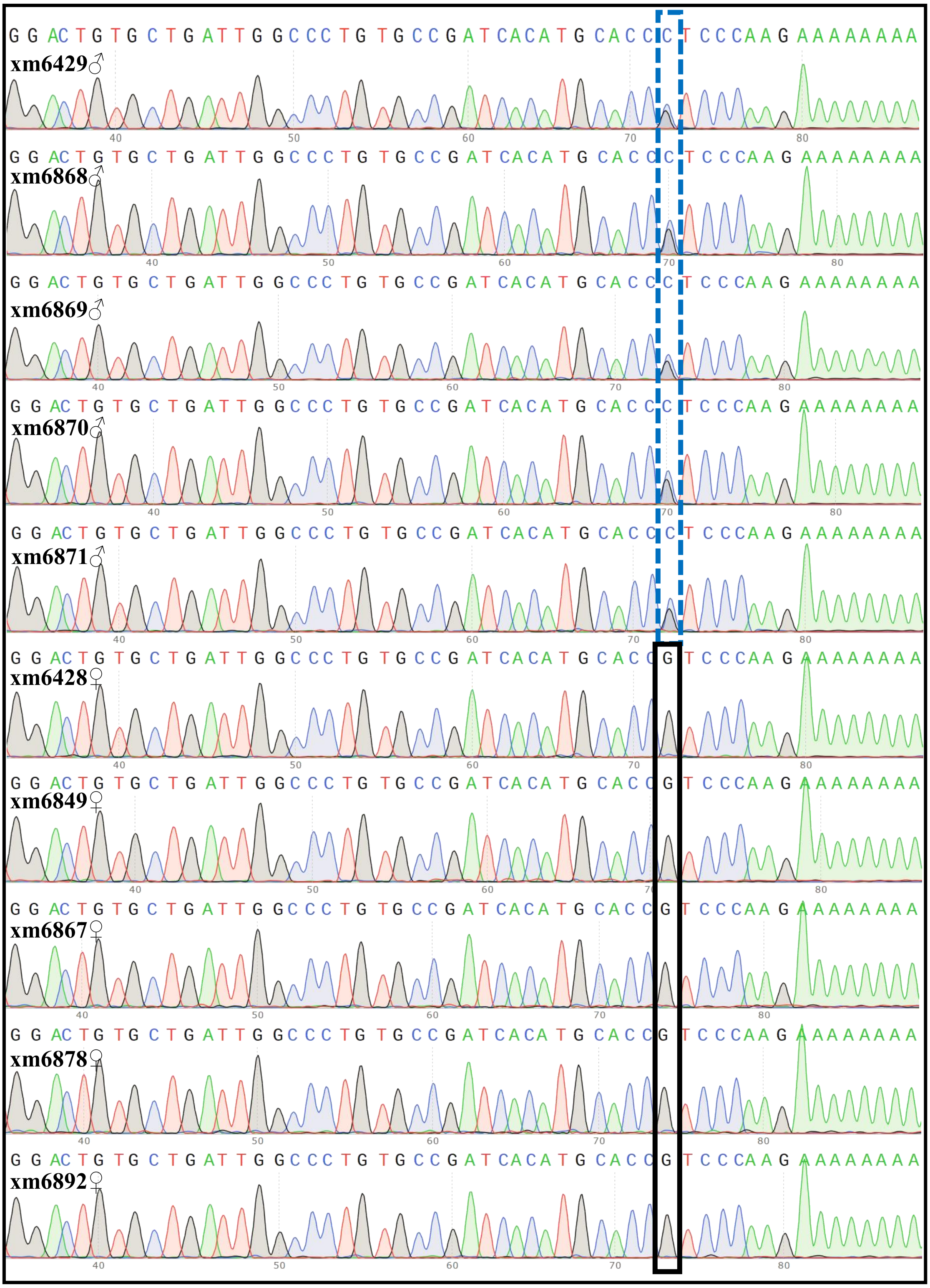

A. loloensis (Clocus_550969)

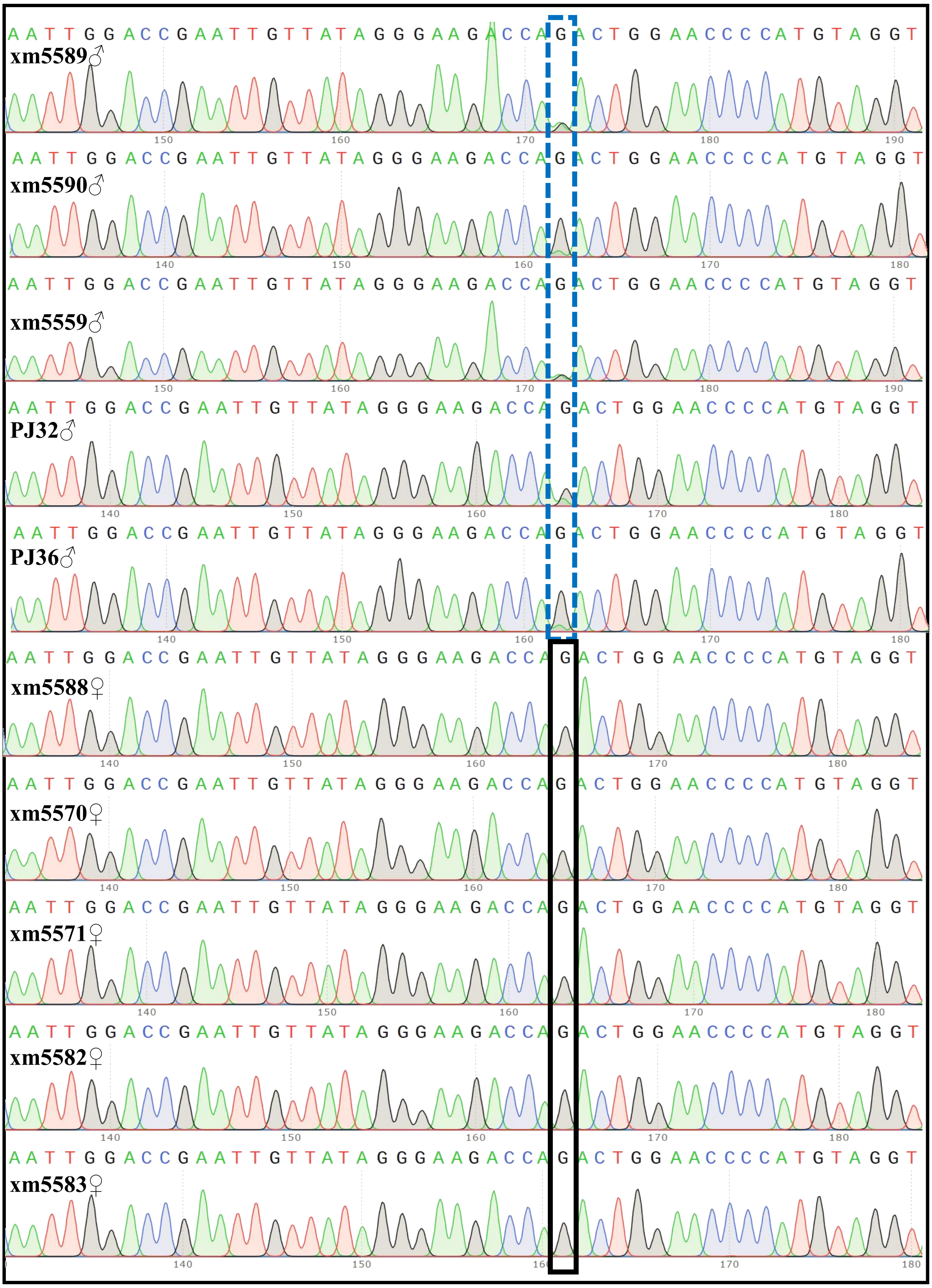

A. mantzorum (Clocus_85869)

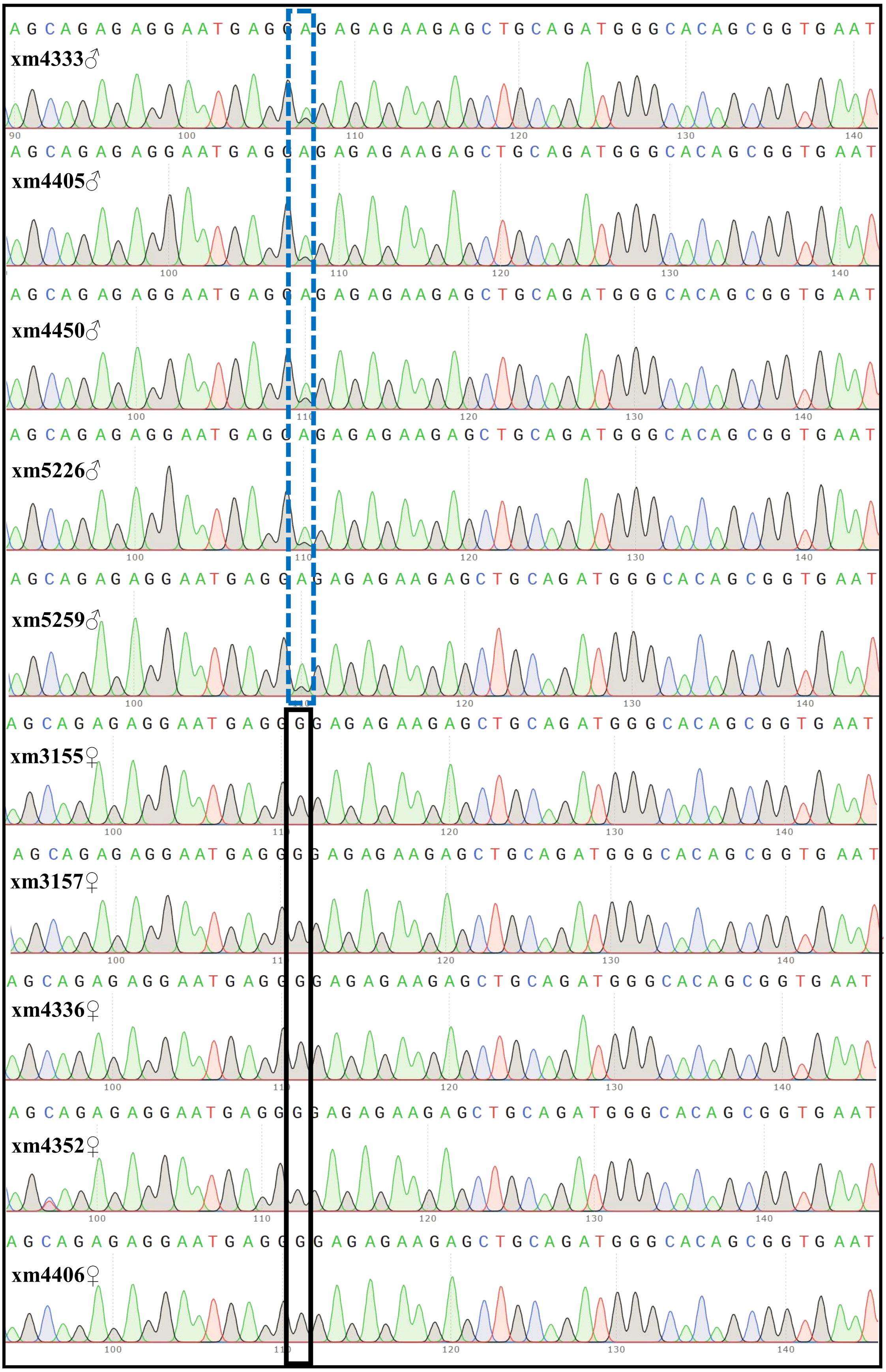

***A.* sp. (Clocus_110086)**

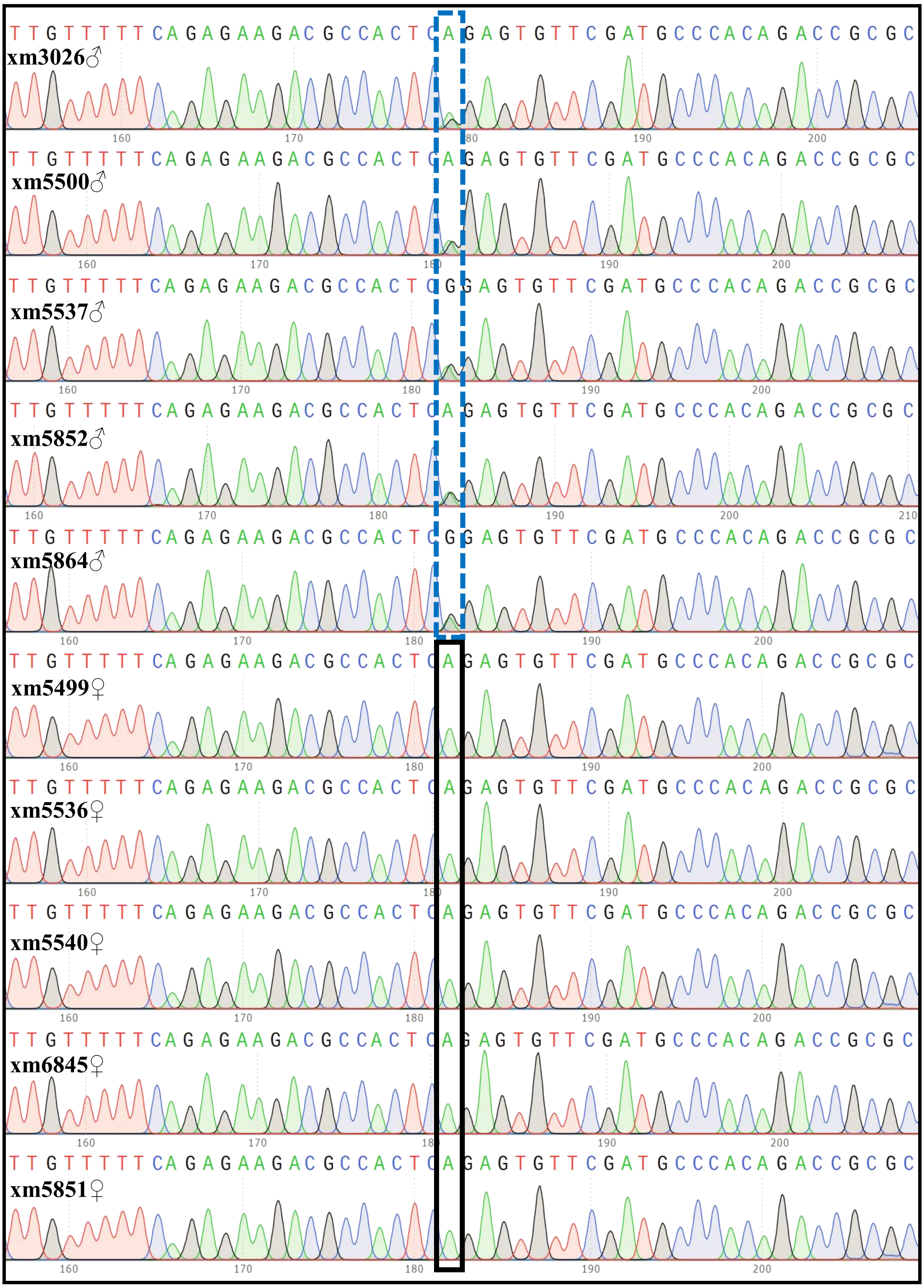

A. sp. (Clocus_208821)

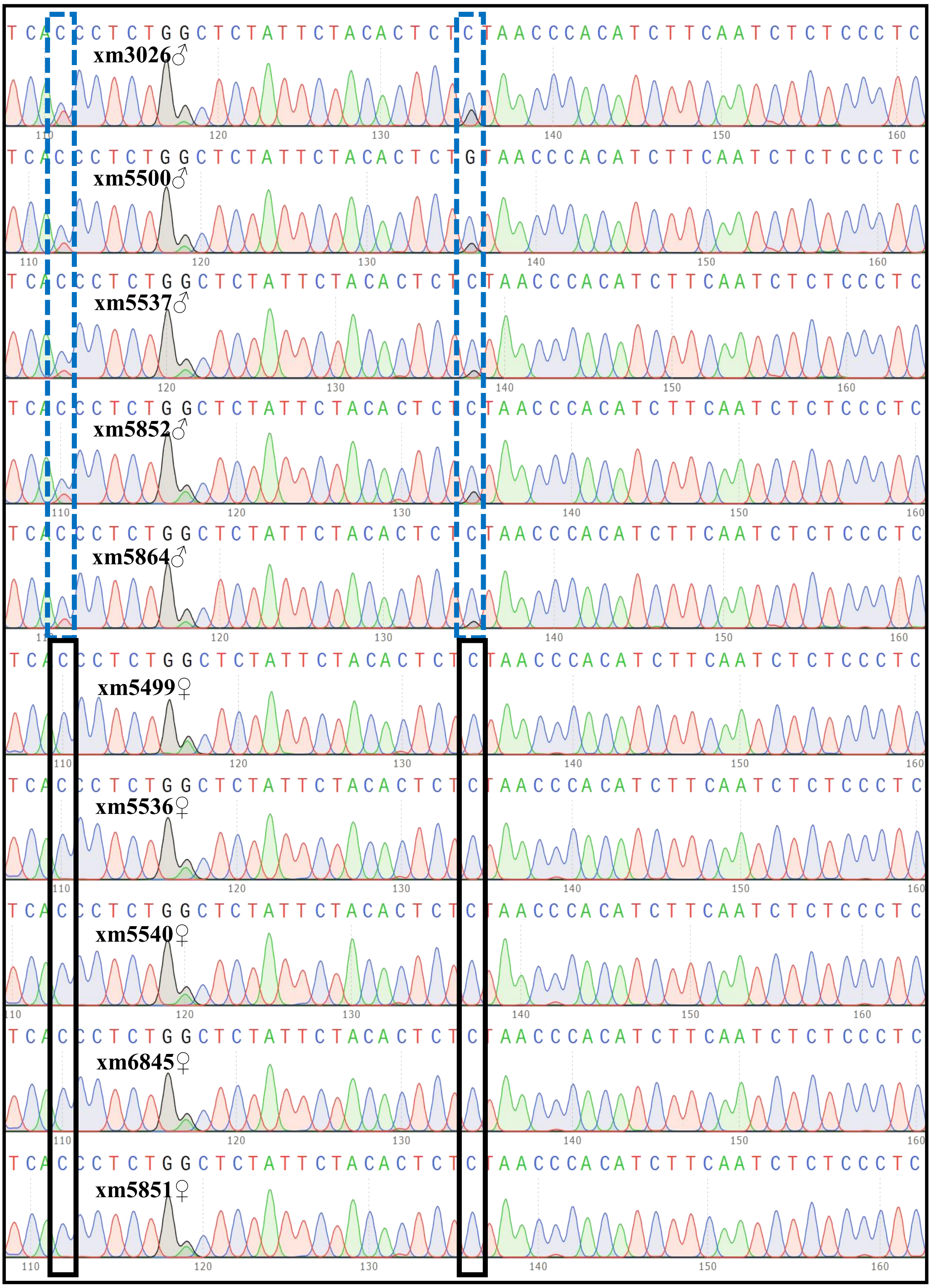

A. sp. (Clocus_271488)

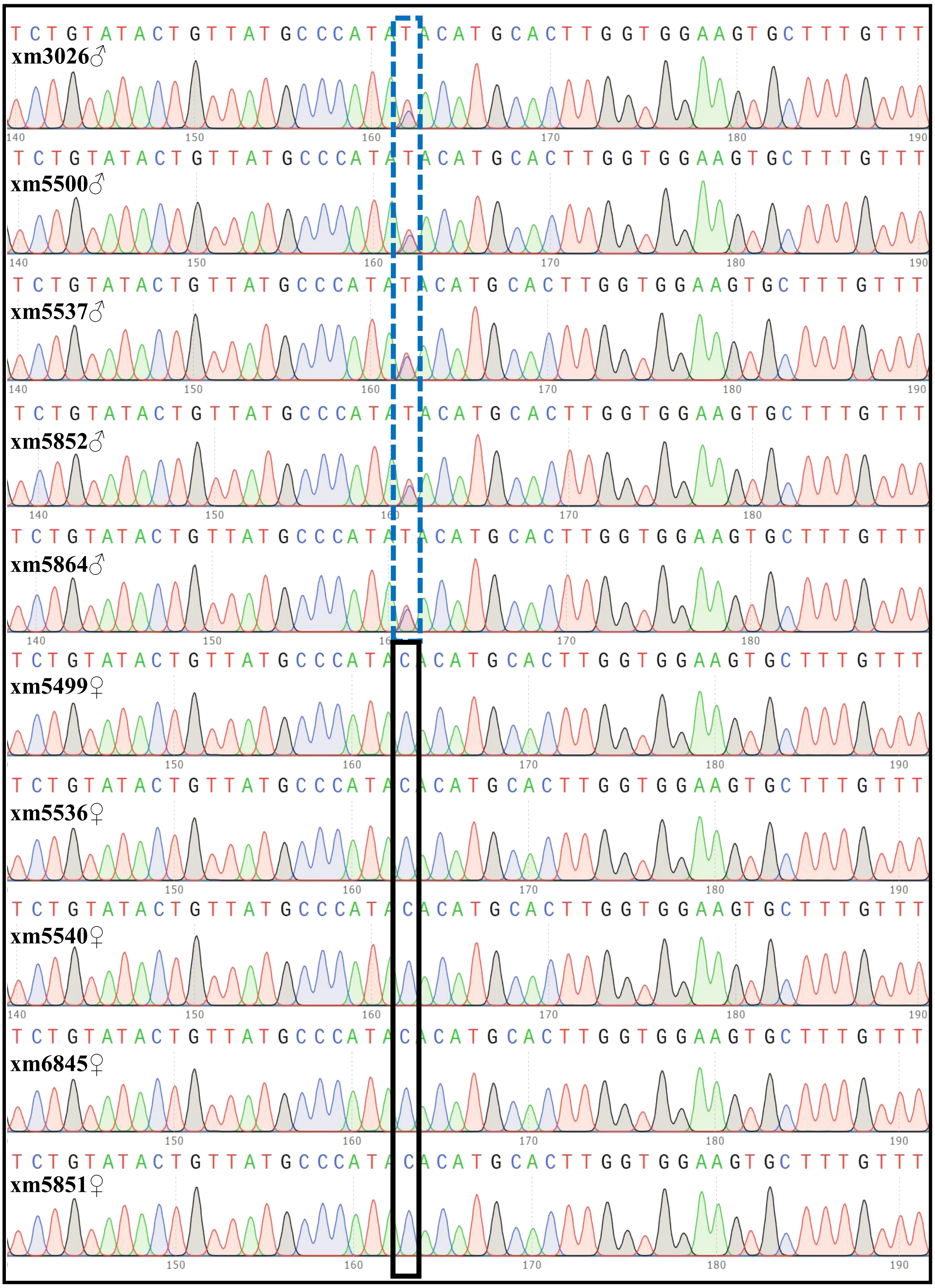

A. sp. (Clocus_476103)

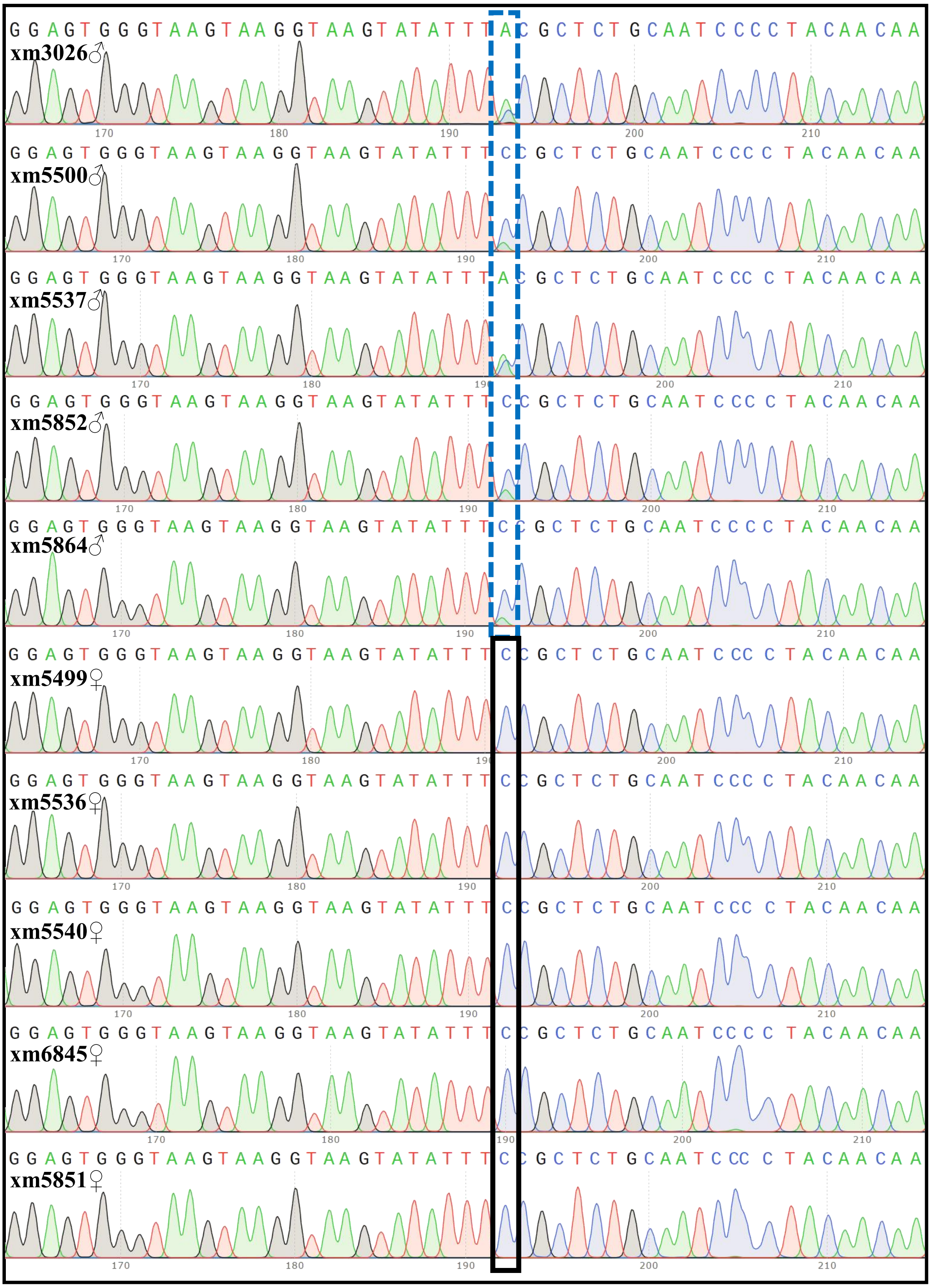

A. sp. (Clocus_1399857)

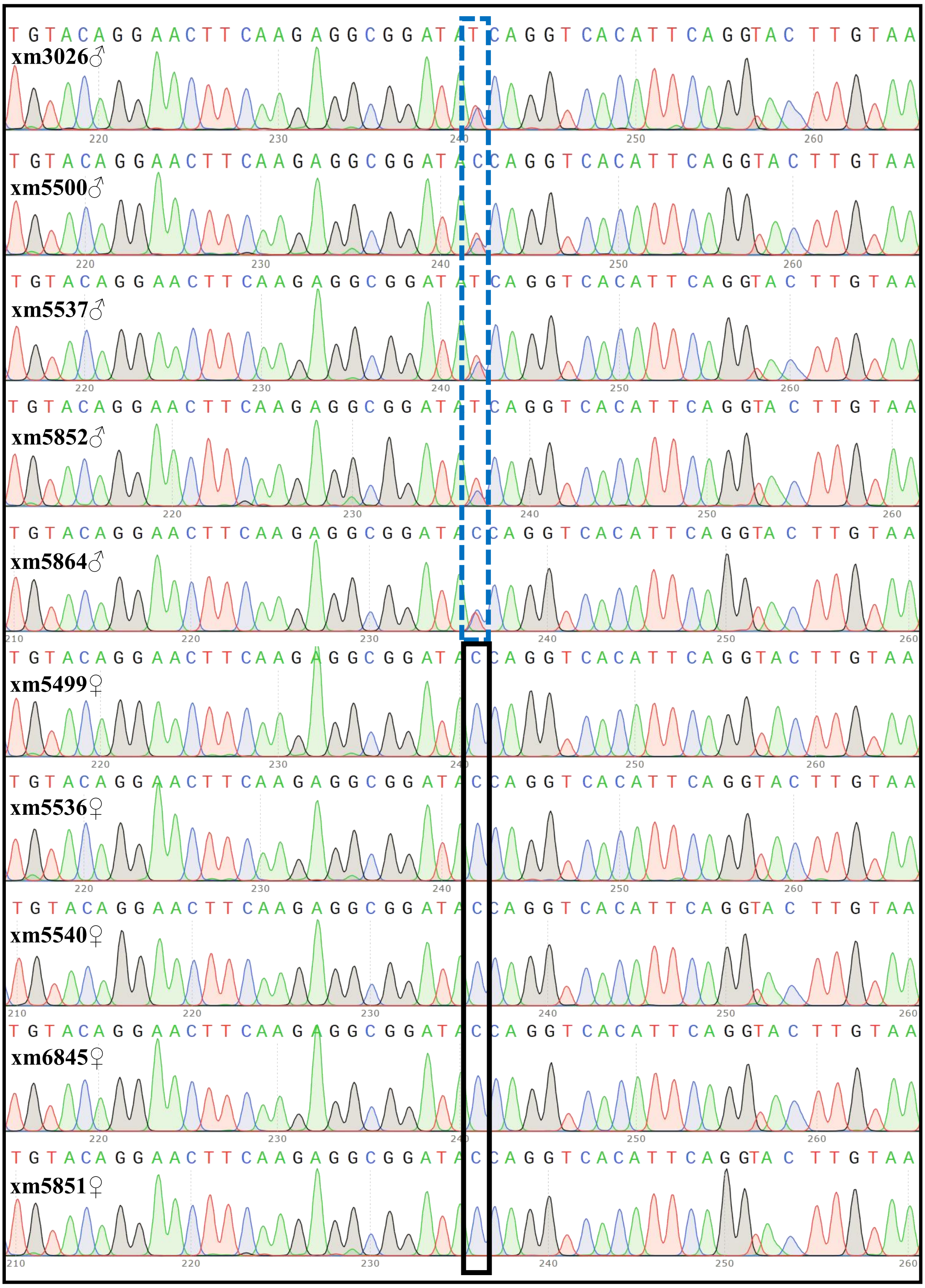

A. tuberodepressus (Clocus_1007365)

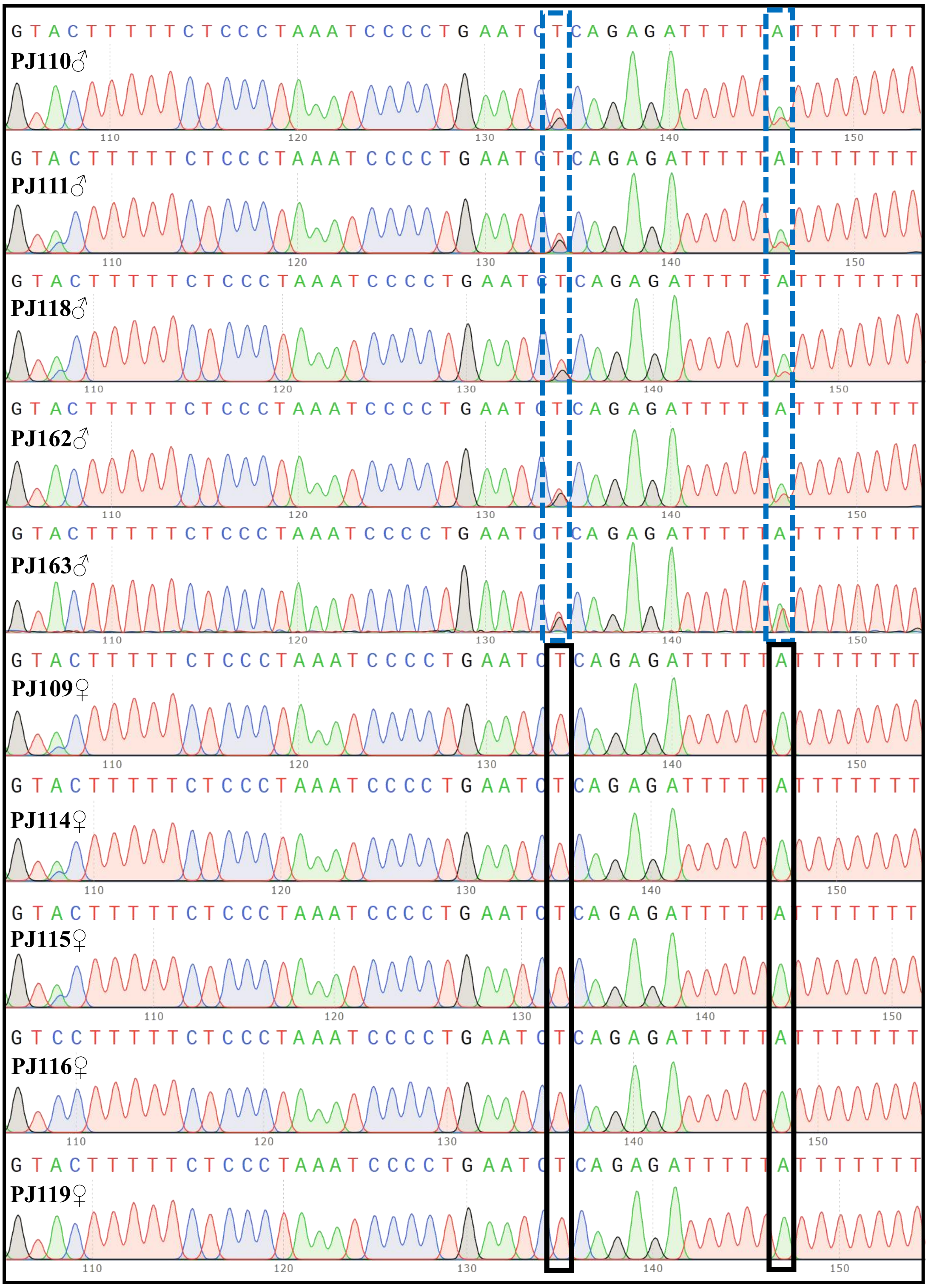

A. tuberodepressus (Clocus_1150683)

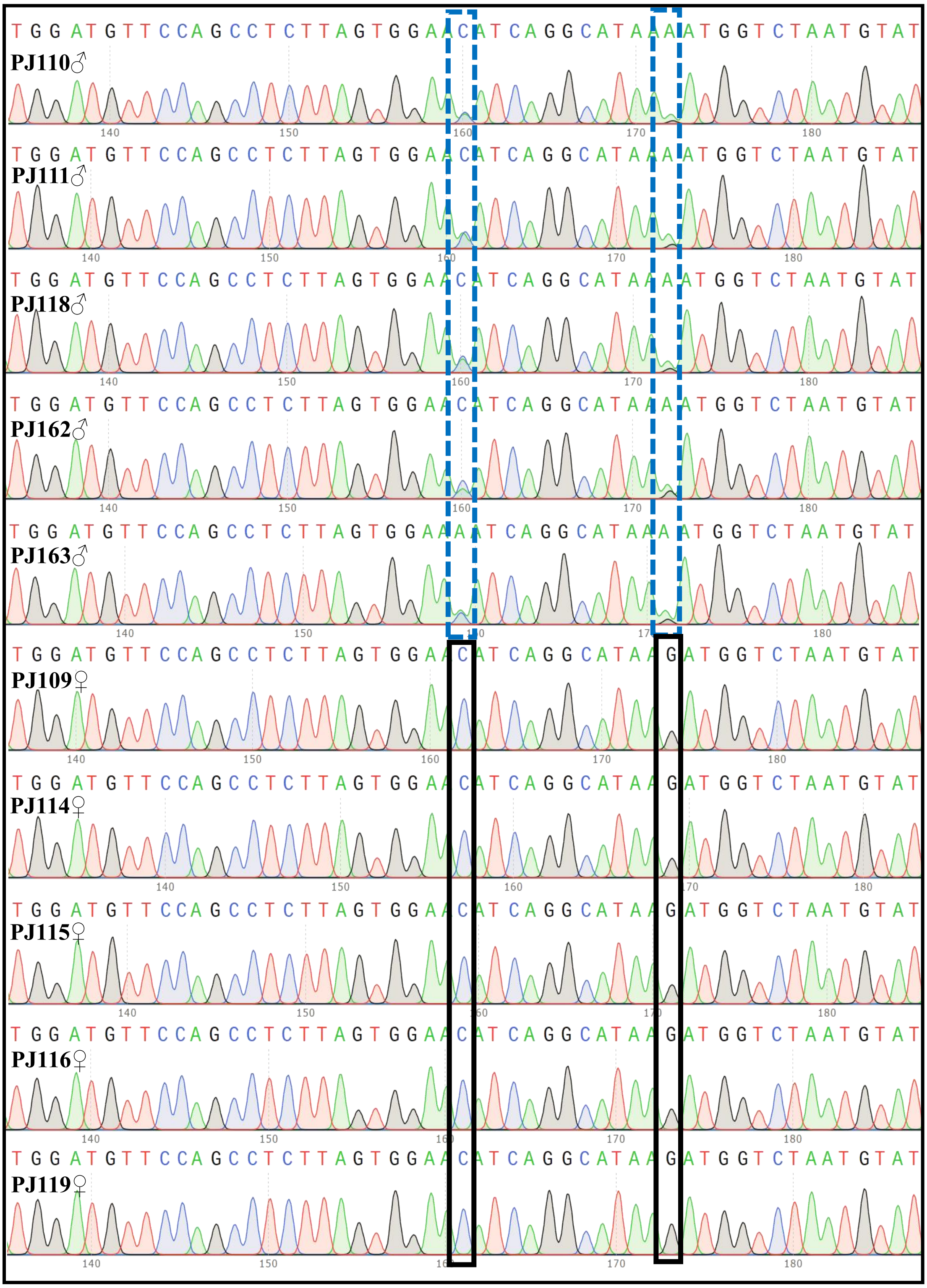

A. tuberodepressus (Clocus_1390631)

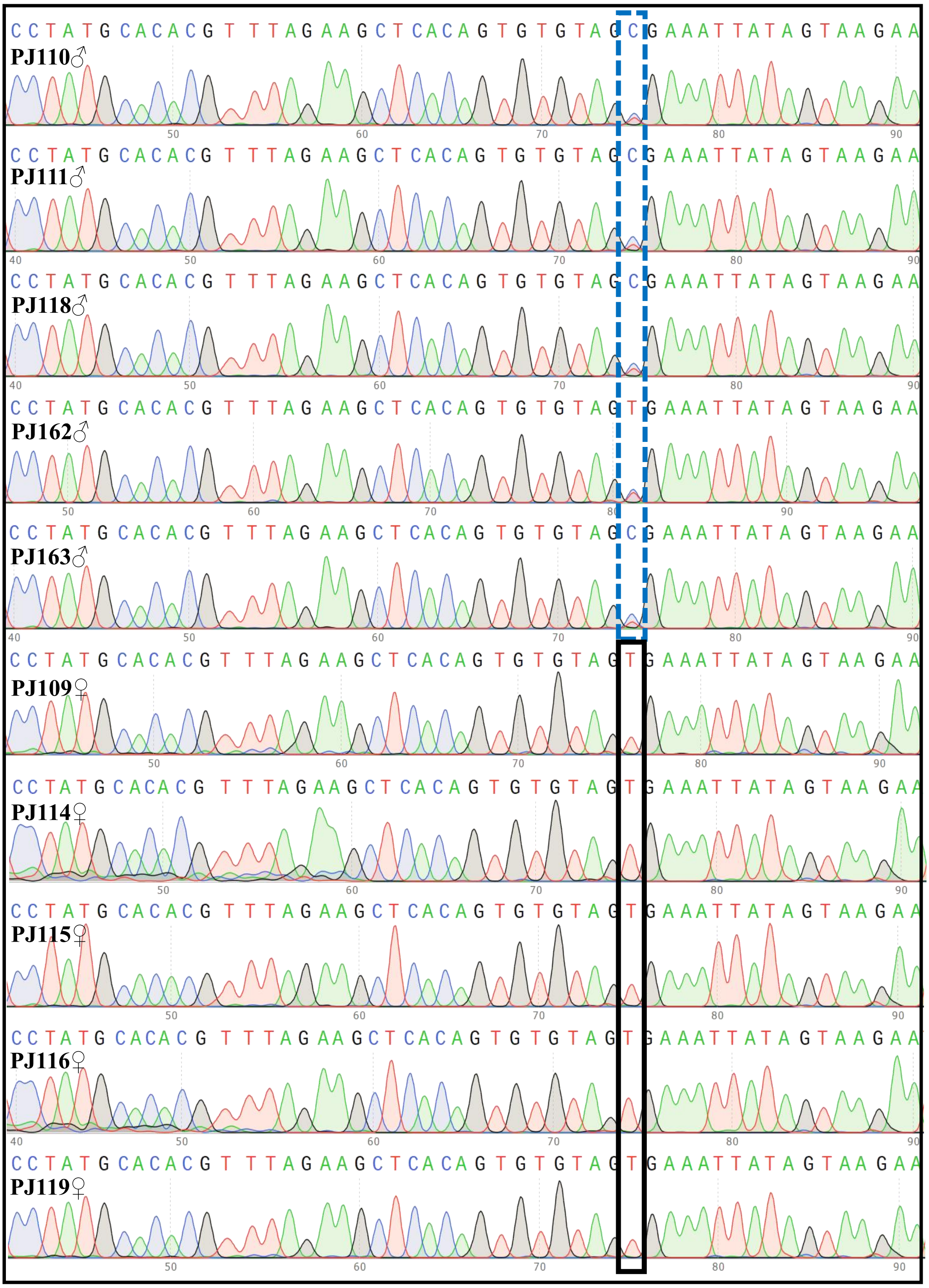

A. tuberodepressus (Clocus_1950702)

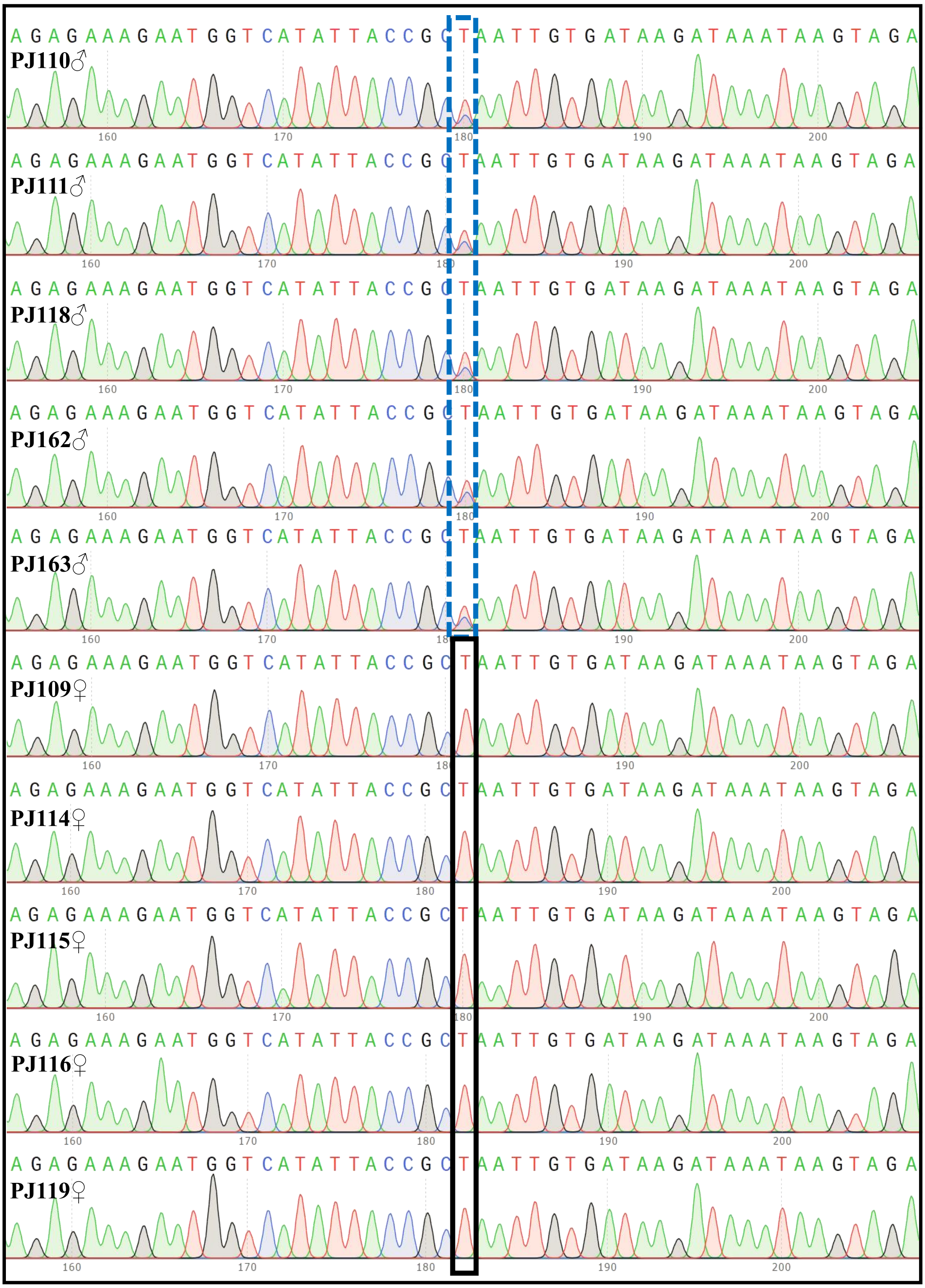

A. tuberodepressus **(Clocus_2024518)**

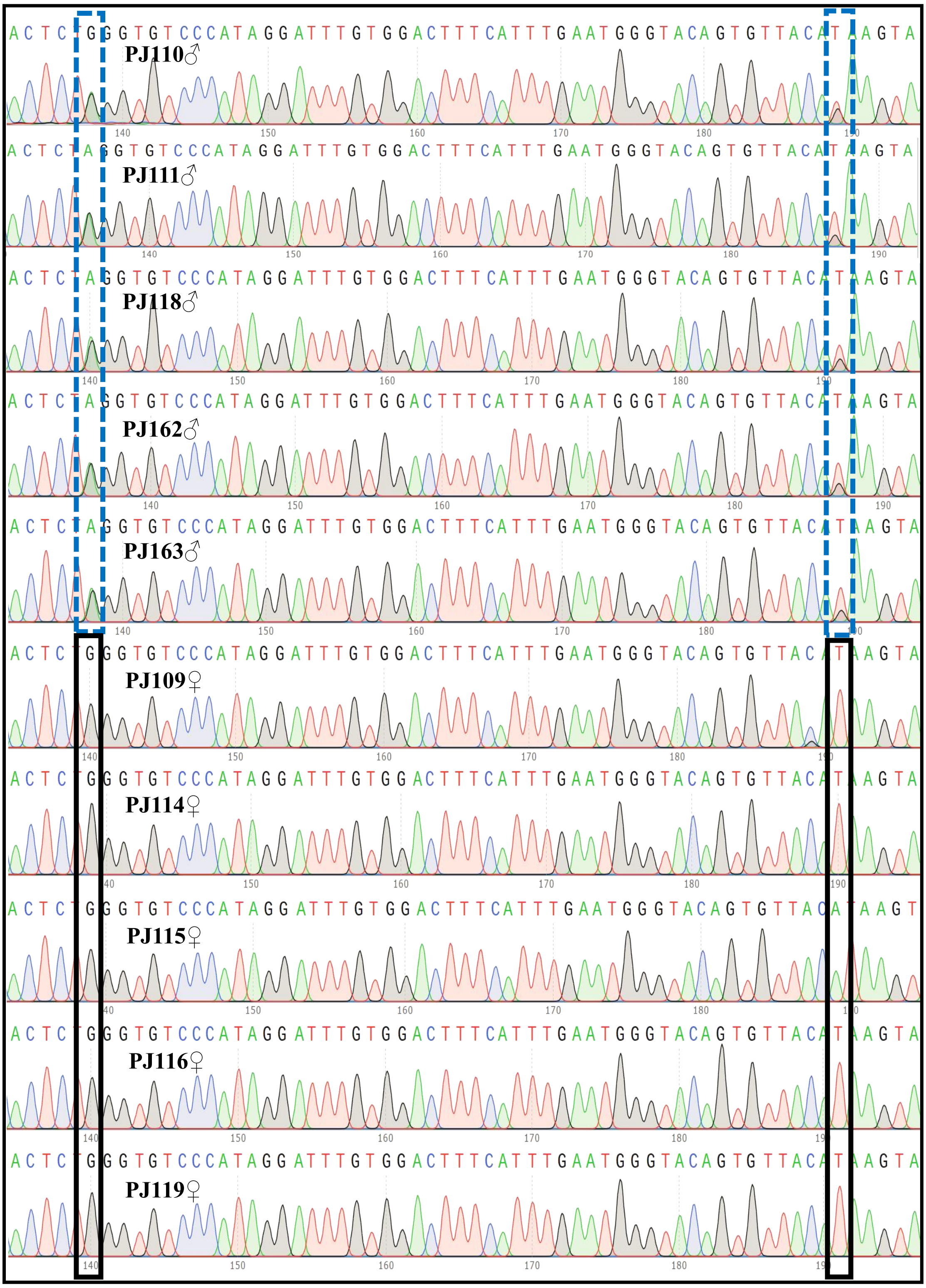

A. tuberodepressus (Clocus_2049833)

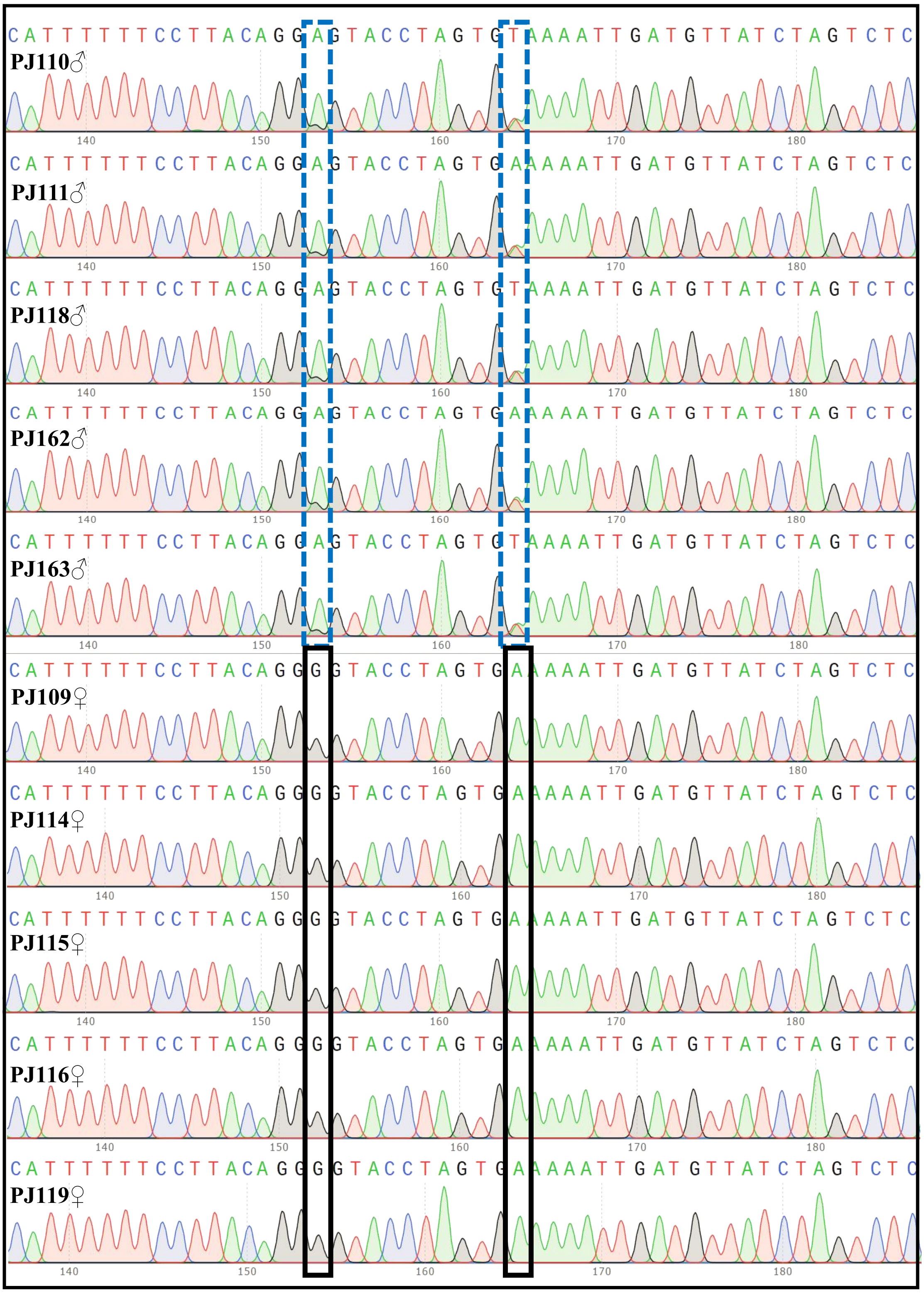

A. wuyiensis (Clocus_117656)

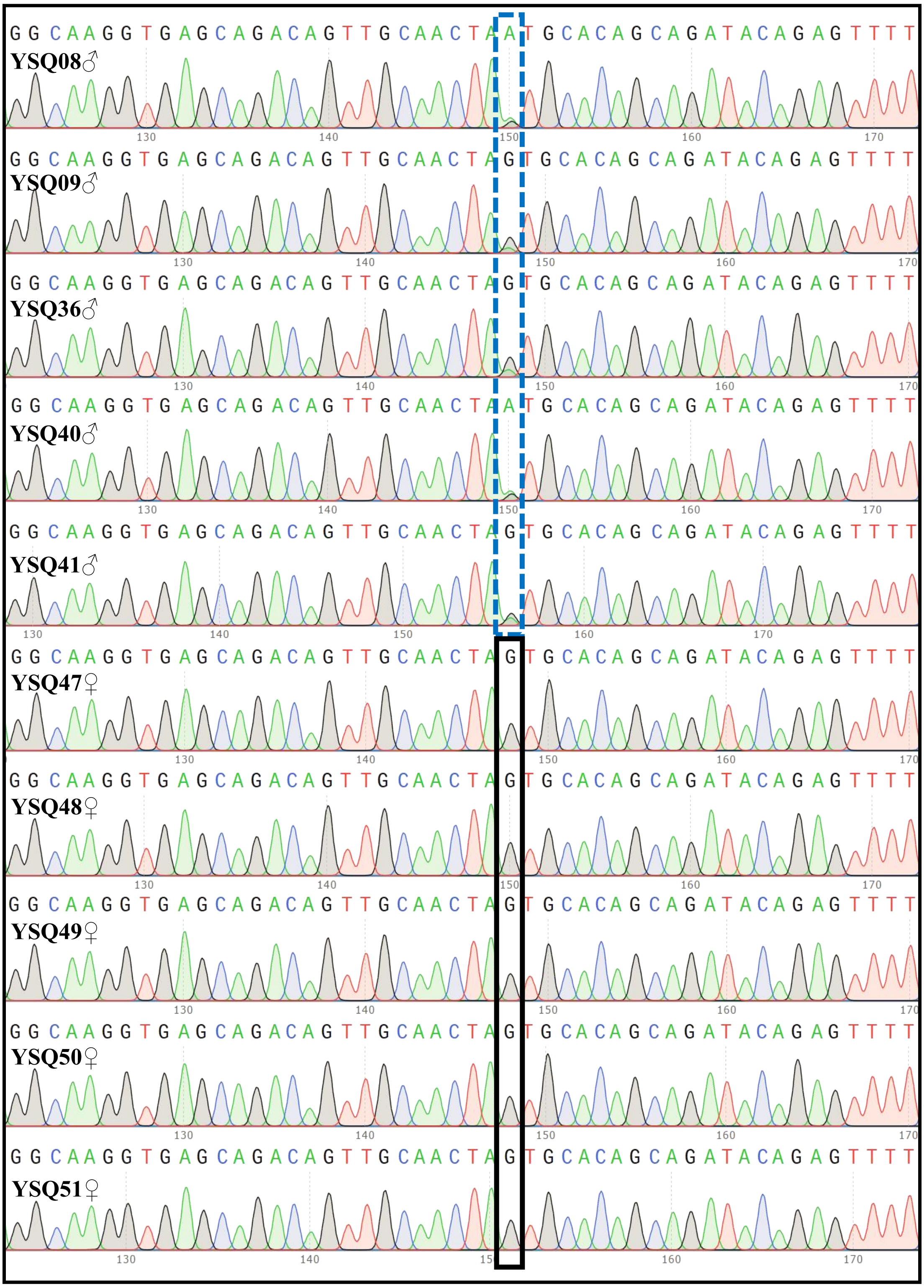

A. wuyiensis (Clocus_120446)

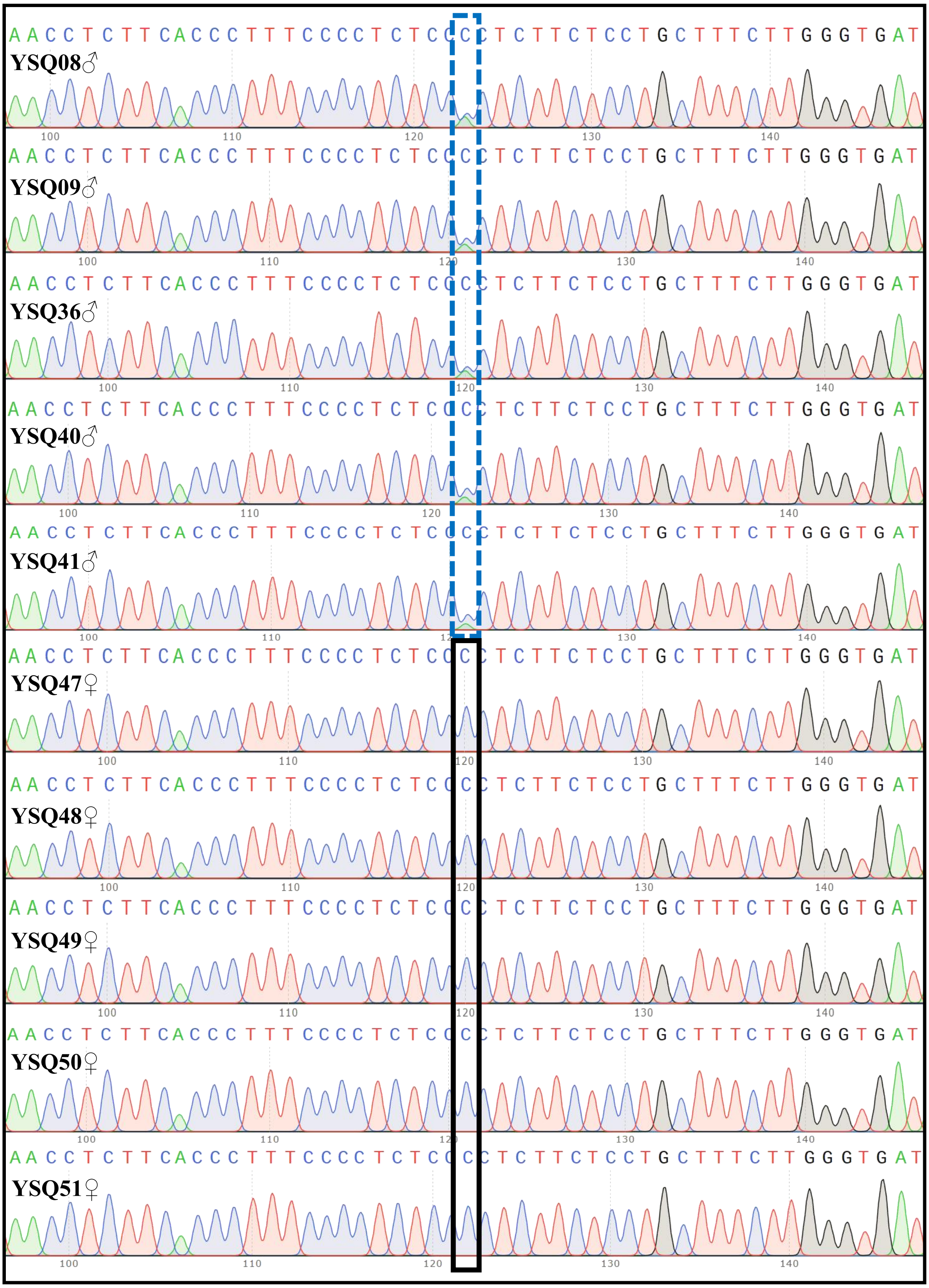

***A. wuyiensis* (Clocus_177528)**

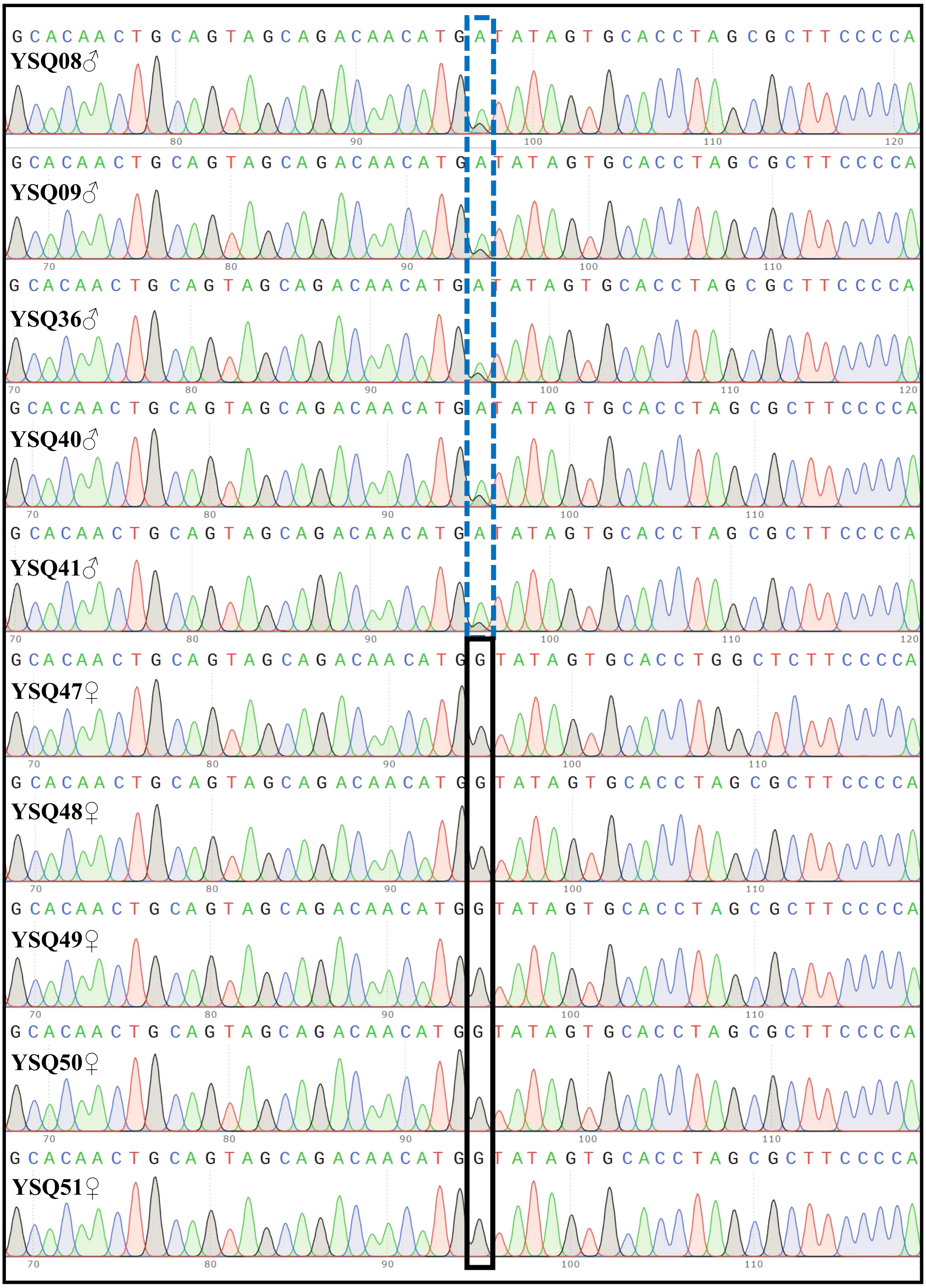

***A. wuyiensis* (Clocus_321845)**

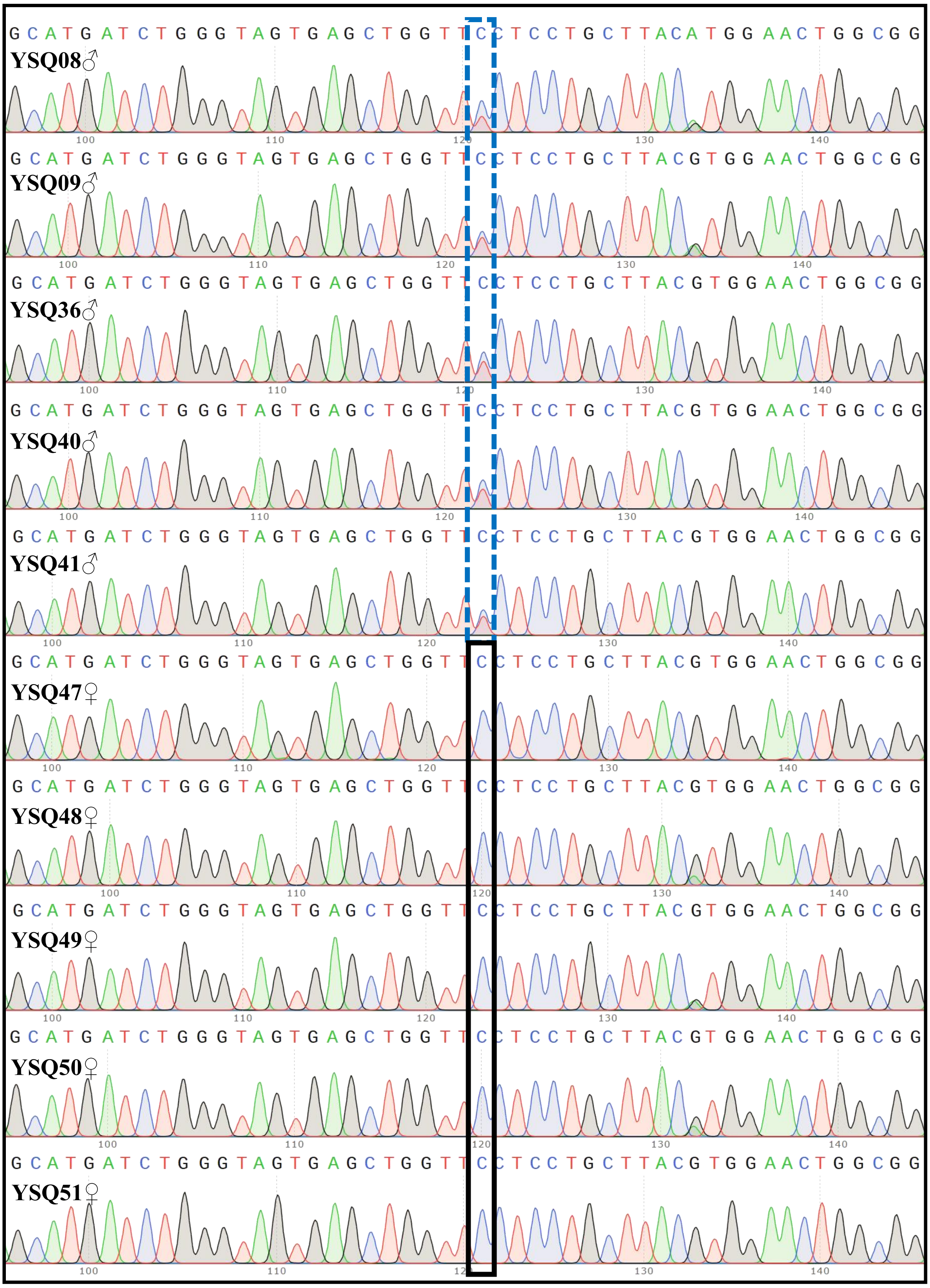

A. wuyiensis **(Clocus_375485)**

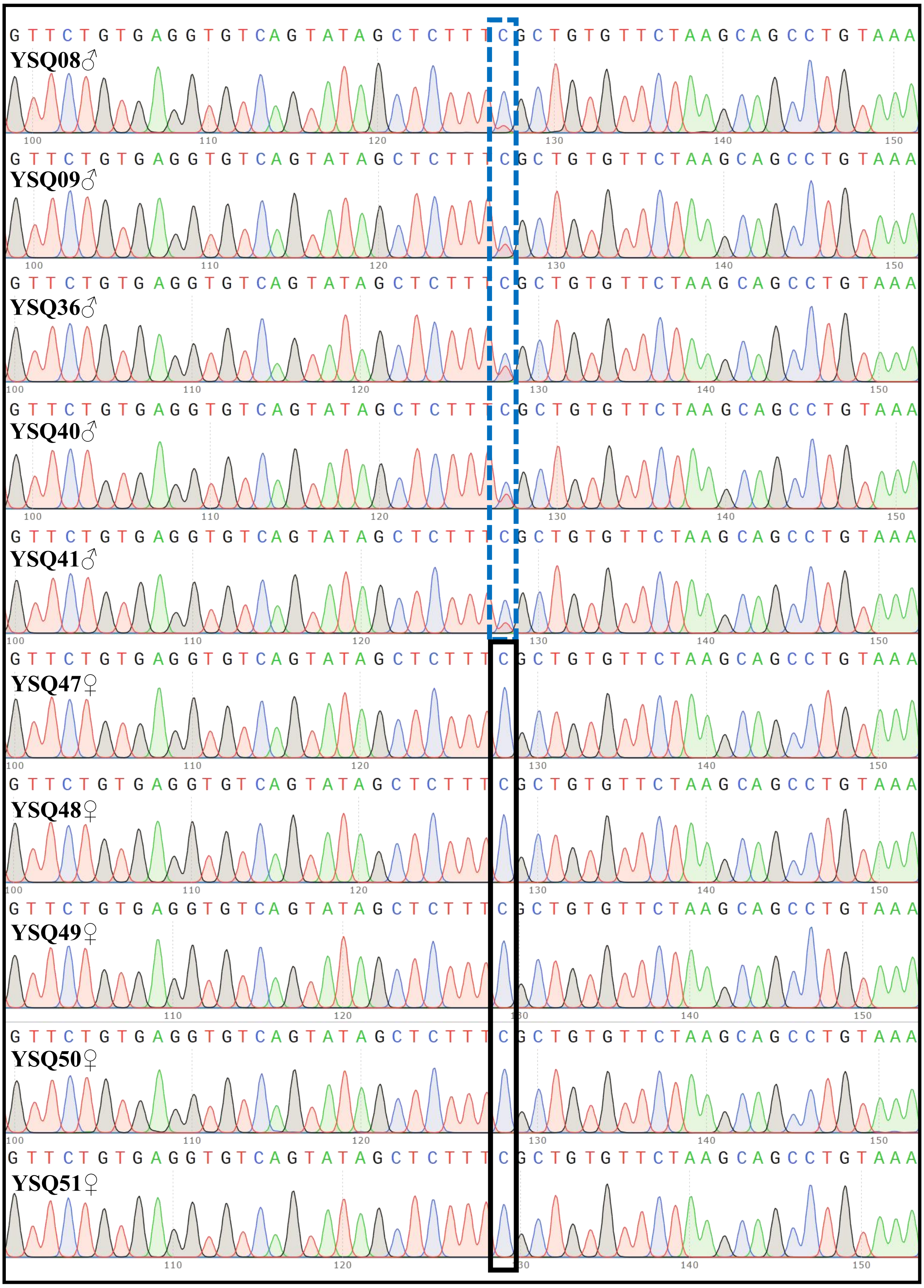

***A. wuyiensis* (Clocus_450377)**

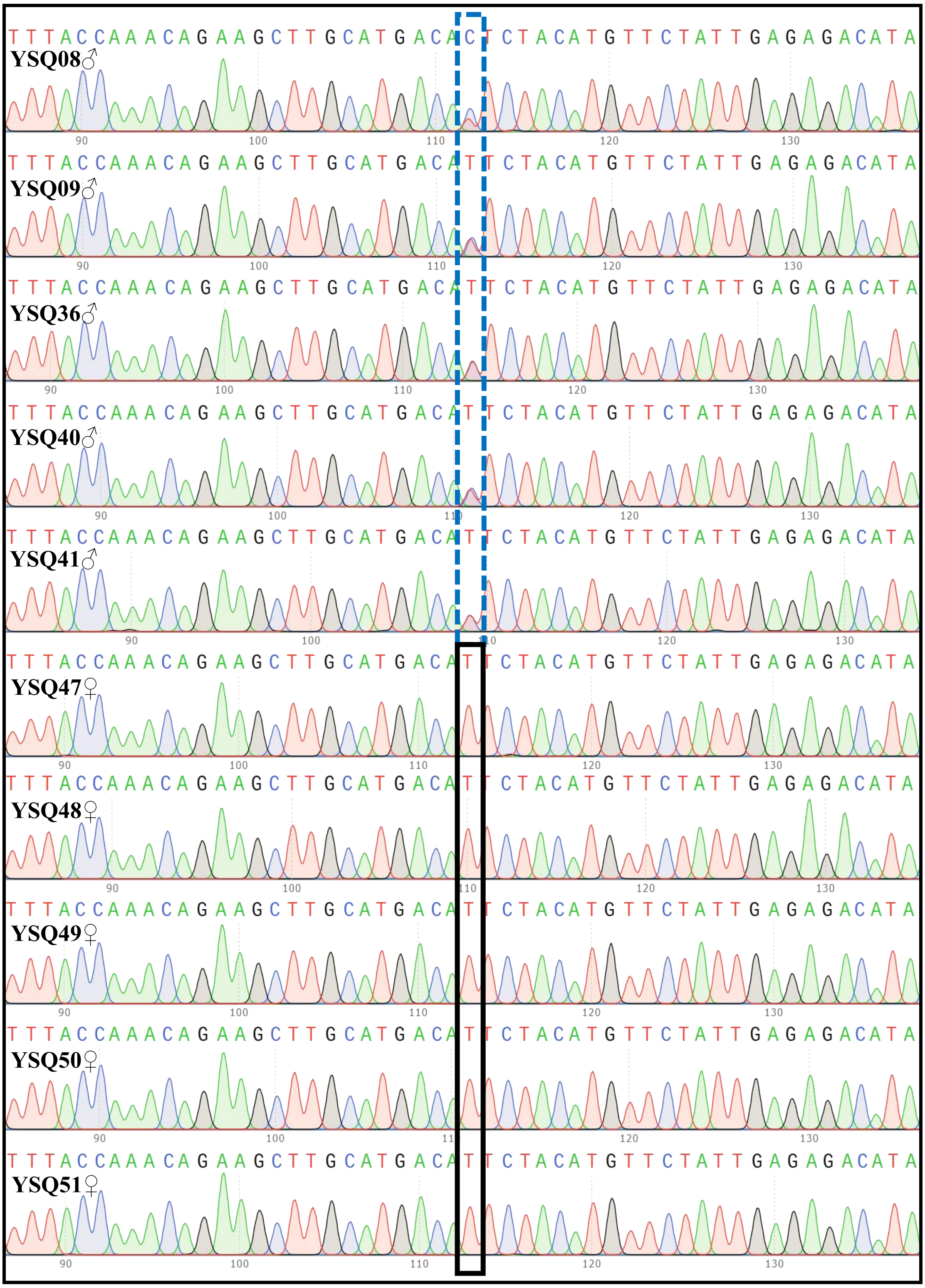

A. wuyiensis (Clocus_1146109)

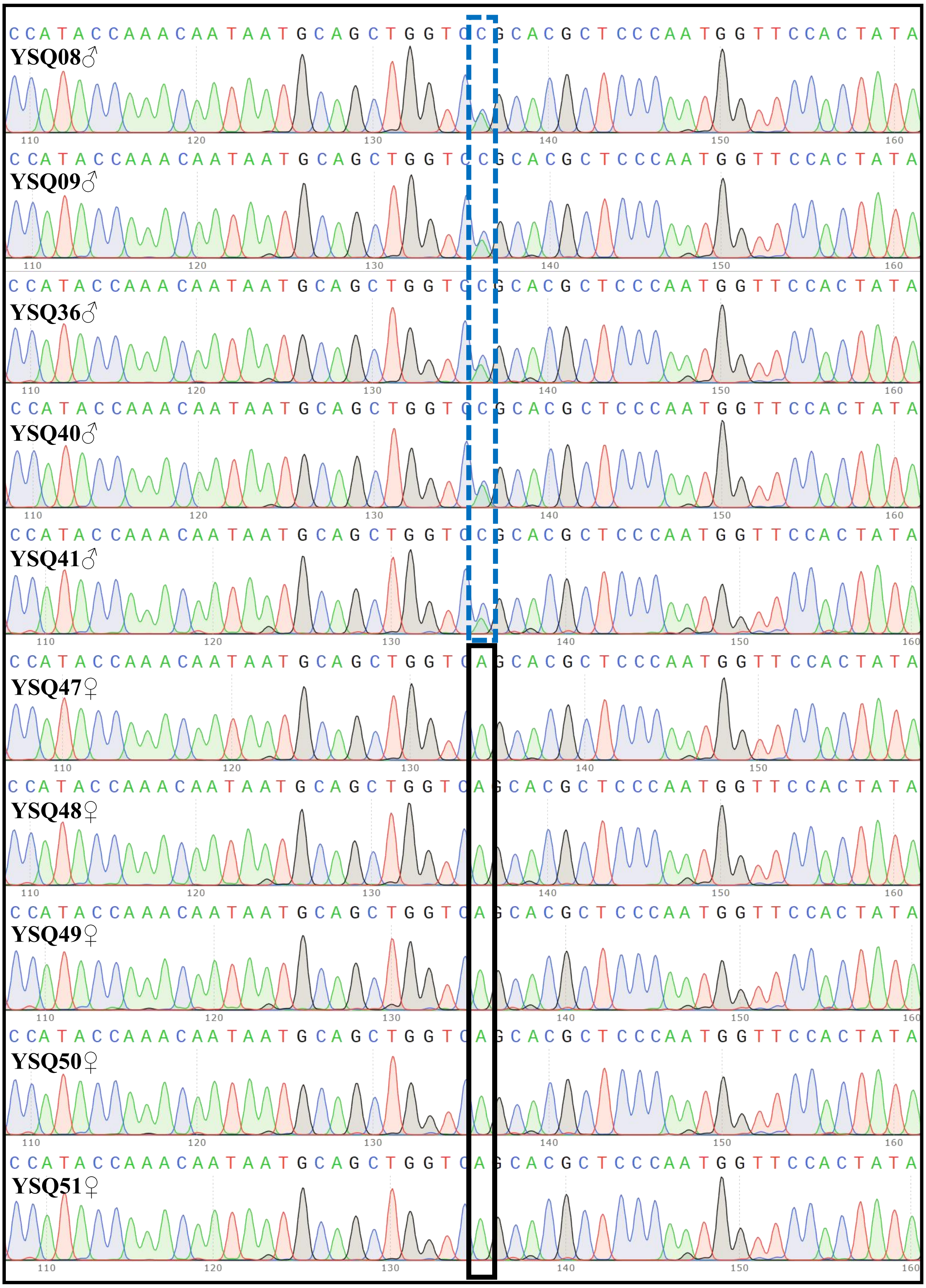

***A. xinduqiao* (Clocus_106254)**

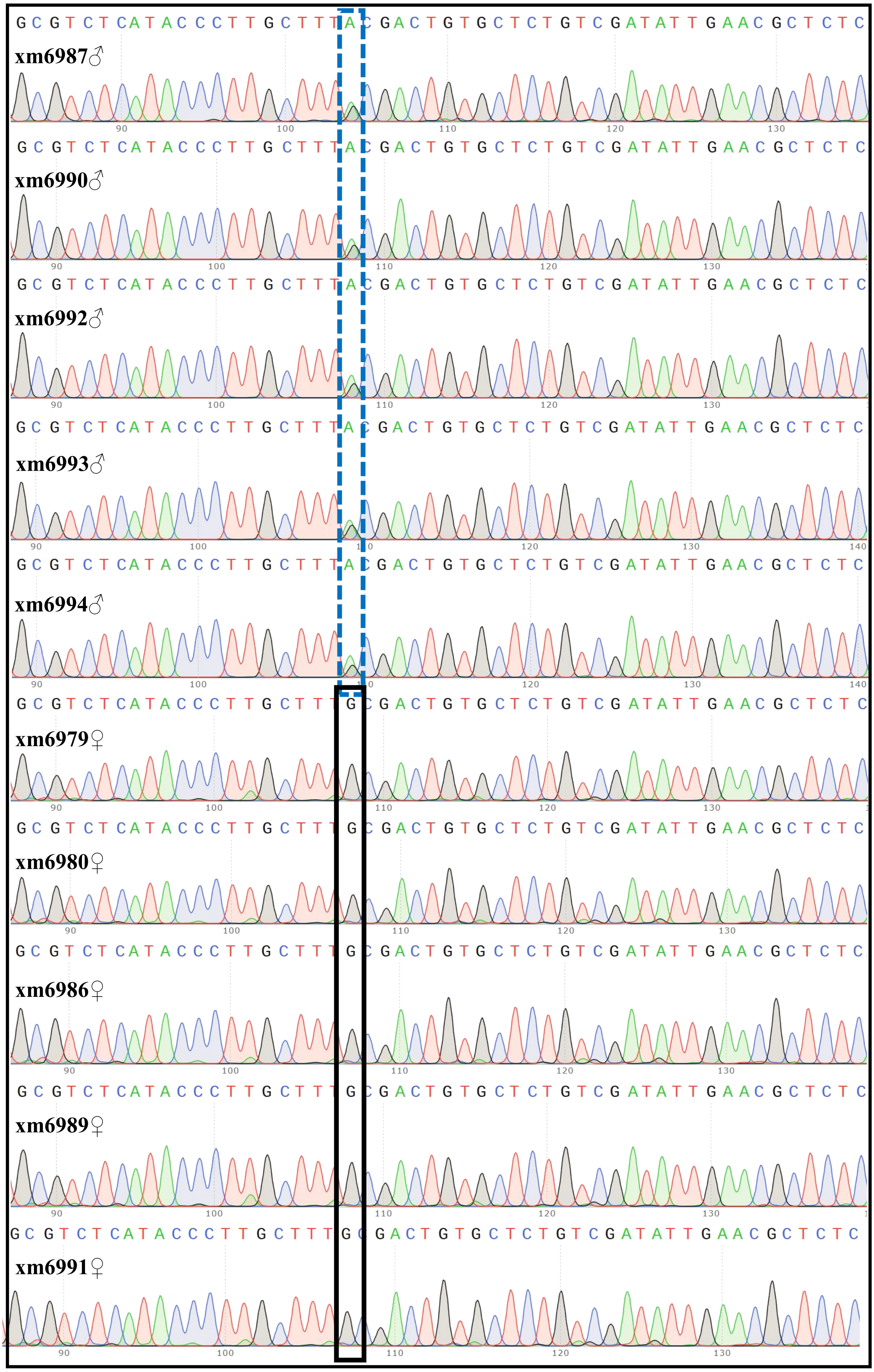

A. xinduqiao **(Clocus_746953)**

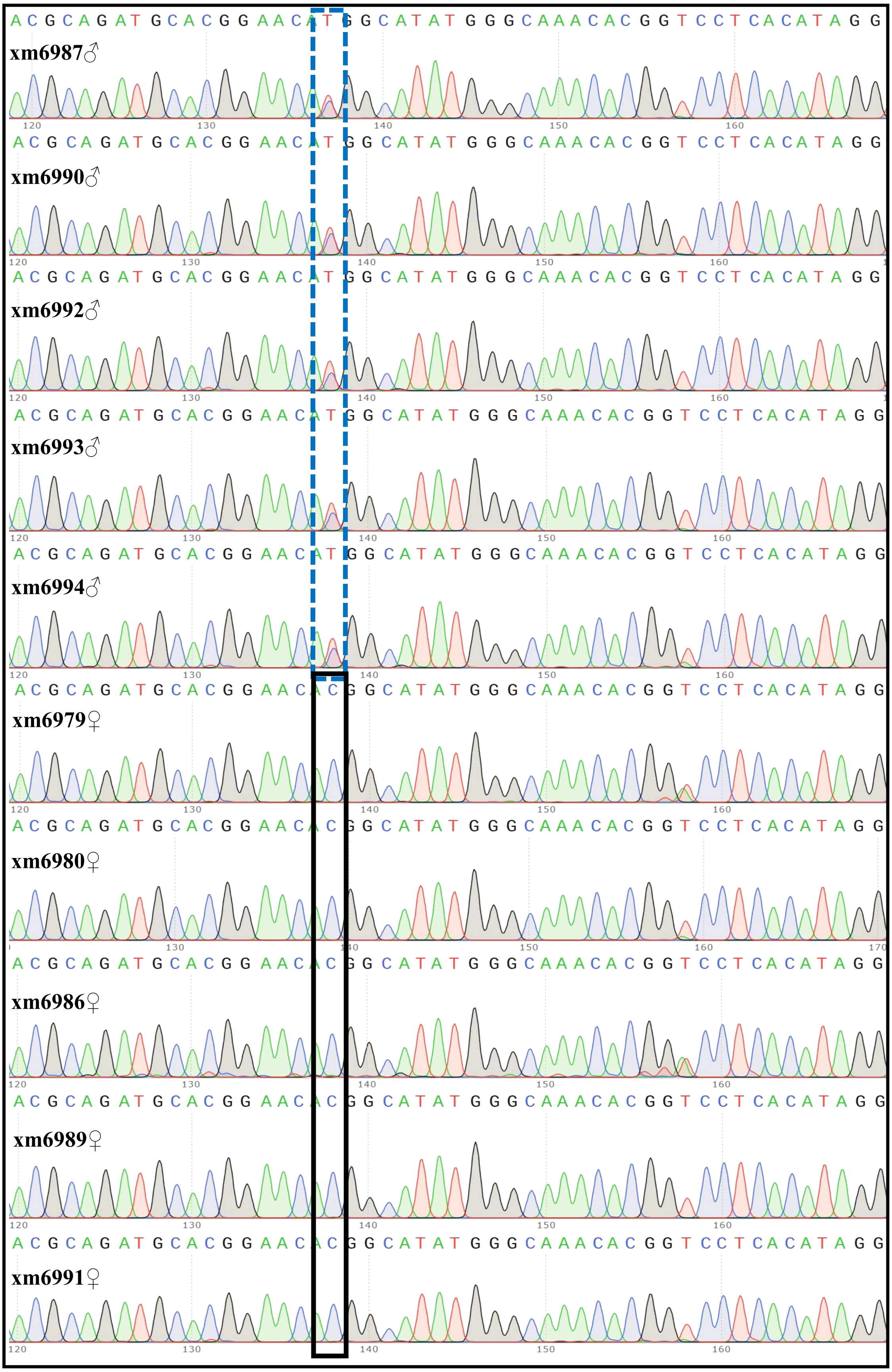

A. xinduqiao **(Clocus_1266715)**

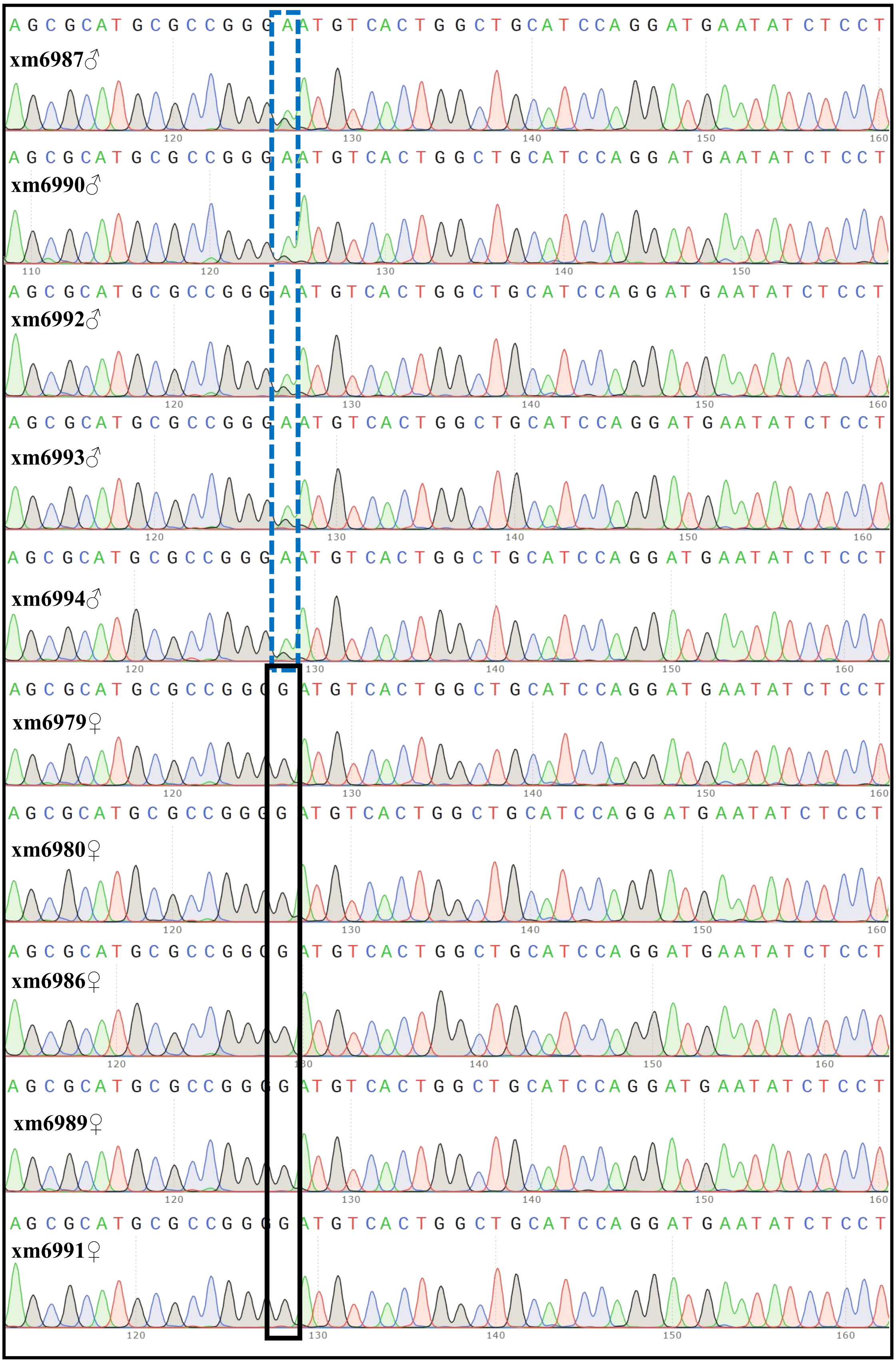

Supplement: Supplementary file 1 [file ijms-23-11146-s001.zip › ijms-1904191-manuscript-supplementary-revised/manuscript-supplementary/File S4.pdf]
