# Supplementary material for: Heterogeneous Evolution of Sex Chromosomes in the Torrent Frog Genus Amolops
Source: Int J Mol Sci. 2022 Sep 22;23(19):11146. doi: 10.3390/ijms231911146 (PMC9570394; doi:10.3390/ijms231911146)

File S5

Rate of aligned sex-linked markers (y axes), per reference chromosome of a third-party genome (*Pyxicephalus adspersus*, *Rana temporaria*, *Rhacophorus kio*), for 9 species of *Amolops* for which a sex chromosome could not be identified due to a lack of a clear consensus from sex linked marker alignments (Indirect positioning method or Direct positioning method). See the materials and methods in the text for details.

Third-party genome: *Pyxicephalus adspersus* (Indirect positioning method)

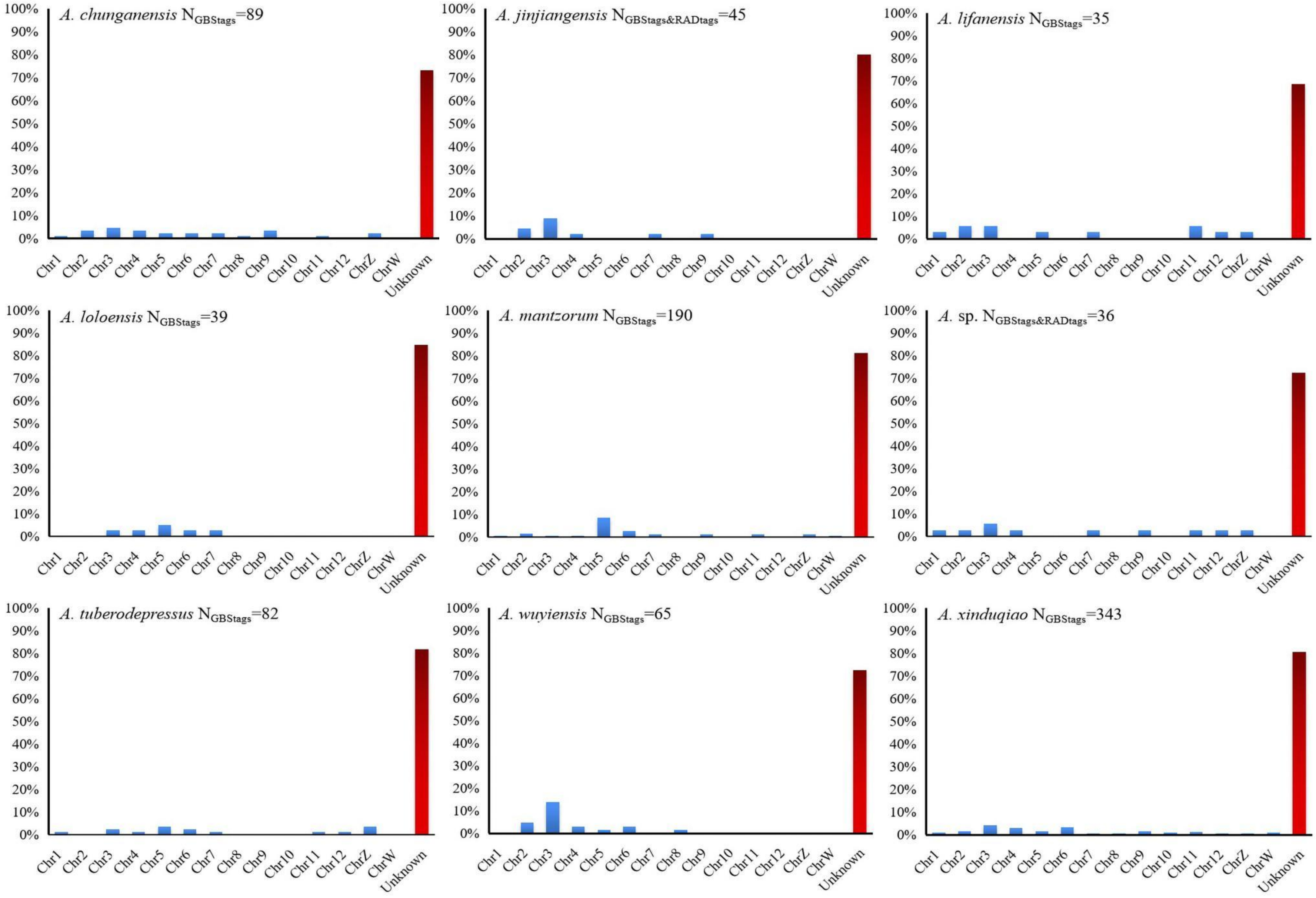

Third-party genome: *Pyxicephalus adspersus* (Direct positioning method)

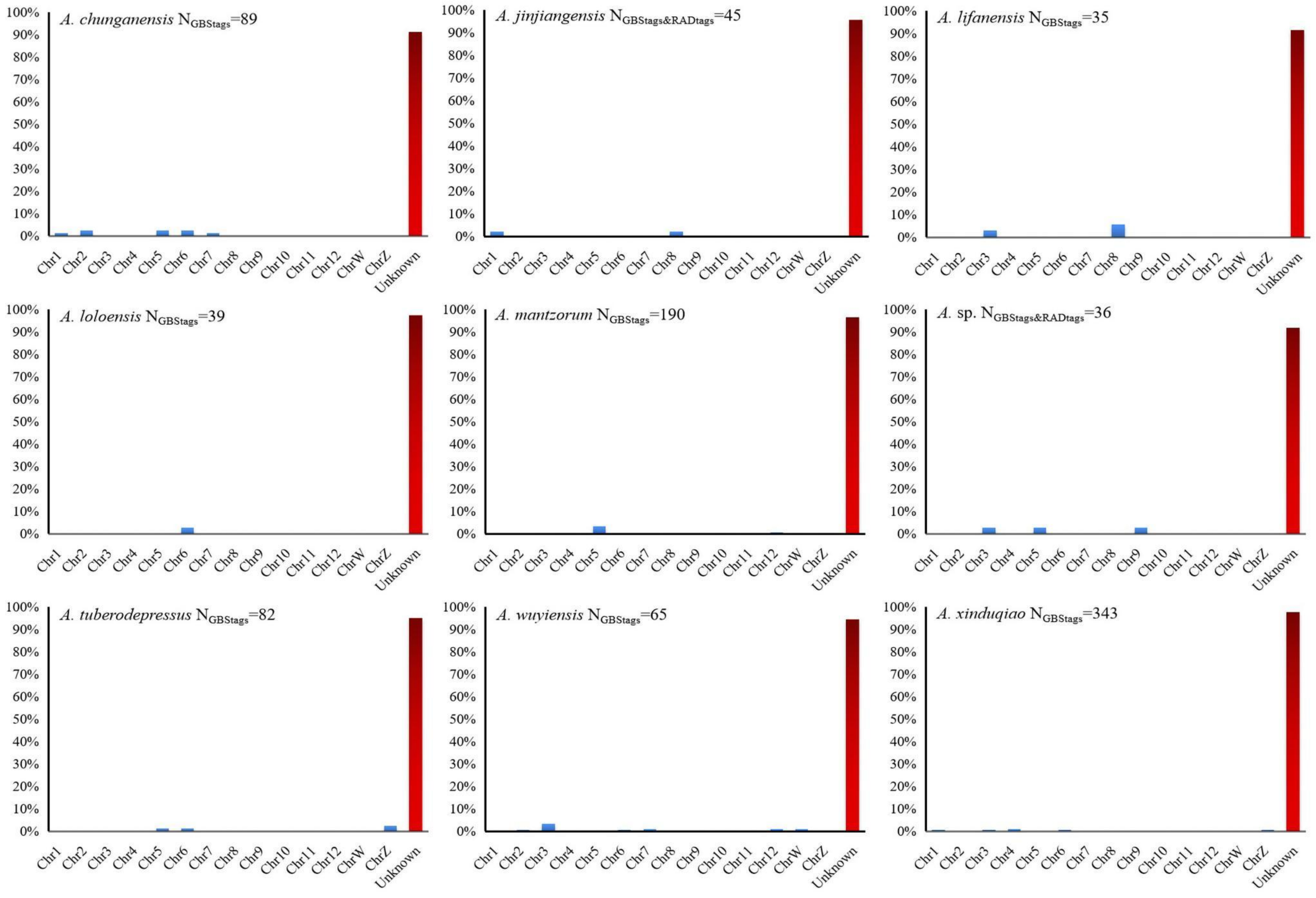

Third-party genome: *Rana temporaria* (Direct positioning method)

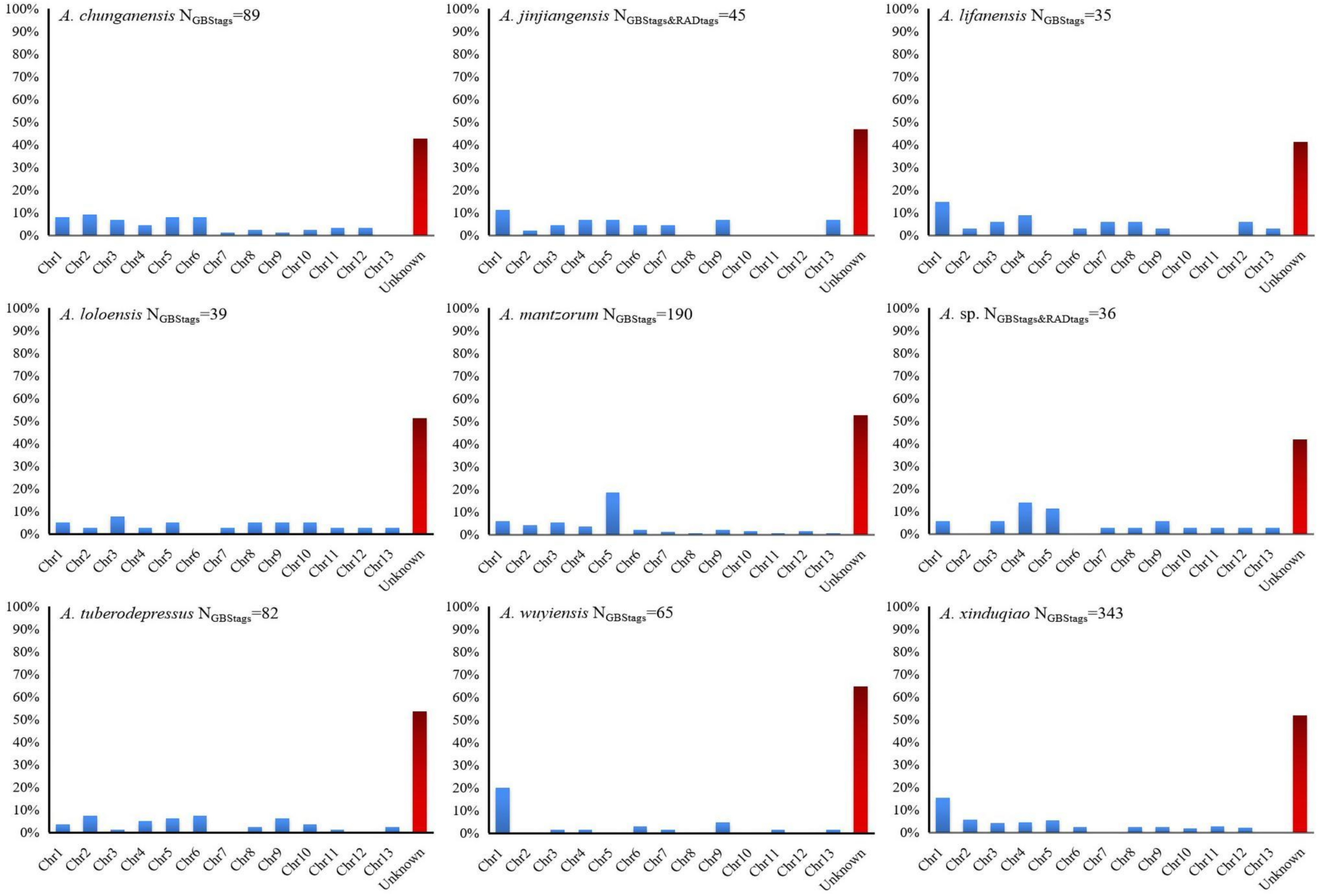

Third-party genome: *Rhacophorus kio* (Direct positioning method)

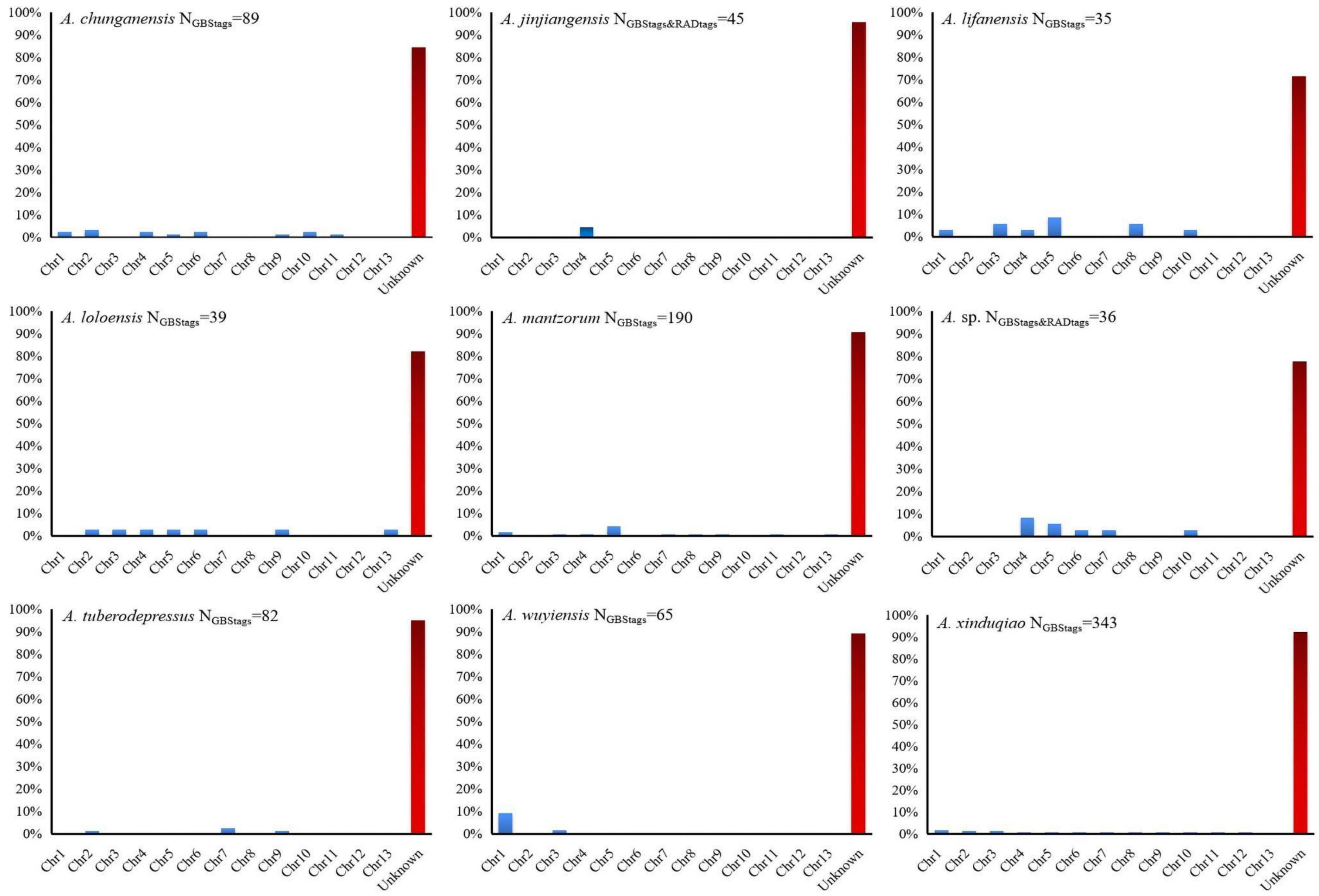

Supplement: Supplementary file 1 [file ijms-23-11146-s001.zip › ijms-1904191-manuscript-supplementary-revised/manuscript-supplementary/File S5.pdf]
