# Supplementary material for: Heterogeneous Evolution of Sex Chromosomes in the Torrent Frog Genus Amolops
Source: Int J Mol Sci. 2022 Sep 22;23(19):11146. doi: 10.3390/ijms231911146 (PMC9570394; doi:10.3390/ijms231911146)
Supplement: Supplementary file 1 [file ijms-23-11146-s001.zip › ijms-1904191-manuscript-supplementary-revised/manuscript-supplementary/File S6.pdf]

File S6

The results of *blastn* alignment between the confirmed sex-linked loci of *Amolops* (*A. chunganensis*, *A. jinjiangensis*, *A. lifanensis*, *A. loloensis*, *A. mantzorum*, *A.* sp., *A. tuberodepressus*, *A. wuyiensis*, *A. xinduqiao*) and the Y chromosome of *A. mantzorum. "*start" and 'end' indicate the nucleotide position.

Table S1. The results of *blastn* alignment between the confirmed sex-linked loci of *A. chunganensis* and the Y chromosome of *A. mantzorum*

Sex-linked markers ID (*A. chunganensis*)	Y chromosome ID	Identical(%)	Evalue	Bitscore	Start (*A. chunganensis*)	End (*A. chunganensis*)	Start (Y chromosome)	End (Y chromosome)
23397	4847706	90.688	1.64E-88	327	1	246	246	1
194216	4653768	79.208	1.26E-30	135	87	287	193	1
239118	5042684	91.632	2.77E-87	324	1	233	123	354
244060	4691324	92.208	9.39E-22	106	1	73	80	4
317179	4951656	89.916	1.09E-35	152	73	189	154	272
343083	4733108	90.863	1.19E-70	268	1	196	192	1
388244	1277224	97.917	4.3E-65	250	155	298	5	148
697780	5071580	80.12	3.45E-61	237	1	306	400	74
726405	4768762	91.209	2.48E-27	124	184	274	235	145
788298	1273427	96.35	7.25E-58	226	8	144	1	137
803318	5051111	91.085	3.68E-95	350	12	269	278	21
865634	4668121	89.352	1.03E-70	268	27	238	218	3
938946	5021552	91.549	2.05E-48	195	155	296	64	203
1222271	4891674	95.437	3.67E-115	416	1	260	262	1
1395828	4629391	91.566	2.91E-62	241	164	329	1	166
1496617	4776381	86.818	6.3E-63	243	3	219	17	235
1499023	4777379	86.134	4.05E-65	250	46	278	2	237
1594920	1500316	93.525	2.63E-52	207	6	144	1	139
1777161	4911526	81.356	3.25E-31	137	71	240	236	60

2038902	3727802	87.64	3.15E-21	104	11	99	34	122
2083719	2551300	85.507	3.4E-51	204	59	265	5	197
2189594	5001519	88.142	5.03E-79	296	26	275	314	65
2246425	5013167	91.667	1.58E-49	198	1	144	296	154
2445832	1518668	95.266	3.94E-70	267	38	206	18	185
2461097	4827833	80.321	3.45E-41	171	1	246	226	2
2463324	5075053	87.259	1.95E-83	311	41	298	42	295
2490984	1168837	96.19	9.58E-42	172	1	105	23	127
2607230	4920253	91.333	3.46E-51	204	155	302	251	102
2743456	4874750	94.253	4.64E-30	134	161	247	148	234
3166758	4900362	85.876	3.45E-41	171	52	225	1	161
3217147	4753254	94.737	6.45E-43	176	1	114	118	230
3589609	5021395	97.656	1.04E-55	219	1	127	243	116
3721673	4762650	91.406	9.2E-42	172	150	277	68	192
39171	4934951	95.139	2.02E-58	228	1	144	146	3
71696	5019902	93.254	2.91E-101	370	1	252	251	1
281026	4187937	90.598	9.65E-37	156	178	294	117	1
343847	1334159	86.826	6.67E-43	176	1	155	167	1
367209	4766533	94.366	1.21E-55	219	157	298	231	90
461783	4882667	91.096	2.05E-48	195	155	297	61	205
647984	4771414	88.889	2.06E-43	178	1	144	53	196
775866	5075936	89.964	1.07E-95	351	1	279	308	39
1799338	4820725	86.722	2.54E-67	257	20	250	245	7
2307679	4972100	91.636	3.77E-105	383	1	275	275	2

Table S2. The results of *blastn* alignment between the confirmed sex-linked loci of *A. jinjiangensis* and the Y chromosome of *A. mantzorum*

Sex-linked markers ID (*A. jinjiangensis*)	Y chromosome ID	Identical(%)	Evalue	Bitscore	Start (*A. jinjiangensis*)	End (*A. jinjiangensis*)	Start (Y chromosome)	End (Y chromosome)
109618	4860987	93.793	4.36E-55	217	1	144	30	173
204309	4721776	97.744	5.67E-59	230	1	133	95	227
261570	5073809	86.139	2.02E-58	228	7	207	315	117
263214	4568628	91.667	4.4E-50	200	1	144	21	164
361666	1418531	98.611	9.25E-67	255	155	298	155	12
421944	1266847	93.571	2.67E-52	207	95	231	140	1
449150	4917731	91.6	2.46E-97	357	51	298	1	246
458997	4803217	94	9.38E-57	222	1	144	196	47
478746	4603552	96.364	1.6E-44	182	1	110	38	147
483348	1293126	97.692	2.61E-57	224	155	284	43	172
1518147	4946231	85.938	4.53E-55	217	84	263	67	258
32511	1892343	83.766	2.63E-32	141	11	159	159	7
273329	4858987	82.833	8.14E-48	193	63	280	232	2
427939	5081765	94.521	9.38E-57	222	2	144	834	979
700074	4838829	92.941	1.41E-64	248	1	170	171	2
1196026	3313201	92.143	4.79E-49	196	1	139	175	36
26967	5050277	99.005	1.23E-98	361	1	201	359	159
210224	4799970	90.698	2.45E-58	228	100	270	245	75
508443	4538782	99.457	1.05E-90	335	90	273	12	195

785681	5071508	84.047	3.81E-60	233	3	258	109	351
1320445	4869987	91.304	4.72E-55	217	1	159	8	167
1690024	5028514	89.732	4.34E-75	283	85	304	336	113
2601070	4636847	95.238	4.02E-70	267	109	276	168	1
3048173	1297365	97.794	9.92E-61	235	4	139	181	46
3081833	1349511	93.617	1.14E-74	281	42	229	191	4
3451145	4757212	94.175	2.13E-37	158	140	242	192	90
3867224	4578511	95.89	3.87E-61	237	1	146	59	204
4557485	1518864	94.667	4.17E-60	233	139	288	2	151
8973283	1297495	95.531	1.98E-76	287	1	179	3	181

Table S3. The results of *blastn* alignment between the confirmed sex-linked loci of *A. lifanensis* and the Y chromosome of *A. mantzorum*

Sex-linked markers ID (*A. lifanensis*)	Y chromosome ID	Identical(%)	Evalue	Bitscore	Start (*A. lifanensis*)	End (*A. lifanensis*)	Start (Y chromosome)	End (Y chromosome)
3479	4643523	85.922	1.35E-55	219	21	224	206	1
128621	4720454	88.596	4.24E-75	283	1	228	8	228
149043	4946728	82.197	1.21E-60	235	1	252	21	284
174627	1083669	97.222	4.43E-45	183	1	108	1	108
345647	4682439	86.364	9.52E-57	222	87	302	1	204
409308	4850610	88	1.49E-24	115	1	97	50	149
430733	4991220	85.985	4.24E-75	283	36	295	1	257
520205	4878112	84.038	1.21E-55	219	1	213	208	1
549218	2505138	87.838	4.46E-40	167	155	298	3	146
611132	4804048	85.366	4.27E-70	267	60	298	1	246
626509	4882993	85	4.3E-65	250	1	260	3	247
630417	5035687	90.53	4.85E-94	346	12	273	1	263
868895	4920882	80.597	8.61E-47	189	22	272	267	2
1198537	4840609	91.473	1.47E-99	364	16	273	255	1
1199993	1608674	93.651	3.42E-46	187	174	298	199	75
1216720	1509566	84.672	3.5E-31	137	2	138	2	138
1622760	4709637	91.593	5.05E-84	313	14	239	226	1

1734074	4829178	84.549	7.2E-63	243	35	267	250	25
952	5067871	85.075	9.1E-52	206	1	201	210	10
367283	4739216	87.342	1.99E-68	261	56	291	227	1
538393	4916377	100	1.21E-30	135	132	204	274	202
742878	4766705	92	7.44E-83	309	61	285	238	22
932951	1944978	97.297	4.99E-84	313	1	183	16	200
1854002	4964521	93.827	4.77E-100	366	59	301	243	1

Table S4. The results of *blastn* alignment between the confirmed sex-linked loci of *A. loloensis* and the Y chromosome of *A. mantzorum*

Sex-linked markers ID (*A. loloensis*)	Y chromosome ID	Identical(%)	Evalue	Bitscore	Start (*A. loloensis*)	End (*A. loloensis*)	Start Y chromosome	End Y chromosome
17160	4374706	97.516	5.99E-74	279	61	221	2	162
108219	5022234	95.862	4.33E-60	233	155	298	122	265
222513	1669284	90.244	2.98E-22	108	156	237	119	38
437654	4996225	93.75	4.36E-55	217	1	144	206	63
474811	1606400	97.315	3.39E-66	254	1	149	21	169
567427	3261089	96.479	4.40E-60	233	161	302	31	170
666795	4826205	91	4.52E-30	134	199	298	75	173
672096	4588967	93.662	2.04E-53	211	1	140	66	207
682957	4804007	86.111	9.18E-72	272	10	252	1	246
759013	1108966	99.038	3.42E-46	187	1	104	104	1
771638	4797436	90.909	1.61E-34	148	7	116	244	135
939843	3494899	94.483	9.66E-57	222	162	306	12	155
1025247	4584187	83.069	5.73E-44	180	47	235	194	10
1179745	1519702	94.215	1.23E-45	185	155	275	141	21
1181621	4590277	94.521	2.61E-57	224	1	144	194	49
1303568	4762580	81.818	2.63E-52	207	43	267	7	237
1441139	4934951	96.503	3.35E-61	237	1	143	146	4
1444271	5081022	77.992	7.41E-43	176	15	269	226	483
1557047	5003094	80.707	3.71E-61	237	42	327	315	5
56610	4851114	88.276	9.58E-42	172	1	144	15	159
108264	4788343	86.301	3.47E-36	154	155	298	100	242
175943	5071735	78.616	3.41E-51	204	2	299	375	65

Table S5. The results of *blastn* alignment between the confirmed sex-linked loci of *A. mantzorum* and the Y chromosome of *A. mantzorum*

Sex-linked markers ID (*A. mantzorum*)	Y chromosome ID	Identical(%)	Evalue	Bitscore	Start (*A. mantzorum*)	End (*A. mantzorum*)	Start (Y chromosome)	End (Y chromosome)
18244	4642954	96.377	1.62E-58	228	100	237	138	1
26775	4968478	100	4.07E-50	200	1	108	108	1
28208	5032852	86.154	1.54E-69	265	28	285	4	247
30397	5022380	84.436	1.02E-65	252	1	257	295	40
37788	4976536	82.963	1.02E-25	119	113	246	1	133
53454	5057235	93.056	2.03E-53	211	155	298	384	241
58043	1420931	93	1.7E-33	145	136	235	193	95
63182	5067196	94.631	3.93E-130	466	1	298	56	348
87000	3211188	96.429	9.72E-32	139	4	87	90	7
95424	3672608	96.61	5.69E-49	196	159	276	200	83
97147	2240852	89.375	1.57E-50	202	1	160	183	24
121923	4972834	91.765	1.05E-95	351	1	253	42	295
155678	5023240	88.538	2.52E-82	307	65	298	332	80
164925	5081665	86.458	8.99E-87	322	1	275	411	125
173183	3249843	93.642	6.73E-68	259	1	173	28	200
190141	4943254	100	3.52E-80	300	1	162	187	26
204894	4633153	100	1.58E-49	198	1	107	90	196
207705	3898677	95.62	3.41E-56	220	148	284	200	64
209662	4574591	95.833	4.33E-60	233	155	298	36	179
232286	5004750	87.395	4.24E-75	283	1	238	84	317

233351	4852818	95.726	6.81E-103	375	65	298	257	31
234342	5012283	100	2.58E-106	387	1	209	209	1
248054	1404413	89.437	5.81E-44	180	161	302	43	184
265765	5015806	98.467	2.01E-127	457	1	257	3	263
265999	2009904	100	3.16E-100	366	39	236	1	198
300023	4031839	96.825	1.75E-53	211	1	126	126	1
328452	4664278	97.561	2.03E-53	211	1	123	49	171
328800	2901679	95.276	4.4E-50	200	1	125	128	2
337208	4979060	83.621	2.56E-52	207	65	289	230	3
389234	5068893	81.034	1.57E-54	215	18	298	127	403
401704	5017859	84.337	3.68E-60	233	10	257	258	20
418422	5035965	90.987	1.21E-84	315	1	233	114	346
480380	5036725	93.6	3.42E-46	187	1	125	126	250
484859	4726950	92.222	7.69E-67	255	1	180	226	47
537404	4791185	95.276	6.76E-108	392	9	262	243	1
562586	4866337	95.868	1.12E-110	401	57	298	1	239
566413	3341701	91.121	1.99E-73	278	36	249	200	1
570183	4987816	90	2.08E-87	324	1	250	26	275
584035	4116935	94	2.49E-81	303	27	226	200	1
615478	4545862	94.595	5.45E-79	296	1	185	182	1
625139	4921675	100	7.24E-67	255	102	239	1	138
647202	4972834	91.732	3.79E-95	350	25	276	294	42
651335	4851316	91.791	3.42E-46	187	166	298	134	1
658924	5054703	85.666	1.92E-78	294	1	293	325	48

685652	3367885	97.917	4.3E-65	250	1	144	153	10
690472	2371873	91.463	6.88E-58	226	107	270	1	164
743122	1571128	91.057	3.79E-40	167	27	149	198	76
744602	1244361	92.632	2.74E-31	137	142	236	127	33
746146	4570675	98.795	1.38E-34	148	177	259	1	83
763836	4772240	93.182	5.23E-29	130	182	269	1	88
769733	4609666	100	1.57E-54	215	183	298	5	120
776062	4766190	95.089	2.46E-97	357	75	298	1	219
776905	1570806	97.987	6.86E-68	259	139	287	1	149
777911	4724642	85.811	9.79E-37	156	1	148	223	78
802711	1410597	100	2.72E-61	237	120	247	193	66
819045	5076008	99.585	1.88E-122	440	1	241	476	236
829082	4623078	100	1.15E-79	298	72	232	205	45
834130	4834986	100	8.17E-27	122	174	239	66	1
852213	4756510	87.838	9.79E-42	172	1	146	230	83
855435	4648778	99.49	2.17E-97	357	71	266	1	196
869165	4760597	91.08	2.64E-76	287	34	245	236	24
883143	1581493	100	2.64E-41	171	143	234	140	49
891681	5013683	91.892	1.23E-50	202	3	145	131	278
894250	3510947	85.279	6.03E-48	193	1	196	5	191
900558	4532774	92.233	4.48E-34	147	110	212	116	14
915590	1576788	97.479	3.4E-51	204	180	298	7	125
916581	4962201	91.489	1.88E-108	394	1	268	282	1
918362	4970510	100	3.42E-46	187	198	298	296	196

922244	4937546	91.971	2.67E-47	191	164	300	223	88
924940	4679482	100	5.77E-39	163	1	88	88	1
940747	4766168	82.581	1.62E-34	148	106	253	157	3
946208	4946515	93.122	6.63E-73	276	2	189	83	270
947145	1662622	93.617	2.03E-53	211	156	296	160	20
973436	5010626	91.011	1.21E-60	235	1	175	143	318
978251	4988413	78.261	3.5E-36	154	24	292	39	296
993947	4737694	100	2.8E-31	137	1	74	74	1
1003786	4962279	88.152	5.9E-63	243	33	243	1	202
1006958	5025138	95.703	5.15E-119	429	1	255	78	333
1008788	5012283	100	8.26E-107	388	41	250	1	210
1012801	3279155	90	3.8E-25	117	128	218	90	1
1023181	3974299	90.476	2.12E-23	111	215	298	1	84
1025093	4992483	86.76	7.03E-83	309	17	299	306	30
1030043	4908215	91.628	1.53E-74	281	84	298	271	74
1040159	4959049	100	4.27E-70	267	155	298	10	153
1091816	4679514	99.31	4.57E-69	263	107	251	1	145
1124302	3818692	88.889	9.82E-36	152	11	136	78	200
1131597	1280879	96.341	1.26E-30	135	3	84	151	70
1173982	4729808	86.854	2.11E-58	228	76	280	216	7
1243679	4735224	100	1.28E-35	152	1	82	82	1
1282458	4626402	100	2.77E-96	353	1	191	191	1
1291353	5076964	83.092	9.64E-46	185	4	208	280	75
1306289	4998853	86.415	1.3E-74	281	1	255	275	11

1318426	4954737	88.06	2.52E-82	307	1	268	257	1
1347628	2398347	96.97	1.76E-23	111	187	252	173	108
1394770	4698575	91.566	4.7E-59	230	5	170	200	35
1408062	5027445	98.438	2.26E-57	224	135	262	1	127
1408174	4813246	99.029	2.7E-101	370	53	258	248	43
1500699	4746871	91.589	4.38E-34	147	115	220	179	73
1509652	4986404	100	2.88E-56	220	140	258	1	119
1516997	5068067	85.909	4.56E-60	233	97	312	135	348
1593732	1689522	93.939	1.29E-64	248	88	252	183	20
1628703	2279256	94.512	1.07E-65	252	96	259	23	185
1654420	4990754	83.045	4.27E-70	267	20	296	307	22
1711378	1703260	100	2.62E-36	154	149	231	177	95
1784854	4734202	84.615	9.48E-52	206	4	222	229	22
1836447	4757494	88.136	4.4E-74	279	4	237	1	236
2009947	4727862	88.889	7.15E-68	259	1	215	20	229
2014881	1880953	86.765	1.04E-35	152	9	143	137	3
2156495	4827517	100	4.8E-29	130	1	70	152	221
4713597	4720454	87.838	1.2E-70	268	89	300	228	8
47865	3332825	91.414	1.54E-69	265	23	220	3	191
85869	4815345	100	3.16E-66	254	148	284	247	111
102407	2315176	92.958	2.63E-52	207	157	298	32	173
185113	4908621	96.512	7.51E-33	143	1	86	12	97
264716	3528847	92.537	1.02E-45	185	118	251	200	74
286416	3610782	93.939	4.05E-65	250	117	281	1	165

287638	4834692	99.197	3.66E-125	449	30	278	1	249
351671	4961062	94.406	1.21E-55	219	3	144	161	19
351961	4992563	82.156	6.67E-58	226	3	269	41	306
413755	1755898	84.138	1.3E-29	132	75	210	175	32
436585	4679190	100	1.63E-93	344	1	186	35	220
513688	4798912	90.123	7.73E-53	209	153	314	214	55
1058326	4665522	100	6.05E-33	143	1	77	77	1
2310697	1638582	93.056	2.04E-53	211	156	299	163	20
4209471	4651556	98.333	2.03E-53	211	179	298	203	84
4404171	2723987	93.5	1.5E-84	315	95	292	1	200

Table S6. The results of *blastn* alignment between the confirmed sex-linked loci of *A.* sp. and the Y chromosome of *A. mantzorum*

Sex-linked markers ID (*A.* sp.)	Y chromosome ID	Identical(%)	Evalue	Bitscore	Start (*A.* sp.)	End (*A.* sp.)	Start (Y chromosome)	End (Y chromosome)
46910	4842059	100	4.27E-70	267	1	144	159	16
87634	5022680	100	4.27E-70	267	1	144	90	233
103972	4554218	98.611	9.25E-67	255	1	144	181	38
208821	4975549	87.456	6.9E-93	342	7	286	287	1
244230	5026205	87.591	1.25E-35	152	5	138	132	1
306265	4979642	98.026	1.58E-69	265	1	152	98	249
476103	2542448	98.361	1.57E-54	215	8	129	200	79
476999	4750696	100	7.36E-48	193	195	298	176	73
497406	4801976	99.306	1.99E-68	261	155	298	231	88
654819	5013689	88.235	1.64E-44	182	1	153	235	85
1042367	4818549	91.011	3.52E-26	121	168	256	122	34
1399857	4606738	81.618	2.12E-23	111	155	289	35	170
1413441	4388296	94.558	7.55E-58	226	1	147	183	38
1417574	5064039	99.306	1.99E-68	261	155	298	25	168
209692	5041325	90.278	3.42E-46	187	155	298	299	157

284336	1298998	97.203	7.2E-63	243	1	143	179	37
121897	1342015	92.857	2.43E-57	224	126	279	18	171
374836	2782463	96.241	9.59E-56	219	109	241	35	167
1740912	4891784	92.275	2.75E-92	340	72	303	230	1
3408995	4890420	88.079	2.01E-43	178	9	159	150	1
4145899	4630072	95.302	4.96E-61	237	176	324	189	41
4533779	5064772	92.857	1.49E-63	244	1	168	269	102
5559010	1945796	87.302	2.51E-32	141	1	126	63	185

Table S7. The results of *blastn* alignment between the confirmed sex-linked loci of *A. tuberodepressus* and the Y chromosome of *A. mantzorum*

Sex-linked markers ID (*A. tuberodepressus*)	Y chromosome ID	Identical(%)	Evalue	Bitscore	Start (*A. tuberodepressus*)	End (*A. tuberodepressus*)	Start (Y chromosome)	End (Y chromosome)
12820	4605902	92.308	1.82E-78	294	58	262	209	2
120578	1263692	94.074	8.23E-52	206	79	213	135	1
131205	5037059	90.074	3.22E-91	337	43	299	348	80
145578	4503836	89.809	9.39E-51	202	50	206	9	165
157801	5068637	86.01	4.59E-50	200	23	215	87	270
234857	1524139	95.833	4.53E-60	233	1	144	174	31
291880	4991220	88.583	8.42E-82	305	18	270	1	251
312165	5008487	90.698	5.41E-24	113	1	85	255	170
312473	4841501	91.498	3.89E-90	333	42	281	255	10
334090	2727309	88.66	1.2E-60	235	43	236	1	193
448630	4588193	95.139	2.02E-58	228	1	144	150	7
476506	2261084	87.5	2.72E-27	124	1	112	95	200
898607	4980170	86.517	8.96E-47	189	109	282	183	10
915884	4906698	84.348	4.33E-60	233	2	219	36	265
1167728	2780333	91.608	1.92E-48	195	3	143	59	200
2016611	4657716	89.205	1.08E-56	222	155	330	176	1
2205008	5064031	95.238	4.52E-30	134	169	252	2	85
2895800	4747971	89.565	2.28E-77	291	42	271	234	7
3320173	1289776	95.238	5.61E-59	230	155	298	159	13

44236	4668816	89.655	4.57E-45	183	1	144	36	179
151493	4301340	96	4.17E-75	283	12	185	24	198
281120	5065425	94.495	4.14E-95	350	1	217	235	20
324456	1305761	95.238	4.52E-30	134	215	298	190	107
657724	4937294	82.857	1.2E-65	252	34	298	280	6
699597	4774882	81.696	3.13E-46	187	19	240	4	225
892878	5043309	79.57	9.45E-52	206	13	285	289	19
927772	1292191	95.604	5.08E-35	150	245	334	1	91
1190626	5059939	93.464	6.93E-58	226	1	152	139	291
1219551	5029678	87.97	2.68E-37	158	166	298	301	169
1223607	5070512	92.308	2.12E-23	111	1	78	306	383
1490677	4984216	84.81	1.45E-54	215	33	266	66	280
1546432	4921609	91.096	7.38E-48	193	155	299	114	254
1669022	4956423	90.909	3.17E-101	370	14	298	1	271
1932617	4928197	97.727	3.03E-81	303	1	176	104	279

Table S8. The results of *blastn* alignment between the confirmed sex-linked loci of *A. wuyiensis* and the Y chromosome of *A. mantzorum*

Sex-linked markers ID (*A. wuyiensis*)	Y chromosome ID	Identical(%)	Evalue	Bitscore	Start (*A. wuyiensis*)	End (*A. wuyiensis*)	Start Y chromosome	End Y chromosome
308740	4609812	86.916	3.52E-26	121	192	298	121	15
407109	5060375	82.386	1.18E-35	152	110	284	32	206
496223	1599208	79.375	3.31E-21	104	109	265	5	158
581766	5036611	91.538	2.06E-43	178	1	128	308	179
764303	4603686	88.034	3.5E-31	137	4	120	202	87
960052	1568656	86.364	4.18E-40	167	20	173	154	2
1146109	5076106	85.906	1.93E-88	327	1	298	111	401
2259651	4724876	93.75	3.35E-26	121	3	82	89	10
165357	4615220	93.151	2.74E-22	108	72	144	85	13

Table S9. The results of *blastn* alignment between the confirmed sex-linked loci of *A. xinduqiao* and the Y chromosome of *A. mantzorum*

Sex-linked markers ID (*A. xinduqiao*)	Y chromosome ID	Identical(%)	Evalue	Bitscore	Start (*A. xinduqiao*)	End (*A. xinduqiao*)	Start (Y chromosome)	End (Y chromosome)
9843	4901710	94.924	2.36E-82	307	3	198	70	265
21814	4015891	87.288	1.12E-30	135	5	122	132	15
34238	5024600	98.131	3.42E-46	187	34	140	3	109
42925	4845090	92.5	5E-24	113	1	80	65	143
49188	4816829	90.984	5.93E-88	326	18	257	1	244
161894	4571131	84.5	1.35E-49	198	25	224	205	6
174020	4925829	93.064	3.61E-65	250	73	245	209	40
177220	4848862	92.5	1.68E-58	228	1	160	243	86
185243	4986522	92.308	3.4E-51	204	155	297	297	155
205093	4703356	95.402	1.64E-73	278	1	174	17	190
235379	5081381	92.766	9.6E-91	335	24	252	496	262
248710	4962503	90.299	1.35E-94	348	1	268	20	283
261179	4771874	93.868	2.77E-86	320	1	212	28	239
270491	4981812	79.891	3.76E-30	134	32	213	287	104
276980	5008837	92.715	2.03E-53	211	1	144	109	259
291938	4551514	88.462	3.53E-26	121	1	100	93	194
295780	4385856	92.135	2.72E-27	124	45	133	1	88
305104	2084386	93.125	3.46E-60	233	86	243	200	41
315504	4675784	97.073	1.81E-93	344	80	283	1	205
317076	4842521	81.435	5.73E-44	180	65	298	253	29

322800	4869989	85.124	1.01E-25	119	53	171	162	46
327585	5062224	95.331	7.4E-112	405	2	258	376	123
335624	4969680	89.216	6.54E-28	126	159	260	161	261
337909	4581586	98.361	2.79E-86	320	81	261	206	24
377554	4561986	90.244	7.46E-38	159	177	298	204	82
381837	1525081	95.172	2.02E-58	228	155	298	194	50
383683	4987816	89.105	4.64E-84	313	1	257	26	275
413110	1546693	93.548	1.41E-44	182	148	267	125	2
427134	4984920	82.258	1.78E-53	211	20	265	16	261
441478	4916733	93.72	6E-83	309	1	207	20	225
451609	4780681	86.014	1.05E-35	152	52	194	219	79
500215	4929185	98.649	1.34E-29	132	145	218	87	14
543427	5002305	95.395	6.35E-63	243	115	266	107	258
546456	4929739	88.934	3.69E-80	300	21	264	243	2
548943	5056782	93.522	9.46E-101	368	6	252	329	83
548972	1374375	92.806	3.62E-50	200	112	250	159	22
558580	5043566	100	1.71E-23	111	1	60	357	298
581564	4947461	90.526	1.05E-65	252	54	243	1	190
621084	5047274	88.931	3.69E-85	316	12	266	6	267
660521	4920508	83.206	3.52E-26	121	1	131	163	33
669622	4723270	86.463	5.19E-59	230	12	217	229	1
676260	5065766	92.135	7.57E-28	126	3	91	413	325
708316	1762492	92.143	6.46E-48	193	1	135	24	163
710425	4898863	88.421	1.82E-58	228	82	271	260	73

717152	5001471	93.966	3.70E-95	350	3	233	83	314
743626	4679096	93.119	9.75E-86	318	35	250	1	218
761785	4872411	93.617	2.29E-72	274	46	229	185	1
773439	4940514	87.5	4.85E-64	246	51	263	20	234
784541	4906408	82.266	1.62E-39	165	104	301	1	190
793228	2312438	85.075	3.05E-31	137	109	242	62	195
795876	4934255	90.717	3.52E-85	316	17	253	1	237
803765	5049660	87.137	3.55E-70	267	18	251	102	342
825300	1627822	89.56	4.99E-59	230	31	210	200	19
827704	4936932	96.026	4.65E-64	246	103	253	242	92
846857	4955311	85.635	5.01E-44	180	11	180	104	284
848368	5043139	87.333	3.19E-96	353	1	297	53	349
855366	4970522	85.606	1.63E-29	132	14	144	260	135
878964	4806097	90.123	3.05E-21	104	146	226	64	143
914376	1520206	93.252	8.46E-62	239	24	186	1	162
932913	3173986	87.719	4.52E-30	134	29	142	200	87
942805	1391549	88.889	5.77E-39	163	1	133	163	31
953266	4623658	94.366	4.36E-55	217	155	295	23	164
963563	4728112	86.585	2.57E-67	257	18	263	230	1
970511	4429492	89.444	1.68E-58	228	74	253	5	184
988318	5078920	78.283	8.42E-27	122	30	224	352	547
990637	1270850	98.131	2.78E-46	187	1	107	133	27
1005114	4985608	94.631	4.76E-59	230	109	257	239	92
1014296	4214655	87.701	3.67E-55	217	68	254	194	9

1020825	4630785	88.966	2.66E-42	174	5	144	212	68
1034214	5068917	92.369	2.60E-96	353	1	248	369	122
1040632	5067274	85.714	1.05E-40	169	42	209	10	168
1043391	4555300	92.174	1.70E-38	161	136	249	179	65
1065118	1313313	90.164	2.13E-37	158	16	136	62	182
1072143	1512504	84.536	2.23E-47	191	62	255	1	193
1080984	4803217	84.906	3.55E-21	104	35	139	60	163
1087523	4983762	91.667	3.30E-71	270	95	298	1	191
1102583	4812800	94.495	3.43E-40	167	94	201	1	108
1133476	4694982	90.265	5.10E-34	147	1	112	102	214
1136078	4842587	85.398	4.86E-59	230	2	226	23	243
1145170	4748683	93.662	2.03E-53	211	1	140	11	152
1155734	5066716	87.931	3.50E-31	137	4	119	116	1
1166734	4921793	93.478	2.94E-31	137	1	92	40	131
1177553	2784233	89.72	1.10E-30	135	92	196	199	93
1178246	5013685	84.431	1.41E-39	165	2	168	196	30
1179969	4771322	91.946	6.26E-53	209	1	149	61	209
1183839	4839007	87.407	9.65E-37	156	155	289	79	213
1208794	1945860	86.301	9.65E-37	156	155	298	27	171
1231889	4902167	95.146	2.22E-87	324	1	205	217	13
1248360	1509474	96.503	3.36E-61	237	155	297	1	143
1258317	4588515	93.701	2.26E-47	191	131	257	198	72
1266544	5069844	95.139	2.02E-58	228	155	298	313	170
1266715	4959813	97.674	5.81E-34	147	213	298	103	19

1284717	4995260	91.954	7.53E-62	239	1	169	92	265
1288168	4909358	82.09	3.37E-56	220	1	238	6	273
1299985	5043975	90.336	3.28E-80	300	1	238	333	110
1326745	4908920	86.486	3.82E-60	233	2	212	42	262
1333912	4646212	94.382	3.11E-31	137	1	89	127	215
1336205	5049142	98.592	2.32E-27	124	1	70	227	157
1339449	4975032	86.784	6.17E-63	243	34	249	298	72
1341381	4942437	91.837	1.44E-29	132	24	115	111	208
1353865	4924945	86.64	5.86E-68	259	1	231	32	278
1371474	1156831	98.78	5.00E-34	147	1	82	83	2
1415038	4568434	95.035	3.37E-56	220	155	295	140	1
1420641	5054431	86.726	6.51E-28	126	2	114	4	116
1443239	5076621	87.2	3.56E-75	283	7	254	266	18
1448966	4560084	93.431	2.91E-51	204	116	252	157	21
1451328	5038666	90.041	4.36E-84	313	3	243	340	100
1464357	4653746	95.62	2.87E-56	220	117	253	203	67
1489231	5027091	83.65	6.35E-63	243	3	263	75	334
1500334	5055282	89.764	6.01E-88	326	1	254	354	101
1508739	4857797	95.652	3.76E-45	183	140	254	146	259
1516483	2545396	91.667	4.94E-54	213	109	264	158	7
1521070	5036503	98.113	1.68E-73	278	100	258	207	49
1580001	4940270	89.695	3.63E-90	333	5	264	271	10
1603325	4830151	93.182	2.10E-28	128	155	242	86	1
1656418	2777109	86.607	1.10E-25	119	2	111	110	3

1667951	1776400	92.784	2.79E-32	141	164	260	123	27
1734850	2519818	86.331	4.49E-35	150	161	298	196	58
1735045	4976424	93.197	5.65E-54	213	155	298	243	97
1793947	4992361	90.816	1.45E-29	132	164	261	120	217
1811491	5036641	89.865	5.26E-104	379	3	298	3	287
1838720	4978568	81.94	2.59E-62	241	3	298	290	8
1870037	5080200	95.349	2.99E-31	137	1	86	340	425
1959643	4947980	83.39	7.10E-73	276	1	291	281	1
1962342	4959399	97.902	5.57E-64	246	156	298	138	278
1969981	5064175	87.129	2.34E-57	224	72	270	410	212
1989698	5075918	85.993	3.21E-91	337	1	297	385	81
2061010	4563417	91.111	3.16E-26	121	11	99	90	1
2068662	1298254	98.473	1.38E-59	231	136	266	183	53
2126331	1470994	91.489	6.47E-68	259	66	253	195	8
2137370	1355381	87.912	2.24E-22	108	80	170	98	8
2190627	4804480	97.531	9.72E-32	139	1	81	230	150
2244384	4898525	88.288	5.23E-29	130	159	269	255	148
2312767	1855526	86.62	5.23E-34	147	66	197	197	58
2488300	3574289	90.71	6.43E-63	243	35	217	182	1
2506788	4902217	79.817	6.52E-33	143	44	260	38	239
2526984	1293865	97.222	8.31E-27	122	2	73	34	105
2613323	1208385	98.824	1.06E-35	152	1	85	126	42
2751317	5036943	91.473	2.74E-96	353	4	260	57	313
2797506	4637267	85.638	2.31E-47	191	29	208	1	188

3035877	5035957	98.148	3.90E-75	283	40	201	3	164
106254	962596	97.436	1.63E-29	132	221	298	91	15
254508	4630910	94.928	3.58E-55	217	43	180	1	138
339039	1311273	87.421	3.74E-45	183	97	255	3	161
376827	4682031	87.121	5.81E-34	147	155	284	191	62
385579	1741112	85.401	6.22E-33	143	115	251	24	160
393996	4123971	83.465	1.27E-25	119	172	298	1	127
546907	1580739	96.97	3.61E-40	167	3	101	99	1
547855	5064880	87.059	8.47E-47	189	71	239	328	161
580884	4996701	90.604	6.57E-48	193	121	269	65	208
679159	4733278	91.176	1.81E-58	228	103	269	13	182
694936	4861473	91.096	5.69E-49	196	1	144	244	99
701190	4683779	93.171	3.45E-80	300	1	205	18	221
1006204	5008788	80.556	1.08E-50	202	1	267	312	25
1320534	5071644	96	1.69E-38	161	147	246	157	59
2022064	1615582	91.25	7.42E-23	110	2	81	80	1
2107800	4774566	91.463	5.89E-24	113	62	143	57	138
2688021	4628819	96.226	1.24E-94	348	20	231	212	1
