# Supplementary material for: Heterogeneous Evolution of Sex Chromosomes in the Torrent Frog Genus Amolops
Source: Int J Mol Sci. 2022 Sep 22;23(19):11146. doi: 10.3390/ijms231911146 (PMC9570394; doi:10.3390/ijms231911146)

File S7

Maximum-likelihood phylogenies of single sex-linked marker and its cross-species homologous sequence in *Amolops* (CH for *A. chunganensis,* JI for *A. jinjiangensis*, LI for *A. lifanensis*, LO for *A. loloensis*, MA for *A. mantzorum*, SP for *A. sp.*, TU for *A. tuberodepressus*, WU for *A. wuyiensis*, XI for *A. xinduqiao*). Bootstrap values are shown for the main branches, when above 50%. Sequence labels feature species, ID number, sex and allele. The trees are constructed using RAxML from a phased male and female sequences. Clades of Y sequences are shown in blue. Clades of X sequences are shown in red. All other sequences from males and females sequences are shown in black.

***A. Chunganensis:* Clocus_697780**

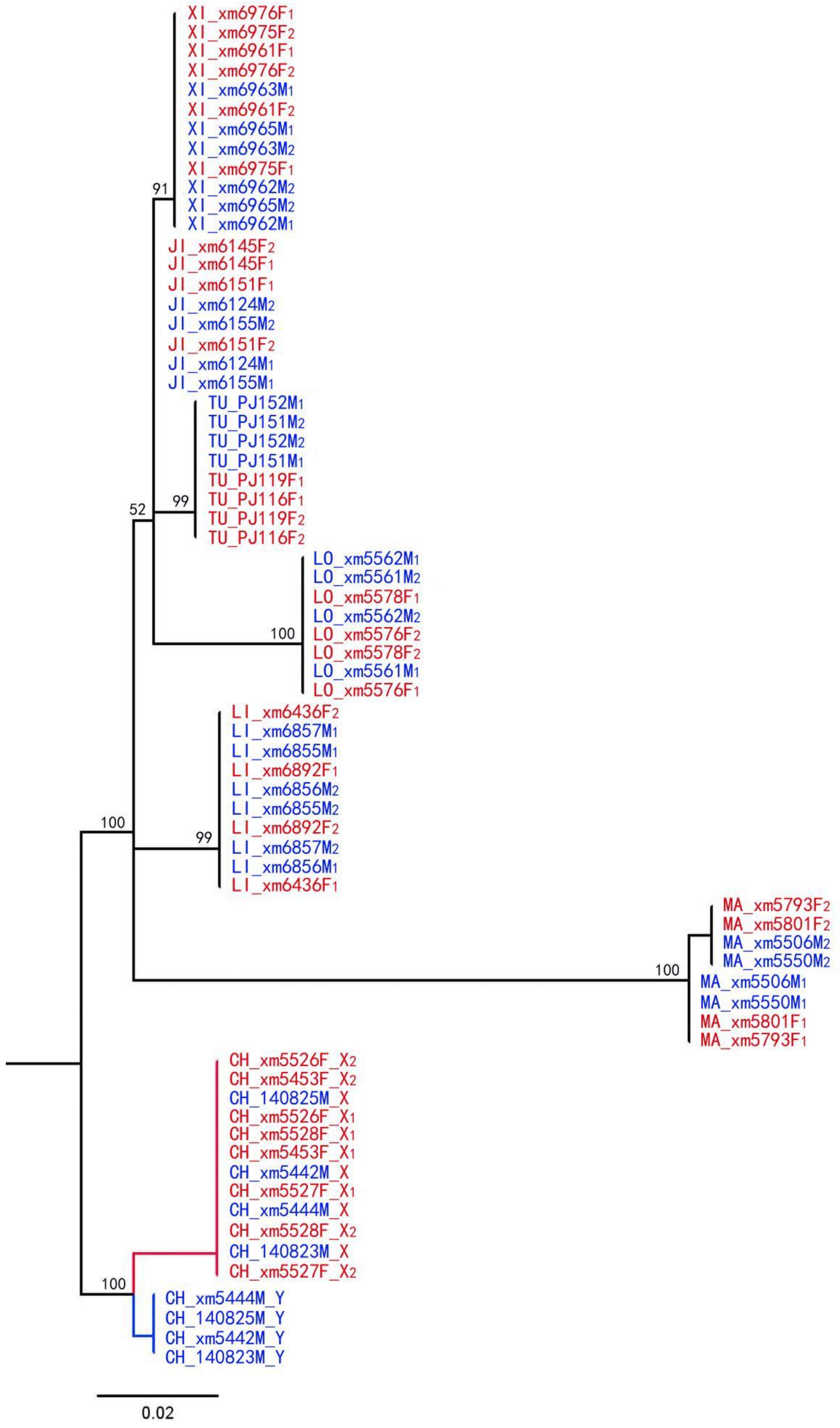

A. Chunganensis: **Clocus_2189594**

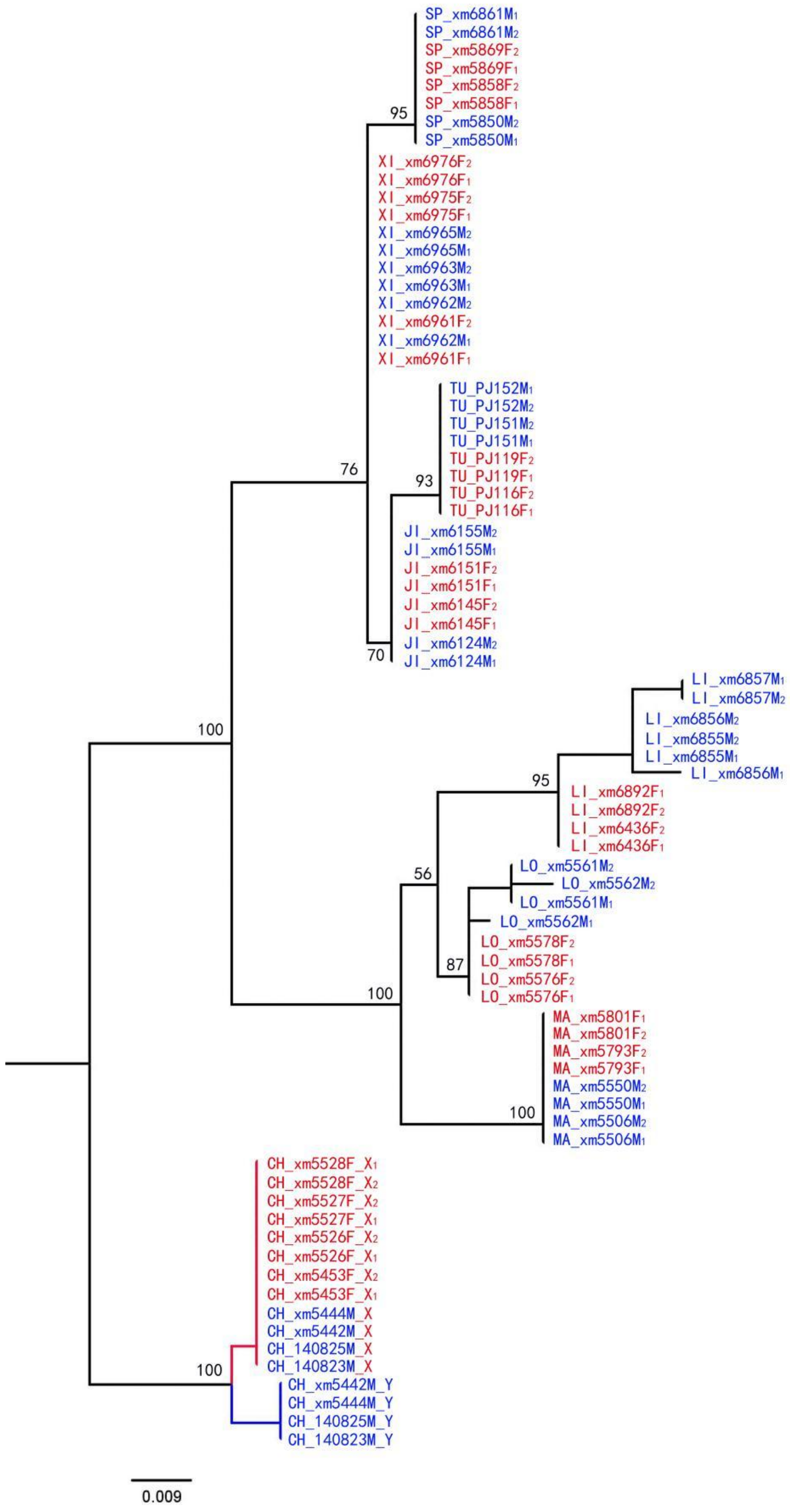

***A. jinjiangensis:* Clocus_94572**

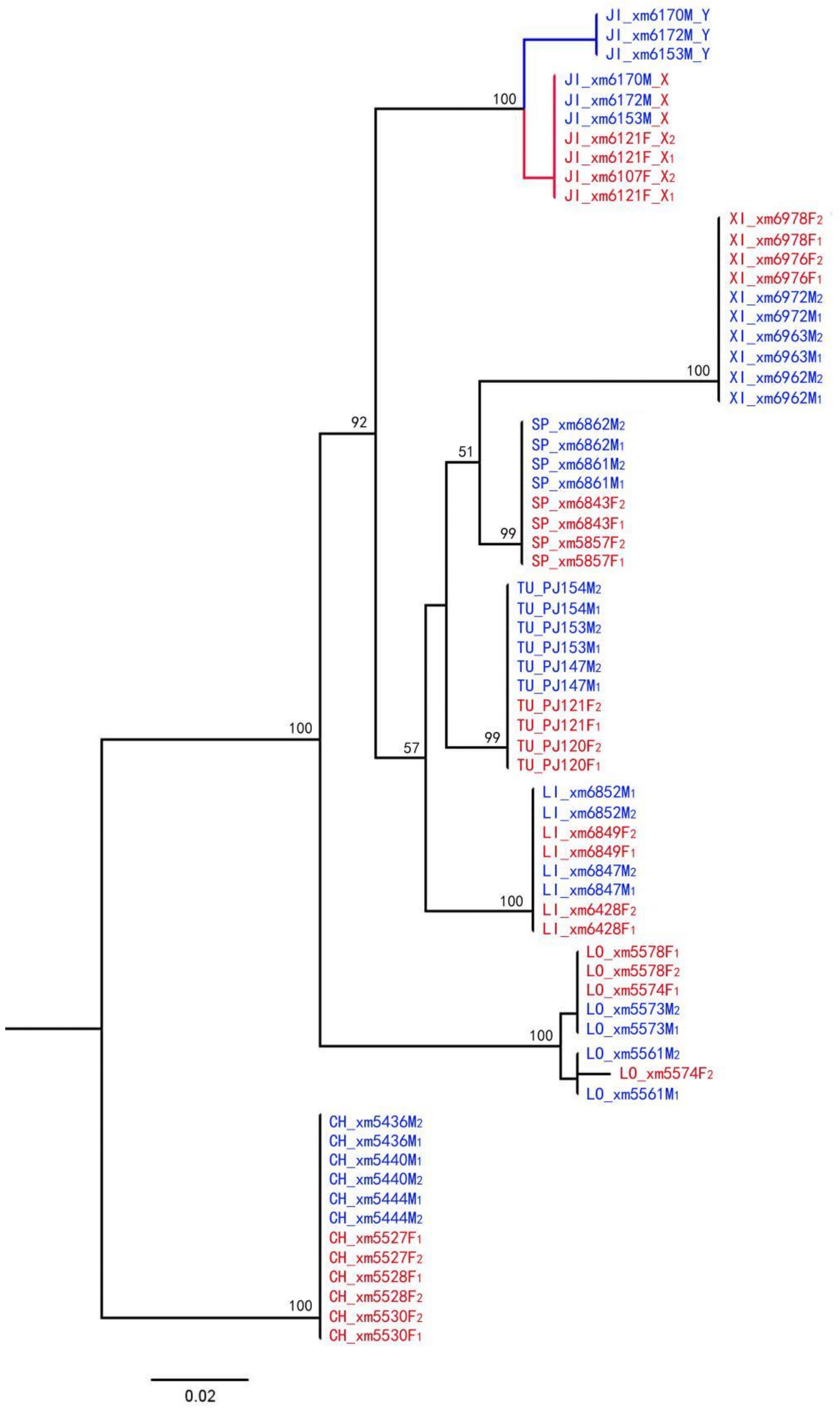

A. jinjiangensis: **Clocus_263214**

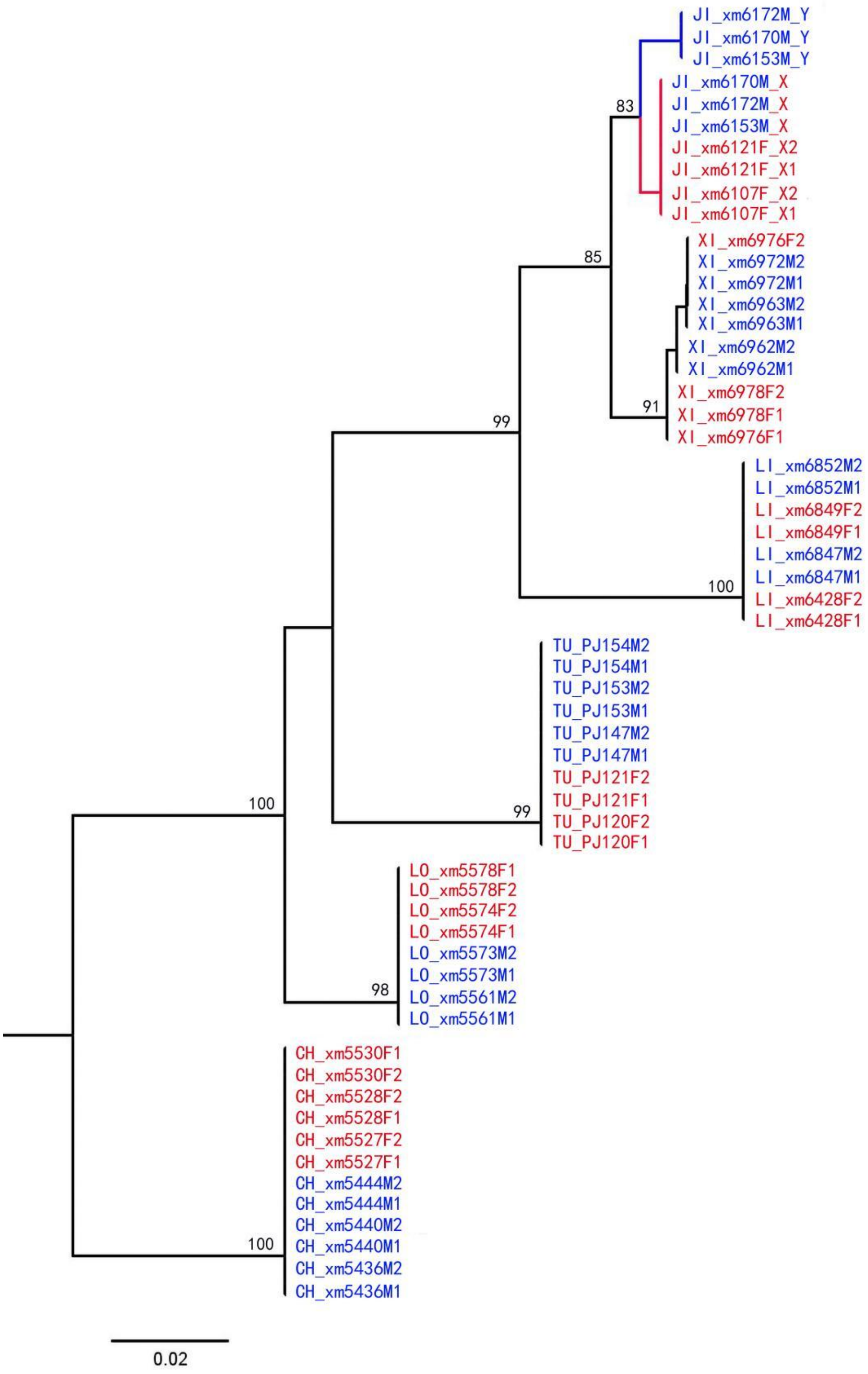

A. lifanensis: Clocus_409308

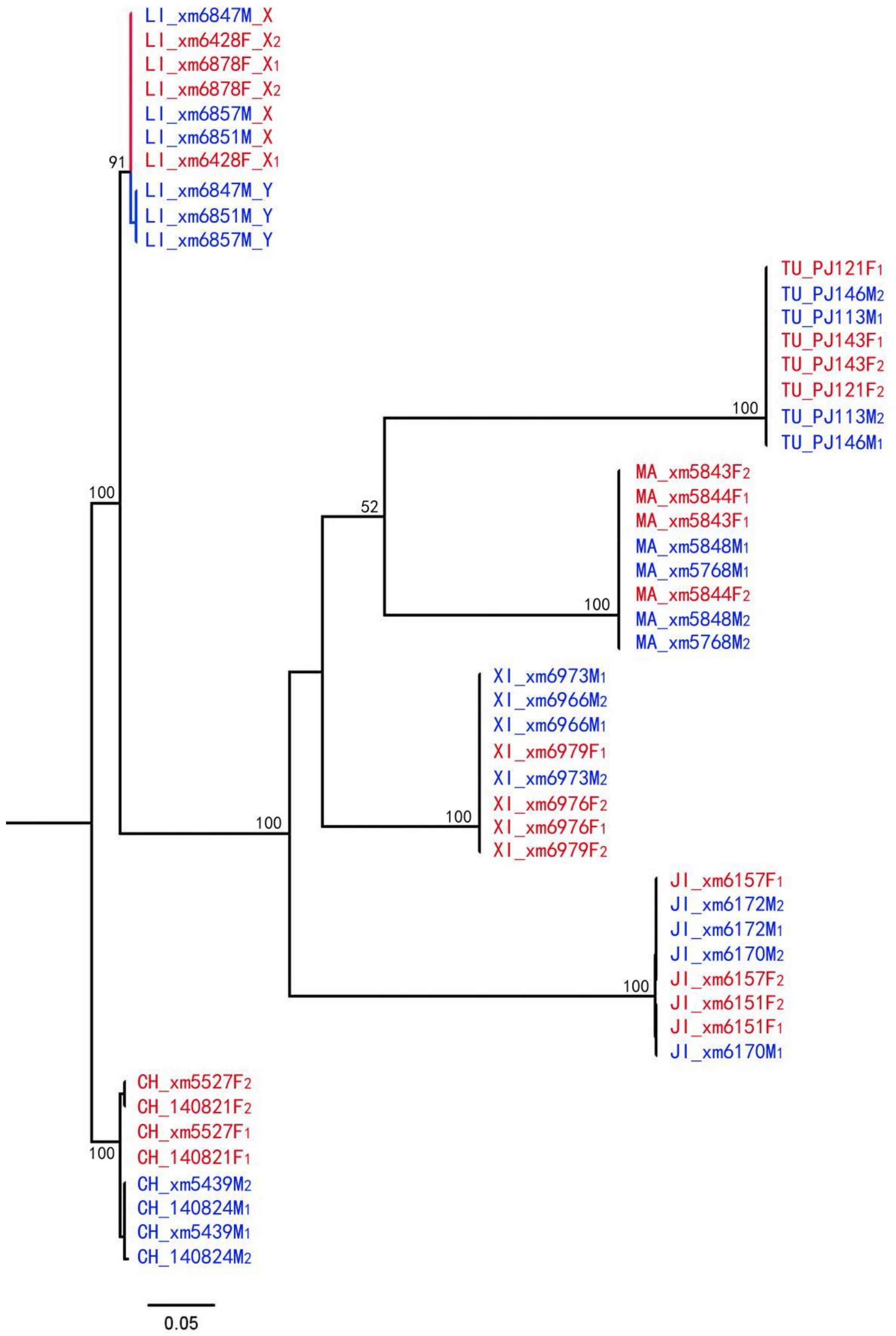

***A. lifanensis*: Clocus_520205**

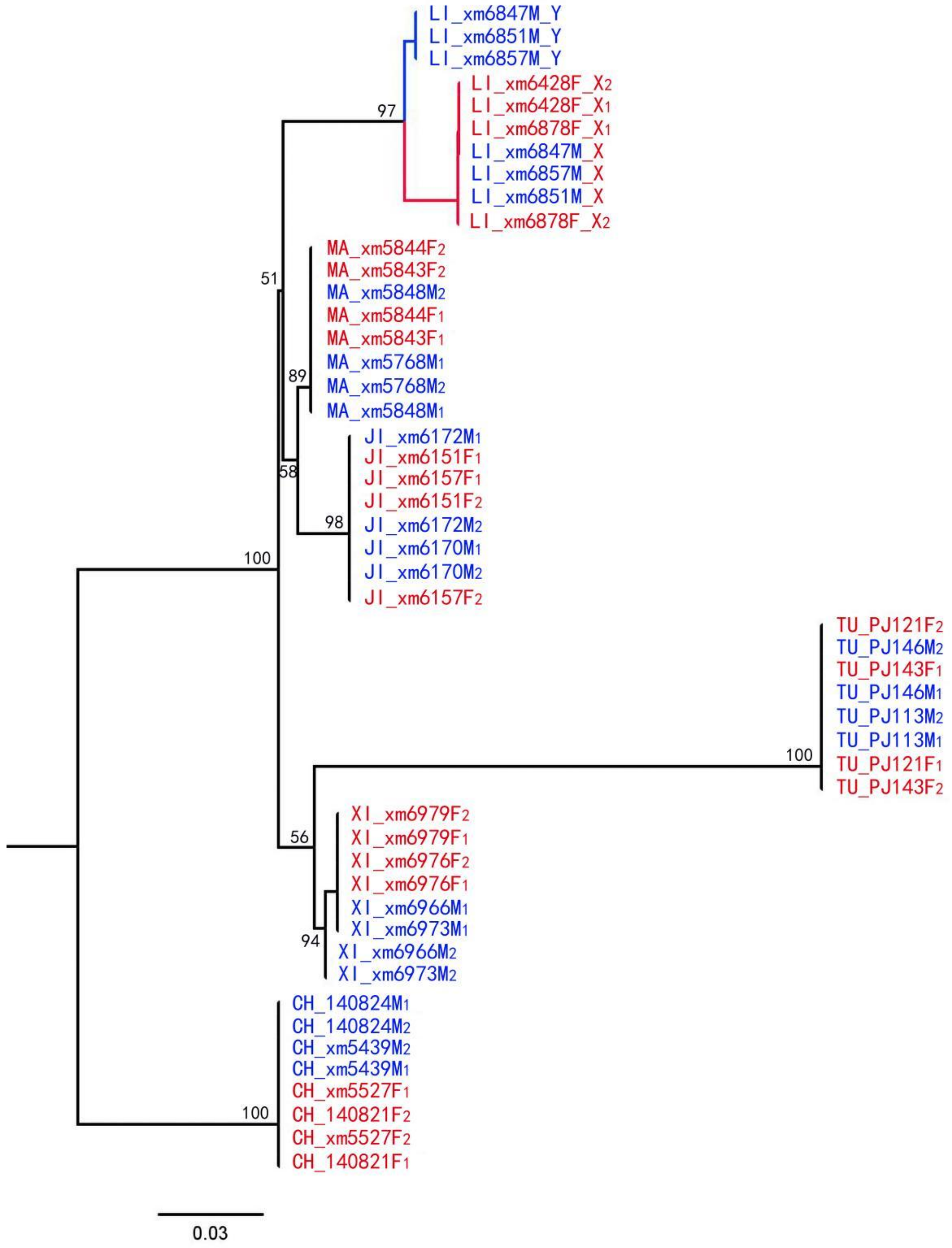

***A. loloensis*: Clocus_108264**

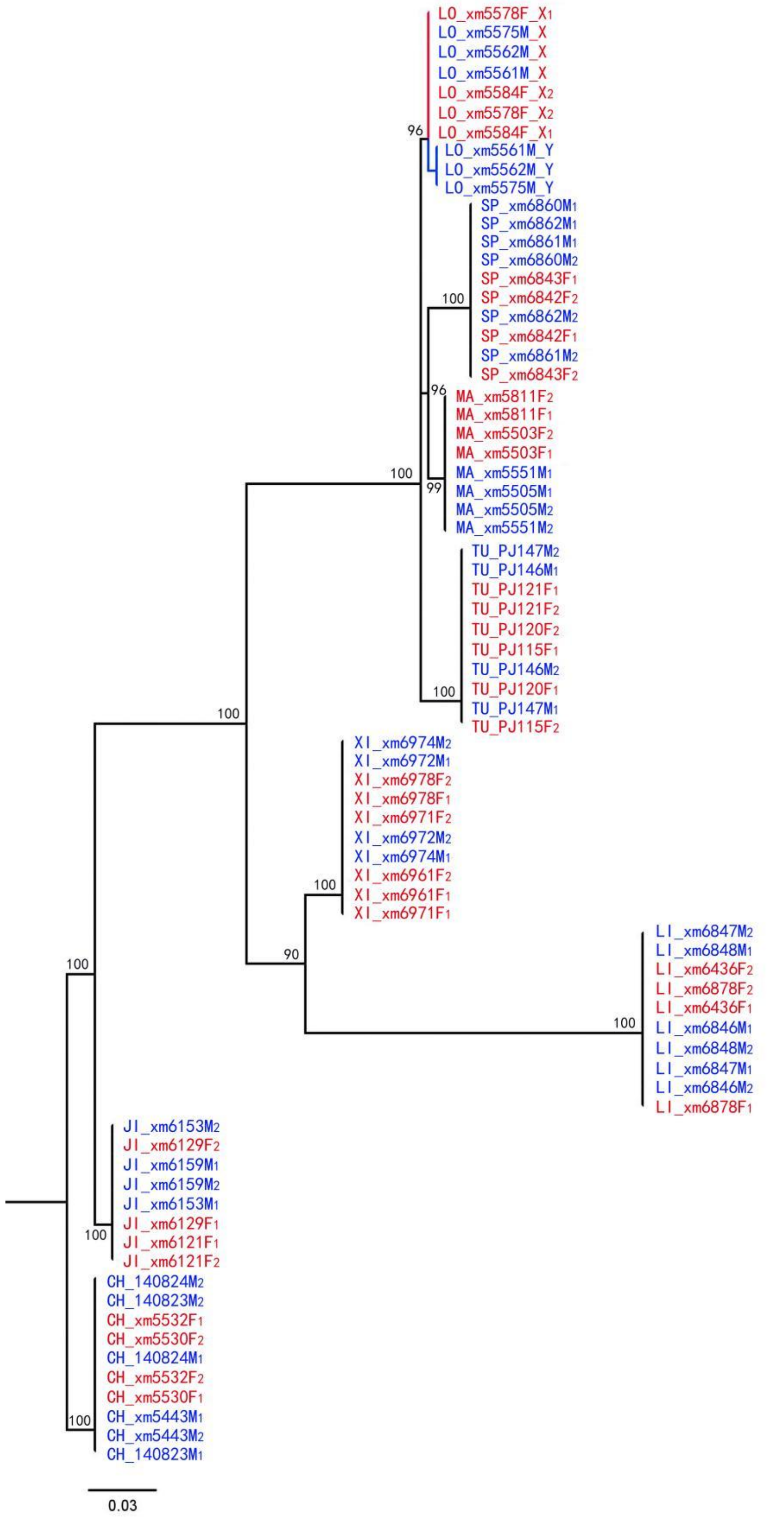

A. loloensis: **Clocus_771638**

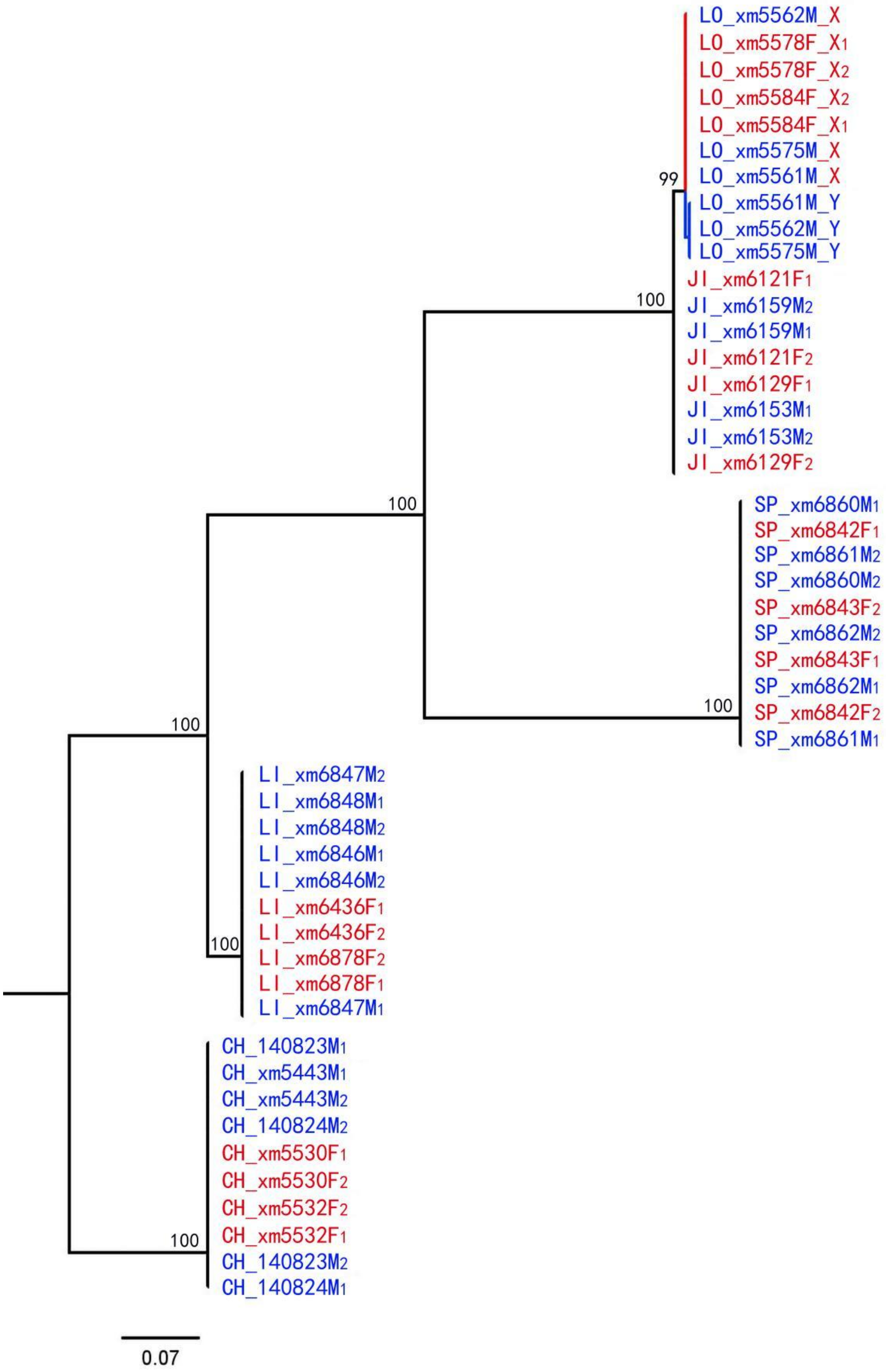

***A. mantzorum*: Clocus_240963**

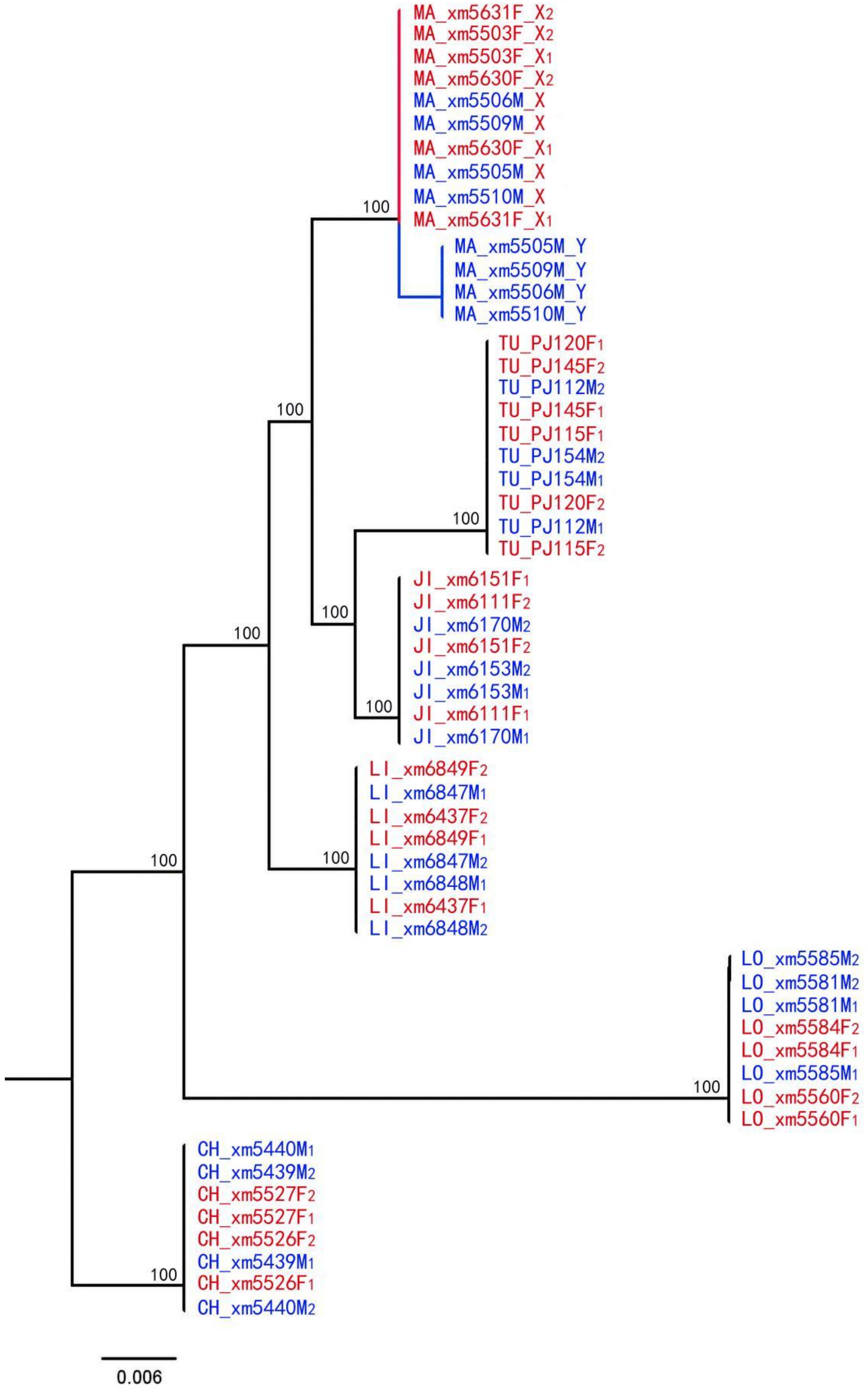

***A. mantzorum*: Clocus_650096**

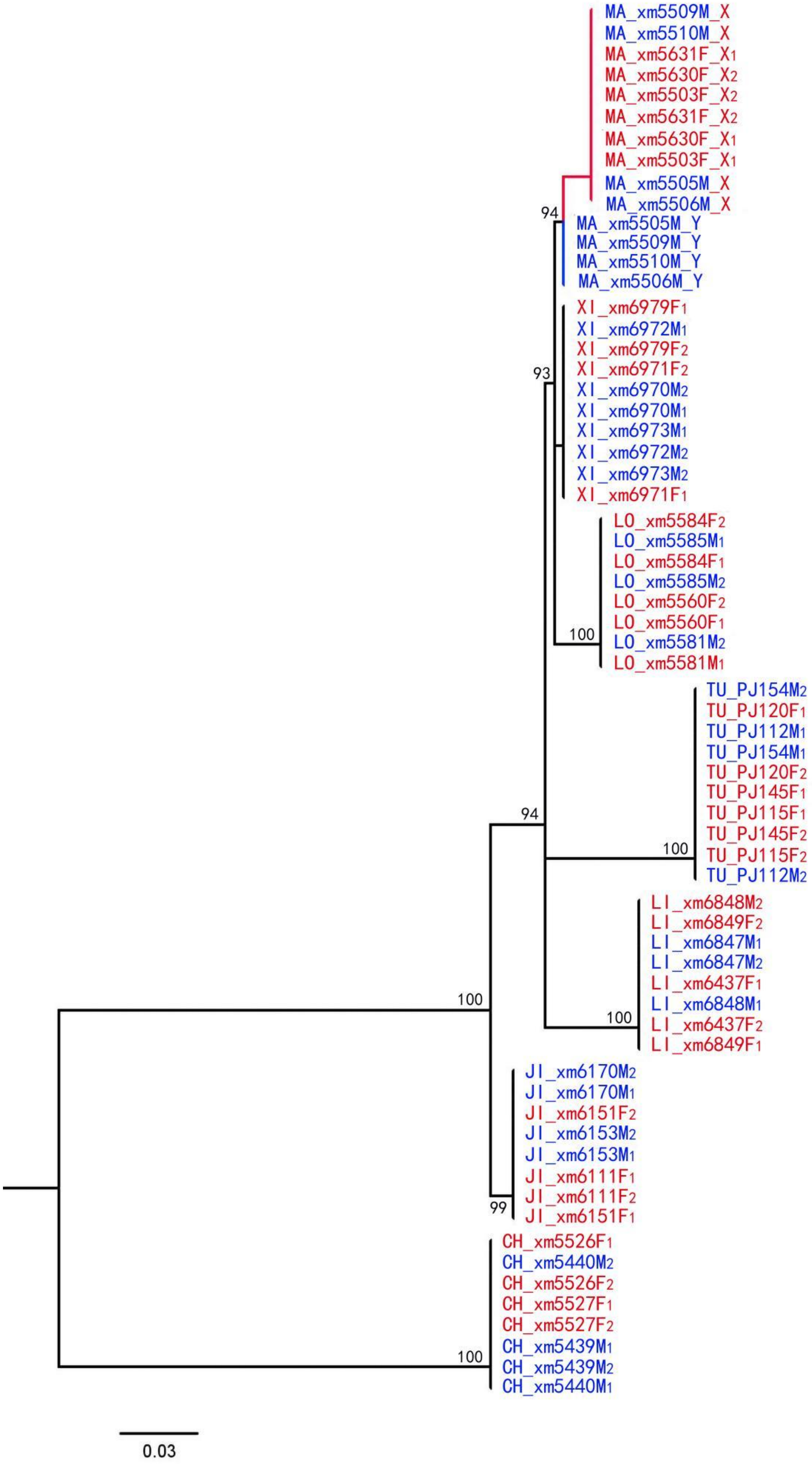

***A.* sp.: Clocus_46910**

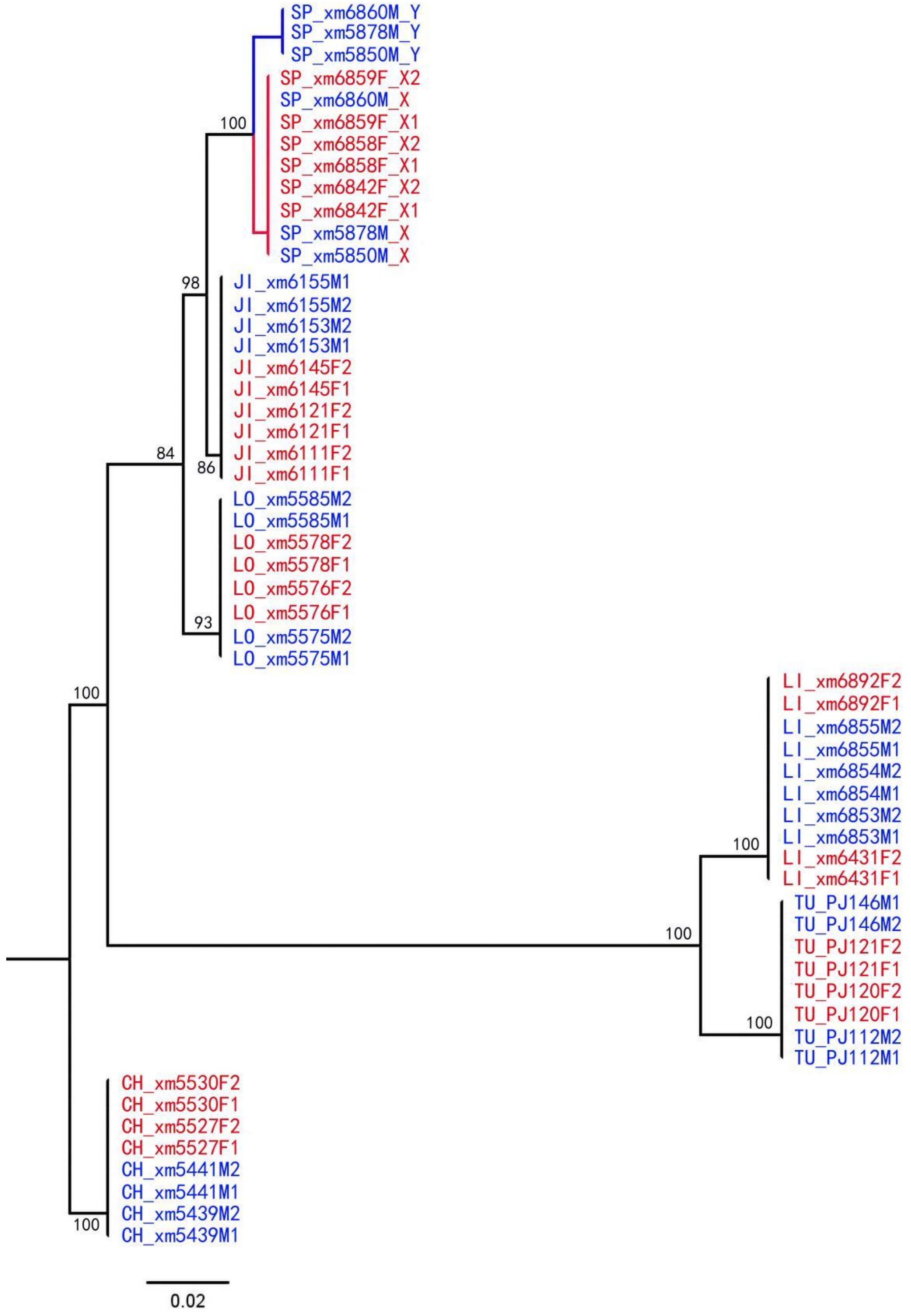

***A*. sp.: Clocus_271488**

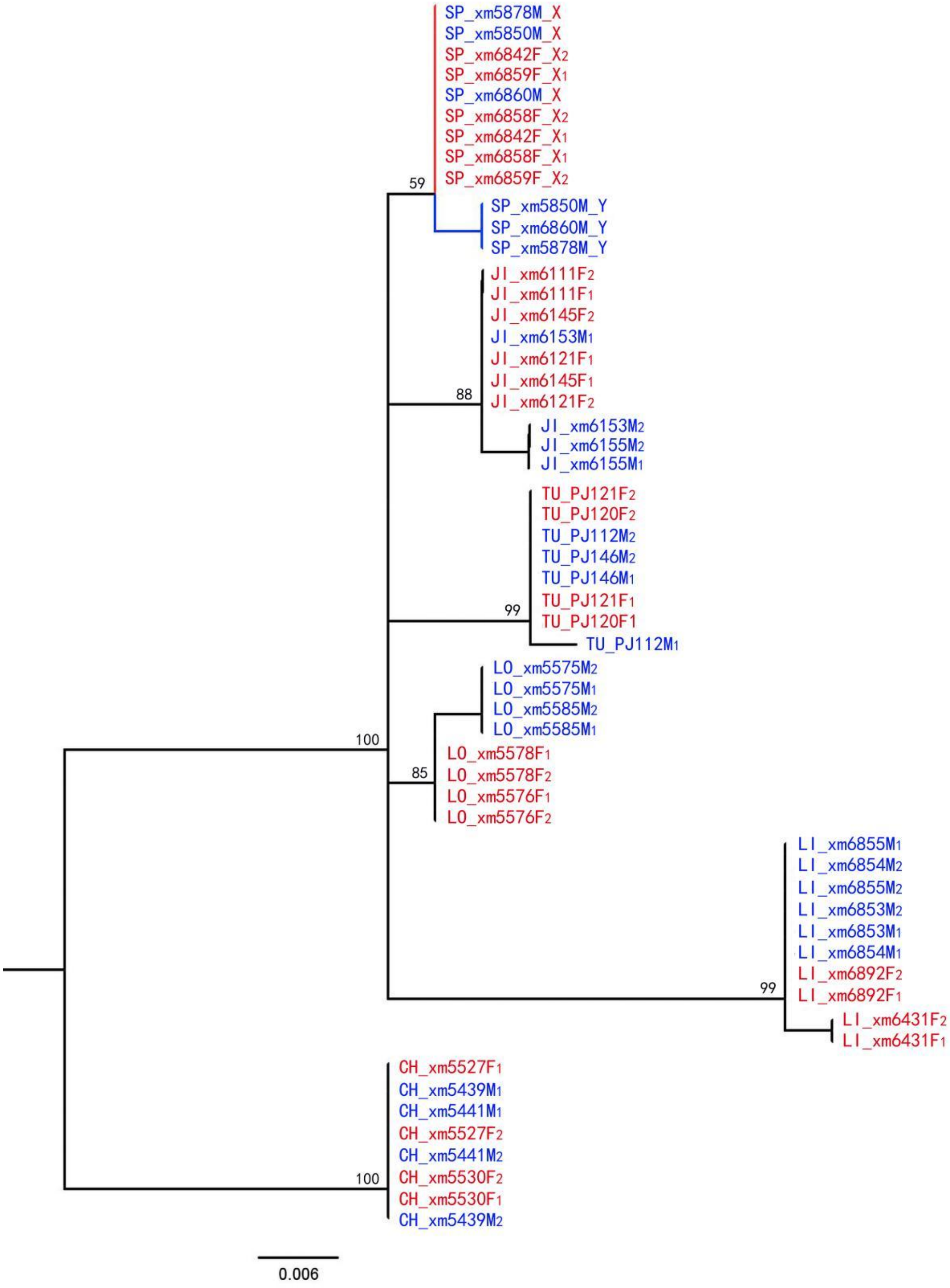

***A. tuberodepressus*: Clocus_312165**

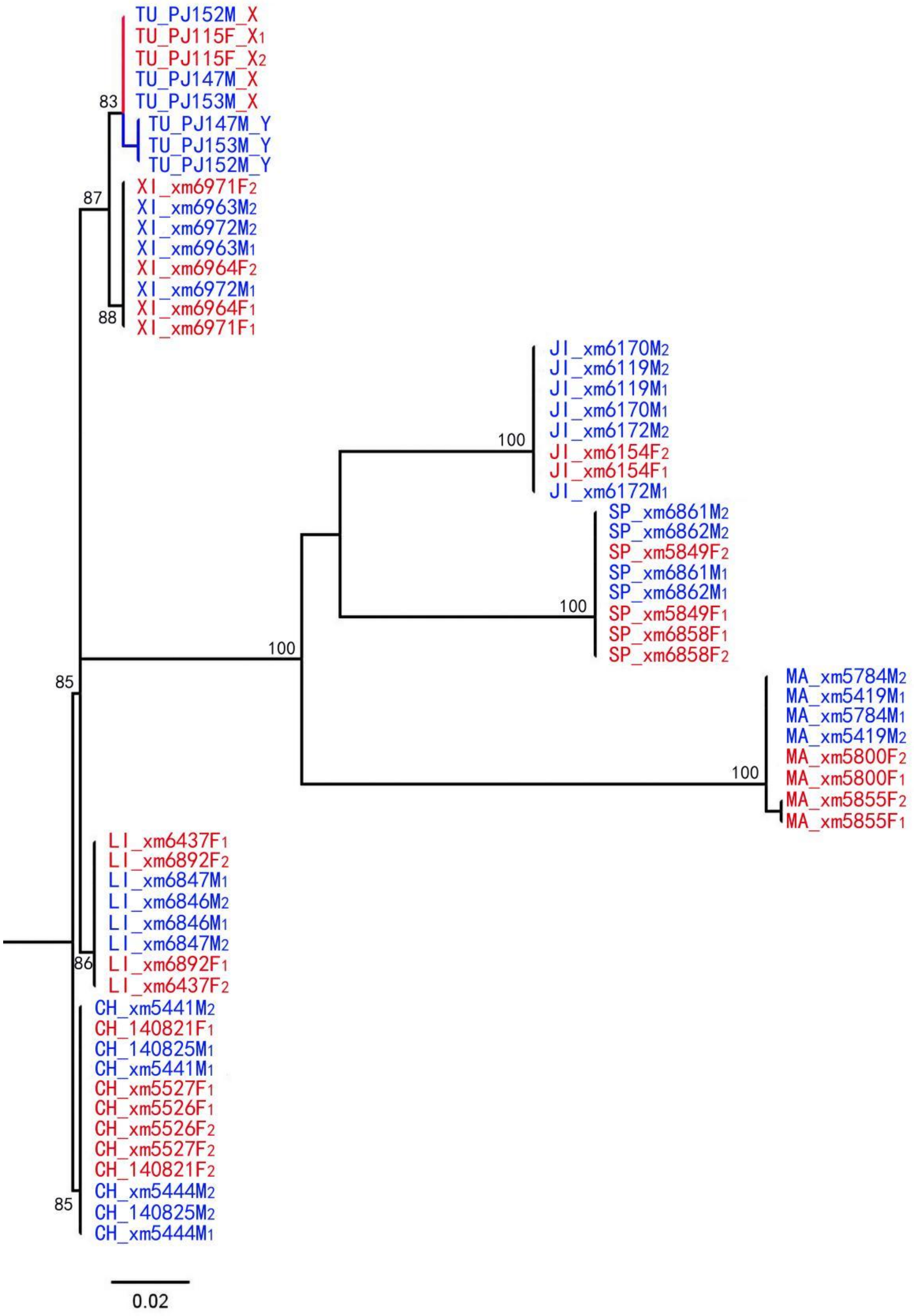

***A. tuberodepressus*: Clocus_657724**

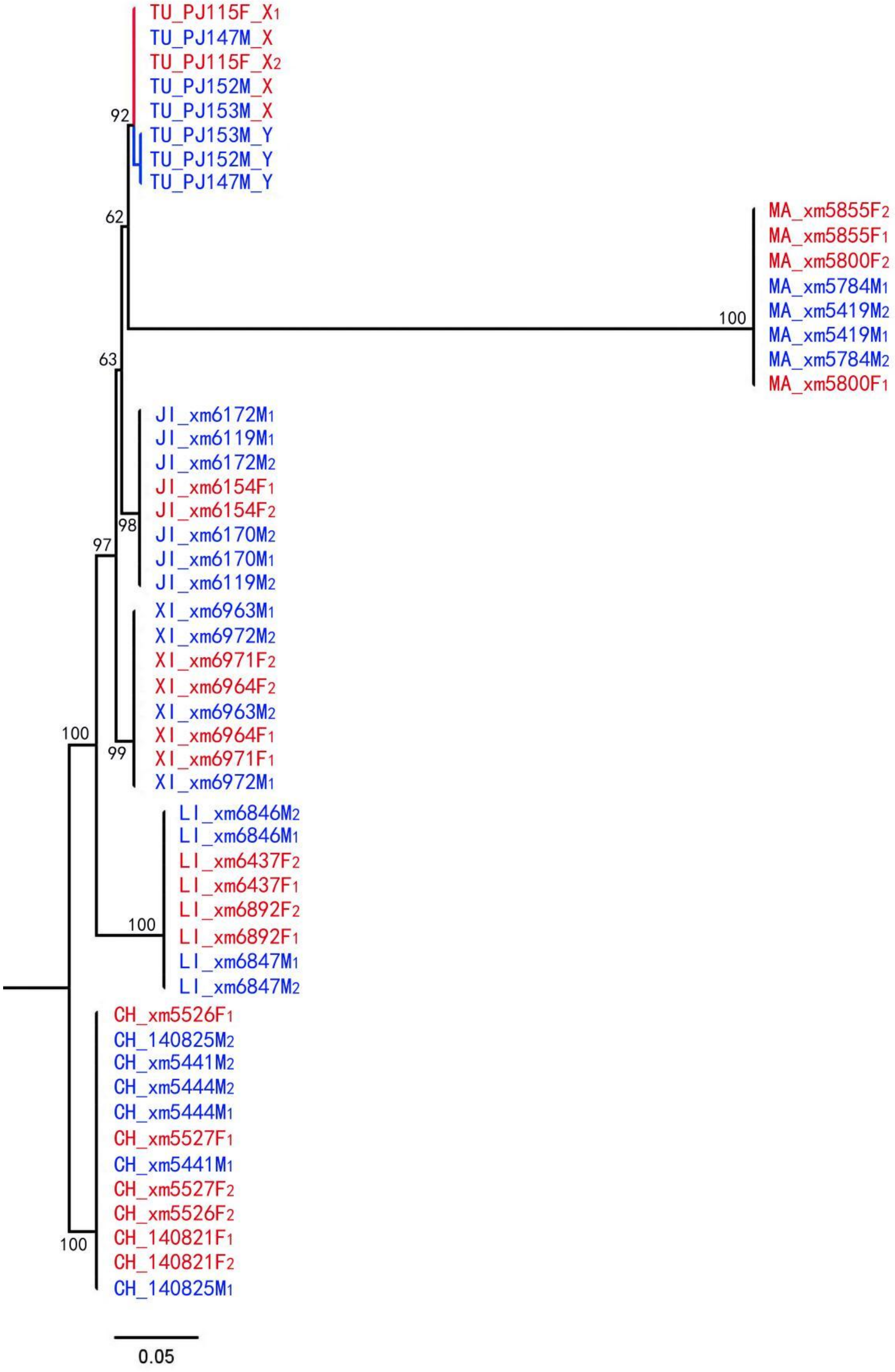

***A. tuberodepressus*: Clocus_934386**

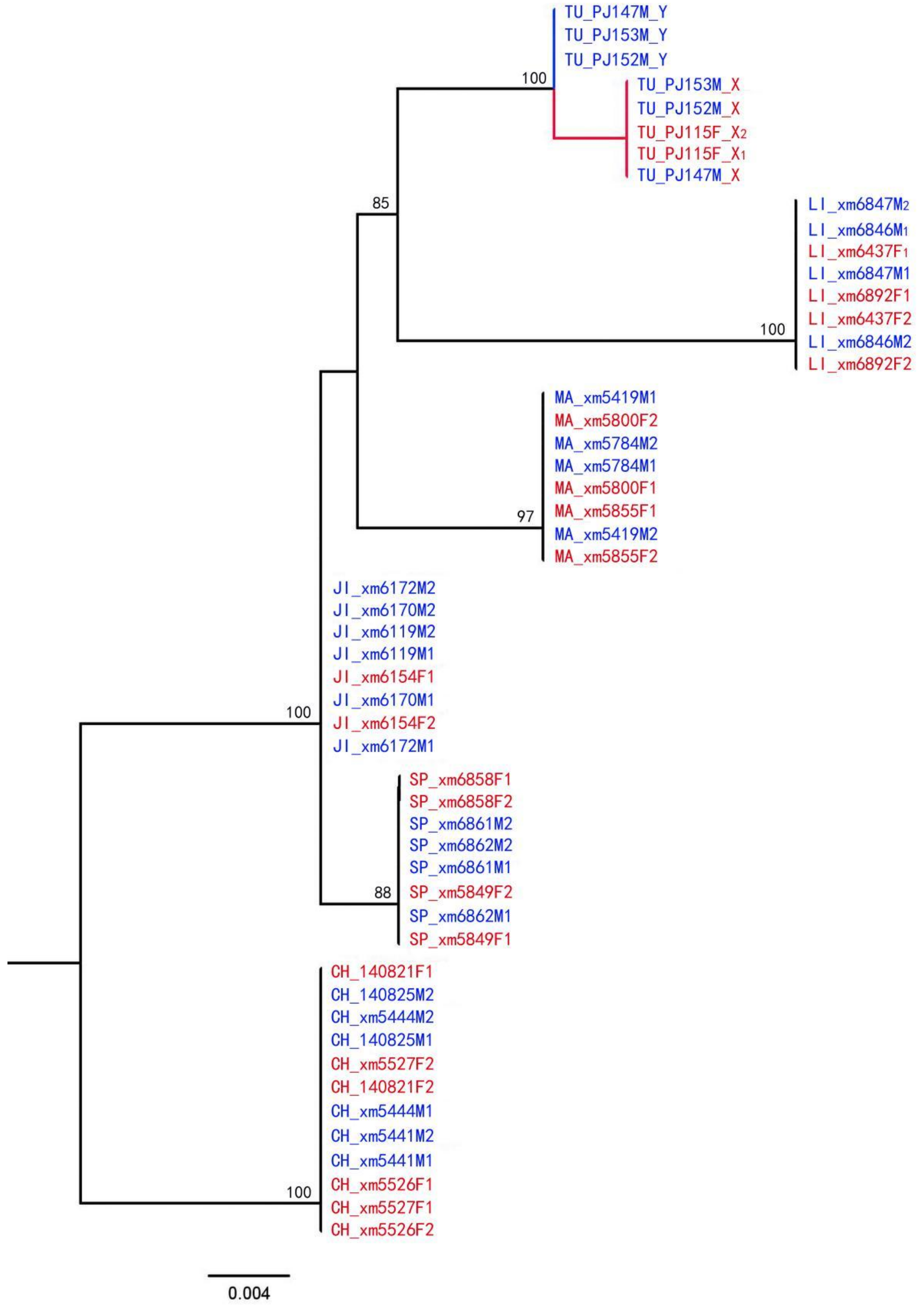

***A. wuyiensis*: Clocus_201111**

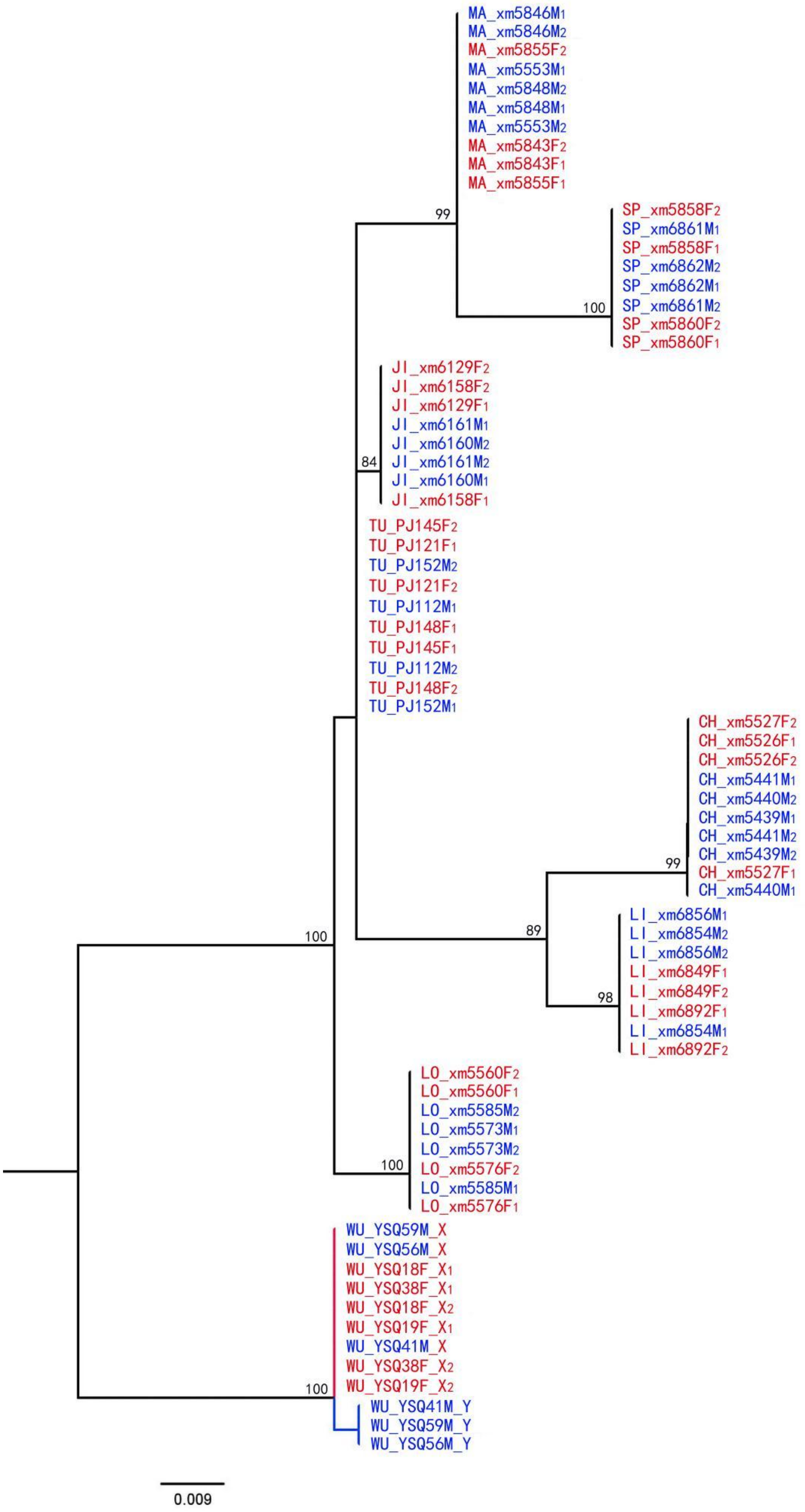

A. wuyiensis: Clocus_312243

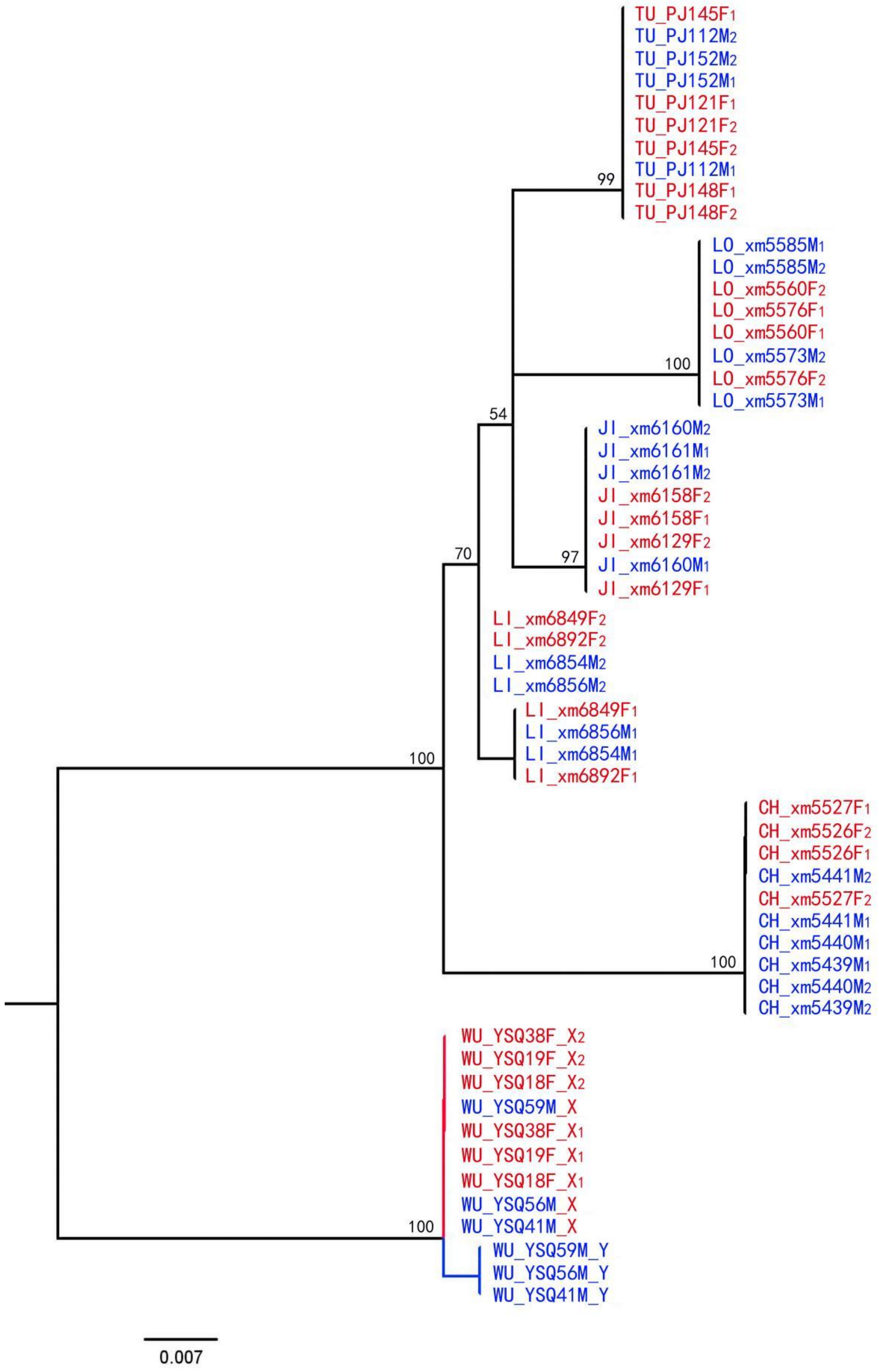

***A. xinduqiao*: Clocus_1829008**

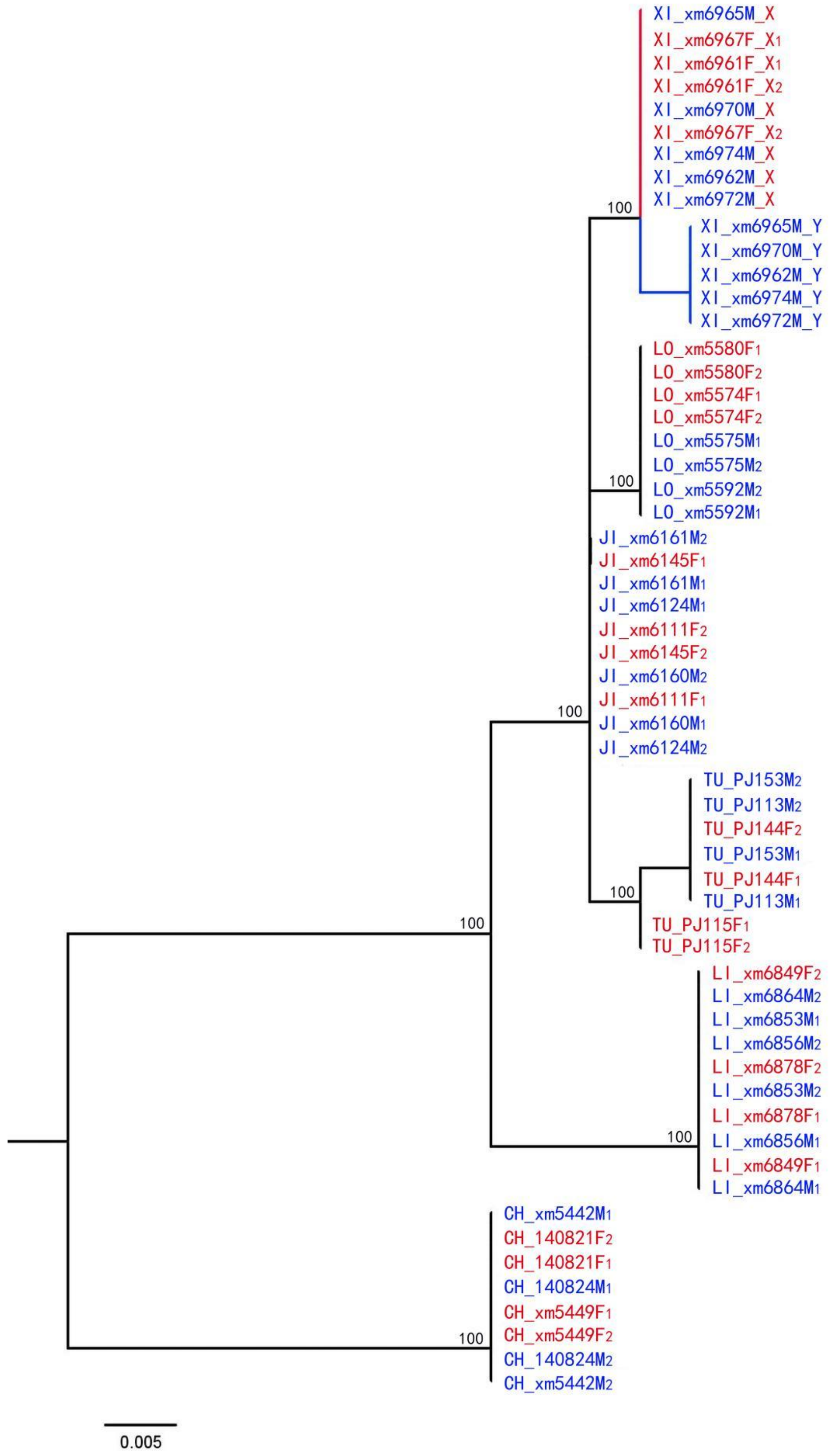

***A. xinduqiao*: Clocus_2190627**

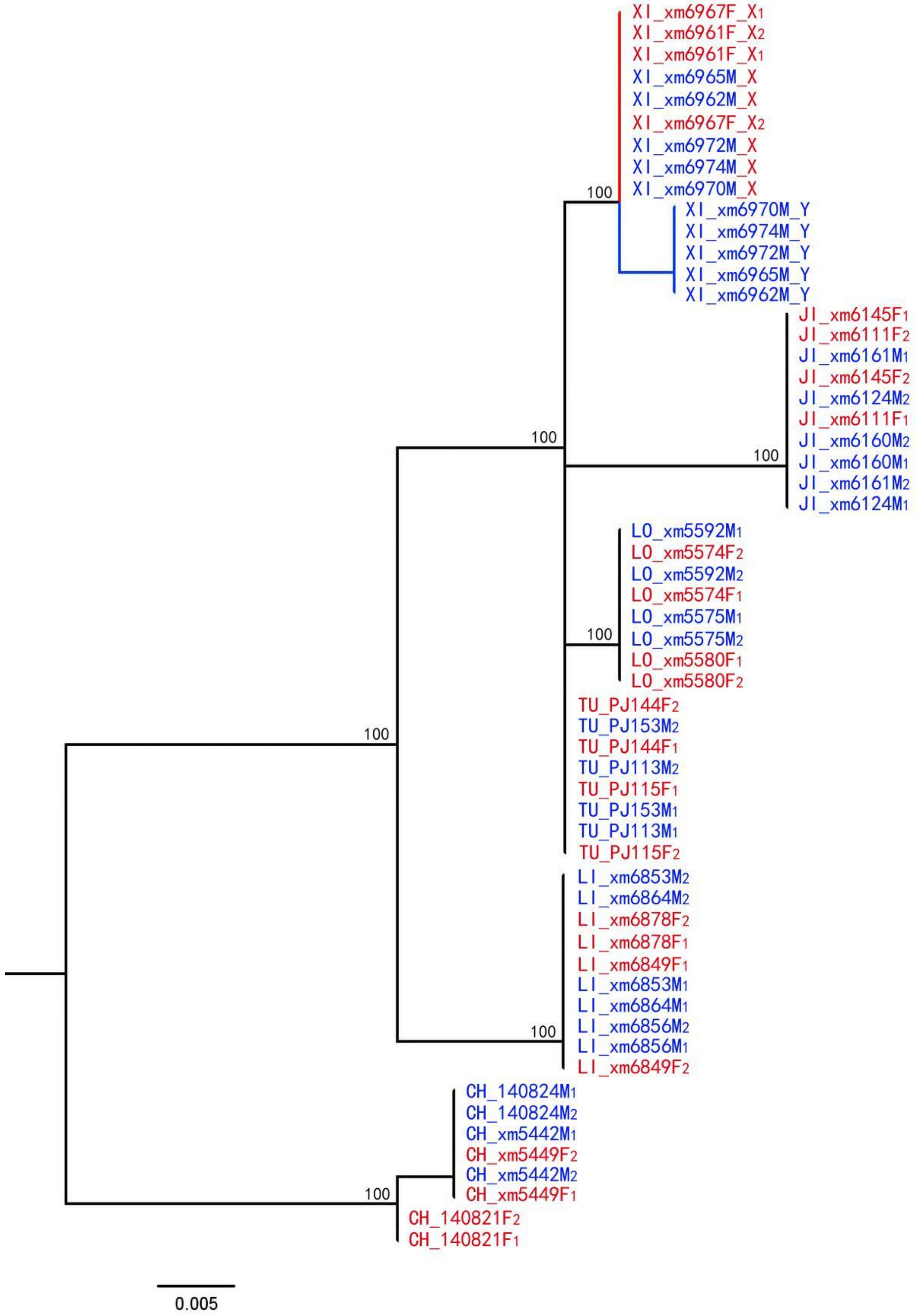

Supplement: Supplementary file 1 [file ijms-23-11146-s001.zip › ijms-1904191-manuscript-supplementary-revised/manuscript-supplementary/File S7.pdf]
