# Supplementary material for: Heterogeneous Evolution of Sex Chromosomes in the Torrent Frog Genus Amolops
Source: Int J Mol Sci. 2022 Sep 22;23(19):11146. doi: 10.3390/ijms231911146 (PMC9570394; doi:10.3390/ijms231911146)
Supplement: Supplementary file 1 [file ijms-23-11146-s001.zip › ijms-1904191-manuscript-supplementary-revised/manuscript-supplementary/File S8.pdf]

File S8

Maximum-likelihood phylogenies of five sex-linked markers and its cross-species homologous sequence in *Amolops* (JI for *A. jinjiangensis*, LI for *A. lifanensis*, LO for *A. loloensis*, MA for *A. mantzorum*, SP for *A. sp.*, TU for *A. tuberodepressus*, WU for *A. wuyiensis*, XI for *A. xinduqiao*). Bootstrap values are shown for the main branches, when above 50%. Sequence labels feature species, ID number, sex and allele. The trees are constructed using RAxML from a phased male and female sequences. Clades of Y sequences are shown in blue. Clades of X sequences are shown in red. All other sequences from males and females sequences are shown in black.

A. jinjiangensis

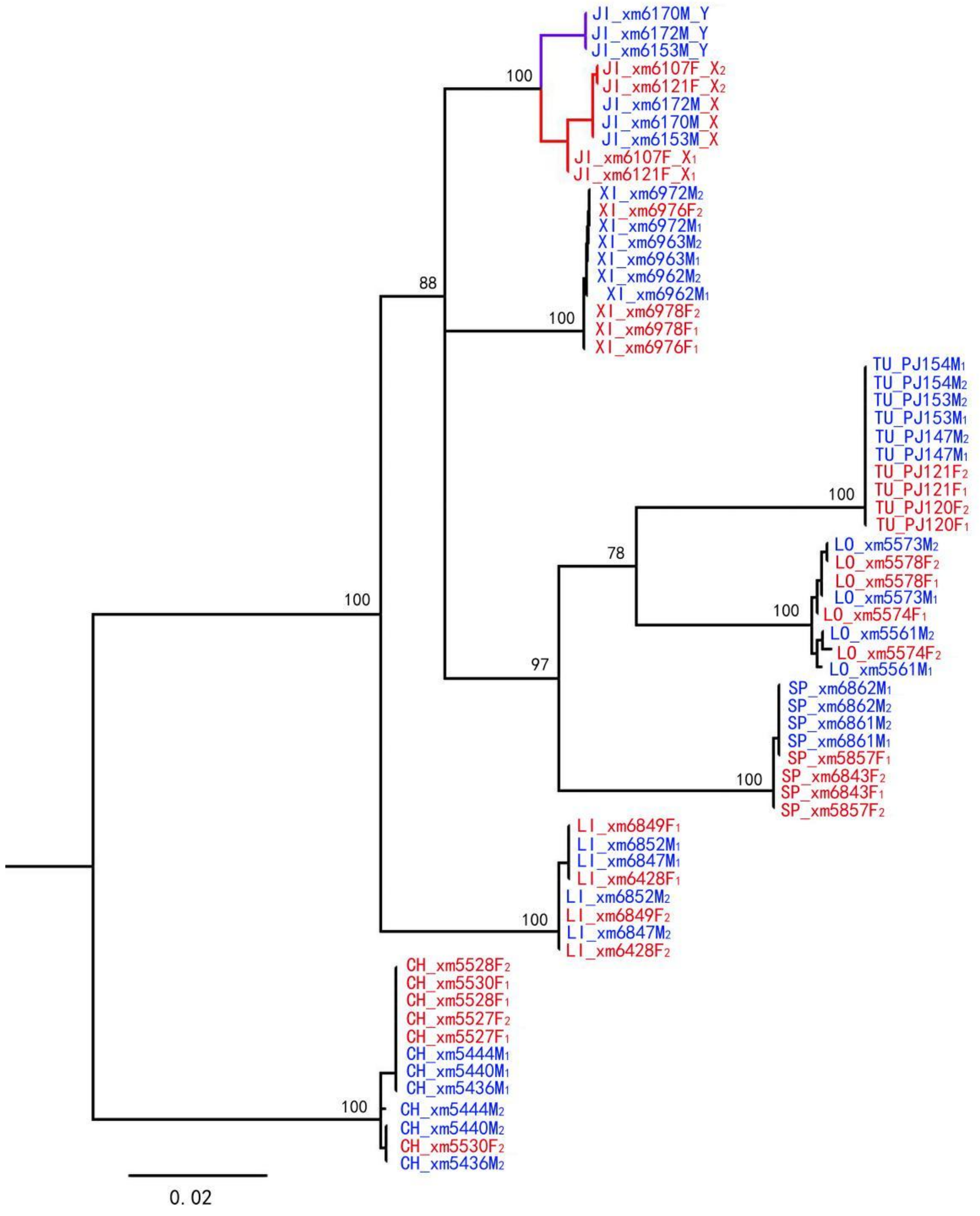

A. lifanensis

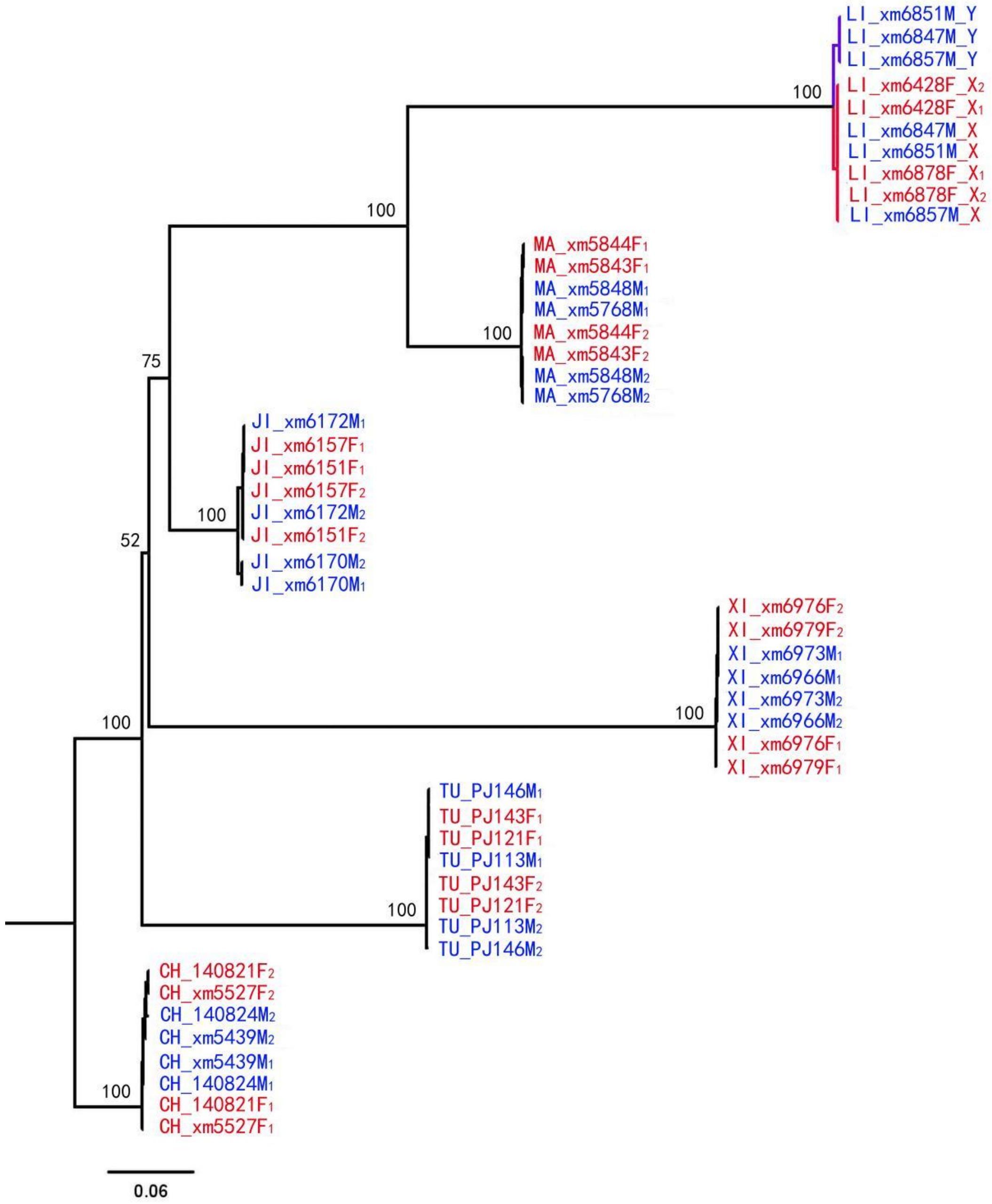

A. loloensis

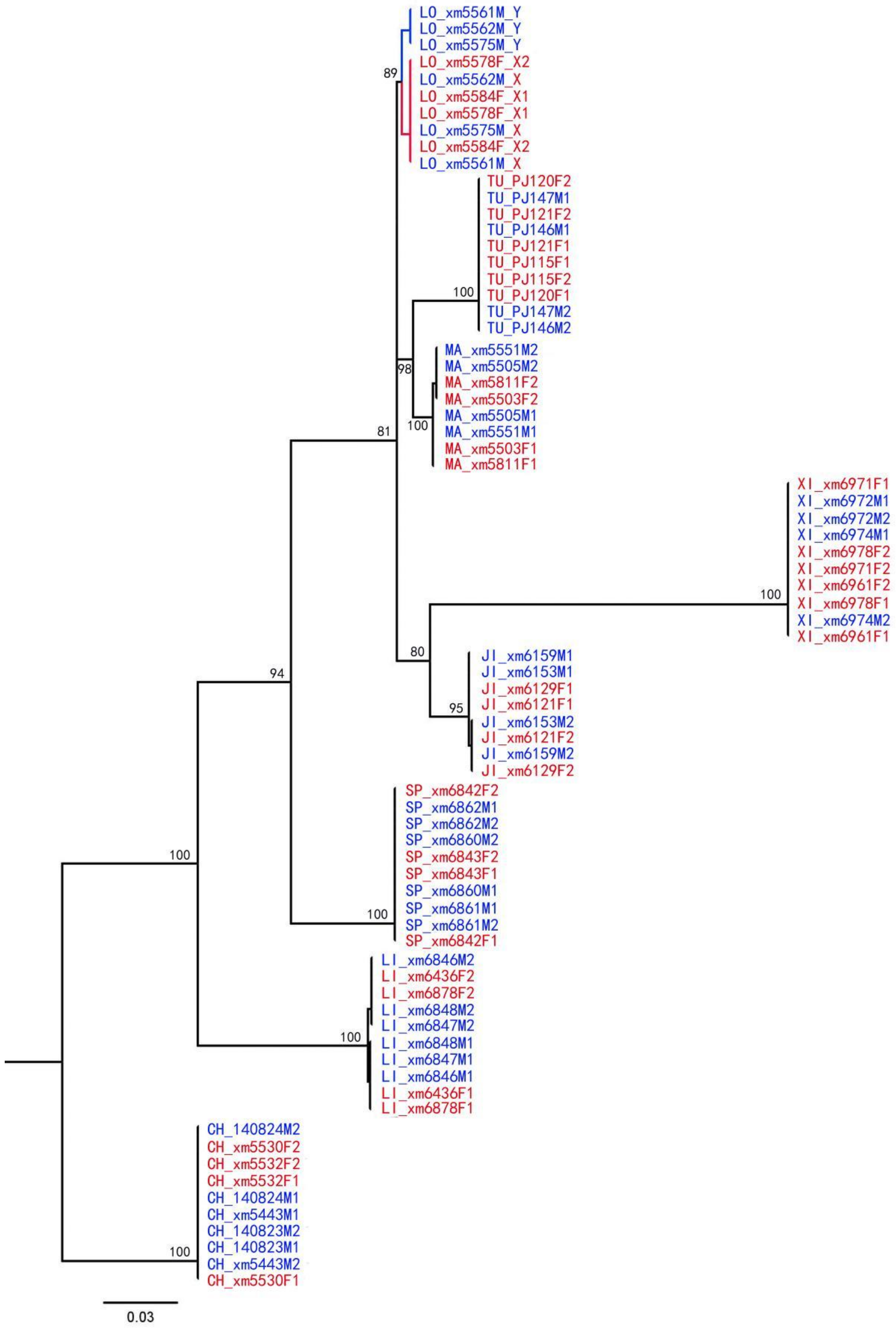
LO_xm5561M_Y
LO_xm5562M_Y
LO_xm5575M_Y
LO_xm5578F_X2
LO_xm5562M_X
LO_xm5584F_X1
LO_xm5578F_X1
LO_xm5575M_X
LO_xm5584F_X2
LO_xm5561M_X
89
TU_PJ120F2
TU_PJ147M1
TU_PJ121F2
TU_PJ146M1
TU_PJ121F1
TU_PJ115F1
TU_PJ115F2
TU_PJ120F1
TU_PJ147M2
TU_PJ146M2
100
MA_xm5551M2
MA_xm5505M2
MA_xm5811F2
MA_xm5503F2
MA_xm5505M1
MA_xm5551M1
MA_xm5503F1
MA_xm5811F1
98
100
81
XI_xm6971F1
XI_xm6972M1
XI_xm6972M2
XI_xm6974M1
XI_xm6978F2
XI_xm6971F2
XI_xm6961F2
XI_xm6978F1
XI_xm6974M2
XI_xm6961F1
100
80
JI_xm6159M1
JI_xm6153M1
JI_xm6129F1
JI_xm6121F1
JI_xm6153M2
JI_xm6121F2
JI_xm6159M2
JI_xm6129F2
95
94
SP_xm6842F2
SP_xm6862M1
SP_xm6862M2
SP_xm6860M2
SP_xm6843F2
SP_xm6843F1
SP_xm6860M1
SP_xm6861M1
SP_xm6861M2
SP_xm6842F1
100
100
LI_xm6846M2
LI_xm6436F2
LI_xm6878F2
LI_xm6848M2
LI_xm6847M2
LI_xm6848M1
LI_xm6847M1
LI_xm6846M1
LI_xm6436F1
LI_xm6878F1
100
CH_140824M2
CH_xm5530F2
CH_xm5532F2
CH_xm5532F1
CH_140824M1
CH_xm5443M1
CH_140823M2
CH_140823M1
CH_xm5443M2
CH_xm5530F1
100
0.03

A. mantzorum

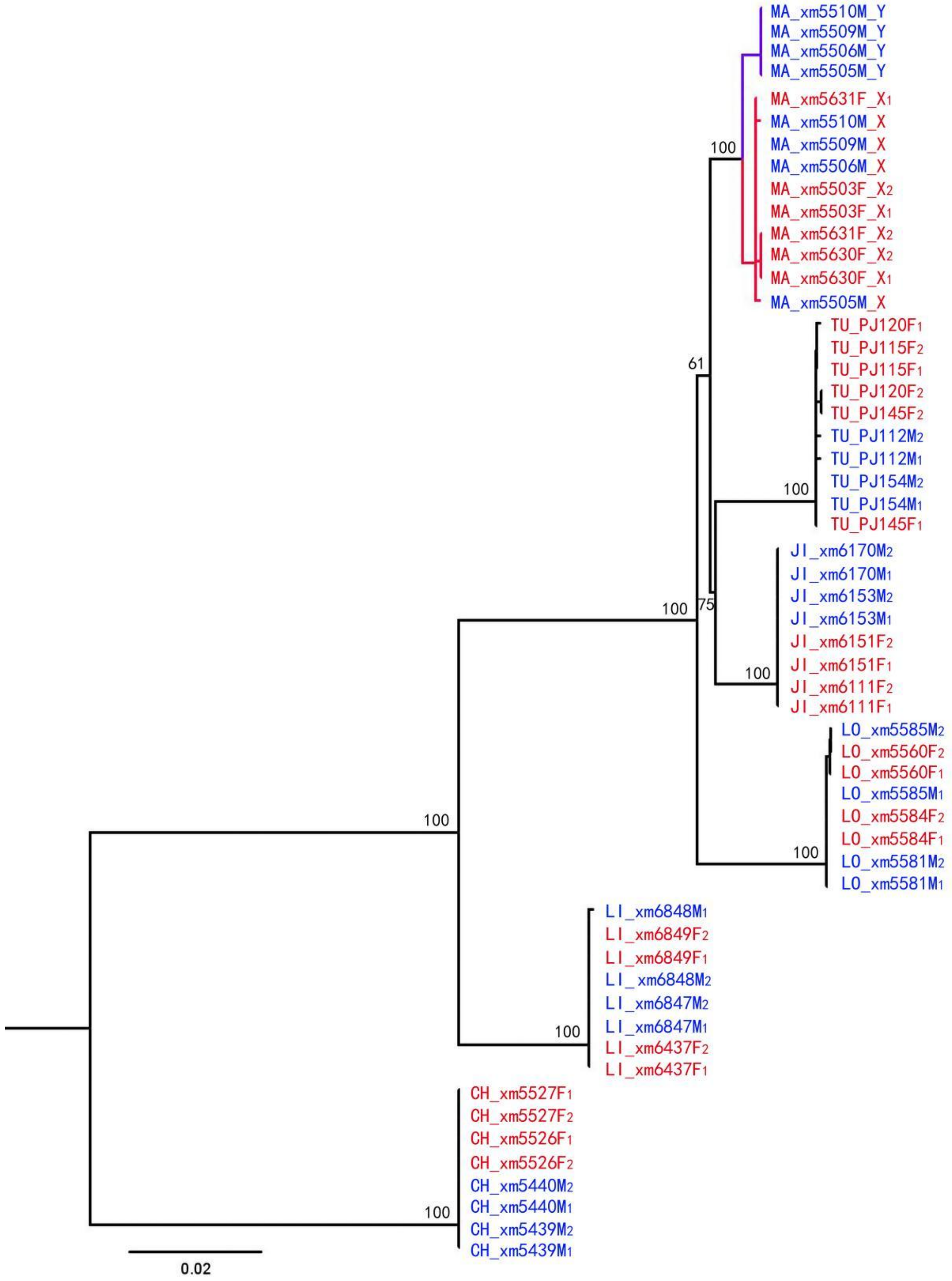
MA_xm5510M_Y
MA_xm5509M_Y
MA_xm5506M_Y
MA_xm5505M_Y
MA_xm5631F_X1
MA_xm5510M_X
MA_xm5509M_X
MA_xm5506M_X
MA_xm5503F_X2
MA_xm5503F_X1
MA_xm5631F_X2
MA_xm5630F_X2
MA_xm5630F_X1
MA_xm5505M_X
TU_PJ120F1
TU_PJ115F2
TU_PJ115F1
TU_PJ120F2
TU_PJ145F2
TU_PJ112M2
TU_PJ112M1
TU_PJ154M2
TU_PJ154M1
TU_PJ145F1
JI_xm6170M2
JI_xm6170M1
JI_xm6153M2
JI_xm6153M1
JI_xm6151F2
JI_xm6151F1
JI_xm6111F2
JI_xm6111F1
LO_xm5585M2
LO_xm5560F2
LO_xm5560F1
LO_xm5585M1
LO_xm5584F2
LO_xm5584F1
LO_xm5581M2
LO_xm5581M1
LI_xm6848M1
LI_xm6849F2
LI_xm6849F1
LI_xm6848M2
LI_xm6847M2
LI_xm6847M1
LI_xm6437F2
LI_xm6437F1
CH_xm5527F1
CH_xm5527F2
CH_xm5526F1
CH_xm5526F2
CH_xm5440M2
CH_xm5440M1
CH_xm5439M2
CH_xm5439M1
100
61
100
75
100
100
100
100
100
100
0.02

***A*. sp.**

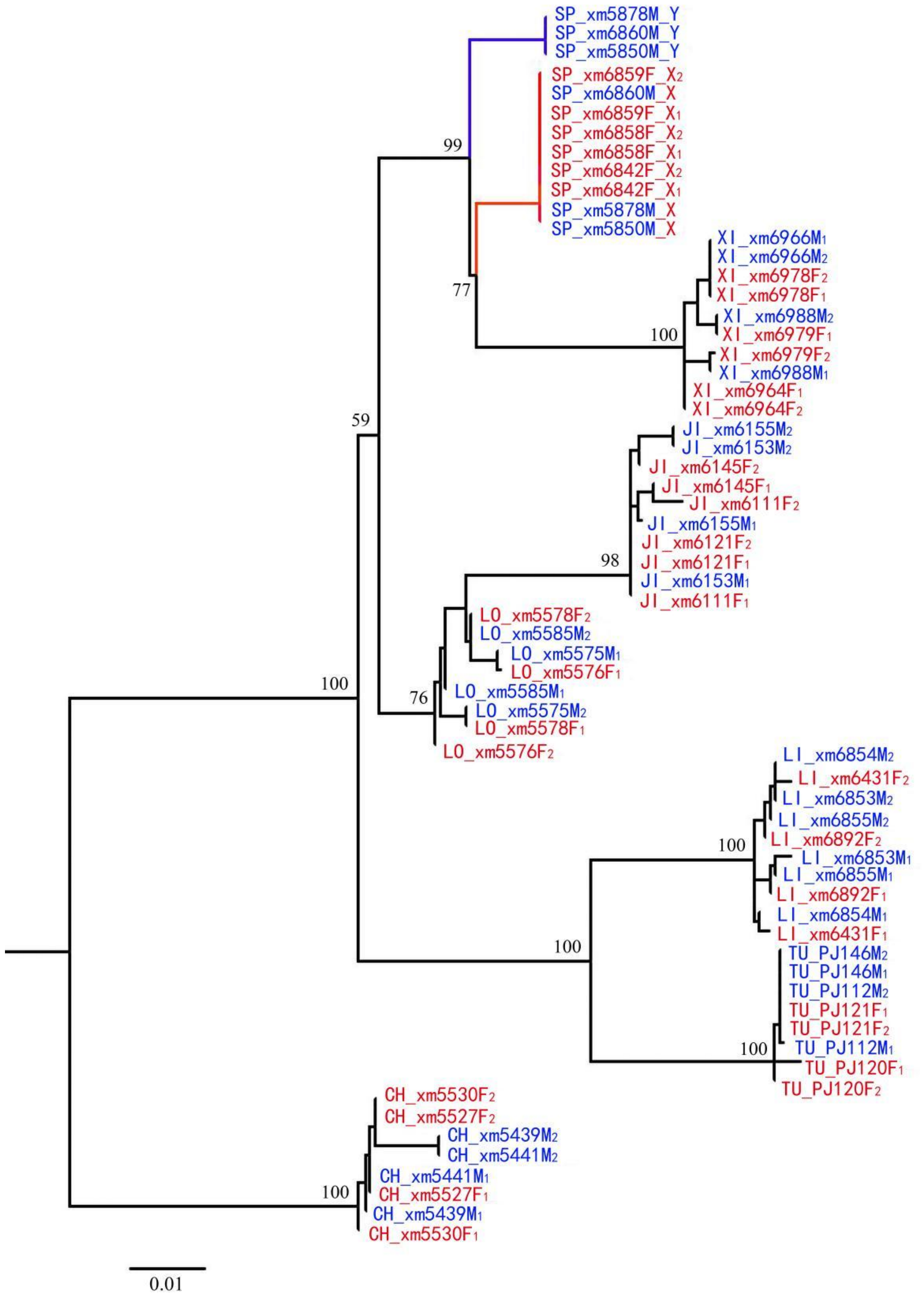

SP_xm5878M_Y
SP_xm6860M_Y
SP_xm5850M_Y
SP_xm6859F_X2
SP_xm6860M_X
SP_xm6859F_X1
SP_xm6858F_X2
SP_xm6858F_X1
SP_xm6842F_X2
SP_xm6842F_X1
SP_xm5878M_X
SP_xm5850M_X
99
77
XI_xm6966M1
XI_xm6966M2
XI_xm6978F2
XI_xm6978F1
XI_xm6988M2
XI_xm6979F1
XI_xm6979F2
XI_xm6988M1
XI_xm6964F1
XI_xm6964F2
100
59
JI_xm6155M2
JI_xm6153M2
JI_xm6145F2
JI_xm6145F1
JI_xm6111F2
JI_xm6155M1
JI_xm6121F2
JI_xm6121F1
JI_xm6153M1
JI_xm6111F1
98
LO_xm5578F2
LO_xm5585M2
LO_xm5575M1
LO_xm5576F1
LO_xm5585M1
LO_xm5575M2
LO_xm5578F1
LO_xm5576F2
100
76
LI_xm6854M2
LI_xm6431F2
LI_xm6853M2
LI_xm6855M2
LI_xm6892F2
LI_xm6853M1
LI_xm6855M1
LI_xm6892F1
LI_xm6854M1
LI_xm6431F1
100
100
TU_PJ146M2
TU_PJ146M1
TU_PJ112M2
TU_PJ121F1
TU_PJ121F2
TU_PJ112M1
TU_PJ120F1
TU_PJ120F2
100
CH_xm5530F2
CH_xm5527F2
CH_xm5439M2
CH_xm5441M2
CH_xm5441M1
CH_xm5527F1
CH_xm5439M1
CH_xm5530F1
100
0.01

A. tuberodepressus

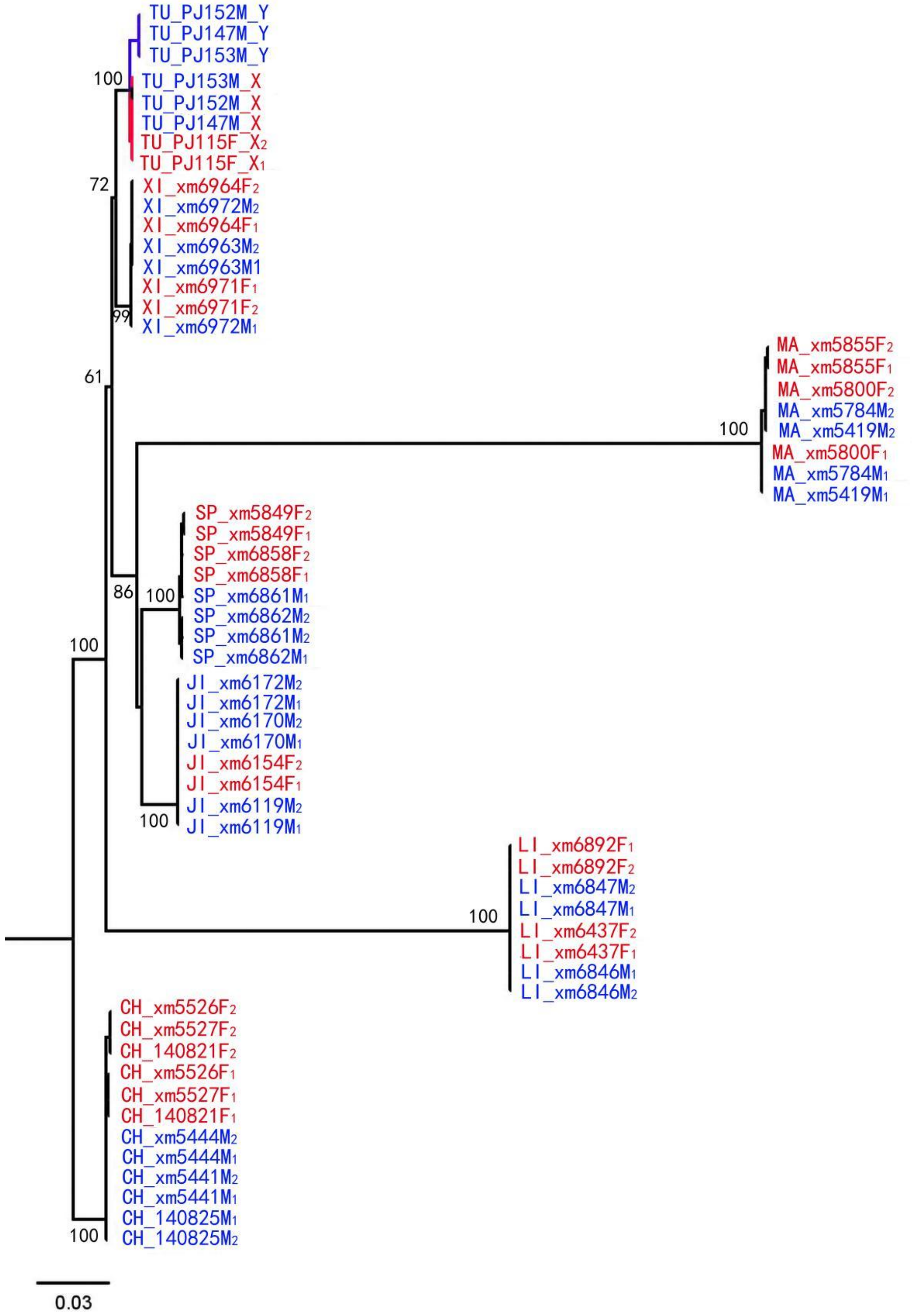

A. wuyiensis

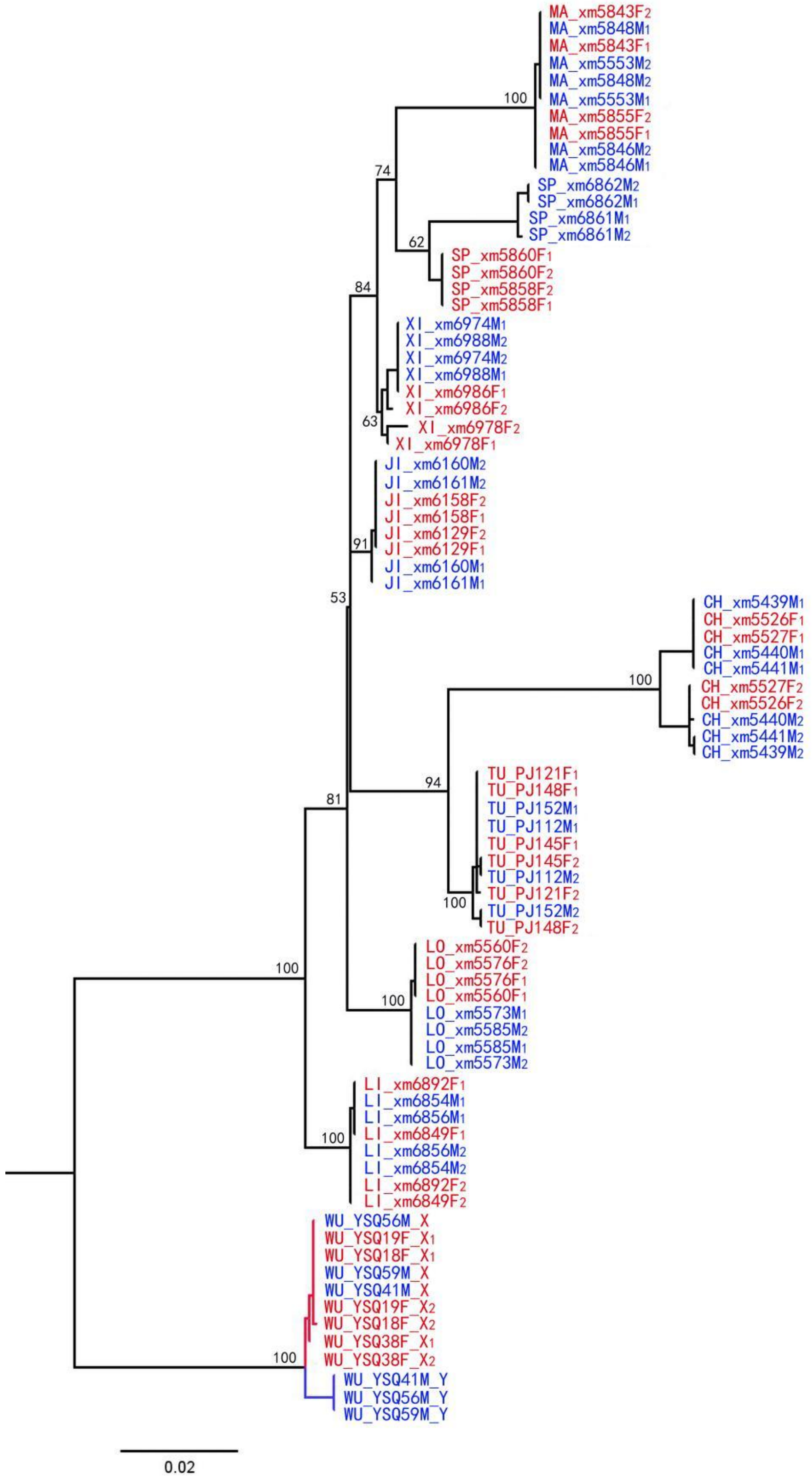
MA_xm5843F2
MA_xm5848M1
MA_xm5843F1
MA_xm5553M2
MA_xm5848M2
MA_xm5553M1
MA_xm5855F2
MA_xm5855F1
MA_xm5846M2
MA_xm5846M1
SP_xm6862M2
SP_xm6862M1
SP_xm6861M1
SP_xm6861M2
SP_xm5860F1
SP_xm5860F2
SP_xm5858F2
SP_xm5858F1
XI_xm6974M1
XI_xm6988M2
XI_xm6974M2
XI_xm6988M1
XI_xm6986F1
XI_xm6986F2
XI_xm6978F2
XI_xm6978F1
JI_xm6160M2
JI_xm6161M2
JI_xm6158F2
JI_xm6158F1
JI_xm6129F2
JI_xm6129F1
JI_xm6160M1
JI_xm6161M1
CH_xm5439M1
CH_xm5526F1
CH_xm5527F1
CH_xm5440M1
CH_xm5441M1
CH_xm5527F2
CH_xm5526F2
CH_xm5440M2
CH_xm5441M2
CH_xm5439M2
TU_PJ121F1
TU_PJ148F1
TU_PJ152M1
TU_PJ112M1
TU_PJ145F1
TU_PJ145F2
TU_PJ112M2
TU_PJ121F2
TU_PJ152M2
TU_PJ148F2
LO_xm5560F2
LO_xm5576F2
LO_xm5576F1
LO_xm5560F1
LO_xm5573M1
LO_xm5585M2
LO_xm5585M1
LO_xm5573M2
LI_xm6892F1
LI_xm6854M1
LI_xm6856M1
LI_xm6849F1
LI_xm6856M2
LI_xm6854M2
LI_xm6892F2
LI_xm6849F2
WU_YSQ56M_X
WU_YSQ19F_X1
WU_YSQ18F_X1
WU_YSQ59M_X
WU_YSQ41M_X
WU_YSQ19F_X2
WU_YSQ18F_X2
WU_YSQ38F_X1
WU_YSQ38F_X2
WU_YSQ41M_Y
WU_YSQ56M_Y
WU_YSQ59M_Y
100
74
62
84
63
91
53
100
94
100
81
100
100
100
100
0.02

A. xinduqiao

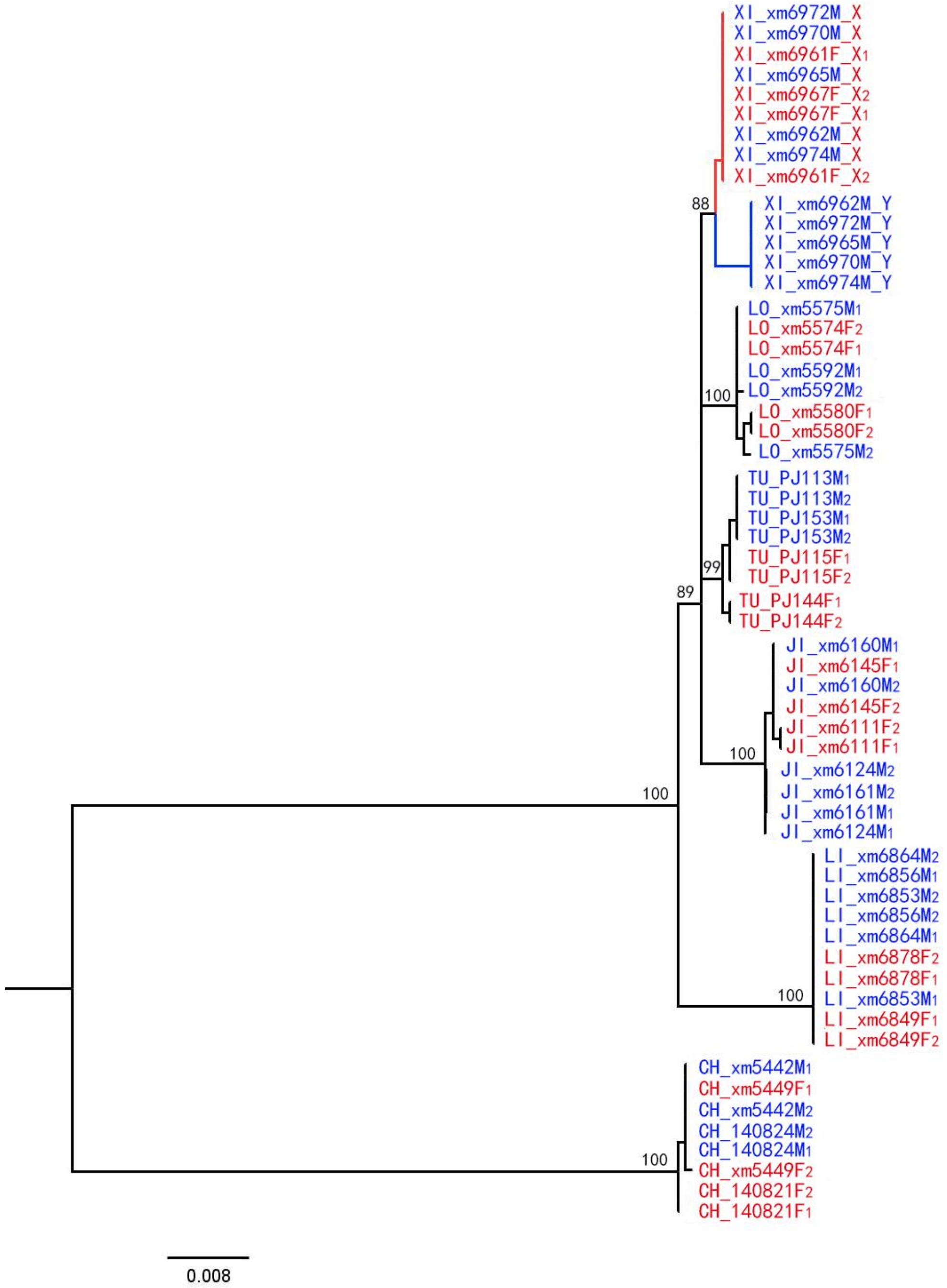
XI_xm6972M_X
XI_xm6970M_X
XI_xm6961F_X1
XI_xm6965M_X
XI_xm6967F_X2
XI_xm6967F_X1
XI_xm6962M_X
XI_xm6974M_X
XI_xm6961F_X2
88
XI_xm6962M_Y
XI_xm6972M_Y
XI_xm6965M_Y
XI_xm6970M_Y
XI_xm6974M_Y
LO_xm5575M1
LO_xm5574F2
LO_xm5574F1
LO_xm5592M1
LO_xm5592M2
100
LO_xm5580F1
LO_xm5580F2
LO_xm5575M2
TU_PJ113M1
TU_PJ113M2
TU_PJ153M1
TU_PJ153M2
TU_PJ115F1
99
TU_PJ115F2
89
TU_PJ144F1
TU_PJ144F2
JI_xm6160M1
JI_xm6145F1
JI_xm6160M2
JI_xm6145F2
JI_xm6111F2
JI_xm6111F1
100
JI_xm6124M2
100
JI_xm6161M2
JI_xm6161M1
JI_xm6124M1
LI_xm6864M2
LI_xm6856M1
LI_xm6853M2
LI_xm6856M2
LI_xm6864M1
LI_xm6878F2
LI_xm6878F1
LI_xm6853M1
100
LI_xm6849F1
LI_xm6849F2
CH_xm5442M1
CH_xm5449F1
CH_xm5442M2
CH_140824M2
CH_140824M1
100
CH_xm5449F2
CH_140821F2
CH_140821F1
0.008
