# Supplementary material for: Heterogeneous Evolution of Sex Chromosomes in the Torrent Frog Genus Amolops
Source: Int J Mol Sci. 2022 Sep 22;23(19):11146. doi: 10.3390/ijms231911146 (PMC9570394; doi:10.3390/ijms231911146)
Supplement: Supplementary file 1 [file ijms-23-11146-s001.zip › ijms-1904191-manuscript-supplementary-revised/manuscript-supplementary/File S9.pdf]

File S9

The information of five sex-linked markers and their cross-species homologous sequences in each species used to construct the maximum likelihood tree. The sequence alignment was completed using the *blastn* command, the homologous sequence of the sex-linked loci of one *Amolops* in other *Amolops* species was selected, and only the alignment result of e-value $\leqslant 1 \times 10^{-20}$ was retained.

Table S1. Homologous sequences of sex-linked loci of *A. chunganensis* in *A. jinjiangensis* data set

Sex-linked markers ID (*A. chunganensis*)	Locus ID (*A. jinjiangensis*)	Identical(%)	Evalue	Bitscore	Start (*A. chunganensis*)	End (*A. chunganensis*)	Start (*A. jinjiangensis*)	End (*A. jinjiangensis*)
461783	2110614	95.318	5.77E-120	436	1	298	298	1
697780	1030788	87.898	1.71E-95	355	1	306	1	298
775866	3460668	96.774	6.84E-129	466	1	279	1	279
1058318	1875209	97.436	6.68E-129	466	1	273	1	273
2189594	2443502	94.224	4.14E-116	424	1	277	277	1

Table S2. Homologous sequences of sex-linked loci of *A. chunganensis* in *A. lifanensis* data set

Sex-linked markers ID (*A. chunganensis*)	Locus ID (*A. lifanensis*)	Identical(%)	Evalue	Bitscore	Start (*A. chunganensis*)	End (*A. chunganensis*)	Start (*A. lifanensis*)	End (*A. lifanensis*)
461783	4145131	95.987	3.21E-123	448	1	298	298	1
697780	2752294	89.644	2.03E-105	388	1	306	1	298
775866	952435	96.774	8.23E-129	466	1	279	1	279
1058318	899717	97.436	6.21E-130	470	1	273	1	273
2189594	209343	91.636	3.04E-103	381	1	272	1	275

Table S3. Homologous sequences of sex-linked loci of *A. chunganensis* in *A. loloensis* data set

Sex-linked markers ID (*A. chunganensis*)	Locus ID (*A. loloensis*)	Identical(%)	Evalue	Bitscore	Start (*A. chunganensis*)	End (*A. chunganensis*)	Start (*A. loloensis*)	End (*A. loloensis*)
461783	1294504	94.04	2.15E-113	414	1	298	1	298
697780	104026	94.667	7.78E-123	446	11	306	298	1
775866	3345839	96.416	2.53E-127	460	1	279	279	1
1058318	2597914	96.715	1.15E-125	455	1	273	273	1
2189594	356968	91.367	9.34E-102	375	1	275	1	275

Table S4. Homologous sequences of sex-linked loci of *A. chunganensis* in *A. mantzorum* data set

Sex-linked markers ID (*A. chunganensis*)	Locus ID (*A. mantzorum*)	Identical(%)	Evalue	Bitscore	Start (*A. chunganensis*)	End (*A. chunganensis*)	Start (*A. mantzorum*)	End (*A. mantzorum*)
461783	2795554	93.709	2.52E-111	409	1	298	1	298
697780	1955749	87.048	1.22E-99	370	1	306	1	332
775866	5052676	96.774	1.38E-128	466	1	279	1	279
1058318	3100419	97.07	6.26E-127	460	1	273	1	273
2189594	4388276	90.909	1.43E-98	366	1	272	1	274

Table S5. Homologous sequences of sex-linked loci of *A. chunganensis* in *A.* sp. data set

Sex-linked markers ID (*A. chunganensis*)	Locus ID (*A.* sp.)	Identical(%)	Evalue	Bitscore	Start (*A. chunganensis*)	End (*A. chunganensis*)	Start (*A.* sp.)	End (*A.* sp.)
461783	977050	94.04	1.86E-113	414	1	298	1	298
697780	1767937	87.58	5.48E-94	350	1	306	298	1
775866	281433	96.774	4.71E-129	466	1	279	279	1
1058318	1644201	96.337	4.63E-124	449	1	273	273	1
2189594	1943477	94.224	2.85E-116	424	1	277	1	277

Table S6. Homologous sequences of sex-linked loci of *A. chunganensis* in *A. tuberodepressus* data set

Sex-linked markers ID (*A. chunganensis*)	Locus ID (*A. tuberodepressus*)	Identical(%)	Evalue	Bitscore	Start (*A. chunganensis*)	End (*A. chunganensis*)	Start (*A. tuberodepressus*)	End (*A. tuberodepressus*)
461783	2178382	93.709	2.21E-111	409	1	298	298	1
697780	1122835	94.175	6.24E-122	444	1	306	305	1
775866	4575332	96.416	1.55E-127	462	1	279	279	1
1058318	1126095	97.07	4.21E-128	464	1	273	1	273
2189594	122696	94.224	7.27E-116	424	1	277	277	1

Table S7. Homologous sequences of sex-linked loci of *A. chunganensis* in *A. xinduqiao* data set

Sex-linked markers ID (*A. chunganensis*)	Locus ID (*A. xinduqiao*)	Identical(%)	Evalue	Bitscore	Start (*A. chunganensis*)	End (*A. chunganensis*)	Start (*A. xinduqiao*)	End (*A. xinduqiao*)
461783	1482208	93.709	2.53E-111	409	1	298	1	298
697780	2979510	89.172	1.58E-103	383	1	306	303	1
775866	12127	96.416	6.45E-127	460	1	279	279	1
1058318	2685487	97.802	2.91E-130	472	1	273	1	273
2189594	3345455	93.502	1.82E-112	412	1	277	1	277

Table S8. Homologous sequences of sex-linked loci of *A. jinjiangensis* in *A. chunganensis* data set

Sex-linked markers ID (*A. jinjiangensis*)	Locus ID (*A. chunganensis*)	Identical(%)	Evalue	Bitscore	Start (*A. jinjiangensis*)	End (*A. jinjiangensis*)	Start (*A. chunganensis*)	End (*A. chunganensis*)
94572	1672562	89.109	2.07E-91	342	1	298	298	1
261570	1215526	94	4.33E-113	414	1	298	299	1
263214	426971	89.597	2.07E-91	342	1	298	1	298
458997	2072145	86.688	4.55E-78	298	1	298	1	298
1196026	661659	95.686	4.7E-112	411	1	255	1	255

Table S9. Homologous sequences of sex-linked loci of *A. jinjiangensis* in *A. lifanensis* data set

Sex-linked markers ID (*A. jinjiangensis*)	Locus ID (*A. lifanensis*)	Identical(%)	Evalue	Bitscore	Start (*A. jinjiangensis*)	End (*A. jinjiangensis*)	Start (*A. lifanensis*)	End (*A. lifanensis*)
94572	1304512	95.333	6.94E-120	436	1	298	1	298
261570	486972	89.644	7.25E-90	337	1	298	298	1
263214	1094956	94.631	1.5E-116	425	1	298	1	298
458997	2347274	94.098	3.25E-113	414	1	298	298	1
1196026	2216461	96.471	1.63E-115	422	1	255	1	255

Table S10. Homologous sequences of sex-linked loci of *A. jinjiangensis* in *A. loloensis* data set

Sex-linked markers ID (*A. jinjiangensis*)	Locus ID (*A. loloensis*)	Identical(%)	Evalue	Bitscore	Start (*A. jinjiangensis*)	End (*A. jinjiangensis*)	Start (*A. loloensis*)	End (*A. loloensis*)
94572	236816	93.709	9.92E-117	425	1	298	1	302
261570	1238477	89.037	2.23E-88	331	1	298	298	1
263214	710099	92.617	1.01E-106	392	1	298	298	1
458997	753555	93.464	2.15E-113	414	1	298	298	1
1196026	2261018	97.647	1.07E-120	438	1	255	255	1

Table S11. Homologous sequences of sex-linked loci of *A. jinjiangensis* in *A.* sp. data set

Sex-linked markers ID (*A. jinjiangensis*)	Locus ID (*A. sp.*)	Identical(%)	Evalue	Bitscore	Start (*A. jinjiangensis*)	End (*A. jinjiangensis*)	Start (*A. sp.*)	End (*A. sp.*)
94572	542643	97	1.82E-128	464	1	298	1	298
261570	2036540	84.158	2E-63	248	1	298	298	1
263214	2217973	98.993	1.8E-138	497	1	298	298	1
458997	76560	94.426	4E-115	420	1	298	298	1
1196026	1459014	97.647	9.28E-121	438	1	255	255	1

Table S12. Homologous sequences of sex-linked loci of *A. jinjiangensis* in *A. tuberodepressus* data set

Sex-linked markers ID (*A. jinjiangensis*)	Locus ID (*A. tuberodepressus*)	Identical(%)	Evalue	Bitscore	Start (*A. jinjiangensis*)	End (*A. jinjiangensis*)	Start (*A. tuberodepressus*)	End (*A. tuberodepressus*)
94572	1103762	97.333	9.99E-130	470	1	298	1	298
261570	472065	85.382	6.5E-72	278	1	298	1	298
263214	2556934	91.611	2.24E-101	375	1	298	298	1
458997	290229	94.098	1.02E-114	420	1	298	1	298
1196026	1957719	97.647	2.37E-120	438	1	255	255	1

Table S13. Homologous sequences of sex-linked loci of *A. jinjiangensis* in *A. xinduqiao* data set

Sex-linked markers ID (*A. jinjiangensis*)	Locus ID (*A. xinduqiao*)	Identical(%)	Evalue	Bitscore	Start (*A. jinjiangensis*)	End (*A. jinjiangensis*)	Start (*A. xinduqiao*)	End (*A. xinduqiao*)
94572	5270733	96.013	5.36E-123	448	1	298	1	298
261570	4392578	90.268	1.57E-88	333	1	292	298	12
263214	1053882	97.987	5.29E-133	481	1	298	1	298
458997	2054748	95.082	5.4E-118	431	1	298	1	298
1196026	1462974	97.647	2.73E-120	438	1	255	1	255

Table S14. Homologous sequences of sex-linked loci of *A. lifanensis* in *A. chunganensis* data set

Sex-linked markers ID (*A. lifanensis*)	Locus ID (*A. chunganensis*)	Identical(%)	Evalue	Bitscore	Start (*A. lifanensis*)	End (*A. lifanensis*)	Start (*A. chunganensis*)	End (*A. chunganensis*)
3479	71109	96.476	2.95E-100	372	1	224	227	1
174627	1203846	98.23	4.75E-48	198	1	113	298	186
409308	1221012	88.699	2.43E-90	339	1	273	292	1
520205	1050739	89.298	3.4E-104	385	1	298	292	1
974082	831512	97.993	4.21E-133	481	1	298	1	298

Table S15. Homologous sequences of sex-linked loci of *A. lifanensis* in *A. jinjiangensis* data set

Sex-linked markers ID (*A. lifanensis*)	Locus ID (*A. jinjiangensis*)	Identical(%)	Evalue	Bitscore	Start (*A. lifanensis*)	End (*A. lifanensis*)	Start (*A. jinjiangensis*)	End (*A. jinjiangensis*)
3479	1733626	98.667	3.04E-108	398	1	224	225	1
174627	1443356	98.23	2.97E-48	198	1	113	280	168
409308	920846	84.298	1.23E-56	226	4	237	16	253
520205	687381	92.617	1.24E-121	442	1	298	293	1
974082	564896	97.315	5.69E-130	470	1	298	298	1

Table S16. Homologous sequences of sex-linked loci of *A. lifanensis* in *A. mantzorum* data set

Sex-linked markers ID (*A. lifanensis*)	Locus ID (*A. mantzorum*)	Identical(%)	Evalue	Bitscore	Start (*A. lifanensis*)	End (*A. lifanensis*)	Start (*A. mantzorum*)	End (*A. mantzorum*)
3479	2258388	97.788	1.02E-105	390	1	224	226	1
174627	5267705	97.345	2.76E-46	193	1	113	395	283
409308	668200	81.513	3.26E-45	189	4	238	16	249
520205	2698178	94.389	1.16E-114	420	1	298	298	1
974082	905811	98.322	1.13E-134	486	1	298	1	298

Table S17. Homologous sequences of sex-linked loci of *A. lifanensis* in *A. tuberodepressus* data set

Sex-linked markers ID (*A. lifanensis*)	Locus ID (*A. tuberodepressus*)	Identical(%)	Evalue	Bitscore	Start (*A. lifanensis*)	End (*A. lifanensis*)	Start (*A. tuberodepressus*)	End (*A. tuberodepressus*)
3479	562102	98.673	4.09E-109	401	1	226	1	226
174627	5419129	95.385	8.65E-51	207	1	130	314	185
409308	1136600	81.818	2.2E-46	193	2	234	38	277
520205	3769210	93.729	2.21E-111	409	1	298	1	298
974082	669400	98.322	9.92E-135	486	1	298	298	1

Table S18. Homologous sequences of sex-linked loci of *A. lifanensis* in *A. xinduqiao* data set

Sex-linked markers ID (*A. lifanensis*)	Locus ID (*A. xinduqiao*)	Identical(%)	Evalue	Bitscore	Start (*A. lifanensis*)	End (*A. lifanensis*)	Start (*A. xinduqiao*)	End (*A. xinduqiao*)
3479	799857	98.667	6.11E-108	398	1	224	225	1
174627	2298766	96.85	2.13E-52	213	1	227	1	227
409308	1585742	84.211	6.95E-57	228	4	239	262	19
520205	1739894	94.389	1.17E-114	420	1	298	298	1
974082	525096	98.322	1.14E-134	486	1	298	298	1

Table S19. Homologous sequences of sex-linked loci of *A. loloensis* in *A. chunganensis* data set

Sex-linked markers ID (*A. loloensis*)	Locus ID (*A. chunganensis*)	Identical(%)	Evalue	Bitscore	Start (*A. loloensis*)	End (*A. loloensis*)	Start (*A. chunganensis*)	End (*A. chunganensis*)
108264	3403088	87.248	9.78E-80	303	1	298	298	1
287692	734673	95.667	1.99E-121	442	1	298	298	1
474811	429568	97.03	2E-131	475	1	303	1	298
771638	1612255	90.345	1.33E-43	183	1	144	304	167
1179745	1719370	96.644	1.97E-126	459	1	298	1	298

Table S20. Homologous sequences of sex-linked loci of *A. loloensis* in *A. jinjiangensis* data set

Sex-linked markers ID (*A. loloensis*)	Locus ID (*A. jinjiangensis*)	Identical(%)	Evalue	Bitscore	Start (*A. loloensis*)	End (*A. loloensis*)	Start (*A. jinjiangensis*)	End (*A. jinjiangensis*)
108264	112941	87.584	1.31E-81	309	1	298	1	298
287692	93880	99.664	1.21E-141	508	1	298	1	298
474811	402610	98.35	1.24E-136	492	1	303	1	299
771638	639274	98.658	1.21E-136	492	1	298	1	298
1179745	506343	98.993	2.61E-138	497	1	298	1	298

Table S21. Homologous sequences of sex-linked loci of *A. loloensis* in *A. lifanensis* data set

Sex-linked markers ID (*A. loloensis*)	Locus ID (*A. lifanensis*)	Identical(%)	Evalue	Bitscore	Start (*A. loloensis*)	End (*A. loloensis*)	Start (*A. lifanensis*)	End (*A. lifanensis*)
108264	3191293	86.622	1.59E-76	292	1	298	1	298
287692	2937545	96.667	1.48E-126	459	1	298	1	298
474811	2883	97.03	1.5E-131	475	1	303	298	1
771638	946832	97.368	4.61E-47	195	1	114	1	114
1179745	2505355	98.658	1.46E-136	492	1	298	298	1

Table S22. Homologous sequences of sex-linked loci of *A. loloensis* in *A. mantzorum* data set

Sex-linked markers ID (*A. loloensis*)	Locus ID (*A. mantzorum*)	Identical(%)	Evalue	Bitscore	Start (*A. loloensis*)	End (*A. loloensis*)	Start (*A. mantzorum*)	End (*A. mantzorum*)
108264	3815641	98.322	1.13E-134	486	1	298	298	1
287692	2130493	100	5.18E-143	514	1	298	1	298
474811	1044498	97.712	5.32E-138	497	1	303	305	1
771638	1031605	98.658	2.43E-136	492	1	298	1	298
1179745	5257305	95.833	1.63E-58	233	1	144	1	144

Table S23. Homologous sequences of sex-linked loci of *A. loloensis* in *A.* sp. data set

Sex-linked markers ID (*A. loloensis*)	Locus ID (*A.* sp.)	Identical(%)	Evalue	Bitscore	Start (*A. loloensis*)	End (*A. loloensis*)	Start (*A.* sp.)	End (*A.* sp.)
108264	1039898	96.98	1.82E-128	464	1	298	298	1
287692	857417	100	1.78E-143	514	1	298	1	298
474811	109312	98.68	5.09E-139	499	1	303	302	1
771638	3226069	97.368	2.64E-47	195	1	144	1	144
1179745	1221440	98.993	1.8E-138	497	1	298	298	1

Table S24. Homologous sequences of sex-linked loci of *A. loloensis* in *A. tuberodepressus* data set

Sex-linked markers ID (*A. loloensis*)	Locus ID (*A. tuberodepressus*)	Identical(%)	Evalue	Bitscore	Start (*A. loloensis*)	End (*A. loloensis*)	Start (*A. tuberodepressus*)	End (*A. tuberodepressus*)
108264	1512792	97.651	2.15E-131	475	1	298	1	298
287692	2789373	97.667	2.15E-131	475	1	298	1	298
474811	1279897	96.04	3.61E-139	501	1	303	299	1
771638	2021930	98.658	2.13E-136	492	1	298	298	1
1179745	2243614	96.364	4.75E-113	414	1	275	1	273

Table S25. Homologous sequences of sex-linked loci of *A. loloensis* in *A. xinduqiao* data set

Sex-linked markers ID (*A. loloensis*)	Locus ID (*A. xinduqiao*)	Identical(%)	Evalue	Bitscore	Start (*A. loloensis*)	End (*A. loloensis*)	Start (*A. xinduqiao*)	End (*A. xinduqiao*)
108264	536937	89.597	2.6E-91	342	1	298	298	1
287692	6866398	100	3.64E-35	156	1	84	1	84
474811	1908187	95.71	5.35E-138	497	1	303	1	300
771638	747170	98.658	2.44E-136	492	1	298	1	298
1179745	3216699	98.993	5.25E-138	497	1	298	298	1

Table S26. Homologous sequences of sex-linked loci of *A. mantzorum* in *A. chunganensis* data set

Sex-linked markers ID (*A. mantzorum*)	Locus ID (*A. chunganensis*)	Identical(%)	Evalue	Bitscore	Start (*A. mantzorum*)	End (*A. mantzorum*)	Start (*A. chunganensis*)	End (*A. chunganensis*)
240963	1902753	96.309	9.18E-125	453	1	298	1	298
305583	1394718	95.638	1.99E-121	442	1	298	298	1
328452	809288	96.309	9.18E-125	453	1	298	1	298
337208	817863	81.046	7.57E-61	241	1	291	1	298
650096	4523944	81.377	3.67E-49	202	1	232	1	245

Table S27. Homologous sequences of sex-linked loci of *A. mantzorum* in *A. jinjiangensis* data set

Sex-linked markers ID (*A. mantzorum*)	Locus ID (*A. jinjiangensis*)	Identical(%)	Evalue	Bitscore	Start (*A. mantzorum*)	End (*A. mantzorum*)	Start (*A. jinjiangensis*)	End (*A. jinjiangensis*)
240963	2294607	98.322	5.65E-135	486	1	298	1	298
305583	936978	97.987	2.63E-133	481	1	298	1	298
328452	1027974	99.329	5.61E-140	503	1	298	298	1
337208	826141	94.631	7.17E-129	466	1	291	298	1
650096	284428	95.38	5.77E-120	436	1	298	1	298

Table S28. Homologous sequences of sex-linked loci of *A. mantzorum* in *A. lifanensis* data set

Sex-linked markers ID (*A. mantzorum*)	Locus ID (*A. lifanensis*)	Identical(%)	Evalue	Bitscore	Start (*A. mantzorum*)	End (*A. mantzorum*)	Start (*A. lifanensis*)	End (*A. lifanensis*)
240963	141660	97.987	3.16E-133	481	1	298	298	1
305583	1731893	98.322	6.8E-135	486	1	298	298	1
328452	913415	97.651	1.47E-131	475	1	298	298	1
337208	1736778	81.373	4.4E-62	244	1	291	298	1
650096	2184090	95.973	3.21E-123	448	1	298	298	1

Table S29. Homologous sequences of sex-linked loci of *A. mantzorum* in *A. loloensis* data set

Sex-linked markers ID (*A. mantzorum*)	Locus ID (*A.loloensis*)	Identical(%)	Evalue	Bitscore	Start (*A. mantzorum*)	End (*A. mantzorum*)	Start (*A. loloensis*)	End (*A. loloensis*)
240963	1185812	94.295	4.62E-115	420	1	298	1	298
305583	448335	98.993	2.07E-138	497	1	298	298	1
328452	729770	98.322	4.49E-135	486	1	298	298	1
337208	709131	92.953	1.24E-120	438	1	291	1	298
650096	193647	97.987	2.09E-133	481	1	298	1	298

Table S30. Homologous sequences of sex-linked loci of *A. mantzorum* in *A. tuberodepressus* data set

Sex-linked markers ID (*A. mantzorum*)	Locus ID (*A.tuberodepressus*)	Identical(%)	Evalue	Bitscore	Start (*A. mantzorum*)	End (*A. mantzorum*)	Start (*A. tuberodepressus*)	End (*A. tuberodepressus*)
240963	392846	97.651	2.15E-131	475	1	298	298	1
305583	1295488	99.329	9.85E-140	503	1	298	298	1
328452	890034	97.987	4.62E-133	481	1	298	1	298
337208	1589583	94.966	2.71E-130	472	1	291	298	1
650096	1198691	95.333	1.01E-119	436	1	298	1	298

Table S31. Homologous sequences of sex-linked loci of *A.* sp. in *A. chunganensis* data set

Sex-linked markers ID (*A.* sp.)	Locus ID (*A. chunganensis*)	Identical(%)	Evalue	Bitscore	Start (*A.* sp.)	End (*A.* sp.)	Start (*A. chunganensis*)	End (*A. chunganensis*)
46910	2443439	90.429	4.39E-103	381	1	298	298	1
208821	3743191	91.946	9.24E-120	436	1	298	1	298
271488	771649	94.983	4.3E-118	431	1	298	298	1
1042367	4855160	94.098	1.2E-118	433	1	298	299	1
1413441	5846814	91.154	4.6E-98	364	1	251	1	260

Table S32. Homologous sequences of sex-linked loci of *A.* sp. in *A. jinjiangensis* data set

Sex-linked markers ID (*A.* sp.)	Locus ID (*A. jinjiangensis*)	Identical(%)	Evalue	Bitscore	Start (*A.* sp.)	End (*A.* sp.)	Start (*A. jinjiangensis*)	End (*A. jinjiangensis*)
46910	326494	95.05	2.69E-118	431	1	298	1	298
208821	1780957	98.997	2.61E-138	497	1	298	1	298
271488	396443	99.329	5.61E-140	503	1	298	1	298
1042367	1090232	96.644	2.63E-133	481	1	298	298	1
1413441	874094	92.459	1.29E-121	442	1	296	1	305

Table S33. Homologous sequences of sex-linked loci of *A.* sp. in *A. lifanensis* data set

Sex-linked markers ID (*A.* sp.)	Locus ID (*A. lifanensis*)	Identical(%)	Evalue	Bitscore	Start (*A.* sp.)	End (*A.* sp.)	Start (*A. lifanensis*)	End (*A. lifanensis*)
46910	2580114	88.321	1.59E-76	292	4	277	8	274
208821	857095	95.638	1.49E-121	442	1	298	298	1
271488	1607484	92.81	4.24E-107	394	1	296	298	2
1042367	733364	95.667	1.15E-127	462	1	297	297	1
1413441	3411267	91.586	4.32E-122	444	1	300	1	309

Table S34. Homologous sequences of sex-linked loci of *A.* sp. in *A. loloensis* data set

Sex-linked markers ID (*A.* sp.)	Locus ID (*A. loloensis*)	Identical(%)	Evalue	Bitscore	Start (*A.* sp.)	End (*A.* sp.)	Start (*A. loloensis*)	End (*A. loloensis*)
46910	1869549	94.389	4.62E-115	420	1	298	298	1
208821	533871	97.342	5.8E-134	483	1	298	1	300
271488	920489	98.993	2.07E-138	497	1	298	1	298
1042367	333574	96.98	4.49E-135	486	1	298	298	1
1413441	1397218	93.115	3.64E-126	457	1	296	1	305

Table S35. Homologous sequences of sex-linked loci of *A.* sp. in *A. tuberodepressus* data set

Sex-linked markers ID (*A.* sp.)	Locus ID (*A. tuberodepressus*)	Identical(%)	Evalue	Bitscore	Start (*A.* sp.)	End (*A.* sp.)	Start (*A. tuberodepressus*)	End (*A. tuberodepressus*)
46910	1903916	88.321	2.32E-76	292	4	277	8	274
208821	5191929	95.973	4.68E-123	448	1	298	298	1
271488	497483	98.322	9.92E-135	486	1	298	298	1
1042367	2260144	93.023	1.02E-114	420	1	298	1	298
1413441	2727	93.443	1.74E-127	462	1	296	1	305

Table S36. Homologous sequences of sex-linked loci of *A.* sp. in *A. xinduqiao* data set

Sex-linked markers ID (*A.* sp.)	Locus ID (*A. xinduqiao*)	Identical(%)	Evalue	Bitscore	Start (*A.* sp.)	End (*A.* sp.)	Start (*A. xinduqiao*)	End (*A. xinduqiao*)
46910	1650563	99.664	2.43E-141	508	1	298	298	1
208821	1672060	96.309	1.15E-124	453	1	298	1	298
271488	6970501	99.664	2.43E-141	508	1	298	298	1
1042367	6937050	98.322	2.43E-141	508	1	298	298	1
1413441	812136	91.532	4.5E-94	351	1	239	1	248

Table S37. Homologous sequences of sex-linked loci of *A. tuberodepressus* in *A. chunganensis* data set

Sex-linked markers ID (*A. tuberodepressus*)	Locus ID (*A. chunganensis*)	Identical(%)	Evalue	Bitscore	Start (*A. tuberodepressus*)	End (*A. tuberodepressus*)	Start (*A. chunganensis*)	End (*A. chunganensis*)
312165	1019332	98.188	1.07E-133	483	1	276	1	276
657724	2450248	94.631	2E-116	425	1	298	298	1
934386	2136270	97.015	3.17E-124	451	1	268	268	1
1219551	2042381	94.966	4.3E-118	431	1	298	298	1
2024518	500922	97.651	1.96E-131	475	1	298	298	1

Table S38. Homologous sequences of sex-linked loci of *A. tuberodepressus* in *A. jinjiangensis* data set

Sex-linked markers ID (*A. tuberodepressus*)	Locus ID (*A. jinjiangensis*)	Identical(%)	Evalue	Bitscore	Start (*A. tuberodepressus*)	End (*A. tuberodepressus*)	Start (*A. jinjiangensis*)	End (*A. jinjiangensis*)
312165	2667252	88.53	1.99E-89	335	1	276	279	1
657724	1416118	98.658	1.21E-136	492	1	298	298	1
934386	3160316	98.881	9.07E-133	479	1	268	1	268
1219551	601217	99.664	1.21E-141	508	1	298	1	298
2024518	1832768	97.987	2.63E-133	481	1	298	1	298

Table S39. Homologous sequences of sex-linked loci of *A. tuberodepressus* in *A. lifanensis* data set

Sex-linked markers ID (*A. tuberodepressus*)	Locus ID (*A. lifanensis*)	Identical(%)	Evalue	Bitscore	Start (*A. tuberodepressus*)	End (*A. tuberodepressus*)	Start (*A. lifanensis*)	End (*A. lifanensis*)
312165	1071496	98.188	2.24E-134	484	1	276	1	276
657724	2235918	87.625	9.31E-94	350	1	298	1	283
934386	224516	97.388	1.42E-126	459	1	268	1	268
1219551	516332	98.322	6.8E-135	486	1	298	298	1
2024518	2740849	98.322	6.8E-135	486	1	298	298	1

Table S40. Homologous sequences of sex-linked loci of *A. tuberodepressus* in *A. mantzorum* data set

Sex-linked markers ID (*A. tuberodepressus*)	Locus ID (*A. mantzorum*)	Identical(%)	Evalue	Bitscore	Start (*A. tuberodepressus*)	End (*A. tuberodepressus*)	Start (*A. mantzorum*)	End (*A. mantzorum*)
312165	725638	89.105	1.88E-82	313	1	257	1	255
657724	13714	82.623	7.4E-72	278	9	298	300	1
934386	841481	99.115	8.68E-111	407	1	226	1	226
1219551	1864040	91.852	3.57E-45	189	158	292	158	292
2024518	808415	98.658	2.43E-136	492	1	298	298	1

Table S41. Homologous sequences of sex-linked loci of *A. tuberodepressus* in *A.* sp. data set

Sex-linked markers ID (*A. tuberodepressus*)	Locus ID (*A.* sp.)	Identical(%)	Evalue	Bitscore	Start (*A. tuberodepressus*)	End (*A. tuberodepressus*)	Start (*A.* sp.)	End (*A.* sp.)
312165	1394266	87.097	2.31E-82	311	4	276	279	1
657724	635208	97.651	8.42E-132	475	1	298	298	1
934386	2610769	99.254	1.34E-134	484	1	268	1	268
1219551	797023	99.329	3.86E-140	503	1	298	298	1
2024518	2169423	97.987	1.81E-133	481	1	298	1	298

Table S42. Homologous sequences of sex-linked loci of *A. tuberodepressus* in *A. xinduqiao* data set

Sex-linked markers ID (*A. tuberodepressus*)	Locus ID (*A. xinduqiao*)	Identical(%)	Evalue	Bitscore	Start (*A. tuberodepressus*)	End (*A. tuberodepressus*)	Start (*A. xinduqiao*)	End (*A. xinduqiao*)
312165	5851835	98.913	6.28E-137	494	1	276	1	276
657724	818188	97.651	2.46E-131	475	1	298	1	298
934386	2692607	99.115	8.73E-111	407	1	226	1	226
1219551	687541	99.329	1.13E-139	503	1	298	298	1
2024518	3105988	98.322	1.14E-134	486	1	298	298	1

Table S43. Homologous sequences of sex-linked loci of *A. wuyiensis* in *A. chunganensis* data set

Sex-linked markers ID (*A. wuyiensis*)	Locus ID (*A. wuyiensis*)	Identical(%)	Evalue	Bitscore	Start (*A. wuyiensis*)	End (*A. wuyiensis*)	Start (*A. chunganensis*)	End (*A. chunganensis*)
201111	464239	92.787	1.22E-108	399	1	300	1	298
312243	1013678	89.333	3.62E-89	335	1	297	1	297
321845	1501634	90.333	9.58E-95	353	1	298	298	1
427217	1616476	86.897	4.55E-78	298	10	298	37	298
1146109	618153	93.624	2.01E-111	409	1	298	1	298

Table S44. Homologous sequences of sex-linked loci of *A. wuyiensis* in *A. jinjiangensis* data set

Sex-linked markers ID (*A. wuyiensis*)	Locus ID (*A. jinjiangensis*)	Identical(%)	Evalue	Bitscore	Start (*A. wuyiensis*)	End (*A. wuyiensis*)	Start (*A. jinjiangensis*)	End (*A. jinjiangensis*)
201111	558847	94.352	3.5E-117	427	1	300	1	298
312243	1075099	92.256	2.22E-104	385	1	297	1	297
321845	741431	90.604	1.29E-96	359	1	298	1	298
427217	1119478	96.528	1.75E-60	239	155	298	144	1
1146109	39416	93.289	1.63E-110	405	1	298	1	298

Table S45. Homologous sequences of sex-linked loci of *A. wuyiensis* in *A. lifanensis* data set

Sex-linked markers ID (*A. wuyiensis*)	Locus ID (*A. lifanensis*)	Identical(%)	Evalue	Bitscore	Start (*A. wuyiensis*)	End (*A. wuyiensis*)	Start (*A. lifanensis*)	End (*A. lifanensis*)
201111	709558	93.729	9.12E-114	416	1	300	1	298
312243	767578	86.452	3.5E-93	348	1	310	1	298
321845	2009742	90.604	1.55E-96	359	1	298	1	298
427217	2474073	95.833	9.78E-59	233	155	298	144	1
1146109	1132330	92.953	3.28E-108	398	1	298	1	298

Table S46. Homologous sequences of sex-linked loci of *A. wuyiensis* in *A. loloensis* data set

Sex-linked markers ID (*A. wuyiensis*)	Locus ID (*A. loloensis*)	Identical(%)	Evalue	Bitscore	Start (*A. wuyiensis*)	End (*A. wuyiensis*)	Start (*A. loloensis*)	End (*A. loloensis*)
201111	768012	93.115	7.78E-113	412	1	300	304	1
312243	432114	85.161	1.07E-86	326	1	310	1	298
321845	68653	90.268	4.75E-95	353	1	298	1	298
427217	1025827	96.528	1.39E-60	239	155	298	155	298
1146109	1354235	92.953	2.16E-108	398	1	298	1	298

Table S47. Homologous sequences of sex-linked loci of *A. wuyiensis* in *A. mantzorum* data set

Sex-linked markers ID (*A. wuyiensis*)	Locus ID (*A. mantzorum*)	Identical(%)	Evalue	Bitscore	Start (*A. wuyiensis*)	End (*A. wuyiensis*)	Start (*A. mantzorum*)	End (*A. mantzorum*)
201111	390044	90.365	1.18E-109	403	1	300	1	293
312243	-	-	-	-	-	-	-	-
321845	3388689	87.919	5.64E-83	315	1	298	1	298
427217	2394765	96.528	3.49E-60	239	155	298	144	1
1146109	2063369	92.953	5.45E-108	398	1	298	1	298

Table S48. Homologous sequences of sex-linked loci of *A. wuyiensis* in *A.* sp. data set

Sex-linked markers ID (*A. wuyiensis*)	Locus ID (*A.* sp.)	Identical(%)	Evalue	Bitscore	Start (*A. wuyiensis*)	End (*A. wuyiensis*)	Start (*A.* sp.)	End (*A.* sp.)
201111	427621	87.324	3.32E-71	274	32	300	276	1
312243	-	-	-	-	-	-	-	-
321845	935352	89.933	1.91E-93	348	1	298	1	298
427217	427217	96.528	1.2E-60	239	155	298	155	298
1146109	1928913	92.953	1.87E-108	398	1	298	1	298

Table S49. Homologous sequences of sex-linked loci of *A. wuyiensis* in *A. tuberodepressus* data set

Sex-linked markers ID (*A. wuyiensis*)	Locus ID (*A. tuberodepressus*)	Identical(%)	Evalue	Bitscore	Start (*A. wuyiensis*)	End (*A. wuyiensis*)	Start (*A. tuberodepressus*)	End (*A. tuberodepressus*)
201111	1956200	94.684	1.32E-118	433	1	300	298	1
312243	1215977	85.531	1.42E-93	350	1	310	1	307
321845	1782183	90.604	2.26E-96	359	1	298	1	298
427217	1114290	95.833	1.43E-58	233	155	298	144	1
1146109	519872	93.289	1.03E-109	403	1	298	298	1

Table S50. Homologous sequences of sex-linked loci of *A. wuyiensis* in *A. xinduqiao* data set

Sex-linked markers ID (*A. wuyiensis*)	Locus ID (*A. xinduqiao*)	Identical(%)	Evalue	Bitscore	Start (*A. wuyiensis*)	End (*A. wuyiensis*)	Start (*A. xinduqiao*)	End (*A. xinduqiao*)
201111	1122249	91.803	4.3E-104	385	1	300	298	1
312243	-	-	-	-	-	-	-	-
321845	3322531	90.268	1.2E-94	353	1	298	1	298
427217	4100399	96.528	3.51E-60	239	155	298	155	298
1146109	291437	92.617	1.52E-108	399	1	298	1	298

Table S51. Homologous sequences of sex-linked loci of *A. xinduqiao* in *A. chunganensis* data set

Sex-linked markers ID (*A. xinduqiao*)	Locus ID (*A.chunganensis*)	Identical(%)	Evalue	Bitscore	Start (*A. xinduqiao*)	End (*A. xinduqiao*)	Start (*A. chunganensis*)	End (*A. chunganensis*)
376827	1863739	97.651	1.96E-131	475	1	298	1	298
942805	114383	96.98	4.24E-128	464	1	298	1	298
946761	207420	91.129	3.72E-39	169	1	124	1	124
1829008	1784981	91.275	7.35E-101	374	6	298	4	298
2190627	1354952	92.453	2.62E-105	388	1	265	281	23

Table S52. Homologous sequences of sex-linked loci of *A. xinduqiao* in *A. jinjiangensis* data set

Sex-linked markers ID (*A. xinduqiao*)	Locus ID (*A. jinjiangensis*)	Identical(%)	Evalue	Bitscore	Start (*A. xinduqiao*)	End (*A. xinduqiao*)	Start (*A. jinjiangensis*)	End (*A. jinjiangensis*)
376827	590117	99.329	5.61E-140	503	1	298	1	298
942805	796025	98.658	1.21E-136	492	1	298	1	298
946761	676133	98.993	7.26E-139	499	1	298	298	1
1829008	585141	99.329	5.61E-140	503	1	298	1	298
2190627	859953	97.987	2.63E-133	481	1	298	1	298

Table S53. Homologous sequences of sex-linked loci of *A. xinduqiao* in *A. lifanensis* data set

Sex-linked markers ID (*A. xinduqiao*)	Locus ID (*A. lifanensis*)	Identical(%)	Evalue	Bitscore	Start (*A. xinduqiao*)	End (*A. xinduqiao*)	Start (*A. lifanensis*)	End (*A. lifanensis*)
376827	2182817	98.322	6.8E-135	486	1	298	1	298
942805	1677637	97.993	3.16E-133	481	1	298	298	1
946761	2271294	98.322	1.89E-135	488	1	298	1	298
1829008	557050	97.315	1.9E-130	472	1	298	298	1
2190627	603800	97.324	6.85E-130	470	1	298	1	298

Table S54. Homologous sequences of sex-linked loci of *A. xinduqiao* in *A. loloensis* data set

Sex-linked markers ID (*A. xinduqiao*)	Locus ID (*A. loloensis*)	Identical(%)	Evalue	Bitscore	Start (*A. xinduqiao*)	End (*A. xinduqiao*)	Start (*A. loloensis*)	End (*A. loloensis*)
376827	221452	98.658	9.65E-137	492	1	298	298	1
942805	1133695	99.664	9.58E-142	508	1	298	298	1
946761	540727	99.329	1.24E-140	505	1	298	298	1
1829008	911422	98.993	2.07E-138	497	1	298	298	1
2190627	1168692	98.993	2.07E-138	497	1	298	298	1

Table S55. Homologous sequences of sex-linked loci of *A. xinduqiao* in *A. tuberodepressus* data set

Sex-linked markers ID (*A. xinduqiao*)	Locus ID (*A. tuberodepressus*)	Identical(%)	Evalue	Bitscore	Start (*A. xinduqiao*)	End (*A. xinduqiao*)	Start (*A. tuberodepressus*)	End (*A. tuberodepressus*)
376827	1376827	98.881	1.47E-132	479	1	268	268	1
942805	869719	99.329	9.85E-140	503	1	298	298	1
946761	2074006	98.993	1.27E-138	499	1	298	298	1
1829008	2562629	98.993	4.58E-138	497	1	298	1	298
2190627	1612836	99.329	9.85E-140	503	1	298	298	1
