# Supplementary material for: Heterogeneous Evolution of Sex Chromosomes in the Torrent Frog Genus Amolops
Source: Int J Mol Sci. 2022 Sep 22;23(19):11146. doi: 10.3390/ijms231911146 (PMC9570394; doi:10.3390/ijms231911146)
Supplement: Supplementary file 1 [file ijms-23-11146-s001.zip › ijms-1904191-manuscript-supplementary-revised/manuscript-supplementary/Table S1.pdf]

Table S1. Summary of identification results of sex-specific markers in *Amolops* species from GBS datasets (Optimal parameters)

Species	Dataset	N Males	N Females	Sex-linked tags (SNPs) identified (XY / ZW)					System
				Frequency method	Heterozygosity method	Sex-specific method	Total	Confirmed	
A. chunganensis	GBS	12	8	409(658)/479(703)	409(658)/478(702)	22/10	429(658)/501(703)	32(37)/0	XY
A. jinjiangensis	GBS	10	11	111(177)/112(172)	111(177)/112(172)	298/35	409(177)/147(172)	10(13)/0	XY
A. lifanensis	GBS	12	8	151(277)/203(291)	151(277)/202(287)	47/31	198(277)/234(291)	9(10)/0	XY
A. loloensis	GBS	9	11	153(218)/144(260)	153(218)/144(260)	108/44	261(218)/188(260)	14(15)/0	XY
A. mantzorum	GBS	10	10	209(260)/18(26)	205(255)/18(26)	72/2	281(260)/20(26)	105(112)/0	XY
A. sp.	GBS	8	9	60(81)/14(16)	60(81)/14(16)	125/51	185/(81)/65(16)	6(7)/0	XY
A. tuberodepressus	GBS	12	12	451(674)/395(582)	451(674)/395(582)	23/317	474(674)/712(582)	44(53)/0	XY
A. wuyiensis	GBS	8	12	43(52)1/(8)	42(51)/1(8)	304/21	347(52)/22(8)	22(25)/0	XY
A. xinduqiao	GBS	10	10	211(304)/9(17)	211(304)/9(17)	26/0	237(304)/9(17)	134(173)/0	XY
