# Supplementary material for: Heterogeneous Evolution of Sex Chromosomes in the Torrent Frog Genus Amolops
Source: Int J Mol Sci. 2022 Sep 22;23(19):11146. doi: 10.3390/ijms231911146 (PMC9570394; doi:10.3390/ijms231911146)
Supplement: Supplementary file 1 [file ijms-23-11146-s001.zip › ijms-1904191-manuscript-supplementary-revised/manuscript-supplementary/Table S2.pdf]

Table S2. Summary of identification results of sex-specific markers in *Amolops* species from GBS and RAD-seq datasets (Parameters: M=2, n=1, m=2)

Species	Dataset	N Males	N Females	Sex-linked tags (SNPs) identified (XY / ZW)					System
				Frequency method	Heterozygosity method	Sex-specific method	Total	Confirmed	
A. chunganensis	GBS	12	8	736(1003)/755(1022)	736(1003)/753(1020)	53/28	789(1003)/783(1022)	67(82)/0	XY
A. jinjiangensis	GBS	10	11	244(395)/217(358)	244(395)/217(358)	749/185	993(395)/402(358)	15(16)/0	XY
A. jinjiangensis	RAD	3	4	181(264)/149(248)	181(264)/149(248)	58254/16207	58435(264)/16356(248)	23(29)/0	XY
A. lifanensis	GBS	12	8	314(427)/350(466)	314(427)/349(465)	197/70	511(427)/420(466)	28(31)/0	XY
A. loloensis	GBS	9	11	392(527)/350(468)	392(527)/350(468)	380/119	772(527)/469(468)	33(35)/0	XY
A. mantzorum	GBS	10	10	327(407)/36(50)	322(402)/36(50)	129/7	456(407)/43(50)	162(185)/0	XY
A. sp.	GBS	8	9	155(203)/78(105)	155(203)/78(105)	438/153	593(203)/231(105)	18(20)/0	XY
A. sp.	RAD	5	4	100(154)/195(241)	100(154)/195(241)	18657/4058	18757(154)/4253(241)	14(28)/0	XY
A. tuberodepressus	GBS	12	12	723(969)/662(908)	722(968)/662(908)	42/514	765(969)/1176(908)	48(62)/0	XY
A. wuyiensis	GBS	8	12	123(152)/7(8)	123(152)/9(10)	839/62	962(152)/71(10)	58(67)/0	XY
A. xinduqiao	GBS	10	10	490(645)/23(30)	490(645)/23(30)	68/20	558(645)/43(30)	307(373)/0	XY
