# Supplementary material for: Heterogeneous Evolution of Sex Chromosomes in the Torrent Frog Genus Amolops
Source: Int J Mol Sci. 2022 Sep 22;23(19):11146. doi: 10.3390/ijms231911146 (PMC9570394; doi:10.3390/ijms231911146)
Supplement: Supplementary file 1 [file ijms-23-11146-s001.zip › ijms-1904191-manuscript-supplementary-revised/manuscript-supplementary/Table S3.pdf]

Table S3. Details of sequencing datasets for the nine species analysed

Species	Dataset	N Males	N Females	Sex-linked tags (SNPs) identified (XY / ZW)				N of PCR marker	System	Sex chromosome
				Frequency method	Heterozygosity method	Sex-specific method	Confirmed			
A. chunganensis	GBS	12	8	1058(1554)/1155(1633)	1058(1554)/1152(1630)	75/38	89(108)/0	2	XY	Chr 05
A. jinjiangensis	GBS+RAD	10+3	11+4	509(789)/451(748)	509(789)/451(748)	59282/16427	45(113)/0	2	XY	Chr 05
A. lifanensis	GBS	12	8	436(669)/553(757)	436(669)/551(752)	241/99	35(39)/0	3	XY	Chr 05
A. loloensis	GBS	9	11	526(725)/474(698)	526(725)/474(698)	484/163	39(41)/0	1	XY	Chr 05
A. mantzorum	GBS	10	10	369(473)/49(71)	363(467)/49(71)	201/9	190(211)/0	1	XY	Chr 05
*A.*sp.	GBS+RAD	8+5	9+4	303(426)/285(360)	303(426)/285(360)	19217/4261	36(53)/0	5	XY	Chr 05
A. tuberodepressus	GBS	12	12	1095(1556)/990(1405)	1094(1555)/990(1405)	65/831	82(104)/0	6	XY	Chr 05
A. wuyiensis	GBS	8	12	146(179)/8(16)	145(178)/10(18)	1131/83	65(75)/0	7	XY	?
A. xinduqiao	GBS	10	10	542(729)/31(46)	542(729)/31(46)	90/20	343(423)/0	3	XY	Chr 05
