# Supplementary material for: Heterogeneous Evolution of Sex Chromosomes in the Torrent Frog Genus Amolops
Source: Int J Mol Sci. 2022 Sep 22;23(19):11146. doi: 10.3390/ijms231911146 (PMC9570394; doi:10.3390/ijms231911146)
Supplement: Supplementary file 1 [file ijms-23-11146-s001.zip › ijms-1904191-manuscript-supplementary-revised/manuscript-supplementary/Table S6.pdf]

Table S6. Summary of primers for identification of sex-specific SNP markers in *Amolops* species

Locus	Primer sequence (5′–3′)	T (°C)	length (bp)	SNP (X/Y)	SNP (Location)
CH_1058318	Forward: ATTGAAACTTGATTACCG<br>Reverse: ATTACTGATGGAGCCTGT	58	245	C/T	76
CH_2909840	Forward: TGAGATAAATGCCCAGGTG<br>Reverse: GCAGATAGAGCAAAACAGGA	58	211	A/G	105
JI_483348	Forward: ACAGCATGTGACTGAAATC<br>Reverse: CTGGTATCATCCAAGAAAA	58	271	A/G	83
JI_1184383	Forward: ATGGTTCATTTAGTCTAAGGGGATA<br>Reverse: CCCGCAACATCTTCTCCC	58	203	T/A	36
LI_174627	Forward: GTATGCTTTGGGTCGTT<br>Reverse: GAATGTGGTTCAGGCTAT	58	234	C/A	171
LI_375069	Forward: ATTGTGGAACCCGACG<br>Reverse: ACCCCTGTGGGACTGT	58	282	G/T	195
LI_430733	Forward: TCTCCAGCAGTTTTCATT<br>Reverse: TCCCCTTCTTTTATTGTC	58	221	G/C	83_130
LO_550969	Forward: GGGGATTTCAGCAACACTTAGG<br>Reverse: GTCCGTTCCAGCCCACCT	58	222	C/T	61
MA_85869	Forward: CTCCTGGCAACTCCTTTCTG<br>Reverse: GAGCCCATATTCAATTCACC	58	189	G/A	228
SP_110086	Forward: GGGAAACGGCCCTTGATA<br>Reverse: GAAGCACATCTCCCAGTCC	58	248	A/G	227
SP_208821	Forward: CGCTTTACACTTCATGCCCA<br>Reverse: AGAGTTGGGGAAGATGCCAA	58	211	C/T_C/G	193_217

SP_271488	Forward: CGCCAATCCCTTCACCTC<br>Reverse: TACAAACAAAGCACTTCCA	58	225	C/T	248
SP_476103	Forward: ATTGACTTTATGCTGTTGGGCTGCTA<br>Reverse: TATTGTTGTAGGGGATTGCAGAGCG	58	253	C/A	265
SP_1399857	Forward: AGATTTCTTCCCGCACTACTTT<br>Reverse: TAATTACAAGTACCTGAATGTGACC	58	284	C/T	271
TU_1007365	Forward: CCCACCAGACAGGAAACAC<br>Reverse: CCGCAGCAATGCCTAAAC	58	222	T/G_A/T	239_251
TU_1150683	Forward: CAGATTTTGGGGTGTAGA<br>Reverse: ATATTAGATAGATCAGCTTGG	58	265	C/A_G/A	230_242
TU_1390631	Forward: GGAAAGAACCAGATGACA<br>Reverse: AGAGTTGAACTCCAGCCT	58	176	T/C	186
TU_1950702	Forward: ATACCAGAGCTCCAGACC<br>Reverse: ATGACCCAATGTAGATGC	58	273	A/G	77
TU_2024518	Forward: ATCTTTGTGCAATCTTTGAT<br>Reverse: CACCCTCTTCCAGTCCAG	58	253	G/A_T/G	202_253
TU_2049833	Forward: TGTCTTCCTGAGTATCTGC<br>Reverse: AATAGTGCCACAACCTTT	58	257	G/A_A/T	238_249
WU_117656	Forward: TGCCTGTGAAATACGAC<br>Reverse: GAACACCCAGTTTGATAA	58	250	C/T	105
WU_120446	Forward: AGCCTACAAATAAAACAC<br>Reverse: ATACTACATCAAATAACAGAG	58	271	C/A	193
WU_177528	Forward: GCAGTAAAATATGCATCTAAC<br>Reverse: CTAAACACCTTCACTTGGA	58	234	G/A	160

WU_321845	Forward: CTACCGCCAAGACGGAAAG<br>Reverse: CGGCTGACAGAAAAGCAGAGT	58	266	G/A	136
WU_375485	Forward: TAACAATGTCCCGAATG<br>Reverse: TATGCGGAGATGAGAAA	58	288	G/A	115
WU_450377	Forward: GGAATGTGCTTGTATGCT<br>Reverse: ACTAATCAAAGTGCCAGG	58	218	A/G	82
WU_1146109	Forward: GAAACCCTCACGATTCTATT<br>Reverse: AGCCAGTCTCCCTCCAT	58	230	A/C	233
XI_106254	Forward: GCGCATACGCTCTGAAGG<br>Reverse: TCCAACTGCCAGCCACCA	58	215	G/A	173
XI_746953	Forward: AAGTGGATGTAAACCCGATTC<br>Reverse: AGATGATGCCCTCCAGACC	58	229	G/A	82
XI_1266715	Forward: ACCTTGGAACGAAGCCCTCA<br>Reverse: GCATGACTCCCAAAGGCAGAG	58	261	G/A	176

Note: The forward and reverse primers were designed at conserved region. T (°C) indicates the annealing temperature of PCR thermal cycling. Length indicates PCR product size. SNP (Location) represents the nucleotide site within the locus, reported using a zero-based offset (first nucleotide is enumerated as 0). In the Locus column, CH for *A. chunganensis*, JI for *A. jinjiangensis*, LI for *A. lifanensis*, LO for *A. loloensis*, MA for *A. mantzorum*, SP for *A.* sp., TU for *A. tuberodepressus*, WU for *A. wuyiensis*, XI for *A. xinduqiao*, and the number represents the locus ID in the catalog.
