# Supplementary material for: Heterogeneous Evolution of Sex Chromosomes in the Torrent Frog Genus Amolops
Source: Int J Mol Sci. 2022 Sep 22;23(19):11146. doi: 10.3390/ijms231911146 (PMC9570394; doi:10.3390/ijms231911146)
Supplement: Supplementary file 1 [file ijms-23-11146-s001.zip › ijms-1904191-manuscript-supplementary-revised/manuscript-supplementary/Table S7.pdf]

Table S7. Matching nucleotide alignment of the blast hit results of sex-linked loci from the NCBI database.

Species	Locus	Length (bp)	NCBI Hit	Query Cover	E-value	identity	start	end
A. jinjiangensis	1320445	320	*Rana rugosa* *CYP19A1* gene for cytochrome P-450 aromatase, promoter region and partial cds (AB379847.1)	38%	1e-19	83%	16209	16392
A. tuberodepressus	131205	299		97%	2e-52	80.69%	14107	14424
A. mantzorum	28208	299		48%	2e-33	86.11%	12255	12398
A. mantzorum	207705	301		41%	6e-28	86.51%	14112	14235
A. mantzorum	651335	298		38%	2e-33	91.23%	14107	14219
A. mantzorum	852213	304		38%	8e-22	84.55%	11703	11824
A. mantzorum	946208#	280		95%	6e-53	81.82%	11703	11997
A. mantzorum	973436	298		58%	2e-42	86.19%	14247	14424
A. mantzorum	1306289	257		98%	3e-60	84.13%	14150	14419
A. mantzorum	1628703	270		69%	1e-39	89.36%	14107	14246
A. mantzorum	1784854#	299		21%	6e-13	92.06%	14151	14212
A. xinduqiao	546456	264		90%	9e-31	78.01%	10170	10407
A. xinduqiao	708316	265		41%	2e-27	88.70%	10268	10382
A. xinduqiao	376827	298		33%	2e-17	85.44%	10245	10344
A. xinduqiao	694936	298		47%	3e-11	77.08%	10264	10407
A. wuyiensis	764303	298	*Rana rugosa* *AR* gene for androgen receptor, promoter, partial sequence (AB491761.1)	11%	1e-04	97.14%	612	646

Note: Locus with # represent sex-linked loci was consistent with those identified by Luo et al. 2020 [34].
